# Exercise training modalities for cardiorespiratory fitness and blood pressure in children and adolescents with obesity: a systematic review and network meta-analysis

**DOI:** 10.3389/fpubh.2026.1842123

**Published:** 2026-05-20

**Authors:** Xinyin Wang, Renchao Lv, Bin Hou

**Affiliations:** 1 School of Physical Education and Sport, Central China Normal University, Wuhan, China; 2 School of Physical Education and Sport, Kunming University, Kunming, China

**Keywords:** adolescents, blood pressure, cardiopulmonary function, exercise training, intervention, obesity

## Abstract

**Background:**

The optimal exercise modality for improving cardiorespiratory fitness and blood pressure in children and adolescents with obesity remains uncertain.

**Methods:**

We conducted a systematic review and network meta-analysis of randomized controlled trials comparing exercise interventions in individuals aged 5–19 years with obesity. PubMed, Embase, Cochrane Library, and Web of Science were searched from inception to December 27, 2025. Outcomes included VO_2_peak, VO_2_max, maximal aerobic speed (MAS), systolic blood pressure (SBP), diastolic blood pressure (DBP), maximal heart rate (HRmax), and resting heart rate (RHR). Random-effects models were applied, interventions were ranked using SUCRA, and evidence certainty was assessed using CINeMA.

**Results:**

Forty-five trials involving 2,635 participants were included. For cardiorespiratory fitness, HIIT significantly improved VO_2_peak (MD 3.81 mL/kg/min, 95% CI 2.41 to 5.22) and MAS (MD 1.22 m/s, 95% CI 0.75 to 1.68) versus SOC, and ranked highest for both outcomes. HIIT combined with MICT was most likely to improve VO_2_max (MD 5.20 mL/kg/min, 95% CI 0.28 to 10.12). Combined high-intensity and resistance-based strategies also showed favorable effects. For cardiovascular outcomes, MICT was most effective for lowering SBP (MD −7.75 mmHg, 95% CI −13.08 to −2.42), whereas MIIT showed the greatest reduction in DBP (MD −4.03 mmHg, 95% CI −6.74 to −1.32). No major global inconsistency was detected. Certainty of evidence ranged from high to very low.

**Conclusions:**

High-intensity exercise modalities, especially HIIT and combined high-intensity protocols, may provide the greatest benefit for cardiorespiratory fitness in youths with obesity, while moderate-intensity exercise appears more effective for blood pressure reduction. Exercise prescriptions in pediatric obesity should therefore be individualized according to target outcomes.

## 1 Introduction

Obesity in children and adolescents has reached alarming levels and is now a major global public health challenge of the 21st century. A 2025 joint analysis by UNICEF, the World Health Organization, and the World Bank estimated that, among individuals aged 5–19 years, one in five were overweight, accounting for approximately 391 million children and adolescents worldwide. Notably, for the first time, obesity prevalence among school-aged children and adolescents surpassed underweight prevalence across most world regions, with around one in ten classified as obese, corresponding to 188 million individuals (1). Childhood obesity is linked to multiple short-term comorbidities, including type 2 diabetes, metabolic dysfunction-associated fatty liver disease, dyslipidemia, hypertension, obstructive sleep apnea, and mental health disorders, and it also increases the risk of cardiovascular disease, cardiovascular mortality, and all-cause mortality later in life (2–4). Cardiorespiratory fitness (CRF), one of the central components of physical fitness, is commonly indexed by maximal oxygen uptake (VO_2_max) or metabolic equivalents (METs). Low CRF is associated with cardiovascular disease (CVD), cancer, and increased morbidity and mortality (5). Children with overweight or obesity tend to have lower CRF, which may in turn elevate their risk of cardiovascular disease in adulthood (6).

Current approaches to the management of adolescent obesity include pharmacotherapy, metabolic and bariatric surgery (MBS), nutrition education, dietary intervention, exercise training, and combined dietary and exercise interventions. However, responses to pharmacotherapy and MBS appear to be highly heterogeneous (7). Among the available strategies, combined dietary and exercise intervention appears to offer the greatest overall benefit. The World Health Organization guidelines on physical activity recommend at least 60 min of moderate-to-vigorous physical activity on average each day, which can improve cardiorespiratory health, lower body fat percentage, and optimize body composition (8). Exercise modalities for adolescents with obesity include MICT, HIIT, LICT, and resistance training. MICT improves cardiometabolic health and may lower blood pressure; HIIT more strongly enhances VO_2_max/VO_2_peak and energy expenditure but induces greater fatigue; LICT enables low-joint-stress energy expenditure; resistance training improves cardiovascular function, increases muscle mass, and reduces body fat.Common indicators used to assess cardiorespiratory function include VO_2_max, VO_2_peak, SBP, DBP, maximal aerobic speed (MAS), HR_max_, and RHR (9).

In addition, direct head-to-head comparisons are limited. Accordingly, this study used a network meta-analysis to compare available exercise modalities indirectly and to identify the most effective intervention for cardiopulmonary function in adolescents with obesity.

## 2 Materials and methods

This network meta-analysis (NMA) was conducted in accordance with the Preferred Reporting Items for Systematic Reviews and Meta-Analyses extension for network meta-analyses (PRISMA-NMA; Supplementary Table 1) (10). To ensure transparency, rigor, and methodological integrity, the study protocol was prospectively registered in the PROSPERO database (CRD420261348002).

### 2.1 Data sources and search strategy

We systematically searched PubMed, EMBASE, the Cochrane Library, and Web of Science databases and the SPORTDiscus database. The search strategy combined free-text terms and Medical Subject Headings (MeSH) and included the following keywords: “obesity,” “overweight,” “child,” “children,” “blood pressure,” “exercise,” “physical activity,” “cardiopulmonary fitness,” “heart rate,” “VO_2_max,” “VO_2_peak,” “SBP,” “DBP,” and “randomized controlled trial.” The search covered the entire database period up to April 17, 2026, without language restrictions.

### 2.2 Selection criteria

(1) Children and adolescents aged 5–19 years with overweight or obesity, defined according to CDC standards (overweight was defined as a BMI-for-age at or above the 85th percentile and below the 95th percentile, and obesity as a BMI-for-age at or above the 95th percentile.) (11).

(2) Randomized controlled trials (RCTs) evaluating exercise interventions, including high-intensity interval training (HIIT), moderate-intensity continuous training (MICT), moderate-intensity interval training (MIIT), low-intensity continuous training (LICT), resistance training (RT), or combinations thereof.

(3) RCTs assessing alternative interventions, such as standard of care (SOC), nutrition education, or non-exercise interventions.

(4) RCTs reporting at least one of the following outcomes: Maximum oxygen uptake(VO_2_max), ppeka oxygen uptake(VO_2_peak), maximal aerobic speed (MAS), systolic blood pressure (SBP), diastolic blood pressure (DBP), maximal heart rate (HR_max_), or resting heart rate (RHR).

Exclusion criteria:

(1) RCTs conducted at multiple time points in the same cohort.

(2) RCTs that did not report the required outcome measures.

(3) Reviews, case reports, retrospective studies, or observational studies.

Prior to full-text screening, studies were initially assessed based on titles and abstracts. All included RCTs were independently verified by two reviewers to ensure that the most recently published data were used.

### 2.3 Exercise intensity classification

Aerobic exercise (AE) refers to physical activity performed under conditions of sufficient oxygen availability. According to the American College of Sports Medicine (12), the intensity of AE is determined using maximal heart rate (HR_max_), metabolic equivalents (METs), percentage of heart rate reserve (HRR), the rating of perceived exertion (RPE), and the talk test. Exercise intensity is categorized as follows:

• Low intensity: 1.5–3.0 METs, ≤ 50% HR_max_, RPE 10–11, HRR < 40%, allowing effortless conversation during activity.

• Moderate intensity: 3.0–6.0 METs, 55–69% HR_max_, 40– < 60% HRR, RPE 12–13, permitting conversation but not singing comfortably.

• High intensity: ≥6.0 METs, 60–89% HRR, 76–96% HR_max_, RPE 15–17, with continuous speech difficult.

When an exercise program specifies a target intensity, participants were classified according to these thresholds (e.g., moderate intensity: 55–69% HR_max_) . Programs with overlapping intensity ranges but a clearly defined progression toward a target intensity were classified according to the higher intended intensity. If the intensity overlap did not meet the moderate-intensity criteria (e.g., HR_ma_x ≤ 50%), the program was classified as low intensity

### 2.4 Data extraction and quality assessment

Data from the randomized controlled trials were extracted independently by the investigators in accordance with PRISMA guidelines, and any disagreements were resolved through discussion with a second author. The extracted variables included first author, publication year, sample size, participants' age, sex, and geographic distribution, intervention duration and frequency, and the intervention protocols for the experimental and control groups. For continuous outcomes, When available, data on the mean change from baseline and its corresponding standard deviation (SD) were extracted. If only baseline and post-intervention means and SDs were reported, the mean change was calculated as the difference between the two time points. The SD of the change score was then estimated from the baseline SD, post-intervention SD, and correlation coefficient (R) using the following formula.


$$\begin{array}{l} SDchange=\sqrt{Baseline\;SD^{2}+Endpoint\;SD^{2}-2R\cdot Baseline\;SD\cdot Endpoint\;SD} \end{array}$$

If the correlation coefficient could not be derived, R was imputed from the trial that had the largest sample size, the lowest risk of bias, and reported change-score data.

Study quality was assessed using the Risk of Bias 2 (RoB 2) tool. Consistent with evidence-based standards for systematic reviews and meta-analyses, this instrument evaluates potential bias across five domains: the randomization process, deviations from intended interventions, missing outcome data, measurement of the outcome, and selection of the reported result. Each domain was rated as low risk, some concerns, or high risk (13).

### 2.5 Statistical analysis

Network meta-analysis was performed using Stata/MP 17.0. For continuous outcomes, MDs with 95% CIs were used when studies reported outcomes on the same scale and in the same units; otherwise, SMDs with 95% CIs were used. For multi-arm trials, pairwise comparisons were generated in augment format, with the within-study correlation structure retained to avoid underestimation of standard errors due to repeated use of the same control group.

The primary analysis was based on a random-effects consistency model, with τ^2^ estimated by REML. Where closed loops were present, global inconsistency tests were used to assess overall consistency and node-splitting analyses were performed to evaluate local inconsistency; *P* < 0.1 was considered to indicate possible inconsistency. Closed-loop consistency was further assessed using the IF; if its 95% CI included 0, no statistical evidence of inconsistency between direct and indirect evidence was found.

Network plots were used to visualize the geometry of the evidence network, with node size proportional to the total sample size for each intervention and edge thickness proportional to the number of studies informing each direct comparison. Treatments were ranked using SUCRA, PrBest, and mean rank to improve the reliability and interpretability of the results.

Publication bias and small-study effects were assessed using comparison-adjusted funnel plots when more than 10 studies were available. Robustness was examined by leave-one-out sensitivity analyses, in which the random-effects consistency model was re-fitted after omitting each study in turn, and the direction and magnitude of the pooled estimates were compared. Univariable network meta-regression was further performed to evaluate the impact of study-level covariates on treatment effects, with regression coefficients, 95% CIs, and Wald test *P* values reported. *P* < 0.05 was considered to indicate statistically significant effect modification by the covariate.

### 2.6 GRADE classification

The certainty of evidence for the network meta-analysis was assessed using CINeMA within the GRADE framework. Evidence from randomized controlled trials was initially considered high certainty. Certainty was then judged across six domains: within-study bias, indirectness, imprecision, heterogeneity, incoherence, and across-study bias (publication bias or small-study effects). Each domain was classified as no concerns, some concerns, or major concerns. In accordance with GRADE, evidence was downgraded by one level for some concerns and by two levels for major concerns, yielding an overall rating of high, moderate, low, or very low certainty.

## 3 Result

### 3.1 Study selection and characteristics

**Figure 1:**
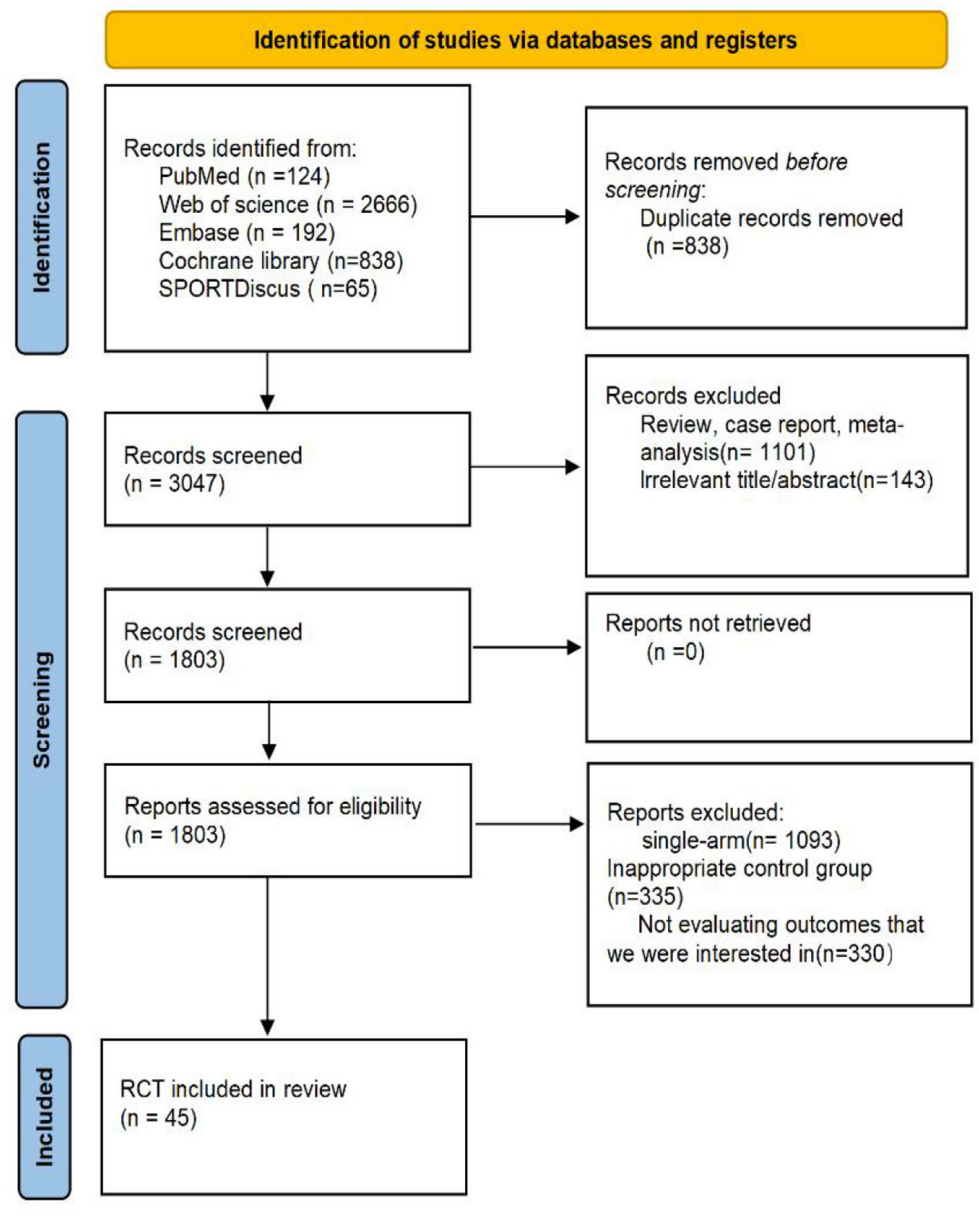
Flow of information the phases of the systematic review and meta-analysis.

The initial search yielded 3,885 records. After removal of duplicates and exclusion of irrelevant articles during title and abstract screening, 1,803 studies were eligible for full-text assessment. Of these, 45 studies met the inclusion criteria and were included in the final analysis (Figure 1) (14–55).

In total, 2,635 patients were enrolled across 17 intervention categories: HIIT, HIIT-AT, MICT, MIIT, LICT, AT, NUT, SOC, HICT, HIIT-MICT, MICT-AT, LICT-AT, MICT-NUT, MIIT-AT, LICT-NUT, HICT-AT, and HIIT-NUT. Intervention duration varied by modality, with HIIT typically lasting 8 weeks, HICT 4–12 weeks, MIIT or MICT 8–12 weeks, aerobic exercise combined with resistance training 12–16 weeks, resistance training alone 12 weeks, and nutrition education or SOC 16 weeks. The studies were conducted across several countries, including China (*n* = 9), the United States (*n* = 5), South Korea (*n* = 6), and others. Sample sizes were moderate overall, and participants were predominantly children and adolescents, with mean ages generally ranging from 9 to 19 years. Sex distribution was relatively balanced. Post-intervention follow-up varied across studies, most commonly from 8 to 24 weeks. Detailed study characteristics are provided in Table 1.

**Table 1:** Characteristics of included trials.

Number	Reference	Age (Experimental/ Control)	Country	Intervention Measures for the Experimental Group	Background antipsychotic	Duration (weeks)	Basic information		Outcome metrics	Numbers (*n*)	
							Gender (Male/ Female)	Patient		Intervention	Control
1	Aaron L Carrel (14)	12.5/12.5	United States	A fitness program oriented toward lifestyle and emphasizing physical exercise, lasting for 9 months	Standard fitness program, duration of 9 months	36 weeks	14/13	Overweight children	vo2max	27	23
2	Andreas A Meyer (15)	13.7/14.7	Germany	A regular exercise regimen lasting 6 months, consisting of 3 sessions per week with each session lasting 1 h.	standard of care	24 weeks	17/17	Obese children	Flow–mediated vasodilation (FMD, assessing endothelial function), carotid intima–media thickness (IMT), systolic blood pressure	33	34
3	Angela S Alberga (16)	15.5/15.9/15.5/15.6	Canada	22 weeks of moderate–intensity aerobic training, 22 weeks of moderate–intensity resistance training, and 22 weeks of combined moderate–intensity aerobic and resistance training	standard of care	24 weeks	3.0/7	Obese adolescents	vo2peak	75/78/75	76
4	Ana Sofia R Alves (17)	14.77	Portuguesa	10–week multi–component training program	standard of care	10 weeks		Obese children	Aerobic capacity	13	14
5	Wissal Abassi (19)	16.1/16.5/16.10	Tunisia	High–intensity interval training: 12–week training program, 3 times per week; Moderate–intensity interval training: 12–week training program, 3 times per week;	standard of care	10 weeks	0/24	Overweight/ obese adolescent girl	Aerobic capacity	8	8
6	María José Aguilar–Cordero (18)	10.43	Spain	Physical activity and Nutritional recommendations	Nutrition–only courses/ recommendations	36 weeks	52/46	Overweight/ obese children	hypertension	49	49
7	Wissal Abassi (19)	16.4	Tunisia	High–intensity interval training (HIIT group): 2 × 6–8 sets of 30–s exercises performed at 100–110% maximal aerobic speed (MAS), with 30–s rest intervals between repetitions. Active recovery periods of 30 s were applied during repetitions performed at 50% MAS. Sample size *n* = 13. Moderate–intensity interval training (MIT group): 2 × 6–8 sets of 30–s exercises performed at an average intensity of 70–80%, with 30–s active recovery intervals between repetitions. Recovery periods were set at 50% of the average intensity. Sample size *n* = 13.	standard of care	12 weeks	0/38	Overweight Girl	Blood pressure, maximum heart rate, systolic blood pressure	13	13
8	A C Benson (20)	12.3/12.2	New Zealand	A 10–week comprehensive intervention (including physical activity, nutrition, and psychological counseling)	standard of care	8 weeks	46/32	Overweight children	Cardiopulmonary function, left ventricular mass	29	34
9	Thaynã Alves Bezerra (21)	7.9	Brazil	12–week intensive exercise program (90 min per day, twice weekly)	standard of care	10 weeks	18/23	Overweight children	VO2max	20	21
10	Hyun–Wook Chae (22)	10.4/10.6	Korea	HIIT group (8 × 2 min, peak output 90%) and super HIIT group (8 × 20 s, peak output 170%), trained 3 times per week for 12 weeks	standard of care	12 weeks	21/17	Obese children	VO2peak, vascular function	19	19
11	Napasakorn Chuensiri (23)	11.0/10.6	Thailand	HIIT	standard of care	12 weeks	48/0	Obese male adolescents	VO2peak, vascular function	10.0/11	11
12	N Cvetković(24)	12.5	Serbia	Entertainment football–related training, high–intensity interval training, and leisure ball–touching activities	standard of care	12 weeks	42/0	Obese male adolescents	Resting heart rate, blood pressure	10.0/11	14
13	Meng Cao (25)	11.2/10.9	China	A 12–week HIIT intervention, administered three times per week	standard of care	12 weeks	20/20	Obese children	VO2max	20	20
14	Katrin A Dias (26)	12.4/11.8	Australia	A 12–week high–intensity interval training (HIIT) program, and a 12–week moderate–intensity continuous training (MICT) program	Nutrition Advice Group Only	12 weeks	46/53	Obese children	Cardiopulmonary Fitness (CRF)	33/29	31
15	Catherine L Davis (27)	9.6/9.7	United States	An 8–month daily post–class aerobic exercise program, 40 min per day	standard of care	32 weeks	68/107	Overweight/ obese children	VO2peak, diastolic blood pressure	90	85
16	Ragab K Elnaggar (28)	12.69/13.36	Arab	8–week incremental aerobic training	standard of care	8 weeks	15.0/12	Obese children with asthma	VO2peak, maximum heart rate, heart rate	13	14
17	Nathalie J Farpour–Lambert (29)	9.1/8.8	Switzerland	Training 3 times per week for 60 min each session, over a period of 3 months	standard of care	12 weeks	16/28	Adolescent obesity in children	SBP	22	22
18	Vandana Jain (30)	11.7/11.4	India	Yoga and Dietary Adjustments	standard of care	18 weeks	112/53	Overweight/ obese children	SBP	35/40	27
19	Jun Kim (31)	15/15	Korea	12–week rope skipping exercise	standard of care	12 weeks	0/48	Obese adolescent female	SBP	24	24
20	Yun Hee Lee (32)	13	Korea	10 weeks of aerobic exercise training, 10 weeks of combined exercise training	standard of care	10 weeks	45.0/9	Obese children	SBP	20/16	18
21	Danielle Lambrick (33)	9.3	United States	Active game intervention twice weekly, 40 min per session	standard of care	6 weeks	32/23	Obese children	VO2max	15	14
22	Cao Meng (34)	11.4/11.0	China	High–intensity interval training group, moderate–intensity continuous training group	standard of care	12 weeks	45/0	Obese boy	Cardiorespiratory fitness (CRF) and cardiac metabolic biomarkers	12.0/11	13
23	D M Prado (35)	10.3/10.2	Brazil	Low–calorie diet and exercise training	Only low–calorie diet	12 weeks		Obese children	VO2peak, heart rate	18	15
24	G Racil (36)	15.6/16.3/15.9	Tunisia	High–intensity interval training, moderate–intensity interval training	standard of care	12 weeks	0/34	Adolescent girls with obesity	VO2max	11	12
25	Ghazi Racil (37)	16.6/16.5/16.9	Tunisia	12–week explosive exercise combined with high–intensity interval training, 12–week high–intensity interval training	standard of care	12 weeks	0/68	Adolescent girls with obesity	vo2peak	23/26	19
26	Hee–Tae Roh (38)	12.60/12.50	Korea	Taekwondo training was conducted five times per week for a duration of 16 weeks.	standard of care	16 weeks	7.0/3	Overweight/ obese adolescents	VO2max	10	10
27	Won–Mok Son (39)	15	Korea	CRAE (Combined Resistance and Aerobic Exercise) Training: Lasted for 12 weeks, conducted 3 times per week	standard of care	12 weeks	0/40	Adolescent girls with obesity	SBP, DBP	20	20
28	Young–Gyun Seo (40)	12.92/12.39	Korea	16–week exercise	standard of care	16 weeks	63/40	Overweight/ obese children	DBP	22	36
29	Marit Salus (41)	13.1/13.7	Estonia	Sprint Interval Training (SIT): 3 times per week, performing 4 sets of 30–s maximal sprints with 4–minute rest intervals between sets, for a total duration of 12 weeks.	standard of care	12 weeks	28/0	Obese children	Cardiorespiratory fitness (CRF)	14	14
30	Fabrício Vasconcellos (42)	14.1/14.8	Brazil	The 12–week recreational football program consists of sessions three times per week, each lasting 60 min.	standard of care	12 weeks	14.0/6	Obese adolescents	VO2peak, blood pressure	10	10
31	Patricia C H Wong (43)	13.8/14.3	Singapore	Additional exercise sessions twice weekly at 12 weeks (combining cyclic resistance training and aerobic exercise with regular physical education classes)	The school offers two 40–minute physical education classes per week.	12 weeks	24/0	Obese adolescent males	Resting heart rate, systolic blood pressure	12	12
32	Hong–Jie Yu (44)	9.9/9.7	China	An 8–month intervention involving nutritional education and physical activity	standard of care	32 weeks	136/35	Obese children	DBP	99	72
33	Ragab K Elnaggar (28)	14.38/14.12/13.92	Saudi Arabia	Constant–load aerobic training group (CL–AE), Progressive aerobic training group (G–AE)	standard of care	12 weeks	50/28	Obese children	vo2reak, VE, ve/vo2, svco2, Hrmax, HRR1	26	26
34	C Y Rodriguez–Triviño (45)	8	Colombia	16–week high–intensity intermittent exercise/16–week moderate–intensity intermittent exercise	standard of care	16 weeks	30/32	Obese children	heart rate	29/33	0
35	Chongwen Zuo (60)	8.1/7.9	China	15 × 20 s at 85–95% maximal aerobic speed (MAS), with 15 × 20 s of recovery in between, followed by 50% MAS, performed 3 days per week; 30 min at 60–70% MAS, performed 3 days per week	standard of care	8 weeks	40/0	Overweight adolescent boys	vo2peak	20	0
36	Yuhang Gao (46)	10.1	China	Short interval running and aerobic exercise, twice weekly for 8 weeks; each training session includes 10 min of sprint interval running and 10 min of aerobic exercise, or short interval running and strength training), twice weekly for 8 weeks; each training session includes 10 min of sprint interval running and 10 min of strength training.	standard of care	8 weeks	15/15	Obese children	vo2max	10	10
37	Xinghao Wang (47)	10.52	China	Moderate–intensity continuous training, high–intensity interval training (HIIT), and HIIT combined with dietary intervention group	standard of care	9 weeks	15/15	Overweight children	heart rate	30/30/30	0
38	Shitong Shao (48)	12.2/12.3/12.6	China	Short–duration interval rope skipping training 3 times per week for 12 weeks, followed by long–duration interval rope skipping training 3 times per week for 12 weeks.	standard of care	12 weeks	60/60	Overweight/ obese adolescents	Cardiorespiratory fitness (CRF)	40/40	40
39	Ting Liao (49)	12.76/13.64	China	A 4–week, 3–times–per–week aquatic high–intensity interval training program, each session lasting 1 h (20 min warm–up and 30 min HIIT + 10 min stretching and relaxation), followed by a 4–week, 3–times–per–week land–based high–intensity interval training program, each session also lasting 1 h (with the same protocol as the aquatic program).	standard of care	4 weeks	15/13	Overweight/ obese adolescents	Resting heart rate, systolic blood pressure, diastolic blood pressure, vital capacity	46343	0
40	Neiva Leite (50)	13.5	Brazil	Aerobic exercise performed 3 times per week for 12 weeks, consisting of “high–intensity interval training (35 min per session, reserve heart rate 80–100%) and moderate–intensity continuous training (60 min per session, reserve heart rate 35–75%)”.	standard of care	12 weeks	107/0	Overweight adolescent boys	SBP, DBP	26	12
41	Zheng–yu Su (51)	15/14	China	The training regimen lasted for 8 weeks and was conducted as follows: 10 sets of "1–minute high–intensity training (peak heart rate 85%−95%) and 2–minute active recovery (peak heart rate 60%−70%)	An 8–week program of 35–minute moderate–intensity continuous training (peak heart rate 65%−75%)	8 weeks	44/0	Overweight adolescent boys	vo2peak	22/22	0
42	Mattia D'Alleva (52)	15.7/16.2	Italy	MICT–HIIT	30 sessions of moderate–intensity continuous training alone	3 weeks	21/0	Overweight adolescent boys	vo2peak	10.0/11	0
43	Noelia González–Gálvez (53)	12.51	Spain	The training regimen consisted of 8 weeks of sessions conducted during the physical education class relaxation phase, with 2 sessions per week and each session lasting 12 min. The training protocol included 6 sets of “60–s high–intensity sprints (heart rate reaching 90–95% of maximum heart rate) and 60–s rest periods (heart rate at 50–55% of maximum heart rate).” Alternatively, the training was performed over 8 weeks during the same relaxation phase, with 2 sessions per week and each session lasting 12 min. The alternative protocol comprised 3 sets of “2–minute high–intensity training (heart rate at 80–85% of maximum heart rate) and 2–minute rest periods (heart rate at 50–55% of maximum heart rate).”	standard of care	8 weeks	18/14	Obese adolescents	SBP, DBP, vo2max	9.0/11	12
44	Emir Tas (54)	15.2/15.4	United States	5–week supervised high–intensity interval training (HIIT)	standard of care	4 weeks	16/21	Obese adolescents	Cardiorespiratory fitness (CRF)	31	6
45	Ghazi Racil (55)	14.34/14.18/14.50	Tunisia	8 weeks of High–Intensity Interval Training (HIIT) or Moderate–Intensity Interval Training (MIIT)	standard of care	8 weeks	0/35	Adolescent girls with obesity	Maximum oxygen uptake, blood pressure,	12.0/11	11

### 3.2 Risk-of-bias assessment

Risk of bias in the 45 included randomized controlled trials was evaluated using the RoB 2 tool. Overall, 5 studies were judged as low risk, 39 as some concerns, and 1 as high risk, suggesting that the overall quality of the included evidence was relatively high.

Across individual domains, 17 studies were rated as low risk for the randomization process, and 12 for deviations from intended interventions. For missing outcome data, 22 studies were classified as low risk. Low risk was also assigned to 26 studies for outcome measurement and to 25 studies for selection of the reported result.

### 3.3 Consistency assessment

Closed loops were identified in the networks for VO_2_peak, VO_2_max, SBP, DBP, MAS, HRmax, and RHR, and global inconsistency tests were therefore performed for these outcomes. All *P* values exceeded 0.05, indicating no significant global inconsistency (Supplementary Table 3). Local inconsistency was further assessed using the node-splitting approach, with all corresponding *P* values also > 0.05 (Supplementary Tables 4–10).

Consistency between direct and indirect evidence was additionally examined by loop inconsistency analysis. Except for MICT–MIIT–NUT–SOC for VO_2_peak (0.25–9.70), HIIT–MIIT–AT–SOC for VO_2_max (0.42–7.54), and LICT–MIIT–SOC for RHR (1.08–11.10), the confidence intervals of all inconsistency factors included 0. These results support good overall consistency of the network (Supplementary Figures 2–8). Therefore, the main analysis was performed using the consistency model.

## 4 Pulmonary function

### 4.1 VO_2_peak

**Figure 2:**
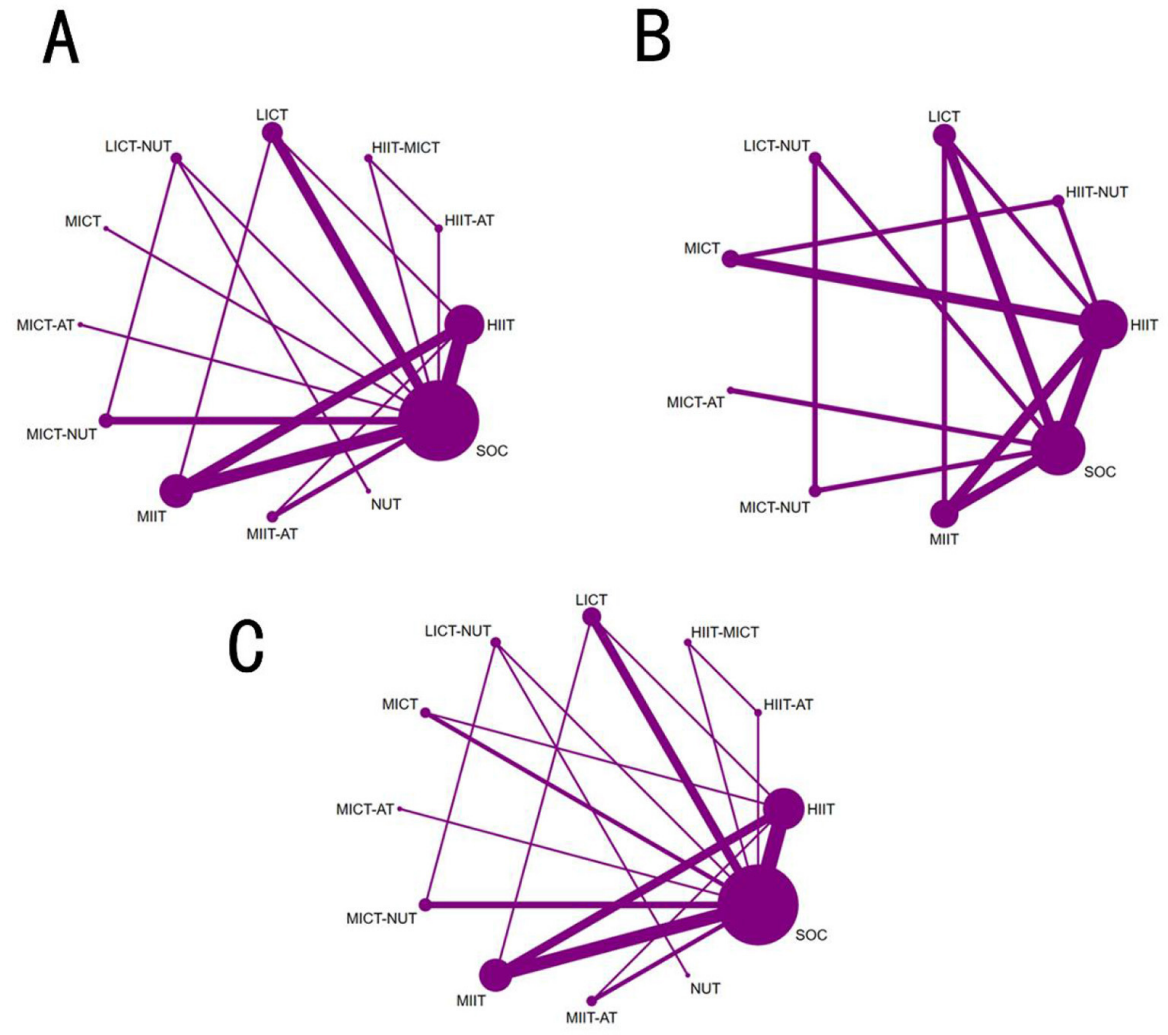
Network maps showing connections between various physical activity and dietary approaches for adolescents with obesity: **(A)** VO_2_peak; **(B)** VO_2_max; **(C)** MAS.

**Figure 3:**
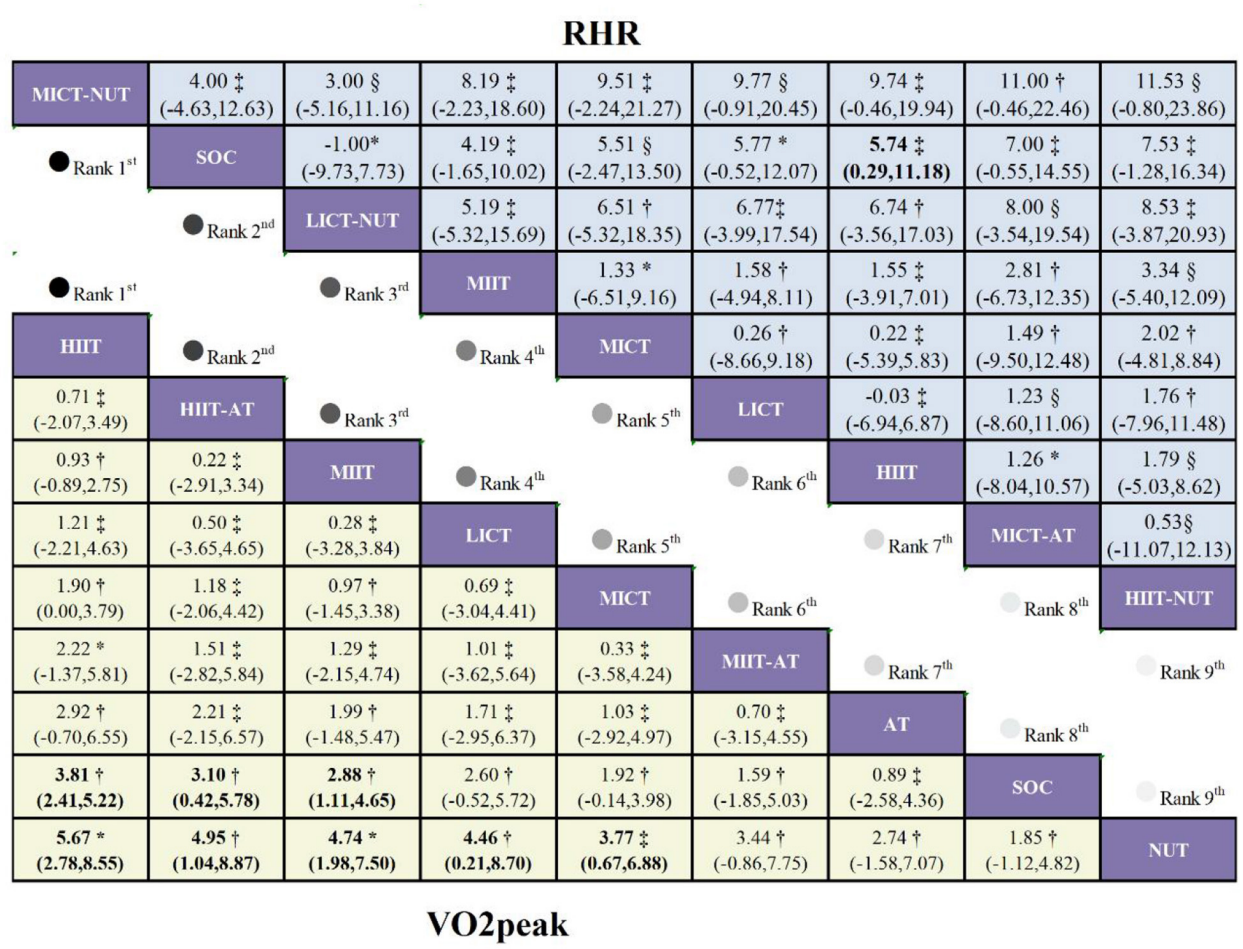
League table comparing exercise interventions in children with obesity. Treatment effects for VO_2_peak are presented in the lower-left triangle (yellow shading), while effects for RHR are shown in the upper-right triangle (blue shading). The certainty of evidence, assessed using the GRADE framework, is denoted as follows: * high certainty, † moderate certainty, ‡ low certainty, and § v ery low certainty.

Eighteen studies involving 1,167 patients and 9 interventions reported VO_2_peak outcomes (Supplementary Table 11; Figure 2A). Moderate-certainty evidence indicated that, relative to SOC, HIIT (MD = 3.81 mL·kg^−1^·min^−1^, 95% CI 2.41 to 5.22; Figure 3), HIIT-AT (MD = 3.10 mL·kg^−1^·min^−1^, 95% CI 0.42 to 5.78), and MIIT (MD = 2.88 mL·kg^−1^·min^−1^, 95% CI 1.11 to 4.65) significantly increased VO_2_peak. LICT also showed a tendency to improve VO_2_peak versus SOC, but the between-group difference was not statistically significant (MD = 2.60 mL·kg^−1^·min^−1^, 95% CI −0.52 to 5.72).

Based on SUCRA, HIIT ranked highest (88.9%), followed by HIIT-AT (72.1%). Probability ranking likewise identified HIIT as the intervention most likely to be best (PrBest = 42.4%), with HIIT-AT ranking second (22.5%). The mean rank yielded a consistent pattern, placing HIIT first (1.9) and HIIT-AT second (3.2).

One important correction: in your Chinese text, the effect estimate for HIIT-AT was mistakenly written as kg/m^−1^, but for VO_2_peak it should clearly be mL·kg^−1^·min^−1^.

### 4.2 VO_2_max

**Figure 4:**
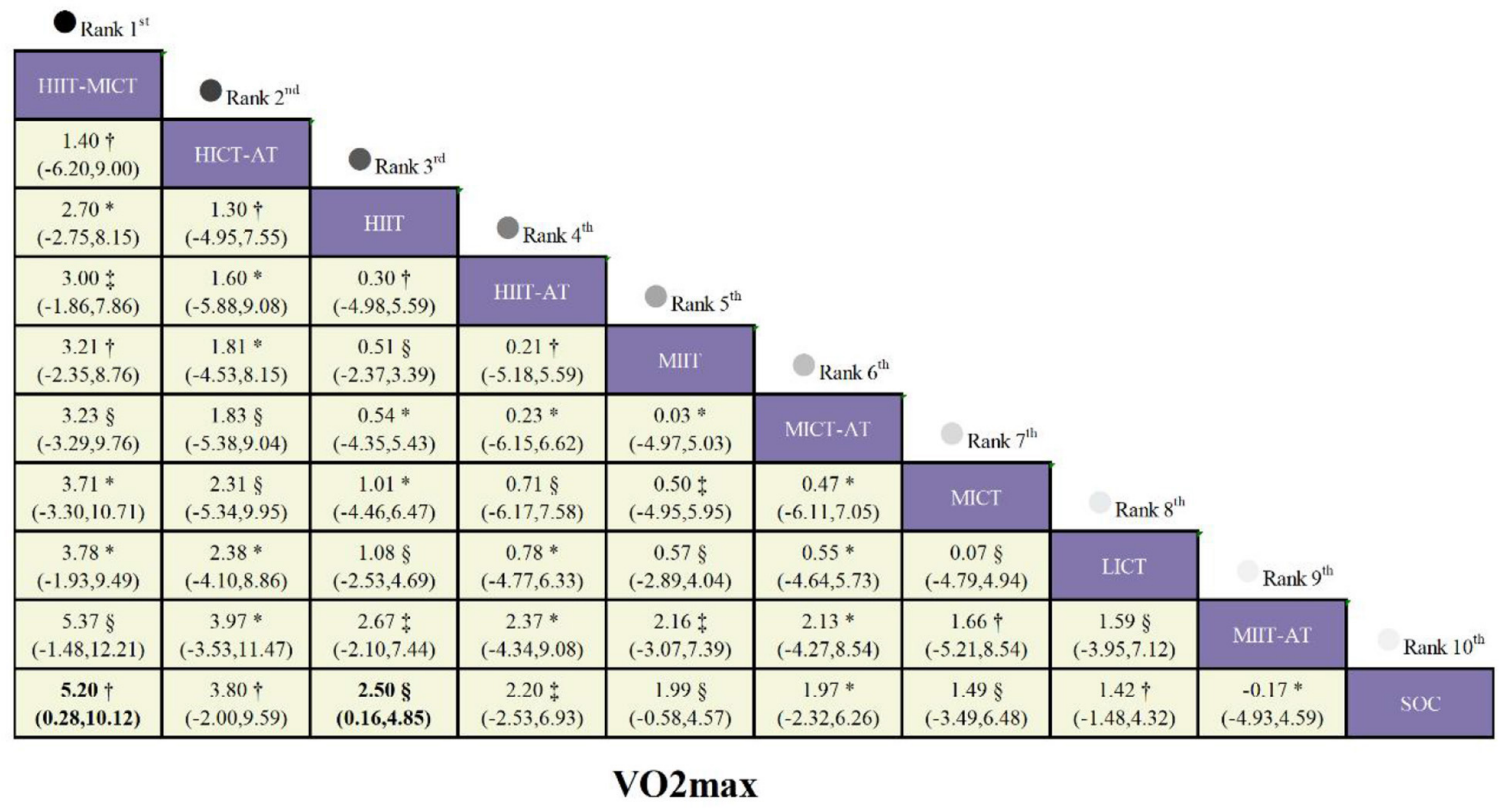
League table comparing exercise interventions in children with obesity. Treatment effects for VO_2_max are presented in the lower-left triangle (yellow shading).

For the outcome of VO_2_max, data were pooled from 11 eligible studies, which evaluated the efficacy of 10 distinct interventions in a total of 484 patients (Supplementary Table 12; Figure 2B). Moderate-certainty evidence showed that the HIIT-MICT regimen resulted in a considerably greater increase in VO_2_max compared with SOC (MD 5.20 mL·kg^−1^·min^−1^, 95% CI 0.28 to 10.12; Figure 4). Low-certainty evidence further demonstrated that standalone high-intensity interval training (HIIT) also produced a statistically significant increase in VO_2_max relative to SOC (MD 2.50 mL·kg^−1^·min^−1^, 95% CI 0.16 to 4.85). Moderate-certainty evidence also indicated that high-intensity continuous training combined with aerobic training (HICT-AT) was associated with a trend toward increased VO_2_max versus SOC (MD 3.80 mL·kg^−1^·min^−1^, 95% CI−2.00 to 9.59), though this between-group difference did not reach statistical significance.

According to the SUCRA values, HIIT-MICT ranked first with a score of 85.9%, followed by HICT-AT at 70.0%, demonstrating the relative overall efficacy advantage of these two interventions. However, probability analysis revealed that HIIT-MICT had the highest likelihood of being the most effective intervention, with a 48.5% probability of ranking first, followed by HICT-AT (28.1% probability of ranking first). Furthermore, mean rank analysis further corroborated the relative superiority of HIIT-MICT and HICT-AT: HIIT-MICT occupied the top position with the lowest mean rank of 2.3, and HICT-AT followed closely with a mean rank of 3.7.

### 4.3 MAS

**Figure 5:**
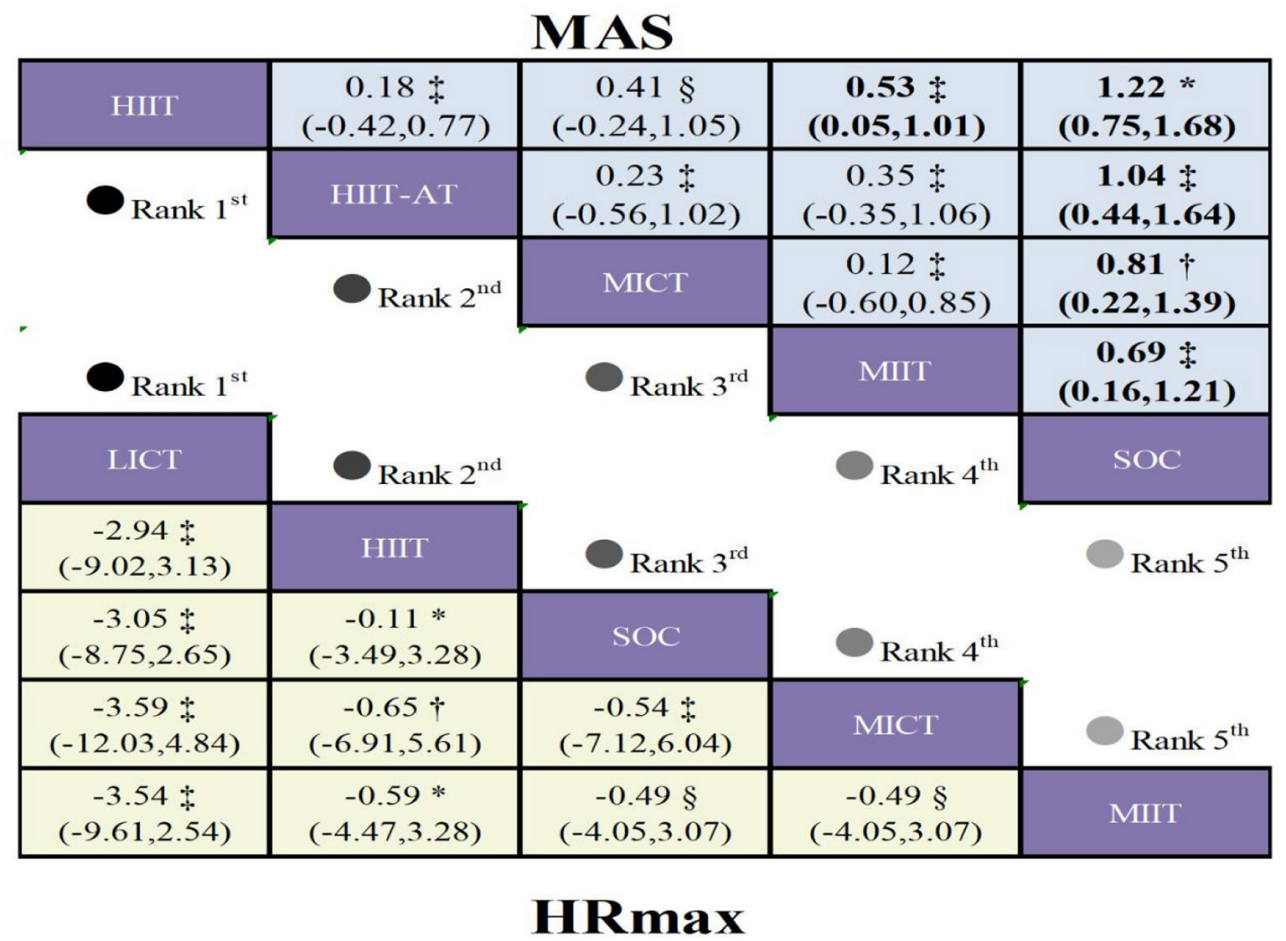
League table comparing exercise interventions in children with obesity. Treatment effects for HR_max_ are presented in the lower-left triangle (yellow shading), while effects for MAS are shown in the upper-right triangle (blue shading). The certainty of evidence, assessed using the GRADE framework, is denoted as follows: * high certainty, † moderate certainty, ‡ low certainty, and § very low certainty.

Pooled analyses for maximal aerobic speed (MAS) were based on 6 studies comprising 239 patients and investigating 5 distinct interventions (Supplementary Table 13; Figure 2C). High-certainty evidence indicated that HIIT significantly improved MAS relative to SOC, with a mean difference (MD) of 1.22 m·s^−1^ (95% CI 0.75 to 1.68). Additionally, low-certainty evidence showed that both HIIT-AT (MD = 1.04 m·s^−1^, 95% CI 0.44 to 1.64; Figure 5) and MIIT (MD = 0.69 m·s^−1^, 95% CI 0.16 to 1.21) also yielded statistically significant improvements in MAS compared with SOC.

Based on SUCRA values, HIIT achieved the highest rank with a score of 90.3%, followed by HIIT-AT at 70.9%, indicating their superior overall efficacy. Probabilistic ranking analysis further revealed that HIIT had the greatest likelihood of being the optimal intervention (66.0% probability of being first), followed by HIIT-AT (25.8%). Consistent with these findings, mean rank analysis confirmed the relative superiority of HIIT and HIIT-AT: HIIT was ranked first with the lowest mean rank of 1.4, and HIIT-AT followed closely at 2.2.

## 5 Cardiac function

### 5.1 HR_*max*_

**Figure 6:**
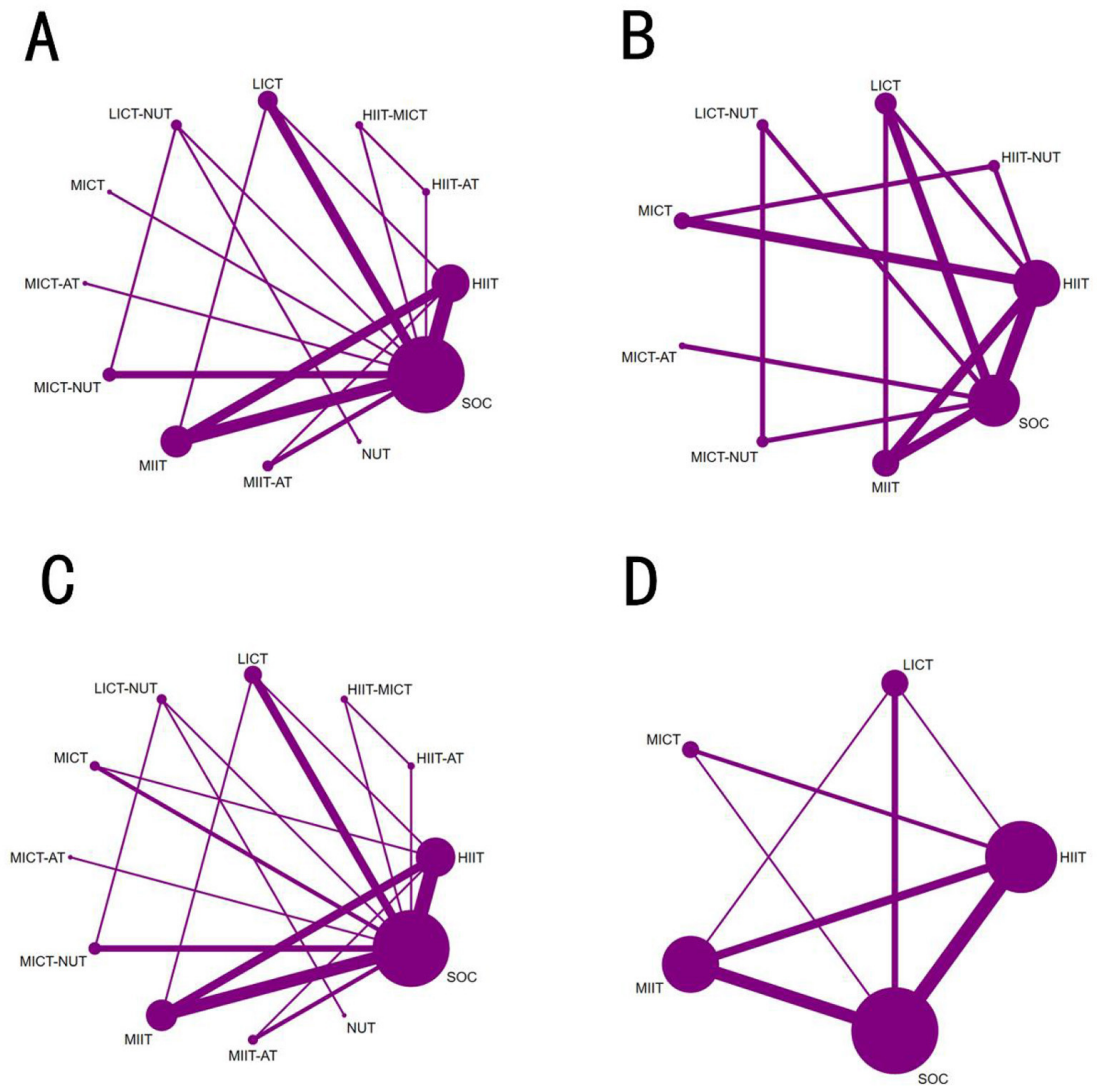
Network maps showing connections between various physical activity and dietary approaches for seniors with muscle-depleting obesity: **(A)** HR_max_; **(B)** SBP; **(C)** DBP; **(D)** RHR.

A total of 13 studies evaluating HR_max_ were included, encompassing 521 patients and five intervention modalities (Supplementary Table 14; Figure 6A). High-certainty evidence indicated that, compared with standard of care (SOC), HIIT may not significantly influence maximal heart rate in the short term. (MD = −0.11 bpm, 95% CI −3.49 to 3.28; Figure 5); and this effect was not statistically significant. LICT also does not significantly affect maximal heart rate over a short period. (MD = −3.05 bpm, 95% CI −8.75 to 2.65).

According to SUCRA rankings, LICT demonstrated the highest probability of being among the most effective interventions (83.0%), followed by HIIT (47.9%), indicating a relative advantage for these approaches in terms of overall efficacy. Consistent with this, probability ranking analysis identified LICT as the most likely optimal intervention (67.1%), with MICT ranking second (15.6%). Mean rank analysis further supported these findings, with LICT achieving the lowest mean rank (1.7), followed by HIIT (3.1).

### 5.2 SBP

**Figure 7:**
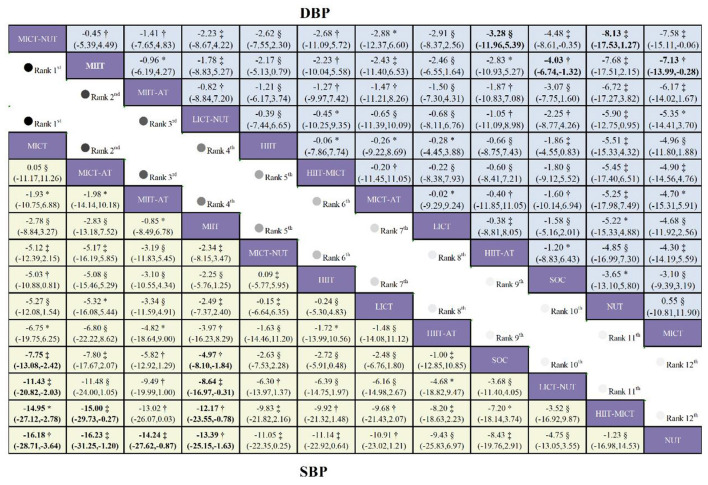
League table comparing exercise interventions in children with obesity. Treatment effects for SBP are presented in the lower-left triangle (yellow shading), while effects for DBP are shown in the upper-right triangle (blue shading). The certainty of evidence, assessed using the GRADE framework, is denoted as follows: * high certainty, † moderate certainty, ‡ low certainty, and § very low certainty.

A total of 23 studies assessing systolic blood pressure (SBP) were included, comprising 1,360 patients and 12 intervention modalities (Supplementary Table 15; Figure 6B). Overall, long-term exercise training was linked to reductions in SBP. Low-certainty evidence indicated that, compared with standard of care (SOC), MICT significantly reduced SBP (MD = −7.75 mmHg, 95% CI −13.08 to −2.42; Figure 7). Moderate-certainty evidence further supported a significant reduction with MIIT relative to SOC (MD = −4.97 mmHg, 95% CI −8.10 to −1.84). In contrast, although MIIT-AT showed a tendency to lower SBP compared with SOC (MD = −5.82 mmHg, 95% CI −12.92 to 1.29), this effect did not reach statistical significance.

According to SUCRA rankings, MICT ranked highest (87.8%), followed by MICT-AT (81.3%), indicating their relative advantage in overall efficacy. However, probability ranking analysis suggested that MICT-AT had the greatest likelihood of being the most effective intervention (38.6%), with MICT ranking second (33.2%). Consistent with these findings, mean rank analysis placed MICT first (mean rank 2.3), followed by MICT-AT (3.1).

### 5.3 DBP

A total of 21 studies reporting diastolic blood pressure (DBP) were included, comprising 1,257 patients and 12 intervention modalities (Supplementary Table 16; Figure 6C). Overall, long-term exercise training was correlated with reductions in DBP. Moderate-certainty evidence indicated that, compared with standard of care (SOC), MIIT significantly reduced DBP (MD = −4.03 mmHg, 95% CI −6.74 to −1.32; Figure 7). In contrast, although high-certainty evidence suggested a potential reduction with HIIT-AT relative to SOC (MD = −1.20 mmHg, 95% CI −8.83 to 6.43), this effect was not statistically significant.

According to SUCRA rankings, MICT-NUT achieved the highest score (80.3%), followed closely by MIIT (78.5%), indicating a relative advantage for these interventions in overall efficacy. Probability ranking analysis, however, identified MICT-NUT as the most likely optimal intervention (24.8%), with MICT-AT ranking second (14.9%). Mean rank analysis yielded a broadly consistent pattern, with MICT-AT ranked first (mean rank 3.2), followed by MIIT (3.4).

### 5.4 RHR

A total of eight studies reporting resting heart rate (RHR) were included, encompassing 525 patients and nine intervention modalities (Supplementary Table 17; Figure 6D). Low-certainty evidence indicated that, compared with standard of care (SOC), HIIT was associated with a statistically significant increase in RHR (MD = 5.74 bpm, 95% CI 0.29 to 11.18; Figure 3). In contrast, although both MICT-AT (MD = 7.00 bpm, 95% CI −0.55 to 14.55) and HIIT-NUT (MD = 7.53 bpm, 95% CI −1.28 to 16.34) showed trends toward increased RHR, neither reached statistical significance.

According to SUCRA rankings, MICT-NUT achieved the highest score (91.2%), indicating a clear advantage in overall efficacy. This was supported by probability ranking analysis, which identified MICT-NUT as the most likely optimal intervention (63.4%). Consistently, mean rank analysis ranked MICT-NUT first, with the lowest mean rank (1.7).

### 5.5 Meta-regression, sensitivity analyses, and publication bias

Baseline profiles were generally well balanced across treatment groups, with no meaningful between-group differences observed. Meta-regression analyses indicated that mean age, intervention duration, and study location (country) were not significantly associated with changes in cardiorespiratory outcomes, supporting the plausibility of the transitivity assumption (Supplementary Tables 25–45).

Sensitivity analyses were conducted using a leave-one-out approach to evaluate the influence of individual studies on the network estimates. The results were robust: exclusion of any single study did not alter the direction of the relative effects between mind–body exercise interventions and NEI. Variations in effect sizes were minimal, 95% confidence intervals largely overlapped, and conclusions regarding statistical significance remained unchanged, indicating good stability of the primary findings (Supplementary Tables 18–21).

Funnel plots were generated for all outcomes to assess small-study effects and the potential for publication bias. Visual inspection showed largely symmetrical distributions, with no evident asymmetry or extreme outliers, suggesting a low likelihood of publication bias (Supplementary Figures 9–15).

### 5.6 Certainty of evidence (GRADE)

The certainty of evidence for network estimates was evaluated using the GRADE framework, implemented through the CINeMA approach (Supplementary Tables 46–52). Across all outcomes, a total of seven network comparisons were assessed. The distribution of certainty ratings was as follows: 52 high-certainty, 64 moderate-certainty, 84 low-certainty, and 69 very low-certainty comparisons.

Higher certainty ratings were typically observed in comparisons supported by multiple direct head-to-head trials, with confidence intervals that neither crossed the line of no effect nor extended beyond the minimal important difference (MID). In contrast, low or very low certainty ratings were more common in sparse networks or in comparisons relying predominantly on indirect evidence, often accompanied by wide confidence intervals and potential small-study effects.

## 6 Discussion

### 6.1 Main findings

Because multiple exercise-based interventions are available for improving cardiorespiratory fitness in adolescents with obesity, yet direct head-to-head evidence remains limited, we used network meta-analysis to integrate direct and indirect comparisons and evaluate the relative effects of HIIT, MIIT, MICT, LICT, AT, SOC, and combined aerobic–resistance training. This systematic review included 45 randomized controlled trials involving 2,635 adolescents with obesity and 17 intervention modalities [1.1].

For cardiorespiratory fitness outcomes, moderate-certainty evidence showed that, relative to standard of care (SOC) or nutritional intervention alone (NUT), both high-intensity interval training (HIIT) and high-intensity circuit training (HICT) significantly improved maximal oxygen uptake (VO_2_max) and peak oxygen uptake (VO_2_peak). In addition, high-certainty evidence indicated that HIIT significantly increased maximal aerobic speed (MAS). Collectively, these findings suggest that high-intensity interval-based aerobic exercise and high-intensity circuit training are the most effective approaches for enhancing cardiorespiratory fitness in adolescents with obesity (34).

Moderate-certainty evidence also indicated that HIIT combined with resistance training (HIIT-AT) improved cardiorespiratory fitness compared with SOC, although its effects appeared somewhat smaller than those observed with HIIT or HICT alone. This finding is consistent with previous studies showing that appropriately prescribed resistance training, when combined with aerobic exercise, may provide additional benefits over aerobic training alone, including improvements in cardiovascular tolerance and upper- and lower-limb strength (56). By contrast, the certainty of evidence for the remaining interventions was generally low or very low.

For cardiovascular outcomes, moderate-certainty evidence indicated that moderate-intensity interval training (MIIT) significantly reduced diastolic blood pressure (DBP), systolic blood pressure (SBP), and resting heart rate (RHR). These results suggest that sustained moderate-intensity interval aerobic exercise may be particularly effective for improving blood pressure control and reducing cardiac workload in adolescents with obesity (57). The certainty of evidence for other interventions in this domain was likewise low or very low.

### 6.2 Interpretation of the findings

HIIT was used as a key comparator because of its established efficacy and widespread use, and it consistently improved cardiorespiratory fitness by enhancing oxygen delivery and utilization, thereby increasing VO_2_max and VO_2_peak.

These effects are likely mediated by multiple adaptations. HIIT may activate AMPK–PGC-1α signaling, promote mitochondrial biogenesis, enhance oxidative enzyme activity and capillarization, and improve oxygen extraction and intramuscular transport, thereby increasing aerobic metabolic efficiency (58).

Several other modalities, including HIIT-AT, HICT, MIIT, and LIIT, also improved cardiorespiratory fitness. Among them, HIIT-AT may be particularly effective, likely because combined aerobic and resistance training produces complementary adaptations in cardiac function, muscle mass, oxygen utilization, body composition, and overall physical performance, thereby yielding greater improvements in VO_2_max and exercise capacity than aerobic training alone (59).

### 6.3 Implications for clinical practice and future research

Our results underscore the pivotal role of exercise training in the management of adolescent obesity. As a feasible first-line intervention, exercise-based strategies provide a practical and evidence-based means of improving health outcomes in this population. By offering a comparatively comprehensive synthesis of the available evidence, this study may help inform more precise and clinically actionable recommendations.

Exercise training is a low-cost, safe, and scalable strategy for adolescents with obesity, but its effects vary across individuals, supporting personalized prescription. HIIT appears particularly effective during this critical developmental period for improving cardiorespiratory fitness and may also help reduce or better control BMI.

Among the evaluated interventions, HIIT-AT may offer broader benefits than single-modality exercise and may be particularly suitable for adolescents. However, because HIIT is not appropriate for all individuals, especially those with lower baseline fitness, MIIT may serve as a more tolerable initial option that supports gradual adaptation.

### 6.4 Comparison with previous studies

The present study differs from earlier reviews in several important respects. First, our analysis was not confined to a single exercise modality. Whereas Tian et al. focused primarily on the overall effects of high-intensity interval training (HIIT) in adolescents with obesity, We separately assessed a range of exercise interventions to enable a more accurate comparison of their relative effectiveness. In addition, we incorporated resistance training into the intervention framework, thereby extending the analysis beyond aerobic exercise alone. Our findings indicate that combined training may confer greater benefits than aerobic training performed in isolation.

This review also extends previous work through a broader assessment of outcomes. Compared with the study by João Victor Affornali Tozo et al., we included a wider range of clinically relevant endpoints, including VO_2_peak, HRmax, MAS, and RHR. Notably, our analysis showed that combined aerobic and resistance training, compared with standard of care, resulted in statistically significant improvements in HRmax and VO_2_max. João Victor Affornali Tozo et al. likewise reported favorable effects of combined training on SBP and DBP. Taken together, these findings suggest that combined exercise modalities may provide more comprehensive benefits across both cardiorespiratory and cardiovascular domains in adolescents with obesity.

### 6.5 Strengths and limitations

This study was conducted with a rigorous methodological approach. Risk of bias in the included studies was assessed using the ROB 2 tool, and the certainty of the evidence was evaluated within the CINeMA framework. Sensitivity analyses and network meta-regression were further undertaken to test the robustness of the main findings. The primary outcomes were also interpreted against established MCID thresholds within a predefined grading framework, which strengthened their clinical interpretability and the certainty of the resulting inferences. Importantly, interventions were classified in as much detail as the available data allowed, enabling specific intervention types to be evaluated separately rather than combined across conceptually heterogeneous approaches. This approach improved the precision of the comparative analyses and increased the clinical significance of the results. By focusing specifically on children and adolescents aged 5–19 years with obesity, the study addressed a relatively homogeneous target population and helped to fill an important gap in the comparative evidence base for multiple intervention strategies in this area.

Several limitations should also be acknowledged. Blinding of participants and intervention personnel is often impractical in exercise-based trials. As a result, some included studies were judged to raise some concerns or to be at high risk of bias, which may have reduced the reliability of the effect estimates, particularly for comparisons supported by only a small number of studies. In addition, the certainty of evidence for some outcomes was rated as low or very low, which limits the generalizability of the conclusions. There was also considerable variation across studies in intervention characteristics, including exercise frequency, session duration, intensity, and overall intervention period. Most studies had relatively short follow-up durations, leaving long-term effects insufficiently understood. Secondary outcomes such as safety, adherence, and quality of life were not assessed. Finally, as this was a study-level meta-analysis, subgroup analyses at the individual-participant level were not possible.

## 7 Conclusion

This network meta-analysis suggests that, in children and adolescents with obesity, high-intensity exercise modalities may offer the greatest benefit for improving cardiorespiratory fitness, while moderate-intensity exercise may be more effective for reducing blood pressure. HIIT-AT also showed promising effects, but its long-term efficacy and safety require further confirmation. Overall, exercise prescription in pediatric obesity should be individualized according to the primary therapeutic goal. Further well-designed head-to-head trials with longer follow-up are needed.

## Data Availability

The original contributions presented in the study are included in the article/Supplementary material, further inquiries can be directed to the corresponding author.
