# Supplementary material for: Exercise training modalities for cardiorespiratory fitness and blood pressure in children and adolescents with obesity: a systematic review and network meta-analysis
Source: Front Public Health. 2026 May 20;14:1842123. doi: 10.3389/fpubh.2026.1842123 (PMC13229893; doi:10.3389/fpubh.2026.1842123)

**Table S1** PRISMA NMA Checklist of Items to Include When Reporting a Systematic Review Involving a Network Meta-analysis

| Section/Topic | Item # | Checklist Item |  |
| --- | --- | --- | --- |
| TITLE |  |  |  |
| Title | 1 | Identify the report as a systematic review *incorporating*  anetwork meta-analysis (or related form of meta-analysis). |  |
|  |  |  |  |
| ABSTRACT |  |  |  |
| Structured summary | 2 | Provide a structured summary including, as applicable:  **Background:** main objectives  **Methods:** data sources; study eligibility criteria, participants, and interventions; study appraisal; and *synthesis methods, such as network meta-analysis.*  **Results:** number of studies and participants identified; summary estimates with corresponding confidence/credible intervals; treatment rankings may also be discussed. Authors may choose to summarize pairwise comparisons against a chosen treatment included in their analyses for brevity.  **Discussion/Conclusions:** limitations; conclusions and implications of findings.  **Other:** systematic review registration number with registry name. |  |
|  |  |  |  |
| INTRODUCTION |  |  |  |
| Rationale | 3 | Describe the rationale for the review in the context of what is already known*, including mention of why a network meta-analysis has been conducted.* |  |
| Objectives | 4 | Provide an explicit statement of questions being addressed, with reference to participants, interventions, comparisons, outcomes, and study design (PICOS). |  |
|  |  |  |  |
| METHODS |  |  |  |
| Protocol and registration | 5 | Indicate whether a review protocol exists and if and where it can be accessed (e.g., Web address); and, if available, provide registration information, including registration number. |  |
| Eligibility criteria | 6 | Specify study characteristics (e.g., PICOS, length of follow-up) and report characteristics (e.g., years considered, language, publication status) used as criteria for eligibility, giving rationale. *Clearly describe eligible treatments included in the treatment network, and note whether any have been clustered or merged into the same node (with justification).* |  |
| Information sources | 7 | Describe all information sources (e.g., databases with dates of coverage, contact with study authors to identify additional studies) in the search and date last searched. |  |
| Search | 8 | Present full electronic search strategy for at least one database, including any limits used, such that it could be repeated. |  |
| Study selection | 9 | State the process for selecting studies (i.e., screening, eligibility, included in systematic review, and, if applicable, included in the meta-analysis). |  |
| Data collection process | 10 | Describe method of data extraction from reports (e.g., piloted forms, independently, in duplicate) and any processes for obtaining and confirming data from investigators. |  |
| Data items | 11 | List and define all variables for which data were sought (e.g., PICOS, funding sources) and any assumptions and simplifications made. |  |
| Geometry of the network | S1 | Describe methods used to explore the geometry of the treatment network under study and potential biases related to it. This should include how the evidence base has been graphically summarized for presentation, and what characteristics were compiled and used to describe the evidence base to readers. |  |
| Risk of bias within individual studies | 12 | Describe methods used for assessing risk of bias of individual studies (including specification of whether this was done at the study or outcome level), and how this information is to be used in any data synthesis. |  |
| Summary measures | 13 | State the principal summary measures (e.g., risk ratio, difference in means). Also describe the use of additional summary measures assessed, such as treatment rankings and surface under the cumulative ranking curve (SUCRA) values, as well as modified approaches used to present summary findings from meta-analyses. |  |
| Planned methods of analysis | 14 | Describe the methods of handling data and combining results of studies for each network meta-analysis. This should include, but not be limited to:   - Handling of multi-arm trials; - Selection of variance structure; - Selection of prior distributions in Bayesian analyses; and - Assessment of model fit. |  |
| Assessment of Inconsistency | S2 | Describe the statistical methods used to evaluate the agreement of direct and indirect evidence in the treatment network(s) studied. Describe efforts taken to address its presence when found. |  |
| Risk of bias across studies | 15 | Specify any assessment of risk of bias that may affect the cumulative evidence (e.g., publication bias, selective reporting within studies). |  |
| Additional analyses | 16 | Describe methods of additional analyses if done, indicating which were pre-specified. This may include, but not be limited to, the following:   - Sensitivity or subgroup analyses; - Meta-regression analyses; - Alternative formulations of the treatment network; and - Use of alternative prior distributions for Bayesian analyses (if applicable). |  |
| RESULTS† |  |  |  |
| Study selection | 17 | Give numbers of studies screened, assessed for eligibility, and included in the review, with reasons for exclusions at each stage, ideally with a flow diagram. |  |
| Presentation of network structure | S3 | Provide a network graph of the included studies to enable visualization of the geometry of the treatment network. |  |
| Summary of network geometry | S4 | Provide a brief overview of characteristics of the treatment network. This may include commentary on the abundance of trials and randomized patients for the different interventions and pairwise comparisons in the network, gaps of evidence in the treatment network, and potential biases reflected by the network structure. |  |
| Study characteristics | 18 | For each study, present characteristics for which data were extracted (e.g., study size, PICOS, follow-up period) and provide the citations. |  |
| Risk of bias within studies | 19 | Present data on risk of bias of each study and, if available, any outcome level assessment. |  |
| Results of individual studies | 20 | For all outcomes considered (benefits or harms), present, for each study: 1) simple summary data for each intervention group, and 2) effect estimates and confidence intervals. *Modified approaches may be needed to deal with information from larger networks.* |  |
| Synthesis of results | 21 | Present results of each meta-analysis done, including confidence/credible intervals. In larger networks, authors may focus on comparisons versus a particular comparator (e.g. placebo or standard care), with full findings presented in an appendix. League tables and forest plots may be considered to summarize pairwise comparisons. If additional summary measures were explored (such as treatment rankings), these should also be presented. |  |
| Exploration for inconsistency | S5 | Describe results from investigations of inconsistency. This may include such information as measures of model fit to compare consistency and inconsistency models, *P* values from statistical tests, or summary of inconsistency estimates from different parts of the treatment network. |  |
| Risk of bias across studies | 22 | Present results of any assessment of risk of bias across studies for the evidence base being studied. |  |
| Results of additional analyses | 23 | Give results of additional analyses, if done (e.g., sensitivity or subgroup analyses, meta-regression analyses*, alternative network geometries studied, alternative choice of prior distributions for Bayesian analyses,* and so forth). |  |
|  |  |  |  |
| DISCUSSION |  |  |  |
| Summary of evidence | 24 | Summarize the main findings, including the strength of evidence for each main outcome; consider their relevance to key groups (e.g., healthcare providers, users, and policy-makers). |  |
| Limitations | 25 | Discuss limitations at study and outcome level (e.g., risk of bias), and at review level (e.g., incomplete retrieval of identified research, reporting bias). *Comment on the validity of the assumptions, such as transitivity and consistency. Comment on any concerns regarding network geometry (e.g., avoidance of certain comparisons).* |  |
| Conclusions | 26 | Provide a general interpretation of the results in the context of other evidence, and implications for future research. |  |
|  |  |  |  |
| FUNDING |  |  |  |
| Funding | 27 | Describe sources of funding for the systematic review and other support (e.g., supply of data); role of funders for the systematic review. This should also include information regarding whether funding has been received from manufacturers of treatments in the network and/or whether some of the authors are content experts with professional conflicts of interest that could affect use of treatments in the network. |  |

PICOS = population, intervention, comparators, outcomes, study design.

* Text in italics indicate S wording specific to reporting of network meta-analyses that has been added to guidance from the PRISMA statement.

† Authors may wish to plan for use of appendices to present all relevant information in full detail for items in this section.

| **Table S2.Literature Search Strategy** | |
| --- | --- |
| **Pubmed** | #1((("Obesity"[Mesh]) OR ("Overweight"[Mesh])) OR (Obesity [Title/Abstract])) OR (Overweight [Title/Abstract]) "Obesity"[MeSH Terms] OR "Overweight"[MeSH Terms] OR "Obesity"[Title/Abstract] OR "Overweight"[Title/Abstract] 500,431 1:23:15 2025/12/26  #2 "Child"[Mesh] Most Recent “Child"[MeSH Terms] 2,304,401 1:29:38 2025/12/26  #3 ("Child"[Mesh]) OR (Children [Title/Abstract]) "Child"[MeSH Terms] OR "Children"[Title/Abstract] 2,718,472 1:32:23 2025/12/26  #4 #17 and #19 ("Obesity"[MeSH Terms] OR "Overweight"[MeSH Terms] OR "Obesity"[Title/Abstract] OR "Overweight"[Title/Abstract]) AND ("Child"[MeSH Terms] OR "Children"[Title/Abstract]) 74,635 1:34:30 2025/12/26  #5 ((((((("Blood Pressure"[Mesh]) OR (vo2max[Title/Abstract])) OR (vo2peak[Title/Abstract])) OR (Cardiopulmonary Fitness[Title/Abstract])) OR (SBP[Title/Abstract])) OR (DBP[Title/Abstract])) OR (Heart Rate[Title/Abstract])) OR (HR[Title/Abstract]) "Blood Pressure"[MeSH Terms] OR "vo2max"[Title/Abstract] OR "vo2peak"[Title/Abstract] OR "cardiopulmonary fitness"[Title/Abstract] OR "SBP"[Title/Abstract] OR "DBP"[Title/Abstract] OR "heart rate"[Title/Abstract] OR "HR"[Title/Abstract] 838,597 2:06:27 2025/12/26  #6 "Exercise"[Mesh] Most Recent “Exercise"[MeSH Terms] 281,013 2:21:41 2025/12/26  #7 ((("Exercise"[Mesh]) OR (Physical Activity [Title/Abstract])) OR (HIIT[Title/Abstract]) OR (MICT[Title/Abstract]) "Exercise"[MeSH Terms] OR "physical activity"[Title/Abstract] OR "HIIT"[Title/Abstract] OR "MICT"[Title/Abstract] 388,775 2:23:22 2025/12/26  #8 ((#17 and #19) AND (((("Exercise"[Mesh]) OR (Physical Activity[Title/Abstract])) OR (HIIT[Title/Abstract])) OR (MICT[Title/Abstract]))) AND (((((((("Blood Pressure"[Mesh]) OR (vo2max[Title/Abstract])) OR (vo2peak[Title/Abstract])) OR (Cardiopulmonary Fitness[Title/Abstract])) OR (SBP[Title/Abstract])) OR (DBP[Title/Abstract])) OR (Heart Rate[Title/Abstract])) OR (HR[Title/Abstract])) ("Obesity"[MeSH Terms] OR "Overweight"[MeSH Terms] OR "Obesity"[Title/Abstract] OR "Overweight"[Title/Abstract]) AND ("Child"[MeSH Terms] OR "Children"[Title/Abstract]) AND ("Exercise"[MeSH Terms] OR "physical activity"[Title/Abstract] OR "HIIT"[Title/Abstract] OR "MICT"[Title/Abstract]) AND ("Blood Pressure"[MeSH Terms] OR "vo2max"[Title/Abstract] OR "vo2peak"[Title/Abstract] OR "cardiopulmonary fitness"[Title/Abstract] OR "SBP"[Title/Abstract] OR "DBP"[Title/Abstract] OR "heart rate"[Title/Abstract] OR "HR"[Title/Abstract]) 875 2:25:19 2025/12/26  #9 ((#17 and #19) AND (((("Exercise"[Mesh]) OR (Physical Activity[Title/Abstract])) OR (HIIT[Title/Abstract])) OR (MICT[Title/Abstract]))) AND (((((((("Blood Pressure"[Mesh]) OR (vo2max[Title/Abstract])) OR (vo2peak[Title/Abstract])) OR (Cardiopulmonary Fitness[Title/Abstract])) OR (SBP[Title/Abstract])) OR (DBP[Title/Abstract])) OR (Heart Rate[Title/Abstract])) OR (HR[Title/Abstract])) Randomized Controlled Trial (("Obesity"[MeSH Terms] OR "Overweight"[MeSH Terms] OR "Obesity"[Title/Abstract] OR "Overweight"[Title/Abstract]) AND ("Child"[MeSH Terms] OR "Children"[Title/Abstract]) AND ("Exercise"[MeSH Terms] OR "physical activity"[Title/Abstract] OR "HIIT"[Title/Abstract] OR "MICT"[Title/Abstract]) AND ("Blood Pressure"[MeSH Terms] OR "vo2max"[Title/Abstract] OR "vo2peak"[Title/Abstract] OR "cardiopulmonary fitness"[Title/Abstract] OR "SBP"[Title/Abstract] OR "DBP"[Title/Abstract] OR "heart rate"[Title/Abstract] OR "HR"[Title/Abstract])) AND (randomizedcontrolledtrial[Filter]) 124 2:29:55 2025/12/26  #10 (((#17 and #19) AND (((#17 and #19) AND (((("Exercise"[Mesh]) OR (Physical Activity[Title/Abstract])) OR (HIIT[Title/Abstract])) OR (MICT[Title/Abstract]))) AND (((((((("Blood Pressure"[Mesh]) OR (vo2max[Title/Abstract])) OR (vo2peak[Title/Abstract])) OR (Cardiopulmonary Fitness[Title/Abstract])) OR (SBP[Title/Abstract])) OR (DBP[Title/Abstract])) OR (Heart Rate[Title/Abstract])) OR (HR[Title/Abstract])))) AND (((((((("Blood Pressure"[Mesh]) OR (vo2max[Title/Abstract])) OR (vo2peak[Title/Abstract])) OR (Cardiopulmonary Fitness[Title/Abstract])) OR (SBP[Title/Abstract])) OR (DBP[Title/Abstract])) OR (Heart Rate[Title/Abstract])) OR (HR[Title/Abstract]))) AND (((#17 and #19) AND (((("Exercise"[Mesh]) OR (Physical Activity[Title/Abstract])) OR (HIIT[Title/Abstract])) OR (MICT[Title/Abstract]))) AND (((((((("Blood Pressure"[Mesh]) OR (vo2max[Title/Abstract])) OR (vo2peak[Title/Abstract])) OR (Cardiopulmonary Fitness[Title/Abstract])) OR (SBP[Title/Abstract])) OR (DBP[Title/Abstract])) OR (Heart Rate[Title/Abstract])) OR (HR[Title/Abstract])) AND (randomizedcontrolledtrial[Filter])) Randomized Controlled Trial (("Obesity"[MeSH Terms] OR "Overweight"[MeSH Terms] OR "Obesity"[Title/Abstract] OR "Overweight"[Title/Abstract]) AND ("Child"[MeSH Terms] OR "Children"[Title/Abstract]) AND (("Obesity"[MeSH Terms] OR "Overweight"[MeSH Terms] OR "Obesity"[Title/Abstract] OR "Overweight"[Title/Abstract]) AND ("Child"[MeSH Terms] OR "Children"[Title/Abstract]) AND ("Exercise"[MeSH Terms] OR "physical activity"[Title/Abstract] OR "HIIT"[Title/Abstract] OR "MICT"[Title/Abstract]) AND ("Blood Pressure"[MeSH Terms] OR "vo2max"[Title/Abstract] OR "vo2peak"[Title/Abstract] OR "cardiopulmonary fitness"[Title/Abstract] OR "SBP"[Title/Abstract] OR "DBP"[Title/Abstract] OR "heart rate"[Title/Abstract] OR "HR"[Title/Abstract])) AND ("Blood Pressure"[MeSH Terms] OR "vo2max"[Title/Abstract] OR "vo2peak"[Title/Abstract] OR "cardiopulmonary fitness"[Title/Abstract] OR "SBP"[Title/Abstract] OR "DBP"[Title/Abstract] OR "heart rate"[Title/Abstract] OR "HR"[Title/Abstract]) AND (("Obesity"[MeSH Terms] OR "Overweight"[MeSH Terms] OR "Obesity"[Title/Abstract] OR "Overweight"[Title/Abstract]) AND ("Child"[MeSH Terms] OR "Children"[Title/Abstract]) AND ("Exercise"[MeSH Terms] OR "physical activity"[Title/Abstract] OR "HIIT"[Title/Abstract] OR "MICT"[Title/Abstract]) AND ("Blood Pressure"[MeSH Terms] OR "vo2max"[Title/Abstract] OR "vo2peak"[Title/Abstract] OR "cardiopulmonary fitness"[Title/Abstract] OR "SBP"[Title/Abstract] OR "DBP"[Title/Abstract] OR "heart rate"[Title/Abstract] OR "HR"[Title/Abstract]) AND "randomized controlled trial"[Publication Type])) AND (randomizedcontrolledtrial[Filter]) 124 2:34:00 2025/12/26 |
| **Web of Science** | 1: "  - WOS: 1985 to 2025  - BIOSIS: 2002 to 2025  - CSCD: 1989 to 2025  - GRANTS: 1953 to 2025  - INSPEC: 1898 to 2025  - KJD: 1980 to 2025  - MEDLINE: 1950 to 2025  - PPRN: 1991 to 2025  - PQDT: 1637 to 2025  - RC: 2015 to 2025  - SCIELO: 2002 to 2025" 1 "TS=((obes* OR overweight* OR adiposity OR ""excess weight"") AND (child* OR adolescen* OR pediatric OR paediatric OR youth OR teen* OR infant*)) Editions: WOS.IC,WOS.CCR,WOS.SCI,WOS.AHCI,WOS.ESCI,WOS.SSCI,BIOSIS.PREVIEWS,CSCD.CSCD,GRANTS.GRANTS,INSPEC.INSPEC,KJD.KJD,MEDLINE.MEDLINE,PQDT.PQDT,SCIELO.SCIELO " All Databases 264230 Sat Dec 27 2025 15:13:33 GMT+0800 (中国标准时间)  2: "  - WOS: 1985 to 2025  - BIOSIS: 2002 to 2025  - CSCD: 1989 to 2025  - GRANTS: 1953 to 2025  - INSPEC: 1898 to 2025  - KJD: 1980 to 2025  - MEDLINE: 1950 to 2025  - PPRN: 1991 to 2025  - PQDT: 1637 to 2025  - RC: 2015 to 2025  - SCIELO: 2002 to 2025" 2 "TS=(exercis* OR ""physical activit*"" OR ""physical train*"" OR ""exercise training"" OR (train* NEAR/3 (program* OR session* OR intervention* OR therap* OR regimen* OR plan* OR protocol*)) OR ""resistance train*"" OR ""strength train*"" OR ""aerobic train*"" OR ""endurance train*"" OR ""interval train*"" OR ""high intensity interval train*"" OR HIIT OR (circuit NEAR/3 train*) OR plyometric* OR sport* OR ""exercise therapy"" OR ""exercise program*"" OR physiotherap* OR rehab* OR ""physical rehabilitation"" OR ""cardiorespiratory fitness"" OR ""motor skill train*"" OR ""skill train*"" OR ""balance train*"" OR ""flexibility train*"" OR ""strength training"" OR ""weight training"" OR ""power training"" OR ""muscle strength"") Editions: WOS.IC,WOS.CCR,WOS.SCI,WOS.AHCI,WOS.ESCI,WOS.SSCI,BIOSIS.PREVIEWS,CSCD.CSCD,GRANTS.GRANTS,INSPEC.INSPEC,KJD.KJD,MEDLINE.MEDLINE,PQDT.PQDT,SCIELO.SCIELO " All Databases 2711491 Sat Dec 27 2025 15:18:56 GMT+0800 (中国标准时间)  3: "  - WOS: 1985 to 2025  - BIOSIS: 2002 to 2025  - CSCD: 1989 to 2025  - GRANTS: 1953 to 2025  - INSPEC: 1898 to 2025  - KJD: 1980 to 2025  - MEDLINE: 1950 to 2025  - PPRN: 1991 to 2025  - PQDT: 1637 to 2025  - RC: 2015 to 2025  - SCIELO: 2002 to 2025" 3 "TS=((VO2max OR ""VO2 max"" OR VO2peak OR ""VO2 peak"" OR ""peak VO2"" OR ""maximal oxygen uptake"" OR ""maximum oxygen consumption"" OR ""peak oxygen uptake"" OR ""peak oxygen consumption"" OR ""maximal aerobic capacity"" OR ""aerobic capacity"" OR ""cardiorespiratory fitness"" OR ""cardiorespiratory capacity"" OR ""cardiopulmonary fitness"" OR ""cardiopulmonary function"" OR CPET OR ""cardiopulmonary exercise testing"") OR (""heart rate"" OR ""resting heart rate"" OR ""maximum heart rate"" OR ""peak heart rate"" OR ""heart-rate variability"" OR HRV OR HR) OR (""blood pressure"" OR ""systolic blood pressure"" OR SBP OR ""diastolic blood pressure"" OR DBP)) Editions: WOS.IC,WOS.CCR,WOS.SCI,WOS.AHCI,WOS.ESCI,WOS.SSCI,BIOSIS.PREVIEWS,CSCD.CSCD,GRANTS.GRANTS,INSPEC.INSPEC,KJD.KJD,MEDLINE.MEDLINE,PQDT.PQDT,SCIELO.SCIELO " All Databases 1727620 Sat Dec 27 2025 15:25:18 GMT+0800 (中国标准时间)  4: "  - WOS: 1985 to 2025  - BIOSIS: 2002 to 2025  - CSCD: 1989 to 2025  - GRANTS: 1953 to 2025  - INSPEC: 1898 to 2025  - KJD: 1980 to 2025  - MEDLINE: 1950 to 2025  - PPRN: 1991 to 2025  - PQDT: 1637 to 2025  - RC: 2015 to 2025  - SCIELO: 2002 to 2025" 4 "TS=(  random* OR randomi* OR randomly  OR placebo* OR sham  OR trial OR ""clinical trial"" OR ""controlled clinical trial""  OR ""double blind"" OR ""single blind"" OR ""triple blind""  OR crossover OR ""cross over"" OR ""parallel group""  OR cluster random*  OR RCT  ) Editions: WOS.IC,WOS.CCR,WOS.SCI,WOS.AHCI,WOS.ESCI,WOS.SSCI,BIOSIS.PREVIEWS,CSCD.CSCD,GRANTS.GRANTS,INSPEC.INSPEC,KJD.KJD,MEDLINE.MEDLINE,PQDT.PQDT,SCIELO.SCIELO " All Databases 6633262 Sat Dec 27 2025 15:26:37 GMT+0800 (中国标准时间)  5 "  - WOS: 1985 to 2025  - BIOSIS: 2002 to 2025  - CSCD: 1989 to 2025  - GRANTS: 1953 to 2025  - INSPEC: 1898 to 2025  - KJD: 1980 to 2025  - MEDLINE: 1950 to 2025  - PPRN: 1991 to 2025  - PQDT: 1637 to 2025  - RC: 2015 to 2025  - SCIELO: 2002 to 2025" 5 "#1 AND #2 AND #3 AND #4 Editions: WOS.IC,WOS.CCR,WOS.SCI,WOS.AHCI,WOS.ESCI,WOS.SSCI,BIOSIS.PREVIEWS,CSCD.CSCD,GRANTS.GRANTS,INSPEC.INSPEC,KJD.KJD,MEDLINE.MEDLINE,PQDT.PQDT,SCIELO.SCIELO " All Databases 2666 Sat Dec 27 2025 15:41:46 GMT+0800 (中国标准时间) |
| **Cochrane** | #1 MeSH descriptor: [Obesity] explode all trees 22614  #2 Overweight 25413  #3 #1 OR #2 40316  #4 MeSH descriptor: [Child] explode all trees 84007  #5 Children OR Adolescent 334169  #6 #4 OR #5 334169  #7 MeSH descriptor: [Exercise] explode all trees 41560  #8 Training OR Physical Activity 211589  #9 #7 OR #8 226488  #10 MeSH descriptor: [Cardiorespiratory Fitness] explode all trees 768  #11 VO2max OR VO2peak OR Heart Rate OR Blood Pressure OR SBP OR DBP 214063  #12 #10 OR #11 214414  #13 #3 AND #6 AND #9 AND #12 838 |
| **Embase** | #1 ('obesity'/exp OR 'obesity' OR obesity:ti,ab OR obese:ti,ab OR overweight:ti,ab OR 'over weight':ti,ab OR 'high bmi':ti,ab) AND ('adolescent'/exp OR 'adolescent' OR adolescent:ti,ab OR adolescents:ti,ab OR teenager*:ti,ab OR youth:ti,ab OR 'young people':ti,ab) AND ('exercise'/exp OR 'exercise' OR 'exercise therapy'/exp OR 'exercise therapy' OR 'physical activity'/exp OR 'physical activity' OR exercise*:ti,ab OR 'physical activit*':ti,ab OR 'physical exercise*':ti,ab OR aerobic:ti,ab OR 'aerobic training':ti,ab OR 'endurance training':ti,ab OR 'resistance training':ti,ab OR 'strength training':ti,ab OR 'high intensity interval training':ti,ab OR hiit:ti,ab OR 'interval training':ti,ab OR 'circuit training':ti,ab OR sport*:ti,ab) AND ('cardiorespiratory fitness'/exp OR 'cardiorespiratory fitness' OR 'maximal oxygen uptake'/exp OR 'maximal oxygen uptake' OR 'exercise tolerance'/exp OR 'exercise tolerance' OR 'cardiorespiratory fitness':ti,ab OR 'cardiopulmonary fitness':ti,ab OR 'cardiorespiratory capacity':ti,ab OR 'cardiopulmonary function':ti,ab OR 'aerobic capacity':ti,ab OR 'exercise capacity':ti,ab OR vo2max:ti,ab OR 'vo2 max':ti,ab OR vo2peak:ti,ab OR 'peak vo2':ti,ab OR 'maximal oxygen uptake':ti,ab OR 'peak oxygen uptake':ti,ab OR cpet:ti,ab OR 'cardiopulmonary exercise test':ti,ab OR '6mwt':ti,ab OR '6-minute walk':ti,ab) 1710 28-Dec-25  #2 #1 AND 'randomized controlled trial'/de 192 28-Dec-25 |

**P-value Table for Each Outcome Measure**

**Table S3** Global inconsistency analysis p-values for each outcome measure

| outcome measures | p-value of the global inconsistency analysis |
| --- | --- |
| VO_2_peak | 0.8595 |
| V0_2_amx | 0.8641 |
| HR_max_ | 0.8736 |
| RHR | 0.7276 |
| SBP | 0.9622 |
| DBP | 0.7327 |
| MAS | 0.7186 |

**Node Splitting Diagram**

| **Side** | **Coef.** | **Std. Err.** | **Coef.** | **Std. Err.** | **Coef.** | **Std. Err.** | **P>z** | **tau** |
| --- | --- | --- | --- | --- | --- | --- | --- | --- |
| AT VS MIIT | 1.800001 | 2.058676 | 2.803375 | 4.129449 | -1.003375 | 4.617221 | 0.828 | 1.8418 |
| ATVS MIIT-AT | 7 | 1.963691 | -0.7020989 | 321.9928 | 1.402099 | 321.999 | 0.997 | 1.75683 |
| AT VS SOC | -0.7 | 2.0532 | -1.704212 | 4.137557 | 1.004212 | 4.617167 | 0.828 | 1.841802 |
| HIIT VS HIIT-AT | 0.9025128 | 1.695524 | -3.254364 | 2.162579 | 4.156877 | 2.738861 | 0.129 | 1.645836 |
| HIIT VS MICT | -1.831134 | 1.059904 | -2.335047 | 2.827384 | 0.5039123 | 3.014097 | 0.867 | 1.83821 |
| HIIT VS MIIT | -2.294628 | 1.111675 | 0.8051136 | 1.268154 | -3.099741 | 1.683825 | 0.066 | 1.513951 |
| HIIT VS NUT | -4.687665 | 2.373614 | -6.277971 | 1.925603 | 1.590306 | 3.077639 | 0.605 | 1.78719 |
| HIIT VS SOC | -3.848536 | 0.8053531 | -3.619599 | 1.913372 | -0.2289373 | 2.078193 | 0.912 | 1.836459 |
| HIIT-AT VS SOC | -2.340534 | 1.434851 | -7.405845 | 3.349836 | 5.065311 | 3.64223 | 0.164 | 1.681259 |
| LICT VS SOC | -2.601796 | 1.590827 | -1.659772 | 761.7644 | -0.9420239 | 761.7661 | 0.999 | 1.756822 |
| MICT VS NUT | -0.7993006 | 2.169179 | -6.400259 | 1.976076 | 5.600959 | 2.939463 | 0.057 | 1.574333 |
| MICT VS SOC | -2.603387 | 1.410058 | -1.028744 | 1.60745 | -1.574642 | 2.131018 | 0.46 | 1.784529 |
| MIIT VS MIIT-AT | -1.1 | 2.043557 | -2.104082 | 4.123033 | 1.004082 | 4.618236 | 0.828 | 1.841839 |
| MIIT VS NUT | -6.3 | 1.679595 | -2.210551 | 2.179641 | -4.089449 | 2.751704 | 0.137 | 1.64703 |
| MIIT VS SOC | -3.24039 | 1.080368 | -1.97299 | 1.753321 | -1.2674 | 2.054052 | 0.537 | 1.808197 |

**Table S4** Node-splitting diagram for the outcome measure of VO_2_peak

**Table S5** Node Splitting Diagram of the Outcome Measure VO_2_max

| **Side** | **Coef.** | **Std. Err.** | **Coef.** | **Std. Err.** | **Coef.** | **Std. Err.** | **P>z** | **tau** |
| --- | --- | --- | --- | --- | --- | --- | --- | --- |
| HICT-AT VS SOC | -3.8 | 2.956844 | -1.557517 | 223.5388 | -2.242483 | 223.5583 | 0.992 | 2.341366 |
| HIIT VS MIIT | -1.490769 | 1.686434 | 2.18115 | 2.788714 | -3.671919 | 3.258013 | 0.26 | 2.298593 |
| HIIT VS MIIT-AT | -0.8600001 | 2.358505 | -10.15238 | 4.457013 | 9.292383 | 4.850082 | 0.055 | 1.980878 |
| HIIT VS SOC | -2.418649 | 1.30802 | -4.185307 | 5.790966 | 1.766659 | 5.936237 | 0.766 | 2.51129 |
| HIIT-AT VS SOC | -2.2 | 2.41535 | -4.866053 | 1047.361 | 2.666053 | 1047.364 | 0.998 | 2.341349 |
| HIIT-MICT VS SOC | -5.2 | 2.510162 | -7.864784 | 1047.737 | 2.664784 | 1047.74 | 0.998 | 2.341348 |
| LICT VS MICT | 0.13 | 2.813852 | -0.2492096 | 6.384218 | 0.3792096 | 6.898384 | 0.956 | 2.495089 |
| LICT VS MIIT | 1.199696 | 2.607793 | -0.0953079 | 2.673337 | 1.295004 | 3.735612 | 0.729 | 2.495992 |
| LICT VS SOC | -1.560177 | 1.630716 | 0.2059517 | 5.704436 | -1.766129 | 5.936496 | 0.766 | 2.511303 |
| MICT VS SOC | -1.42 | 3.006103 | -1.799255 | 6.115684 | 0.3792545 | 6.898384 | 0.956 | 2.495092 |
| MICT-AT VS SOC | -1.96609 | 2.188505 | -7.697284 | 2414.964 | 5.731194 | 2414.965 | 0.998 | 2.341338 |
| MIIT VS SOC | -2.449866 | 1.419941 | 1.015664 | 3.640011 | -3.465531 | 3.90375 | 0.375 | 2.362975 |
| MIIT-AT VS SOC | 1.91 | 2.333213 | -7.382404 | 4.496765 | 9.292404 | 4.850077 | 0.055 | 1.980877 |

**Table S6** Node Splitting Diagram of the Outcome Measure HRmax

| **Side** | **Coef.** | **Std. Err.** | **Coef.** | **Std. Err.** | **Coef.** | **Std. Err.** | **P>z** | **tau** |
| --- | --- | --- | --- | --- | --- | --- | --- | --- |
| HIIT VS LICT | -2.273454 | 5.278924 | -3.490508 | 4.009755 | 1.217054 | 6.56358 | 0.853 | 4.578452 |
| HIIT VS MICT | 1.87712 | 3.428167 | -7.293521 | 8.685895 | 9.170642 | 9.329462 | 0.326 | 4.345833 |
| HIIT VS MIIT | -0.7361508 | 2.432942 | 3.392315 | 3.526523 | -4.128466 | 4.284073 | 0.335 | 4.411373 |
| HIIT VS SOC | 0.1665051 | 1.957251 | -0.233684 | 4.692876 | 0.4001891 | 5.100135 | 0.937 | 4.573345 |
| LICT VS MIIT | -0.021344 | 4.550539 | 6.695398 | 4.276889 | -6.716742 | 6.24915 | 0.282 | 4.441344 |
| LICT VS SOC | 4.247509 | 3.329737 | -1.74521 | 6.883459 | 5.992718 | 7.671956 | 0.435 | 4.530414 |
| MICT VS SOC | 1.98464 | 4.553854 | -3.686522 | 5.085056 | 5.671162 | 6.837454 | 0.407 | 4.392269 |
| MIIT VS SOC | -1.695176 | 1.919102 | 5.864995 | 4.405603 | -7.560171 | 4.819889 | 0.117 | 4.168702 |

**Table S7** Node Splitting Diagram of the Outcome Measure SBP

| **Side** | **Coef.** | **Std. Err.** | **Coef.** | **Std. Err.** | **Coef.** | **Std. Err.** | **P>z** | **tau** |
| --- | --- | --- | --- | --- | --- | --- | --- | --- |
| HIIT VS LICT | 3.460646 | 6.580879 | -0.3954541 | 2.877454 | 3.8561 | 7.193245 | 0.592 | 3.740578 |
| HIIT VS MICT | -0.0053723 | 4.384359 | -8.791098 | 3.749642 | 8.785726 | 5.75255 | 0.127 | 3.474214 |
| HIIT VS MIIT | -2.672419 | 2.252229 | -1.463782 | 3.151497 | -1.208637 | 3.87276 | 0.755 | 3.79231 |
| HIIT VS MIIT-AT | -2.192942 | 5.973519 | -3.715477 | 4.922217 | 1.522534 | 7.644974 | 0.842 | 3.753117 |
| HIIT VS SOC | 1.726097 | 1.84984 | 5.856669 | 3.27797 | -4.130572 | 3.755471 | 0.271 | 3.600799 |
| HIIT-AT VS SOC | 1 | 6.047259 | 10.56406 | 5978.165 | -9.564057 | 5978.168 | 0.999 | 3.661604 |
| HIIT-MICT VS SOC | -7.2 | 5.583399 | 2.365121 | 5975.303 | -9.565121 | 5975.307 | 0.999 | 3.661603 |
| LICT VS MIIT | 0.0158462 | 4.089896 | -3.982598 | 3.175954 | 3.998445 | 5.174726 | 0.44 | 3.69486 |
| LICT VS SOC | 2.264407 | 2.359489 | 4.434263 | 6.902222 | -2.169856 | 7.329838 | 0.767 | 3.74625 |
| LICT-NUT VS MICT-NUT | -6 | 4.387311 | -8.002628 | 9.985494 | 2.002629 | 10.86087 | 0.854 | 3.814249 |
| LICT-NUT VS NUT | 4.750004 | 4.23369 | -12.78068 | 6526.376 | 17.53068 | 6526.377 | 0.998 | 3.661594 |
| LICT-NUT VS SOC | -4 | 4.45558 | -1.997212 | 9.894372 | -2.002788 | 10.86086 | 0.854 | 3.814249 |
| MICT VS SOC | 8.734169 | 2.777134 | -1.994513 | 8.32996 | 10.72868 | 8.668616 | 0.216 | 3.586247 |
| MICT-AT VS L | 7.8 | 5.034035 | 5.432907 | 5306.213 | 2.367093 | 5306.215 | 1 | 3.661603 |
| MICT-NUT VS SOC | 2.628165 | 2.502758 | 9.336876 | 3077.559 | -6.708711 | 3077.56 | 0.998 | 3.661618 |
| MIIT VS SOC | 5.270037 | 1.832919 | 3.857054 | 3.57483 | 1.412983 | 4.020267 | 0.725 | 3.761635 |
| MIIT-AT VS SOC | 6.060915 | 3.901285 | 3.970564 | 10.70087 | 2.09035 | 11.38817 | 0.854 | 3.747625 |

**Table S8** Node Splitting Diagram of the Outcome Measure DBP

| **Side** | **Coef.** | **Std. Err.** | **Coef.** | **Std. Err.** | **Coef.** | **Std. Err.** | **P>z** | **tau** |
| --- | --- | --- | --- | --- | --- | --- | --- | --- |
| HIIT VS LICT | 2.836853 | 4.400435 | -0.4910835 | 2.430868 | 3.327937 | 4.966135 | 0.503 | 3.132672 |
| HIIT VS MIIT | -3.829584 | 1.724078 | 1.337531 | 2.481114 | -5.167115 | 3.019676 | 0.087 | 2.824938 |
| HIIT VS MIIT-AT | 1.002338 | 3.395021 | -3.97475 | 3.790007 | 4.977088 | 5.090738 | 0.328 | 3.094546 |
| HIIT VS SOC | 2.221102 | 1.58769 | 0.5813427 | 3.033137 | 1.639759 | 3.437747 | 0.633 | 3.188429 |
| HIIT-AT VS SOC | 1.200005 | 3.894197 | 2.929032 | 3268.103 | -1.729027 | 3268.105 | 1 | 3.091079 |
| HIIT-MICT VS SOC | 1.799995 | 3.736681 | 3.51385 | 3256.215 | -1.713855 | 3256.217 | 1 | 3.091081 |
| LICT VS MIIT | -0.0103389 | 3.403908 | -3.912811 | 2.637275 | 3.902472 | 4.31075 | 0.365 | 3.073864 |
| LICT VS SOC | 1.84434 | 2.012248 | -0.1151153 | 5.414601 | 1.959456 | 5.749117 | 0.733 | 3.241643 |
| LICT-NUT VS MICT-NUT | -2 | 3.724285 | -3.414659 | 8.548752 | 1.414659 | 9.377101 | 0.88 | 3.226389 |
| LICT-NUT VS NUT | 5.9 | 3.49383 | 0.7317206 | 5019.116 | 5.168279 | 5019.118 | 0.999 | 3.091085 |
| LICT-NUT VS SOC | 2 | 3.820864 | 3.414702 | 8.4199 | -1.414702 | 9.377083 | 0.88 | 3.226389 |
| MICT VS SOC | -3.1 | 3.21075 | 3.725204 | 1712.731 | -6.825204 | 1712.734 | 0.997 | 3.091087 |
| MICT-AT VS SOC | 1.600003 | 4.358117 | 3.721721 | 4720.57 | -2.121718 | 4720.572 | 1 | 3.091092 |
| MICT-NUT VS SOC | 4.483597 | 2.106795 | 4.797993 | 2480.226 | -0.3143963 | 2480.227 | 1 | 3.091085 |
| MIIT VS SOC | 3.838005 | 1.609256 | 4.82942 | 3.091866 | -0.9914149 | 3.482235 | 0.776 | 3.224971 |

**Table S9** Node Splitting Diagram of the Outcome Measure MAS

| **Side** | **Coef.** | **Std. Err.** | **Coef.** | **Std. Err.** | **Coef.** | **Std. Err.** | **P>z** | **tau** |
| --- | --- | --- | --- | --- | --- | --- | --- | --- |
| HIIT VS HIIT-AT | -9.04E-12 | 0.3903891 | -0.9713896 | 0.8600642 | 0.9713896 | 0.958071 | 0.311 | 0.3190898 |
| HIIT VS MICT | -0.4486404 | 0.4037314 | -0.4448119 | 0.8630243 | -0.0038285 | 0.9467562 | 0.997 | 0.3704868 |
| HIIT VS MIIT | -0.6324342 | 0.2883905 | 0.1832716 | 0.8074644 | -0.8157058 | 0.8686268 | 0.348 | 0.3139114 |
| HIIT VS SOC | -1.295018 | 0.2608376 | -0.7766404 | 1.130366 | -0.5183777 | 1.165988 | 0.657 | 0.3724734 |
| HIIT-AT VS SOC | -0.9 | 0.3743503 | -1.87139 | 0.8811961 | 0.9713896 | 0.9580709 | 0.311 | 0.3190898 |
| MICT VS SOC | -0.8242921 | 0.3693004 | -0.8204635 | 0.8925545 | -0.0038285 | 0.9467563 | 0.997 | 0.3704868 |
| MIIT VS SOC | -0.8005685 | 0.3881259 | -0.5578281 | 0.5149786 | -0.2427404 | 0.642472 | 0.706 | 0.3711636 |
| Side | Coef. | Std. Err. | Coef. | Std. Err. | Coef. | Std. Err. | P>z | tau |

**Table S10** Node Splitting Diagram of the Outcome Measure RHR

| **Side** | **Coef.** | **Std. Err.** | **Coef.** | **Std. Err.** | **Coef.** | **Std. Err.** | **P>z** | **tau** |
| --- | --- | --- | --- | --- | --- | --- | --- | --- |
| HIIT VS HIIT-NUT | -1.1 | 4.238565 | -8.120001 | 12.41085 | 7.020001 | 13.1126 | 0.592 | 4.213239 |
| HIIT VS LICT | 2.149223 | 6.881587 | -1.063611 | 4.452848 | 3.212834 | 8.231479 | 0.696 | 4.142342 |
| HIIT VS MIIT | 0.3173287 | 3.346623 | 2.227926 | 5.546598 | -1.910597 | 6.467982 | 0.768 | 4.40109 |
| HIIT VS SOC | 6.89152 | 2.942957 | 3.336026 | 6.392251 | 3.555493 | 6.989616 | 0.611 | 4.326982 |
| HIIT-NUT VS MICT | 2.71 | 4.23884 | -4.31 | 12.41054 | 7.02 | 13.11258 | 0.592 | 4.21323 |
| LICT VS MIIT | 1.042509 | 4.54661 | 0.6776811 | 6.237599 | 0.3648277 | 7.711272 | 0.962 | 4.303692 |
| LICT VS SOC | 6.938692 | 3.794923 | 2.991333 | 9.460496 | 3.947359 | 10.16898 | 0.698 | 4.433133 |
| LICT-NUT VS MICT-NUT | 3.721603 | 2.266999 | 3.82446 | 24.32222 | -0.1028576 | 24.19023 | 0.997 | 0.9999999 |
| LICT-NUT VS SOC | -1 | 4.593809 | 15.04554 | 5856.746 | -16.04554 | 5856.748 | 0.998 | 3.802034 |
| MICT-AT VS SOC | 7 | 4.010264 | 12.22255 | 1936.893 | -5.222554 | 1936.897 | 0.998 | 3.802046 |
| MICT-NUT VS SOC | -4 | 4.54024 | 12.04946 | 5875.904 | -16.04946 | 5875.907 | 0.998 | 3.802032 |

**SUCRA value**

**Table S11** SUCRA Value Table of the Outcome Measure VO_2_peak

| **Treatm~t** | **SUCRA** | **PrBest** | **MeanRank** |
| --- | --- | --- | --- |
| AT | 35.5 | 2.8 | 6.2 |
| HIIT | 88.9 | 42.4 | 1.9 |
| HIIT-AT | 72.1 | 22.5 | 3.2 |
| LICT | 62.9 | 17.6 | 4 |
| MICT | 50.7 | 1.1 | 4.9 |
| MIIT | 70.3 | 7.7 | 3.4 |
| MIIT-AT | 47.1 | 5.9 | 5.2 |
| NUT | 4 | 0 | 8.7 |
| SOC | 18.5 | 0 | 7.5 |

**Table S12** SUCRA Value Table of the Outcome Measure VO_2_max

| **Treatm~t** | **SUCRA** | **PrBest** | **MeanRank** |
| --- | --- | --- | --- |
| HIIT-MICT | 85.9 | 48.5 | 2.3 |
| HICT-AT | 70 | 28.1 | 3.7 |
| HIIT | 61.1 | 2.9 | 4.5 |
| HIIT-AT | 52.6 | 4.1 | 5.3 |
| MIIT | 52.3 | 1.9 | 5.3 |
| MICT-AT | 50.9 | 6.4 | 5.4 |
| MICT | 43.4 | 5.8 | 6.1 |
| LICT | 42.8 | 1 | 6.2 |
| MIIT-AT | 23.8 | 1.2 | 7.9 |
| SOC | 17.3 | 0 | 8.4 |

**Table S13** SUCRA Value Table of the Outcome Measure HR_max_

| **Treatm~t** | **SUCRA** | **PrBest** | **MeanRank** |
| --- | --- | --- | --- |
| LICT | 83 | 67.1 | 1.7 |
| HIIT | 47.9 | 7.4 | 3.1 |
| SOC | 44.3 | 5.3 | 3.2 |
| MICT | 39.2 | 15.6 | 3.4 |
| MIIT | 35.6 | 4.6 | 3.6 |

**Table S14** SUCRA Value Table of the Outcome Measure SBP

| **Treatm~t** | **SUCRA** | **PrBest** | **MeanRank** |
| --- | --- | --- | --- |
| MICT | 87.8 | 33.2 | 2.3 |
| MICT-AT | 81.3 | 38.6 | 3.1 |
| MIIT-AT | 75 | 16.3 | 3.7 |
| MIIT | 73.8 | 2.8 | 3.9 |
| MICT-NUT | 55.1 | 1.1 | 5.9 |
| HIIT | 54.3 | 0.1 | 6 |
| LICT | 53.1 | 0.6 | 6.2 |
| HIIT-AT | 46.9 | 7.1 | 6.8 |
| SOC | 32.3 | 0 | 8.4 |
| LICT-NUT | 20.6 | 0.1 | 9.7 |
| HIIT-MICT | 11.5 | 0 | 10.7 |
| NUT | 8.2 | 0.1 | 11.1 |

**Table S15** SUCRA Value Table of the Outcome Measure DBP

| **Treatm~t** | **SUCRA** | **PrBest** | **MeanRank** |
| --- | --- | --- | --- |
| MICT-NUT | 80.3 | 24.8 | 3.2 |
| MIIT | 78.5 | 13 | 3.4 |
| MIIT-AT | 66.6 | 12.3 | 4.7 |
| LICT-NUT | 58 | 11.2 | 5.6 |
| HIIT | 53 | 0.5 | 6.2 |
| HIIT-MICT | 52.9 | 11.4 | 6.2 |
| MICT-AT | 51.4 | 14.9 | 6.4 |
| LICT | 50.2 | 1.8 | 6.5 |
| HIIT-AT | 47.5 | 8.8 | 6.8 |
| SOC | 29.9 | 0 | 8.7 |
| NUT | 16.5 | 1 | 10.2 |
| MICT | 15.5 | 0.3 | 10.3 |

**Table S16** SUCRA Value Table of the Outcome Measure MAS

| **Treatm~t** | **SUCRA** | **PrBest** | **MeanRank** |
| --- | --- | --- | --- |
| HIIT | 90.3 | 66 | 1.4 |
| HIIT-AT | 70.9 | 25.8 | 2.2 |
| MICT | 50.1 | 7.5 | 3 |
| MIIT | 38.4 | 0.7 | 3.5 |
| SOC | 0.2 | 0 | 5 |

**Table S17** SUCRA Value Table of the Outcome Measure RHR

| **Treatm~t** | **SUCRA** | **PrBest** | **MeanRank** |
| --- | --- | --- | --- |
| MICT-NUT | 91.2 | 63.4 | 1.7 |
| SOC | 78.8 | 10.8 | 2.7 |
| LICT-NUT | 76.1 | 19.6 | 2.9 |
| MIIT | 48.2 | 1.6 | 5.1 |
| MICT | 38 | 2.2 | 6 |
| LICT | 34.2 | 0.9 | 6.3 |
| HIIT | 33.9 | 0.1 | 6.3 |
| MICT-AT | 27.1 | 0.7 | 6.8 |
| HIIT-NUT | 22.5 | 0.8 | 7.2 |

**Sensitivity Analysis**

**Table S18** Sensitivity Analysis Using VO_2_peak as the Outcome Measure.

| **dropped_id** | **comparison** | **eff** | **lci** | **uci** | **connected** |
| --- | --- | --- | --- | --- | --- |
| [Angela S Alberga 2015](https://pubmed.ncbi.nlm.nih.gov/?term=Alberga+AS&cauthor_id=26881317) | HIIT VS SOC | 1.1573237 | 0.4038594 | 1.910788 | 1 |
| [Angela S Alberga 2015](https://pubmed.ncbi.nlm.nih.gov/?term=Alberga+AS&cauthor_id=26881317) | HIIT-AT VS SOC | 1.1197558 | -0.3324632 | 2.571975 | 1 |
| [Angela S Alberga 2015](https://pubmed.ncbi.nlm.nih.gov/?term=Alberga+AS&cauthor_id=26881317) | LICT VS SOC | 1.084261 | -0.4892434 | 2.657765 | 1 |
| [Angela S Alberga 2015](https://pubmed.ncbi.nlm.nih.gov/?term=Alberga+AS&cauthor_id=26881317) | MICT VS SOC | 0.61388292 | -0.4824029 | 1.710169 | 1 |
| [Angela S Alberga 2015](https://pubmed.ncbi.nlm.nih.gov/?term=Alberga+AS&cauthor_id=26881317) | MIIT VS SOC | 1.3257283 | 0.2496186 | 2.401838 | 1 |
| [Angela S Alberga 2015](https://pubmed.ncbi.nlm.nih.gov/?term=Alberga+AS&cauthor_id=26881317) | NUT VS SOC | -1.3365934 | -3.111663 | 0.4384758 | 1 |
| [A C Benson 2008](https://pubmed.ncbi.nlm.nih.gov/?term=Benson+AC&cauthor_id=18283282) | AT VS SOC | 0.48376545 | -1.344572 | 2.312103 | 1 |
| [A C Benson 2008](https://pubmed.ncbi.nlm.nih.gov/?term=Benson+AC&cauthor_id=18283282) | HIIT VS SOC | 1.1694838 | 0.4510107 | 1.887957 | 1 |
| [A C Benson 2008](https://pubmed.ncbi.nlm.nih.gov/?term=Benson+AC&cauthor_id=18283282) | HIIT-AT VS SOC | 1.8654345 | -0.0002747 | 3.731144 | 1 |
| [A C Benson 2008](https://pubmed.ncbi.nlm.nih.gov/?term=Benson+AC&cauthor_id=18283282) | LICT VS SOC | 1.0845444 | -0.4217112 | 2.5908 | 1 |
| [A C Benson 2008](https://pubmed.ncbi.nlm.nih.gov/?term=Benson+AC&cauthor_id=18283282) | MICT VS SOC | 0.62089098 | -0.4276119 | 1.669394 | 1 |
| [A C Benson 2008](https://pubmed.ncbi.nlm.nih.gov/?term=Benson+AC&cauthor_id=18283282) | MIIT VS SOC | 1.1662029 | 0.2420585 | 2.090347 | 1 |
| [A C Benson 2008](https://pubmed.ncbi.nlm.nih.gov/?term=Benson+AC&cauthor_id=18283282) | MIIT-AT VS SOC | 0.61011997 | -1.218759 | 2.438999 | 1 |
| [A C Benson 2008](https://pubmed.ncbi.nlm.nih.gov/?term=Benson+AC&cauthor_id=18283282) | NUT VS SOC | -1.332504 | -3.041404 | 0.3763963 | 1 |
| [Napasakorn Chuensiri 2018](https://pubmed.ncbi.nlm.nih.gov/?term=Chuensiri+N&cauthor_id=29099231) | AT VS SOC | 0.44771137 | -1.571885 | 2.467308 | 1 |
| [Napasakorn Chuensiri 2018](https://pubmed.ncbi.nlm.nih.gov/?term=Chuensiri+N&cauthor_id=29099231) | HIIT VS SOC | 1.1401389 | 0.3208133 | 1.959465 | 1 |
| [Napasakorn Chuensiri 2018](https://pubmed.ncbi.nlm.nih.gov/?term=Chuensiri+N&cauthor_id=29099231) | HIIT-AT VS SOC | 1.1159094 | -0.4026114 | 2.63443 | 1 |
| [Napasakorn Chuensiri 2018](https://pubmed.ncbi.nlm.nih.gov/?term=Chuensiri+N&cauthor_id=29099231) | LICT VS SOC | 1.0840059 | -0.5582336 | 2.726245 | 1 |
| [Napasakorn Chuensiri 2018](https://pubmed.ncbi.nlm.nih.gov/?term=Chuensiri+N&cauthor_id=29099231) | MICT VS SOC | 0.58854214 | -0.5658466 | 1.742931 | 1 |
| [Napasakorn Chuensiri 2018](https://pubmed.ncbi.nlm.nih.gov/?term=Chuensiri+N&cauthor_id=29099231) | MIIT VS SOC | 1.0940411 | -0.0219041 | 2.209986 | 1 |
| [Napasakorn Chuensiri 2018](https://pubmed.ncbi.nlm.nih.gov/?term=Chuensiri+N&cauthor_id=29099231) | MIIT-AT VS SOC | 0.57404998 | -1.446041 | 2.594141 | 1 |
| [Napasakorn Chuensiri 2018](https://pubmed.ncbi.nlm.nih.gov/?term=Chuensiri+N&cauthor_id=29099231) | NUT VS SOC | -1.4672948 | -3.312046 | 0.3774566 | 1 |
| [Katrin A Dias 2018](https://pubmed.ncbi.nlm.nih.gov/?term=Dias+KA&cauthor_id=28853029) | AT VS SOC | 0.26389856 | -0.9116201 | 1.439417 | 1 |
| [Katrin A Dias 2018](https://pubmed.ncbi.nlm.nih.gov/?term=Dias+KA&cauthor_id=28853029) | HIIT VS SOC | 1.2154792 | 0.7202541 | 1.710704 | 1 |
| [Katrin A Dias 2018](https://pubmed.ncbi.nlm.nih.gov/?term=Dias+KA&cauthor_id=28853029) | HIIT-AT VS SOC | 1.1170689 | 0.1950106 | 2.039127 | 1 |
| [Katrin A Dias 2018](https://pubmed.ncbi.nlm.nih.gov/?term=Dias+KA&cauthor_id=28853029) | LICT VS SOC | 1.0885546 | 0.0802334 | 2.096876 | 1 |
| [Katrin A Dias 2018](https://pubmed.ncbi.nlm.nih.gov/?term=Dias+KA&cauthor_id=28853029) | MICT VS SOC | 0.95046302 | 0.1719987 | 1.728927 | 1 |
| [Katrin A Dias 2018](https://pubmed.ncbi.nlm.nih.gov/?term=Dias+KA&cauthor_id=28853029) | MIIT VS SOC | 0.72645663 | 0.0924875 | 1.360426 | 1 |
| [Katrin A Dias 2018](https://pubmed.ncbi.nlm.nih.gov/?term=Dias+KA&cauthor_id=28853029) | MIIT-AT VS SOC | 0.39024926 | -0.7861179 | 1.566616 | 1 |
| [Katrin A Dias 2018](https://pubmed.ncbi.nlm.nih.gov/?term=Dias+KA&cauthor_id=28853029) | NUT VS SOC | -5.8838652 | -8.218555 | -3.549175 | 1 |
| [Catherine L Davis 2020](https://pubmed.ncbi.nlm.nih.gov/?term=Davis+CL&cauthor_id=31754238) | AT VS SOC | 0.47747604 | -1.465434 | 2.420386 | 1 |
| [Catherine L Davis 2020](https://pubmed.ncbi.nlm.nih.gov/?term=Davis+CL&cauthor_id=31754238) | HIIT VS SOC | 1.1102418 | 0.3582071 | 1.862277 | 1 |
| [Catherine L Davis 2020](https://pubmed.ncbi.nlm.nih.gov/?term=Davis+CL&cauthor_id=31754238) | HIIT-AT VS SOC | 1.1064598 | -0.3651082 | 2.578028 | 1 |
| [Catherine L Davis 2020](https://pubmed.ncbi.nlm.nih.gov/?term=Davis+CL&cauthor_id=31754238) | LICT VS SOC | 1.0416194 | -1.306409 | 3.389648 | 1 |
| [Catherine L Davis 2020](https://pubmed.ncbi.nlm.nih.gov/?term=Davis+CL&cauthor_id=31754238) | MICT VS SOC | 0.57138041 | -0.5331712 | 1.675932 | 1 |
| [Catherine L Davis 2020](https://pubmed.ncbi.nlm.nih.gov/?term=Davis+CL&cauthor_id=31754238) | MIIT VS SOC | 1.1535966 | 0.1785338 | 2.128659 | 1 |
| [Catherine L Davis 2020](https://pubmed.ncbi.nlm.nih.gov/?term=Davis+CL&cauthor_id=31754238) | MIIT-AT VS SOC | 0.60382233 | -1.339597 | 2.547242 | 1 |
| [Catherine L Davis 2020](https://pubmed.ncbi.nlm.nih.gov/?term=Davis+CL&cauthor_id=31754238) | NUT VS SOC | -1.4345817 | -3.216535 | 0.3473716 | 1 |
| [Ragab K Elnaggar 2021](https://pubmed.ncbi.nlm.nih.gov/?term=Elnaggar+RK&cauthor_id=33761464) | AT VS SOC | 0.48219878 | -1.47269 | 2.437087 | 1 |
| [Ragab K Elnaggar 2021](https://pubmed.ncbi.nlm.nih.gov/?term=Elnaggar+RK&cauthor_id=33761464) | HIIT VS SOC | 1.1128199 | 0.3516518 | 1.873988 | 1 |
| [Ragab K Elnaggar 2021](https://pubmed.ncbi.nlm.nih.gov/?term=Elnaggar+RK&cauthor_id=33761464) | HIIT-AT | 1.1071896 | -0.3638099 | 2.578189 | 1 |
| [Ragab K Elnaggar 2021](https://pubmed.ncbi.nlm.nih.gov/?term=Elnaggar+RK&cauthor_id=33761464) | LICT | 1.0841835 | -0.509305 | 2.677672 | 1 |
| [Ragab K Elnaggar 2021](https://pubmed.ncbi.nlm.nih.gov/?term=Elnaggar+RK&cauthor_id=33761464) | MICT | 0.57370359 | -0.5351185 | 1.682526 | 1 |
| [Ragab K Elnaggar 2021](https://pubmed.ncbi.nlm.nih.gov/?term=Elnaggar+RK&cauthor_id=33761464) | MIIT | 1.1630448 | 0.0870769 | 2.239013 | 1 |
| [Ragab K Elnaggar 2021](https://pubmed.ncbi.nlm.nih.gov/?term=Elnaggar+RK&cauthor_id=33761464) | MIIT-AT | 0.60854589 | -1.346853 | 2.563945 | 1 |
| [Ragab K Elnaggar 2021](https://pubmed.ncbi.nlm.nih.gov/?term=Elnaggar+RK&cauthor_id=33761464) | NUT | -1.429266 | -3.221566 | 0.3630346 | 1 |
| [Danielle Lambrick 2016](https://pubmed.ncbi.nlm.nih.gov/?term=Lambrick+D&cauthor_id=26009003) | AT | 0.49818595 | -1.390175 | 2.386547 | 1 |
| [Danielle Lambrick 2016](https://pubmed.ncbi.nlm.nih.gov/?term=Lambrick+D&cauthor_id=26009003) | HIIT | 1.2177615 | 0.4411329 | 1.99439 | 1 |
| [Danielle Lambrick 2016](https://pubmed.ncbi.nlm.nih.gov/?term=Lambrick+D&cauthor_id=26009003) | HIIT-AT | 1.1369149 | -0.2963749 | 2.570205 | 1 |
| [Danielle Lambrick 2016](https://pubmed.ncbi.nlm.nih.gov/?term=Lambrick+D&cauthor_id=26009003) | LICT | 1.0843482 | -0.4675138 | 2.63621 | 1 |
| [Danielle Lambrick 2016](https://pubmed.ncbi.nlm.nih.gov/?term=Lambrick+D&cauthor_id=26009003) | MICT | 0.65485931 | -0.4388474 | 1.748566 | 1 |
| [Danielle Lambrick 2016](https://pubmed.ncbi.nlm.nih.gov/?term=Lambrick+D&cauthor_id=26009003) | MIIT | 1.1950399 | 0.2382257 | 2.151854 | 1 |
| [Danielle Lambrick 2016](https://pubmed.ncbi.nlm.nih.gov/?term=Lambrick+D&cauthor_id=26009003) | MIIT-AT | 0.62453891 | -1.264347 | 2.513425 | 1 |
| [Danielle Lambrick 2016](https://pubmed.ncbi.nlm.nih.gov/?term=Lambrick+D&cauthor_id=26009003) | NUT | -1.3273648 | -3.083757 | 0.4290278 | 1 |
| [Cao Meng 2022](https://pubmed.ncbi.nlm.nih.gov/?term=Meng+C&cauthor_id=35232402) | AT | 0.43255382 | -1.454533 | 2.319641 | 1 |
| [Cao Meng 2022](https://pubmed.ncbi.nlm.nih.gov/?term=Meng+C&cauthor_id=35232402) | HIIT | 0.93143565 | 0.154467 | 1.708404 | 1 |
| [Cao Meng 2022](https://pubmed.ncbi.nlm.nih.gov/?term=Meng+C&cauthor_id=35232402) | HIIT-AT | 1.0537586 | -0.378584 | 2.486101 | 1 |
| [Cao Meng 2022](https://pubmed.ncbi.nlm.nih.gov/?term=Meng+C&cauthor_id=35232402) | LICT | 1.0843524 | -0.4664856 | 2.63519 | 1 |
| [Cao Meng 2022](https://pubmed.ncbi.nlm.nih.gov/?term=Meng+C&cauthor_id=35232402) | MICT | 0.33378441 | -0.8963846 | 1.563953 | 1 |
| [Cao Meng 2022](https://pubmed.ncbi.nlm.nih.gov/?term=Meng+C&cauthor_id=35232402) | MIIT | 1.063739 | 0.1070785 | 2.020399 | 1 |
| [Cao Meng 2022](https://pubmed.ncbi.nlm.nih.gov/?term=Meng+C&cauthor_id=35232402) | MIIT-AT | 0.55889603 | -1.328716 | 2.446508 | 1 |
| [Cao Meng 2022](https://pubmed.ncbi.nlm.nih.gov/?term=Meng+C&cauthor_id=35232402) | NUT | -1.5774219 | -3.351187 | 0.1963433 | 1 |
| [D M Prado 2010](https://pubmed.ncbi.nlm.nih.gov/?term=Prado+DM&cauthor_id=21072735) | AT | 0.26459611 | -0.8732021 | 1.402394 | 1 |
| [D M Prado 2010](https://pubmed.ncbi.nlm.nih.gov/?term=Prado+DM&cauthor_id=21072735) | HIIT | 1.2245522 | 0.7435613 | 1.705543 | 1 |
| [D M Prado 2010](https://pubmed.ncbi.nlm.nih.gov/?term=Prado+DM&cauthor_id=21072735) | HIIT-AT | 1.1178251 | 0.2221452 | 2.013505 | 1 |
| [D M Prado 2010](https://pubmed.ncbi.nlm.nih.gov/?term=Prado+DM&cauthor_id=21072735) | LICT | 1.0889606 | 0.1090888 | 2.068832 | 1 |
| [D M Prado 2010](https://pubmed.ncbi.nlm.nih.gov/?term=Prado+DM&cauthor_id=21072735) | MICT | 0.87969679 | 0.1708375 | 1.588556 | 1 |
| [D M Prado 2010](https://pubmed.ncbi.nlm.nih.gov/?term=Prado+DM&cauthor_id=21072735) | MIIT | 0.72786651 | 0.1114077 | 1.344325 | 1 |
| [D M Prado 2010](https://pubmed.ncbi.nlm.nih.gov/?term=Prado+DM&cauthor_id=21072735) | MIIT-AT | 0.39095114 | -0.7477241 | 1.529626 | 1 |
| [D M Prado 2010](https://pubmed.ncbi.nlm.nih.gov/?term=Prado+DM&cauthor_id=21072735) | NUT | 0.60029736 | -0.6604776 | 1.861072 | 1 |
| [G Racil 2013](https://pubmed.ncbi.nlm.nih.gov/?term=Racil+G&cauthor_id=23824463) | AT | 0.47608203 | -1.546938 | 2.499102 | 1 |
| [G Racil 2013](https://pubmed.ncbi.nlm.nih.gov/?term=Racil+G&cauthor_id=23824463) | HIIT | 1.0878098 | 0.2579874 | 1.917632 | 1 |
| [G Racil 2013](https://pubmed.ncbi.nlm.nih.gov/?term=Racil+G&cauthor_id=23824463) | HIIT-AT | 1.1009471 | -0.4302341 | 2.632128 | 1 |
| [G Racil 2013](https://pubmed.ncbi.nlm.nih.gov/?term=Racil+G&cauthor_id=23824463) | LICT | 1.0839603 | -0.5714981 | 2.739419 | 1 |
| [G Racil 2013](https://pubmed.ncbi.nlm.nih.gov/?term=Racil+G&cauthor_id=23824463) | MICT | 0.51453732 | -0.7970219 | 1.826097 | 1 |
| [G Racil 2013](https://pubmed.ncbi.nlm.nih.gov/?term=Racil+G&cauthor_id=23824463) | MIIT | 1.1507935 | 0.130794 | 2.170793 | 1 |
| [G Racil 2013](https://pubmed.ncbi.nlm.nih.gov/?term=Racil+G&cauthor_id=23824463) | MIIT-AT | 0.60242392 | -1.421086 | 2.625933 | 1 |
| [G Racil 2013](https://pubmed.ncbi.nlm.nih.gov/?term=Racil+G&cauthor_id=23824463) | NUT | -1.5003584 | -3.369339 | 0.3686219 | 1 |
| [Ghazi Racil 2015](https://pubmed.ncbi.nlm.nih.gov/?term=Racil+G&cauthor_id=26701117) | AT | 0.48350358 | -1.432508 | 2.399515 | 1 |
| [Ghazi Racil 2015](https://pubmed.ncbi.nlm.nih.gov/?term=Racil+G&cauthor_id=26701117) | HIIT | 1.1445094 | 0.3487945 | 1.940224 | 1 |
| [Ghazi Racil 2015](https://pubmed.ncbi.nlm.nih.gov/?term=Racil+G&cauthor_id=26701117) | HIIT-AT | 0.1617173 | -2.012518 | 2.335953 | 1 |
| [Ghazi Racil 2015](https://pubmed.ncbi.nlm.nih.gov/?term=Racil+G&cauthor_id=26701117) | LICT | 1.0842629 | -0.4887185 | 2.657244 | 1 |
| [Ghazi Racil 2015](https://pubmed.ncbi.nlm.nih.gov/?term=Racil+G&cauthor_id=26701117) | MICT | 0.59837126 | -0.5133407 | 1.710083 | 1 |
| [Ghazi Racil 2015](https://pubmed.ncbi.nlm.nih.gov/?term=Racil+G&cauthor_id=26701117) | MIIT | 1.1656612 | 0.1946494 | 2.136673 | 1 |
| [Ghazi Racil 2015](https://pubmed.ncbi.nlm.nih.gov/?term=Racil+G&cauthor_id=26701117) | MIIT-AT | 0.60985244 | -1.306676 | 2.526381 | 1 |
| [Ghazi Racil 2015](https://pubmed.ncbi.nlm.nih.gov/?term=Racil+G&cauthor_id=26701117) | NUT | -1.3955844 | -3.177105 | 0.3859363 | 1 |
| [Marit Salus 2022](https://pubmed.ncbi.nlm.nih.gov/?term=Salus+M&cauthor_id=36231972) | AT VS SOC | 0.45126578 | -1.4378 | 2.340332 | 1 |
| [Marit Salus 2022](https://pubmed.ncbi.nlm.nih.gov/?term=Salus+M&cauthor_id=36231972) | HIIT VS SOC | 1.0067288 | 0.232845 | 1.780612 | 1 |
| [Marit Salus 2022](https://pubmed.ncbi.nlm.nih.gov/?term=Salus+M&cauthor_id=36231972) | HIIT-AT VS SOC | 1.0756523 | -0.3580784 | 2.509383 | 1 |
| [Marit Salus 2022](https://pubmed.ncbi.nlm.nih.gov/?term=Salus+M&cauthor_id=36231972) | LICT VS SOC | 1.0843458 | -0.4681312 | 2.636823 | 1 |
| [Marit Salus 2022](https://pubmed.ncbi.nlm.nih.gov/?term=Salus+M&cauthor_id=36231972) | MICT VS SOC | 0.49530021 | -0.5975561 | 1.588156 | 1 |
| [Marit Salus 2022](https://pubmed.ncbi.nlm.nih.gov/?term=Salus+M&cauthor_id=36231972) | MIIT VS SOC | 1.1011723 | 0.1447344 | 2.05761 | 1 |
| [Marit Salus 2022](https://pubmed.ncbi.nlm.nih.gov/?term=Salus+M&cauthor_id=36231972) | MIIT-AT VS SOC | 0.57761098 | -1.31198 | 2.467202 | 1 |
| [Marit Salus 2022](https://pubmed.ncbi.nlm.nih.gov/?term=Salus+M&cauthor_id=36231972) | NUT VS SOC | -1.4850068 | -3.239423 | 0.2694089 | 1 |
| [Fabrício Vasconcellos 2016](https://pubmed.ncbi.nlm.nih.gov/?term=Vasconcellos+F&cauthor_id=26208409) | AT VS SOC | 0.47747603 | -1.465433 | 2.420386 | 1 |
| [Fabrício Vasconcellos 2016](https://pubmed.ncbi.nlm.nih.gov/?term=Vasconcellos+F&cauthor_id=26208409) | HIIT VS SOC | 1.1102418 | 0.3582071 | 1.862277 | 1 |
| [Fabrício Vasconcellos 2016](https://pubmed.ncbi.nlm.nih.gov/?term=Vasconcellos+F&cauthor_id=26208409) | HIIT-AT VS SOC | 1.1064598 | -0.3651082 | 2.578028 | 1 |
| [Fabrício Vasconcellos 2016](https://pubmed.ncbi.nlm.nih.gov/?term=Vasconcellos+F&cauthor_id=26208409) | LICT VS SOC | 1.1206063 | -1.051657 | 3.29287 | 1 |
| [Fabrício Vasconcellos 2016](https://pubmed.ncbi.nlm.nih.gov/?term=Vasconcellos+F&cauthor_id=26208409) | MICT VS SOC | 0.57138041 | -0.5331712 | 1.675932 | 1 |
| [Fabrício Vasconcellos 2016](https://pubmed.ncbi.nlm.nih.gov/?term=Vasconcellos+F&cauthor_id=26208409) | MIIT VS SOC | 1.1535966 | 0.1785336 | 2.128659 | 1 |
| [Fabrício Vasconcellos 2016](https://pubmed.ncbi.nlm.nih.gov/?term=Vasconcellos+F&cauthor_id=26208409) | MIIT-AT VS SOC | 0.60382232 | -1.339597 | 2.547242 | 1 |
| [Fabrício Vasconcellos 2016](https://pubmed.ncbi.nlm.nih.gov/?term=Vasconcellos+F&cauthor_id=26208409) | NUT VS SOC | -1.4345817 | -3.216537 | 0.3473737 | 1 |
| [Ragab K Elnaggar 2024](https://pubmed.ncbi.nlm.nih.gov/?term=Elnaggar+RK&cauthor_id=39612435) | AT VS SOC | 0.5759852 | -1.286433 | 2.438404 | 1 |
| [Ragab K Elnaggar 2024](https://pubmed.ncbi.nlm.nih.gov/?term=Elnaggar+RK&cauthor_id=39612435) | HIIT VS SOC | 0.95708588 | 0.1941731 | 1.719999 | 1 |
| [Ragab K Elnaggar 2024](https://pubmed.ncbi.nlm.nih.gov/?term=Elnaggar+RK&cauthor_id=39612435) | HIIT-AT VS SOC | 1.0605963 | -0.3432673 | 2.46446 | 1 |
| [Ragab K Elnaggar 2024](https://pubmed.ncbi.nlm.nih.gov/?term=Elnaggar+RK&cauthor_id=39612435) | LICT VS SOC | 1.0844809 | -0.4360919 | 2.605054 | 1 |
| [Ragab K Elnaggar 2024](https://pubmed.ncbi.nlm.nih.gov/?term=Elnaggar+RK&cauthor_id=39612435) | MICT VS SOC | 0.46976061 | -0.6029517 | 1.542473 | 1 |
| [Ragab K Elnaggar 2024](https://pubmed.ncbi.nlm.nih.gov/?term=Elnaggar+RK&cauthor_id=39612435) | MIIT VS SOC | 1.3506927 | 0.3022694 | 2.399116 | 1 |
| [Ragab K Elnaggar 2024](https://pubmed.ncbi.nlm.nih.gov/?term=Elnaggar+RK&cauthor_id=39612435) | MIIT-AT VS SOC | 0.70235433 | -1.1606 | 2.565309 | 1 |
| [Ragab K Elnaggar 2024](https://pubmed.ncbi.nlm.nih.gov/?term=Elnaggar+RK&cauthor_id=39612435) | NUT VS SOC | -1.41347 | -3.140829 | 0.3138894 | 1 |
| [Chongwen Zuo 2023](https://pubmed.ncbi.nlm.nih.gov/?term=Zuo+C&cauthor_id=37946224) | AT VS SOC | 0.54470665 | -1.36935 | 2.458763 | 1 |
| [Chongwen Zuo 2023](https://pubmed.ncbi.nlm.nih.gov/?term=Zuo+C&cauthor_id=37946224) | HIIT VS SOC | 1.0476399 | 0.2924819 | 1.802798 | 1 |
| [Chongwen Zuo 2023](https://pubmed.ncbi.nlm.nih.gov/?term=Zuo+C&cauthor_id=37946224) | HIIT-AT VS SOC | 1.0877919 | -0.3579497 | 2.533534 | 1 |
| [Chongwen Zuo 2023](https://pubmed.ncbi.nlm.nih.gov/?term=Zuo+C&cauthor_id=37946224) | LICT VS SOC | 1.0842892 | -0.4821251 | 2.650703 | 1 |
| [Chongwen Zuo 2023](https://pubmed.ncbi.nlm.nih.gov/?term=Zuo+C&cauthor_id=37946224) | MICT VS SOC | 0.53181699 | -0.5586932 | 1.622327 | 1 |
| [Chongwen Zuo 2023](https://pubmed.ncbi.nlm.nih.gov/?term=Zuo+C&cauthor_id=37946224) | MIIT VS SOC | 1.2881022 | 0.2703235 | 2.305881 | 1 |
| [Chongwen Zuo 2023](https://pubmed.ncbi.nlm.nih.gov/?term=Zuo+C&cauthor_id=37946224) | MIIT-AT VS SOC | 0.67106592 | -1.24351 | 2.585642 | 1 |
| [Chongwen Zuo 2023](https://pubmed.ncbi.nlm.nih.gov/?term=Zuo+C&cauthor_id=37946224) | NUT VS SOC | -1.4098676 | -3.162513 | 0.3427782 | 1 |
| Katarzyna Ługowska 2025 | AT VS SOC | 0.47847379 | -1.456133 | 2.413081 | 1 |
| Katarzyna Ługowska 2025 | HIIT VS SOC | 1.1237131 | 0.3714584 | 1.875968 | 1 |
| Katarzyna Ługowska 2025 | HIIT-AT VS SOC | 1.1102578 | -0.3554096 | 2.575925 | 1 |
| Katarzyna Ługowska 2025 | LICT VS SOC | 1.084204 | -0.5039351 | 2.672343 | 1 |
| Katarzyna Ługowska 2025 | MICT VS SOC | 0.49513794 | -0.6731287 | 1.663405 | 1 |
| Katarzyna Ługowska 2025 | MIIT VS SOC | 1.1555942 | 0.1842197 | 2.126969 | 1 |
| Katarzyna Ługowska 2025 | MIIT-AT VS SOC | 0.60482071 | -1.330298 | 2.53994 | 1 |
| Katarzyna Ługowska 2025 | NUT VS SOC | -1.4514019 | -3.230409 | 0.3276048 | 1 |
| Shitong Shao 2025 | AT VS SOC | 0.4782143 | -1.450824 | 2.407253 | 1 |
| Shitong Shao 2025 | HIIT VS SOC | 1.1241818 | 0.3741635 | 1.8742 | 1 |
| Shitong Shao 2025 | HIIT-AT VS SOC | 1.1103193 | -0.3512942 | 2.571933 | 1 |
| Shitong Shao 2025 | LICT VS SOC | 1.0842205 | -0.4996278 | 2.668069 | 1 |
| Shitong Shao 2025 | MICT VS SOC | 0.49300556 | -0.6666263 | 1.652637 | 1 |
| Shitong Shao 2025 | MIIT VS SOC | 1.1550762 | 0.1862283 | 2.123924 | 1 |
| Shitong Shao 2025 | MIIT-AT VS SOC | 0.6045615 | -1.32499 | 2.534113 | 1 |
| Shitong Shao 2025 | NUT VS SOC | -1.4493507 | -3.224474 | 0.3257726 | 1 |
| Ragab K. Elnaggar 2024 | AT VS SOC | 0.50220661 | -1.349672 | 2.354086 | 1 |
| Ragab K. Elnaggar 2024 | HIIT VS SOC | 1.2459049 | 0.4849327 | 2.006877 | 1 |
| Ragab K. Elnaggar 2024 | HIIT-AT VS SOC | 1.1445715 | -0.2621659 | 2.551309 | 1 |
| Ragab K. Elnaggar 2024 | LICT VS SOC | 1.0844669 | -0.4393414 | 2.608275 | 1 |
| Ragab K. Elnaggar 2024 | MICT VS SOC | 0.67770223 | -0.3960661 | 1.751471 | 1 |
| Ragab K. Elnaggar 2024 | MIIT VS SOC | 1.2030907 | 0.2629757 | 2.143206 | 1 |
| Ragab K. Elnaggar 2024 | MIIT-AT VS SOC | 0.62856274 | -1.223851 | 2.480977 | 1 |
| Ragab K. Elnaggar 2024 | NUT VS SOC | -1.2875317 | -3.017878 | 0.4428144 | 1 |

**Table S19** Sensitivity Analysis Using VO_2_max as the Outcome Measure.

| **dropped_id** | **comparison** | **eff** | **lci** | **uci** | **connected** |
| --- | --- | --- | --- | --- | --- |
| [Aaron L Carrel 2005](https://pubmed.ncbi.nlm.nih.gov/?term=Carrel+AL&cauthor_id=16203942) | HICT-AT VS SOC | 0.6683934 | -1.665662 | 3.002449 | 1 |
| [Aaron L Carrel 2005](https://pubmed.ncbi.nlm.nih.gov/?term=Carrel+AL&cauthor_id=16203942) | HIIT VS SOC | 1.1760813 | -0.0039266 | 2.356089 | 1 |
| [Aaron L Carrel 2005](https://pubmed.ncbi.nlm.nih.gov/?term=Carrel+AL&cauthor_id=16203942) | HIIT-AT VS SOC | 1.2216744 | -1.20736 | 3.650709 | 1 |
| [Aaron L Carrel 2005](https://pubmed.ncbi.nlm.nih.gov/?term=Carrel+AL&cauthor_id=16203942) | HIIT-MICT VS SOC | 2.887594 | 0.3536612 | 5.421527 | 1 |
| [Aaron L Carrel 2005](https://pubmed.ncbi.nlm.nih.gov/?term=Carrel+AL&cauthor_id=16203942) | LICT VS SOC | 0.42848929 | -1.137266 | 1.994245 | 1 |
| [Aaron L Carrel 2005](https://pubmed.ncbi.nlm.nih.gov/?term=Carrel+AL&cauthor_id=16203942) | MICT VS SOC | 0.40716887 | -1.786813 | 2.60115 | 1 |
| [Aaron L Carrel 2005](https://pubmed.ncbi.nlm.nih.gov/?term=Carrel+AL&cauthor_id=16203942) | MICT-AT VS SOC | 0.31109552 | -1.359246 | 1.981437 | 1 |
| [Aaron L Carrel 2005](https://pubmed.ncbi.nlm.nih.gov/?term=Carrel+AL&cauthor_id=16203942) | MIIT VS SOC | 1.0071929 | -0.2736716 | 2.288057 | 1 |
| [Aaron L Carrel 2005](https://pubmed.ncbi.nlm.nih.gov/?term=Carrel+AL&cauthor_id=16203942) | MIIT-AT VS SOC | 0.20145627 | -1.901198 | 2.304111 | 1 |
| [Ana Sofia R Alves 2019](https://pubmed.ncbi.nlm.nih.gov/?term=Alves+ASR&cauthor_id=31778458) | HICT-AT VS SOC | 0.6683934 | -1.658127 | 2.994914 | 1 |
| [Ana Sofia R Alves 2019](https://pubmed.ncbi.nlm.nih.gov/?term=Alves+ASR&cauthor_id=31778458) | HIIT VS SOC | 1.1774642 | 0.0010219 | 2.353907 | 1 |
| [Ana Sofia R Alves 2019](https://pubmed.ncbi.nlm.nih.gov/?term=Alves+ASR&cauthor_id=31778458) | HIIT-AT VS SOC | 1.2216744 | -1.200121 | 3.64347 | 1 |
| [Ana Sofia R Alves 2019](https://pubmed.ncbi.nlm.nih.gov/?term=Alves+ASR&cauthor_id=31778458) | HIIT-MICT VS SOC | 2.887594 | 0.3605999 | 5.414588 | 1 |
| [Ana Sofia R Alves 2019](https://pubmed.ncbi.nlm.nih.gov/?term=Alves+ASR&cauthor_id=31778458) | LICT VS SOC | 0.46453228 | -1.079692 | 2.008757 | 1 |
| [Ana Sofia R Alves 2019](https://pubmed.ncbi.nlm.nih.gov/?term=Alves+ASR&cauthor_id=31778458) | MICT VS SOC | 0.31117151 | -1.3539 | 1.976243 | 1 |
| [Ana Sofia R Alves 2019](https://pubmed.ncbi.nlm.nih.gov/?term=Alves+ASR&cauthor_id=31778458) | MICT-AT VS SOC | 1.0140082 | -0.2620796 | 2.290096 | 1 |
| [Ana Sofia R Alves 2019](https://pubmed.ncbi.nlm.nih.gov/?term=Alves+ASR&cauthor_id=31778458) | MIIT VS SOC | 0.20212994 | -1.893752 | 2.298012 | 1 |
| [Hyun-Wook Chae 2010](https://pubmed.ncbi.nlm.nih.gov/?term=Chae+HW&cauthor_id=21158218) | HICT-AT VS SOC | 1.1569406 | 0.0718793 | 2.242002 | 1 |
| [Hyun-Wook Chae 2010](https://pubmed.ncbi.nlm.nih.gov/?term=Chae+HW&cauthor_id=21158218) | HIIT VS SOC | 1.2216744 | -1.014742 | 3.458091 | 1 |
| [Hyun-Wook Chae 2010](https://pubmed.ncbi.nlm.nih.gov/?term=Chae+HW&cauthor_id=21158218) | HIIT-AT VS SOC | 2.887594 | 0.5376648 | 5.237523 | 1 |
| [Hyun-Wook Chae 2010](https://pubmed.ncbi.nlm.nih.gov/?term=Chae+HW&cauthor_id=21158218) | HIIT-MICT VS SOC | 0.42028173 | -0.7627413 | 1.603305 | 1 |
| [Hyun-Wook Chae 2010](https://pubmed.ncbi.nlm.nih.gov/?term=Chae+HW&cauthor_id=21158218) | LICT VS SOC | 0.40305133 | -1.566101 | 2.372203 | 1 |
| [Hyun-Wook Chae 2010](https://pubmed.ncbi.nlm.nih.gov/?term=Chae+HW&cauthor_id=21158218) | MICT VS SOC | 0.31339826 | -1.216336 | 1.843133 | 1 |
| [Hyun-Wook Chae 2010](https://pubmed.ncbi.nlm.nih.gov/?term=Chae+HW&cauthor_id=21158218) | MICT-AT VS SOC | 0.99750194 | -0.1655155 | 2.160519 | 1 |
| [Hyun-Wook Chae 2010](https://pubmed.ncbi.nlm.nih.gov/?term=Chae+HW&cauthor_id=21158218) | MIIT VS SOC | 0.19145117 | -1.730385 | 2.113287 | 1 |
| [Meng Cao 2022](https://pubmed.ncbi.nlm.nih.gov/?term=Cao+M&cauthor_id=36143083) | HICT-AT VS SOC | 0.6683934 | -1.651836 | 2.988623 | 1 |
| [Meng Cao 2022](https://pubmed.ncbi.nlm.nih.gov/?term=Cao+M&cauthor_id=36143083) | HIIT VS SOC | 1.2749942 | -0.0829042 | 2.632893 | 1 |
| [Meng Cao 2022](https://pubmed.ncbi.nlm.nih.gov/?term=Cao+M&cauthor_id=36143083) | HIIT-AT VS SOC | 1.2216744 | -1.194078 | 3.637426 | 1 |
| [Meng Cao 2022](https://pubmed.ncbi.nlm.nih.gov/?term=Cao+M&cauthor_id=36143083) | HIIT-MICT VS SOC | 2.887594 | 0.3663907 | 5.408797 | 1 |
| [Meng Cao 2022](https://pubmed.ncbi.nlm.nih.gov/?term=Cao+M&cauthor_id=36143083) | LICT VS SOC | 0.42835696 | -0.8607481 | 1.717462 | 1 |
| [Meng Cao 2022](https://pubmed.ncbi.nlm.nih.gov/?term=Cao+M&cauthor_id=36143083) | MICT VS SOC | 0.40710127 | -1.730381 | 2.544583 | 1 |
| [Meng Cao 2022](https://pubmed.ncbi.nlm.nih.gov/?term=Cao+M&cauthor_id=36143083) | MICT-AT VS SOC | 0.3112355 | -1.349435 | 1.971906 | 1 |
| [Meng Cao 2022](https://pubmed.ncbi.nlm.nih.gov/?term=Cao+M&cauthor_id=36143083) | MIIT VS SOC | 1.0386451 | -0.2436675 | 2.320958 | 1 |
| [Meng Cao 2022](https://pubmed.ncbi.nlm.nih.gov/?term=Cao+M&cauthor_id=36143083) | MIIT-AT VS SOC | 0.25072005 | -1.867087 | 2.368527 | 1 |
| [Nathalie J Farpour-Lambert 2009](https://pubmed.ncbi.nlm.nih.gov/?term=Farpour-Lambert+NJ&cauthor_id=20082930) | HICT-AT VS SOC | 0.6683934 | -1.623434 | 2.960221 | 1 |
| [Nathalie J Farpour-Lambert 2009](https://pubmed.ncbi.nlm.nih.gov/?term=Farpour-Lambert+NJ&cauthor_id=20082930) | HIIT VS SOC | 1.1720881 | 0.0126832 | 2.331493 | 1 |
| [Nathalie J Farpour-Lambert 2009](https://pubmed.ncbi.nlm.nih.gov/?term=Farpour-Lambert+NJ&cauthor_id=20082930) | HIIT-AT VS SOC | 1.2216744 | -1.166811 | 3.61016 | 1 |
| [Nathalie J Farpour-Lambert 2009](https://pubmed.ncbi.nlm.nih.gov/?term=Farpour-Lambert+NJ&cauthor_id=20082930) | HIIT-MICT VS SOC | 2.887594 | 0.3925043 | 5.382684 | 1 |
| [Nathalie J Farpour-Lambert 2009](https://pubmed.ncbi.nlm.nih.gov/?term=Farpour-Lambert+NJ&cauthor_id=20082930) | LICT VS SOC | 0.42134463 | -0.8509707 | 1.69366 | 1 |
| [Nathalie J Farpour-Lambert 2009](https://pubmed.ncbi.nlm.nih.gov/?term=Farpour-Lambert+NJ&cauthor_id=20082930) | MICT VS SOC | 0.40360106 | -1.708232 | 2.515434 | 1 |
| [Nathalie J Farpour-Lambert 2009](https://pubmed.ncbi.nlm.nih.gov/?term=Farpour-Lambert+NJ&cauthor_id=20082930) | MICT-AT VS SOC | -0.03648132 | -2.401121 | 2.328158 | 1 |
| [Nathalie J Farpour-Lambert 2009](https://pubmed.ncbi.nlm.nih.gov/?term=Farpour-Lambert+NJ&cauthor_id=20082930) | MIIT VS SOC | 1.0042403 | -0.2426509 | 2.251132 | 1 |
| [Nathalie J Farpour-Lambert 2009](https://pubmed.ncbi.nlm.nih.gov/?term=Farpour-Lambert+NJ&cauthor_id=20082930) | MIIT-AT VS SOC | 0.1993775 | -1.865234 | 2.263989 | 1 |
| [Yun Hee Lee 2010](https://pubmed.ncbi.nlm.nih.gov/?term=Lee+YH&cauthor_id=20421958) | HICT-AT VS SOC | 0.6683934 | -1.232699 | 2.569485 | 1 |
| [Yun Hee Lee 2010](https://pubmed.ncbi.nlm.nih.gov/?term=Lee+YH&cauthor_id=20421958) | HIIT VS SOC | 1.6215698 | 0.4863129 | 2.756827 | 1 |
| [Yun Hee Lee 2010](https://pubmed.ncbi.nlm.nih.gov/?term=Lee+YH&cauthor_id=20421958) | HIIT-AT VS SOC | 1.2216744 | -0.7948928 | 3.238242 | 1 |
| [Yun Hee Lee 2010](https://pubmed.ncbi.nlm.nih.gov/?term=Lee+YH&cauthor_id=20421958) | HIIT-MICT VS SOC | 2.887594 | 0.7458304 | 5.029357 | 1 |
| [Yun Hee Lee 2010](https://pubmed.ncbi.nlm.nih.gov/?term=Lee+YH&cauthor_id=20421958) | LICT VS SOC | 0.45166636 | -0.6016661 | 1.504999 | 1 |
| [Yun Hee Lee 2010](https://pubmed.ncbi.nlm.nih.gov/?term=Lee+YH&cauthor_id=20421958) | MICT VS SOC | 0.41864619 | -1.343117 | 2.18041 | 1 |
| [Yun Hee Lee 2010](https://pubmed.ncbi.nlm.nih.gov/?term=Lee+YH&cauthor_id=20421958) | MICT-AT VS SOC | 0.31696474 | -1.051152 | 1.685081 | 1 |
| [Yun Hee Lee 2010](https://pubmed.ncbi.nlm.nih.gov/?term=Lee+YH&cauthor_id=20421958) | MIIT VS SOC | 1.1454248 | 0.0863622 | 2.204487 | 1 |
| [Hee-Tae Roh 2020](https://pubmed.ncbi.nlm.nih.gov/?term=Roh+HT&cauthor_id=32268592) | HICT-AT VS SOC | 0.6683934 | -1.623434 | 2.960221 | 1 |
| [Hee-Tae Roh 2020](https://pubmed.ncbi.nlm.nih.gov/?term=Roh+HT&cauthor_id=32268592) | HIIT VS SOC | 1.1720881 | 0.0126832 | 2.331493 | 1 |
| [Hee-Tae Roh 2020](https://pubmed.ncbi.nlm.nih.gov/?term=Roh+HT&cauthor_id=32268592) | HIIT-AT VS SOC | 1.2216744 | -1.166812 | 3.61016 | 1 |
| [Hee-Tae Roh 2020](https://pubmed.ncbi.nlm.nih.gov/?term=Roh+HT&cauthor_id=32268592) | HIIT-MICT VS SOC | 2.887594 | 0.3925043 | 5.382684 | 1 |
| [Hee-Tae Roh 2020](https://pubmed.ncbi.nlm.nih.gov/?term=Roh+HT&cauthor_id=32268592) | LICT VS SOC | 0.42134463 | -0.8509707 | 1.69366 | 1 |
| [Hee-Tae Roh 2020](https://pubmed.ncbi.nlm.nih.gov/?term=Roh+HT&cauthor_id=32268592) | MICT VS SOC | 0.40360106 | -1.708232 | 2.515434 | 1 |
| [Hee-Tae Roh 2020](https://pubmed.ncbi.nlm.nih.gov/?term=Roh+HT&cauthor_id=32268592) | MICT-AT VS SOC | 0.63464582 | -1.64384 | 2.913132 | 1 |
| [Hee-Tae Roh 2020](https://pubmed.ncbi.nlm.nih.gov/?term=Roh+HT&cauthor_id=32268592) | MIIT VS SOC | 1.0042403 | -0.2426509 | 2.251132 | 1 |
| [Hee-Tae Roh 2020](https://pubmed.ncbi.nlm.nih.gov/?term=Roh+HT&cauthor_id=32268592) | MIIT-AT VS SOC | 0.1993775 | -1.865234 | 2.263989 | 1 |
| [Yuhang Gao 2025](https://pubmed.ncbi.nlm.nih.gov/?term=Gao+Y&cauthor_id=41006526) | HICT-AT VS SOC | 0.6683934 | -1.464484 | 2.801271 | 1 |
| [Yuhang Gao 2025](https://pubmed.ncbi.nlm.nih.gov/?term=Gao+Y&cauthor_id=41006526) | HIIT VS SOC | 1.1569406 | 0.0718793 | 2.242002 | 1 |
| [Yuhang Gao 2025](https://pubmed.ncbi.nlm.nih.gov/?term=Gao+Y&cauthor_id=41006526) | HIIT-AT VS SOC | 0.42028173 | -0.7627413 | 1.603305 | 1 |
| [Yuhang Gao 2025](https://pubmed.ncbi.nlm.nih.gov/?term=Gao+Y&cauthor_id=41006526) | HIIT-MICT VS SOC | 0.40305133 | -1.566101 | 2.372203 | 1 |
| [Yuhang Gao 2025](https://pubmed.ncbi.nlm.nih.gov/?term=Gao+Y&cauthor_id=41006526) | LICT VS SOC | 0.31339826 | -1.216336 | 1.843133 | 1 |
| [Yuhang Gao 2025](https://pubmed.ncbi.nlm.nih.gov/?term=Gao+Y&cauthor_id=41006526) | MICT VS SOC | 0.99750194 | -0.1655155 | 2.160519 | 1 |
| [Yuhang Gao 2025](https://pubmed.ncbi.nlm.nih.gov/?term=Gao+Y&cauthor_id=41006526) | MICT-AT VS SOC | 0.19145117 | -1.730385 | 2.113287 | 1 |
| Shitong Shao 2025 | HICT-AT VS SOC | 0.6683934 | -1.89117 | 3.227957 | 1 |
| Shitong Shao 2025 | HIIT VS SOC | 1.2342467 | -0.0852296 | 2.553723 | 1 |
| Shitong Shao 2025 | HIIT-AT VS SOC | 1.2216744 | -1.424787 | 3.868136 | 1 |
| Shitong Shao 2025 | HIIT-MICT VS SOC | 2.887594 | 0.1445361 | 5.630652 | 1 |
| Shitong Shao 2025 | LICT VS SOC | 0.36481772 | -1.446981 | 2.176617 | 1 |
| Shitong Shao 2025 | MICT VS SOC | 0.37542392 | -2.042964 | 2.793812 | 1 |
| Shitong Shao 2025 | MICT-AT VS SOC | 0.30911398 | -1.5191 | 2.137328 | 1 |
| Shitong Shao 2025 | MIIT VS SOC | 1.1599733 | -0.5790994 | 2.899046 | 1 |
| Shitong Shao 2025 | MIIT-AT VS SOC | 0.23086313 | -2.079079 | 2.540806 | 1 |
| Noelia González-Gálvez 2024 | HICT-AT VS SOC | 0.6683934 | -1.516814 | 2.853601 | 1 |
| Noelia González-Gálvez 2024 | HIIT VS SOC | 1.3659894 | 0.0803598 | 2.651619 | 1 |
| Noelia González-Gálvez 2024 | HIIT-AT VS SOC | 1.2216744 | -1.064703 | 3.508052 | 1 |
| Noelia González-Gálvez 2024 | HIIT-MICT VS SOC | 2.887594 | 0.490068 | 5.28512 | 1 |
| Noelia González-Gálvez 2024 | LICT VS SOC | 0.32225991 | -0.9002366 | 1.544756 | 1 |
| Noelia González-Gálvez 2024 | MICT VS SOC | 0.35419025 | -1.663415 | 2.371795 | 1 |
| Noelia González-Gálvez 2024 | MICT-AT VS SOC | 0.31273998 | -1.253565 | 1.879045 | 1 |
| Noelia González-Gálvez 2024 | MIIT VS SOC | 0.5222225 | -0.8897717 | 1.934217 | 1 |
| Noelia González-Gálvez 2024 | MIIT-AT VS SOC | 0.29571851 | -1.699607 | 2.291044 | 1 |
| [Ghazi Racil 2024](https://www.tandfonline.com/author/Racil,+Ghazi) | HICT-AT VS SOC | 0.6683934 | -0.3000827 | 1.63687 | 1 |
| [Ghazi Racil 2024](https://www.tandfonline.com/author/Racil,+Ghazi) | HIIT VS SOC | 0.35961674 | -0.2150157 | 0.9342492 | 1 |
| [Ghazi Racil 2024](https://www.tandfonline.com/author/Racil,+Ghazi) | HIIT-AT VS SOC | 1.2216744 | 0.0425482 | 2.4008 | 1 |
| [Ghazi Racil 2024](https://www.tandfonline.com/author/Racil,+Ghazi) | HIIT-MICT VS SOC | 2.887594 | 1.505224 | 4.269964 | 1 |
| [Ghazi Racil 2024](https://www.tandfonline.com/author/Racil,+Ghazi) | LICT VS SOC | 0.45306058 | -0.0722486 | 0.9783698 | 1 |
| [Ghazi Racil 2024](https://www.tandfonline.com/author/Racil,+Ghazi) | MICT VS SOC | 0.41872034 | -0.5239079 | 1.361349 | 1 |
| [Ghazi Racil 2024](https://www.tandfonline.com/author/Racil,+Ghazi) | MICT-AT VS SOC | 0.36140115 | -0.3629424 | 1.085745 | 1 |
| [Ghazi Racil 2024](https://www.tandfonline.com/author/Racil,+Ghazi) | MIIT VS SOC | 1.0977476 | 0.4347535 | 1.760742 | 1 |
| [Ghazi Racil 2024](https://www.tandfonline.com/author/Racil,+Ghazi) | MIIT-AT VS SOC | -0.21042209 | -1.089219 | 0.6683747 | 1 |

**Table S20** Sensitivity Analysis Using HR_max_ as the Outcome Measure.

| **dropped_id** | **comparison** | **eff** | **lci** | **uci** | **connected** |
| --- | --- | --- | --- | --- | --- |
| [Wissal Abassi 2020](https://pubmed.ncbi.nlm.nih.gov/?term=Abassi+W&cauthor_id=33581014) | HIIT VS SOC | -0.10967283 | -0.6941245 | 0.4747789 | 1 |
| [Wissal Abassi 2020](https://pubmed.ncbi.nlm.nih.gov/?term=Abassi+W&cauthor_id=33581014) | LICT VS SOC | -0.42043572 | -1.261915 | 0.4210433 | 1 |
| [Wissal Abassi 2020](https://pubmed.ncbi.nlm.nih.gov/?term=Abassi+W&cauthor_id=33581014) | MICT VS SOC | 0.02128028 | -1.10397 | 1.14653 | 1 |
| [Wissal Abassi 2020](https://pubmed.ncbi.nlm.nih.gov/?term=Abassi+W&cauthor_id=33581014) | MIIT VS SOC | 0.00909305 | -0.611252 | 0.6294381 | 1 |
| [Wissal Abassi 2022](https://pubmed.ncbi.nlm.nih.gov/?term=Abassi+W&cauthor_id=34749418) | HIIT VS SOC | 0.01253299 | -0.5341243 | 0.5591903 | 1 |
| [Wissal Abassi 2022](https://pubmed.ncbi.nlm.nih.gov/?term=Abassi+W&cauthor_id=34749418) | LICT VS SOC | -0.38226522 | -1.157694 | 0.3931639 | 1 |
| [Wissal Abassi 2022](https://pubmed.ncbi.nlm.nih.gov/?term=Abassi+W&cauthor_id=34749418) | MICT VS SOC | 0.24216524 | -1.359529 | 1.843859 | 1 |
| [Wissal Abassi 2022](https://pubmed.ncbi.nlm.nih.gov/?term=Abassi+W&cauthor_id=34749418) | MIIT VS SOC | 0.04648442 | -0.4934703 | 0.5864392 | 1 |
| [N Cvetković 2018](https://pubmed.ncbi.nlm.nih.gov/?term=Cvetkovi%C4%87+N&cauthor_id=29979479) | HIIT VS SOC | -0.13110383 | -0.7249299 | 0.4627223 | 1 |
| [N Cvetković 2018](https://pubmed.ncbi.nlm.nih.gov/?term=Cvetkovi%C4%87+N&cauthor_id=29979479) | LICT VS SOC | -0.29177643 | -1.317099 | 0.7335466 | 1 |
| [N Cvetković 2018](https://pubmed.ncbi.nlm.nih.gov/?term=Cvetkovi%C4%87+N&cauthor_id=29979479) | MICT VS SOC | 0.00605603 | -1.121979 | 1.134091 | 1 |
| [N Cvetković 2018](https://pubmed.ncbi.nlm.nih.gov/?term=Cvetkovi%C4%87+N&cauthor_id=29979479) | MIIT VS SOC | 0.00453449 | -0.5825781 | 0.591647 | 1 |
| [Meng Cao 2022](https://pubmed.ncbi.nlm.nih.gov/?term=Cao+M&cauthor_id=36143083) | HIIT VS SOC | -0.12603485 | -0.7092692 | 0.4571995 | 1 |
| [Meng Cao 2022](https://pubmed.ncbi.nlm.nih.gov/?term=Cao+M&cauthor_id=36143083) | LICT VS SOC | -0.42429508 | -1.250505 | 0.4019145 | 1 |
| [Meng Cao 2022](https://pubmed.ncbi.nlm.nih.gov/?term=Cao+M&cauthor_id=36143083) | MICT VS SOC | 0.00970197 | -1.098656 | 1.11806 | 1 |
| [Meng Cao 2022](https://pubmed.ncbi.nlm.nih.gov/?term=Cao+M&cauthor_id=36143083) | MIIT VS SOC | -0.00595793 | -0.5823162 | 0.5704003 | 1 |
| [Ragab K Elnaggar 2021](https://pubmed.ncbi.nlm.nih.gov/?term=Elnaggar+RK&cauthor_id=33761464) | HIIT VS SOC | -0.21373404 | -0.7255986 | 0.2981305 | 1 |
| [Ragab K Elnaggar 2021](https://pubmed.ncbi.nlm.nih.gov/?term=Elnaggar+RK&cauthor_id=33761464) | LICT VS SOC | -0.46855828 | -1.23965 | 0.3025337 | 1 |
| [Ragab K Elnaggar 2021](https://pubmed.ncbi.nlm.nih.gov/?term=Elnaggar+RK&cauthor_id=33761464) | MICT VS SOC | -0.05234553 | -1.080469 | 0.9757783 | 1 |
| [Ragab K Elnaggar 2021](https://pubmed.ncbi.nlm.nih.gov/?term=Elnaggar+RK&cauthor_id=33761464) | MIIT VS SOC | -0.18372525 | -0.7537507 | 0.3863001 | 1 |
| [Danielle Lambrick 2016](https://pubmed.ncbi.nlm.nih.gov/?term=Lambrick+D&cauthor_id=26009003) | HIIT VS SOC | -0.18601977 | -0.7679971 | 0.3959576 | 1 |
| [Danielle Lambrick 2016](https://pubmed.ncbi.nlm.nih.gov/?term=Lambrick+D&cauthor_id=26009003) | LICT VS SOC | -0.43942503 | -1.267723 | 0.3888727 | 1 |
| [Danielle Lambrick 2016](https://pubmed.ncbi.nlm.nih.gov/?term=Lambrick+D&cauthor_id=26009003) | MICT VS SOC | -0.03290808 | -1.143442 | 1.077626 | 1 |
| [Danielle Lambrick 2016](https://pubmed.ncbi.nlm.nih.gov/?term=Lambrick+D&cauthor_id=26009003) | MIIT VS SOC | -0.02875067 | -0.6063088 | 0.5488074 | 1 |
| [D M Prado 2010](https://pubmed.ncbi.nlm.nih.gov/?term=Prado+DM&cauthor_id=21072735) | HIIT VS SOC | -0.1142559 | -0.6406499 | 0.4121381 | 1 |
| [D M Prado 2010](https://pubmed.ncbi.nlm.nih.gov/?term=Prado+DM&cauthor_id=21072735) | LICT VS SOC | -0.39189064 | -1.185159 | 0.4013773 | 1 |
| [D M Prado 2010](https://pubmed.ncbi.nlm.nih.gov/?term=Prado+DM&cauthor_id=21072735) | MICT VS SOC | 0.01817319 | -1.037815 | 1.074161 | 1 |
| [D M Prado 2010](https://pubmed.ncbi.nlm.nih.gov/?term=Prado+DM&cauthor_id=21072735) | MIIT VS SOC | 0.11151931 | -0.4780451 | 0.7010837 | 1 |
| [Fabrício Vasconcellos 2016](https://pubmed.ncbi.nlm.nih.gov/?term=Vasconcellos+F&cauthor_id=26208409) | HIIT VS SOC | -0.14051177 | -0.6738356 | 0.392812 | 1 |
| [Fabrício Vasconcellos 2016](https://pubmed.ncbi.nlm.nih.gov/?term=Vasconcellos+F&cauthor_id=26208409) | LICT VS SOC | -0.24185072 | -1.172234 | 0.688533 | 1 |
| [Fabrício Vasconcellos 2016](https://pubmed.ncbi.nlm.nih.gov/?term=Vasconcellos+F&cauthor_id=26208409) | MICT VS SOC | -0.00052051 | -1.075959 | 1.074918 | 1 |
| [Fabrício Vasconcellos 2016](https://pubmed.ncbi.nlm.nih.gov/?term=Vasconcellos+F&cauthor_id=26208409) | MIIT VS SOC | 0.00532063 | -0.5558658 | 0.566507 | 1 |
| [Ragab K Elnaggar 2024](https://pubmed.ncbi.nlm.nih.gov/?term=Elnaggar+RK&cauthor_id=39612435) | HIIT VS SOC | -0.44089133 | -0.8597294 | -0.0220533 | 1 |
| [Ragab K Elnaggar 2024](https://pubmed.ncbi.nlm.nih.gov/?term=Elnaggar+RK&cauthor_id=39612435) | LICT VS SOC | -0.43866692 | -1.029115 | 0.1517815 | 1 |
| [Ragab K Elnaggar 2024](https://pubmed.ncbi.nlm.nih.gov/?term=Elnaggar+RK&cauthor_id=39612435) | MICT VS SOC | -0.21158794 | -1.012927 | 0.5897511 | 1 |
| [Ragab K Elnaggar 2024](https://pubmed.ncbi.nlm.nih.gov/?term=Elnaggar+RK&cauthor_id=39612435) | MIIT VS SOC | -0.11800813 | -0.5573262 | 0.3213099 | 1 |
| [Chongwen Zuo 2023](https://pubmed.ncbi.nlm.nih.gov/?term=Zuo+C&cauthor_id=37946224) | HIIT VS SOC | -0.14812384 | -0.7094647 | 0.413217 | 1 |
| [Chongwen Zuo 2023](https://pubmed.ncbi.nlm.nih.gov/?term=Zuo+C&cauthor_id=37946224) | LICT VS SOC | -0.43495676 | -1.262152 | 0.3922389 | 1 |
| [Chongwen Zuo 2023](https://pubmed.ncbi.nlm.nih.gov/?term=Zuo+C&cauthor_id=37946224) | MICT VS SOC | -0.00599474 | -1.111687 | 1.099698 | 1 |
| [Chongwen Zuo 2023](https://pubmed.ncbi.nlm.nih.gov/?term=Zuo+C&cauthor_id=37946224) | MIIT VS SOC | -0.03703061 | -0.6314356 | 0.5573744 | 1 |
| Shitong Shao 2025 | HIIT VS SOC | -0.18467826 | -0.7316777 | 0.3623211 | 1 |
| Shitong Shao 2025 | LICT VS SOC | -0.83047647 | -1.896709 | 0.2357556 | 1 |
| Shitong Shao 2025 | MICT VS SOC | -0.03193356 | -1.126724 | 1.062857 | 1 |
| Shitong Shao 2025 | MIIT VS SOC | -0.00984967 | -0.6370326 | 0.6173333 | 1 |
| Mattia D’Alleva 2023 | HIIT VS SOC | -0.15769388 | -0.7054418 | 0.390054 | 1 |
| Mattia D’Alleva 2023 | LICT VS SOC | -0.43222777 | -1.257658 | 0.3932021 | 1 |
| Mattia D’Alleva 2023 | MICT VS SOC | -0.0728319 | -1.446102 | 1.300438 | 1 |
| Mattia D’Alleva 2023 | MIIT VS SOC | -0.01798917 | -0.5898036 | 0.5538252 | 1 |
| [Ghazi Racil 2024](https://www.tandfonline.com/author/Racil,+Ghazi) | HIIT VS SOC | -0.14966189 | -0.7478743 | 0.4485505 | 1 |
| [Ghazi Racil 2024](https://www.tandfonline.com/author/Racil,+Ghazi) | LICT VS SOC | -0.42789547 | -1.282489 | 0.4266983 | 1 |
| [Ghazi Racil 2024](https://www.tandfonline.com/author/Racil,+Ghazi) | MICT VS SOC | -0.00716463 | -1.150218 | 1.135888 | 1 |
| [Ghazi Racil 2024](https://www.tandfonline.com/author/Racil,+Ghazi) | MIIT VS SOC | 0.01333441 | -0.6219824 | 0.6486512 | 1 |

**Table S21** Sensitivity Analysis Using SBP as the Outcome Measure.

| **dropped_id** | **comparison** | **eff** | **lci** | **uci** | **connected** |
| --- | --- | --- | --- | --- | --- |
| [Andreas A Meyer 2006](https://pubmed.ncbi.nlm.nih.gov/?term=Meyer+AA&cauthor_id=17084264) | HIIT VS SOC | -0.48281247 | -1.12823 | 0.1626054 | 1 |
| [Andreas A Meyer 2006](https://pubmed.ncbi.nlm.nih.gov/?term=Meyer+AA&cauthor_id=17084264) | HIIT-AT VS SOC | -0.10073636 | -2.045372 | 1.8439 | 1 |
| [Andreas A Meyer 2006](https://pubmed.ncbi.nlm.nih.gov/?term=Meyer+AA&cauthor_id=17084264) | HIIT-MICT VS SOC | 0.72530164 | -1.229396 | 2.68 | 1 |
| [Andreas A Meyer 2006](https://pubmed.ncbi.nlm.nih.gov/?term=Meyer+AA&cauthor_id=17084264) | LICT VS SOC | -0.36903147 | -1.243795 | 0.5057318 | 1 |
| [Andreas A Meyer 2006](https://pubmed.ncbi.nlm.nih.gov/?term=Meyer+AA&cauthor_id=17084264) | LICT-NUT VS SOC | 0.3913445 | -1.255614 | 2.038303 | 1 |
| [Andreas A Meyer 2006](https://pubmed.ncbi.nlm.nih.gov/?term=Meyer+AA&cauthor_id=17084264) | MICT VS SOC | -2.452712 | -3.822566 | -1.082858 | 1 |
| [Andreas A Meyer 2006](https://pubmed.ncbi.nlm.nih.gov/?term=Meyer+AA&cauthor_id=17084264) | MICT-AT VS SOC | -0.88992419 | -2.820694 | 1.040845 | 1 |
| [Andreas A Meyer 2006](https://pubmed.ncbi.nlm.nih.gov/?term=Meyer+AA&cauthor_id=17084264) | MICT-NUT VS SOC | -0.30215435 | -1.343497 | 0.7391881 | 1 |
| [Andreas A Meyer 2006](https://pubmed.ncbi.nlm.nih.gov/?term=Meyer+AA&cauthor_id=17084264) | MIIT VS SOC | -0.80210299 | -1.525492 | -0.0787142 | 1 |
| [Andreas A Meyer 2006](https://pubmed.ncbi.nlm.nih.gov/?term=Meyer+AA&cauthor_id=17084264) | MIIT-AT VS SOC | -0.53691381 | -1.75836 | 0.6845326 | 1 |
| [Andreas A Meyer 2006](https://pubmed.ncbi.nlm.nih.gov/?term=Meyer+AA&cauthor_id=17084264) | NUT VS SOC | 0.83919481 | -1.586891 | 3.265281 | 1 |
| [María José Aguilar-Cordero 2020](https://pubmed.ncbi.nlm.nih.gov/?term=Aguilar-Cordero+MJ&cauthor_id=31678988) | HIIT VS SOC | -0.46759039 | -1.084875 | 0.1496941 | 1 |
| [María José Aguilar-Cordero 2020](https://pubmed.ncbi.nlm.nih.gov/?term=Aguilar-Cordero+MJ&cauthor_id=31678988) | HIIT-AT VS SOC | -0.10073634 | -1.982048 | 1.780575 | 1 |
| [María José Aguilar-Cordero 2020](https://pubmed.ncbi.nlm.nih.gov/?term=Aguilar-Cordero+MJ&cauthor_id=31678988) | HIIT-MICT VS SOC | 0.72530165 | -1.166408 | 2.617012 | 1 |
| [María José Aguilar-Cordero 2020](https://pubmed.ncbi.nlm.nih.gov/?term=Aguilar-Cordero+MJ&cauthor_id=31678988) | LICT VS SOC | -0.35943782 | -1.201143 | 0.4822674 | 1 |
| [María José Aguilar-Cordero 2020](https://pubmed.ncbi.nlm.nih.gov/?term=Aguilar-Cordero+MJ&cauthor_id=31678988) | LICT-NUT VS SOC | 0.39181534 | -1.192658 | 1.976288 | 1 |
| [María José Aguilar-Cordero 2020](https://pubmed.ncbi.nlm.nih.gov/?term=Aguilar-Cordero+MJ&cauthor_id=31678988) | MICT VS SOC | -2.4275943 | -3.757808 | -1.09738 | 1 |
| [María José Aguilar-Cordero 2020](https://pubmed.ncbi.nlm.nih.gov/?term=Aguilar-Cordero+MJ&cauthor_id=31678988) | MICT-AT VS SOC | -0.88992419 | -2.756899 | 0.9770503 | 1 |
| [María José Aguilar-Cordero 2020](https://pubmed.ncbi.nlm.nih.gov/?term=Aguilar-Cordero+MJ&cauthor_id=31678988) | MICT-NUT VS SOC | -0.30114698 | -1.302904 | 0.70061 | 1 |
| [María José Aguilar-Cordero 2020](https://pubmed.ncbi.nlm.nih.gov/?term=Aguilar-Cordero+MJ&cauthor_id=31678988) | MIIT VS SOC | -0.75485731 | -1.402608 | -0.1071067 | 1 |
| [María José Aguilar-Cordero 2020](https://pubmed.ncbi.nlm.nih.gov/?term=Aguilar-Cordero+MJ&cauthor_id=31678988) | MIIT-AT VS SOC | -0.53260691 | -1.709554 | 0.6443406 | 1 |
| [Thaynã Alves Bezerra 2022](https://pubmed.ncbi.nlm.nih.gov/?term=Bezerra+TA&cauthor_id=35699999) | HIIT VS SOC | -0.4680722 | -1.100021 | 0.1638771 | 1 |
| [Thaynã Alves Bezerra 2022](https://pubmed.ncbi.nlm.nih.gov/?term=Bezerra+TA&cauthor_id=35699999) | HIIT-AT VS SOC | -0.10073628 | -2.024625 | 1.823152 | 1 |
| [Thaynã Alves Bezerra 2022](https://pubmed.ncbi.nlm.nih.gov/?term=Bezerra+TA&cauthor_id=35699999) | HIIT-MICT VS SOC | 0.72530171 | -1.208757 | 2.65936 | 1 |
| [Thaynã Alves Bezerra 2022](https://pubmed.ncbi.nlm.nih.gov/?term=Bezerra+TA&cauthor_id=35699999) | LICT VS SOC | -0.36019876 | -1.223078 | 0.502681 | 1 |
| [Thaynã Alves Bezerra 2022](https://pubmed.ncbi.nlm.nih.gov/?term=Bezerra+TA&cauthor_id=35699999) | LICT-NUT VS SOC | 0.4986882 | -1.166077 | 2.163454 | 1 |
| [Thaynã Alves Bezerra 2022](https://pubmed.ncbi.nlm.nih.gov/?term=Bezerra+TA&cauthor_id=35699999) | MICT VS SOC | -2.4414842 | -3.7986 | -1.084368 | 1 |
| [Thaynã Alves Bezerra 2022](https://pubmed.ncbi.nlm.nih.gov/?term=Bezerra+TA&cauthor_id=35699999) | MICT-AT VS SOC | -0.88992417 | -2.799795 | 1.019947 | 1 |
| [Thaynã Alves Bezerra 2022](https://pubmed.ncbi.nlm.nih.gov/?term=Bezerra+TA&cauthor_id=35699999) | MICT-NUT VS SOC | -0.09021739 | -1.33445 | 1.154015 | 1 |
| [Thaynã Alves Bezerra 2022](https://pubmed.ncbi.nlm.nih.gov/?term=Bezerra+TA&cauthor_id=35699999) | MIIT VS SOC | -0.75757234 | -1.421317 | -0.0938273 | 1 |
| [Thaynã Alves Bezerra 2022](https://pubmed.ncbi.nlm.nih.gov/?term=Bezerra+TA&cauthor_id=35699999) | MIIT-AT VS SOC | -0.53274967 | -1.739456 | 0.6739571 | 1 |
| [Thaynã Alves Bezerra 2022](https://pubmed.ncbi.nlm.nih.gov/?term=Bezerra+TA&cauthor_id=35699999) | NUT VS SOC | 0.94653818 | -1.475156 | 3.368232 | 1 |
| [N Cvetković 2018](https://pubmed.ncbi.nlm.nih.gov/?term=Cvetkovi%C4%87+N&cauthor_id=29979479) | HIIT VS SOC | -0.44505298 | -1.152801 | 0.262695 | 1 |
| [N Cvetković 2018](https://pubmed.ncbi.nlm.nih.gov/?term=Cvetkovi%C4%87+N&cauthor_id=29979479) | HIIT-AT VS SOC | -0.10073634 | -2.106451 | 1.904979 | 1 |
| [N Cvetković 2018](https://pubmed.ncbi.nlm.nih.gov/?term=Cvetkovi%C4%87+N&cauthor_id=29979479) | HIIT-MICT VS SOC | 0.72530165 | -1.29017 | 2.740773 | 1 |
| [N Cvetković 2018](https://pubmed.ncbi.nlm.nih.gov/?term=Cvetkovi%C4%87+N&cauthor_id=29979479) | LICT VS SOC | -0.39526159 | -1.443216 | 0.6526927 | 1 |
| [N Cvetković 2018](https://pubmed.ncbi.nlm.nih.gov/?term=Cvetkovi%C4%87+N&cauthor_id=29979479) | LICT-NUT VS SOC | 0.39094429 | -1.315974 | 2.097863 | 1 |
| [N Cvetković 2018](https://pubmed.ncbi.nlm.nih.gov/?term=Cvetkovi%C4%87+N&cauthor_id=29979479) | MICT VS SOC | -2.4584889 | -3.868768 | -1.04821 | 1 |
| [N Cvetković 2018](https://pubmed.ncbi.nlm.nih.gov/?term=Cvetkovi%C4%87+N&cauthor_id=29979479) | MICT-AT VS SOC | -0.88992419 | -2.882198 | 1.102349 | 1 |
| [N Cvetković 2018](https://pubmed.ncbi.nlm.nih.gov/?term=Cvetkovi%C4%87+N&cauthor_id=29979479) | MICT-NUT VS SOC | -0.303019 | -1.382336 | 0.7762981 | 1 |
| [N Cvetković 2018](https://pubmed.ncbi.nlm.nih.gov/?term=Cvetkovi%C4%87+N&cauthor_id=29979479) | MIIT VS SOC | -0.75656124 | -1.456273 | -0.0568498 | 1 |
| [N Cvetković 2018](https://pubmed.ncbi.nlm.nih.gov/?term=Cvetkovi%C4%87+N&cauthor_id=29979479) | MIIT-AT VS SOC | -0.52625876 | -1.79201 | 0.7394925 | 1 |
| [N Cvetković 2018](https://pubmed.ncbi.nlm.nih.gov/?term=Cvetkovi%C4%87+N&cauthor_id=29979479) | NUT VS SOC | 0.83879442 | -1.676811 | 3.354399 | 1 |
| [Catherine L Davis 2020](https://pubmed.ncbi.nlm.nih.gov/?term=Davis+CL&cauthor_id=31754238) | HIIT VS SOC | -0.47679065 | -1.117287 | 0.1637052 | 1 |
| [Catherine L Davis 2020](https://pubmed.ncbi.nlm.nih.gov/?term=Davis+CL&cauthor_id=31754238) | HIIT-AT VS SOC | -0.10073637 | -2.043901 | 1.842428 | 1 |
| [Catherine L Davis 2020](https://pubmed.ncbi.nlm.nih.gov/?term=Davis+CL&cauthor_id=31754238) | HIIT-MICT VS SOC | 0.72530162 | -1.227932 | 2.678535 | 1 |
| [Catherine L Davis 2020](https://pubmed.ncbi.nlm.nih.gov/?term=Davis+CL&cauthor_id=31754238) | LICT VS SOC | -0.44605355 | -1.450657 | 0.5585504 | 1 |
| [Catherine L Davis 2020](https://pubmed.ncbi.nlm.nih.gov/?term=Davis+CL&cauthor_id=31754238) | LICT-NUT VS SOC | 0.39135469 | -1.254155 | 2.036865 | 1 |
| [Catherine L Davis 2020](https://pubmed.ncbi.nlm.nih.gov/?term=Davis+CL&cauthor_id=31754238) | MICT VS SOC | -2.4503029 | -3.819049 | -1.081557 | 1 |
| [Catherine L Davis 2020](https://pubmed.ncbi.nlm.nih.gov/?term=Davis+CL&cauthor_id=31754238) | MICT-AT VS SOC | -0.8899242 | -2.819211 | 1.039363 | 1 |
| [Catherine L Davis 2020](https://pubmed.ncbi.nlm.nih.gov/?term=Davis+CL&cauthor_id=31754238) | MICT-NUT VS SOC | -0.30213233 | -1.342558 | 0.7382929 | 1 |
| [Catherine L Davis 2020](https://pubmed.ncbi.nlm.nih.gov/?term=Davis+CL&cauthor_id=31754238) | MIIT VS SOC | -0.76862758 | -1.441966 | -0.0952889 | 1 |
| [Catherine L Davis 2020](https://pubmed.ncbi.nlm.nih.gov/?term=Davis+CL&cauthor_id=31754238) | MIIT-AT VS SOC | -0.53521371 | -1.755448 | 0.685021 | 1 |
| [Catherine L Davis 2020](https://pubmed.ncbi.nlm.nih.gov/?term=Davis+CL&cauthor_id=31754238) | NUT VS SOC | 0.839205 | -1.584718 | 3.263128 | 1 |
| [Nathalie J Farpour-Lambert 2009](https://pubmed.ncbi.nlm.nih.gov/?term=Farpour-Lambert+NJ&cauthor_id=20082930) | HIIT VS SOC | -0.46527426 | -1.110436 | 0.1798871 | 1 |
| [Nathalie J Farpour-Lambert 2009](https://pubmed.ncbi.nlm.nih.gov/?term=Farpour-Lambert+NJ&cauthor_id=20082930) | HIIT-AT VS SOC | -0.10073633 | -2.047059 | 1.845587 | 1 |
| [Nathalie J Farpour-Lambert 2009](https://pubmed.ncbi.nlm.nih.gov/?term=Farpour-Lambert+NJ&cauthor_id=20082930) | HIIT-MICT VS SOC | 0.72530166 | -1.231075 | 2.681678 | 1 |
| [Nathalie J Farpour-Lambert 2009](https://pubmed.ncbi.nlm.nih.gov/?term=Farpour-Lambert+NJ&cauthor_id=20082930) | LICT VS SOC | -0.36001362 | -1.23416 | 0.5141329 | 1 |
| [Nathalie J Farpour-Lambert 2009](https://pubmed.ncbi.nlm.nih.gov/?term=Farpour-Lambert+NJ&cauthor_id=20082930) | LICT-NUT VS SOC | 0.39133285 | -1.257285 | 2.039951 | 1 |
| [Nathalie J Farpour-Lambert 2009](https://pubmed.ncbi.nlm.nih.gov/?term=Farpour-Lambert+NJ&cauthor_id=20082930) | MICT VS SOC | -2.4475233 | -3.818434 | -1.076612 | 1 |
| [Nathalie J Farpour-Lambert 2009](https://pubmed.ncbi.nlm.nih.gov/?term=Farpour-Lambert+NJ&cauthor_id=20082930) | MICT-AT VS SOC | -0.88992419 | -2.822393 | 1.042544 | 1 |
| [Nathalie J Farpour-Lambert 2009](https://pubmed.ncbi.nlm.nih.gov/?term=Farpour-Lambert+NJ&cauthor_id=20082930) | MICT-NUT | -0.30217959 | -1.344573 | 0.7402143 | 1 |
| [Nathalie J Farpour-Lambert 2009](https://pubmed.ncbi.nlm.nih.gov/?term=Farpour-Lambert+NJ&cauthor_id=20082930) | MIIT VS SOC | -0.75781411 | -1.430639 | -0.0849897 | 1 |
| [Nathalie J Farpour-Lambert 2009](https://pubmed.ncbi.nlm.nih.gov/?term=Farpour-Lambert+NJ&cauthor_id=20082930) | MIIT-AT VS SOC | -0.49347454 | -2.129065 | 1.142116 | 1 |
| [Nathalie J Farpour-Lambert 2009](https://pubmed.ncbi.nlm.nih.gov/?term=Farpour-Lambert+NJ&cauthor_id=20082930) | NUT VS SOC | 0.83918317 | -1.589381 | 3.267748 | 1 |
| [Vandana Jain 2022](https://pubmed.ncbi.nlm.nih.gov/?term=Jain+V&cauthor_id=36124513) | disconnected |  |  |  | 0 |
| [Jun Kim 2021](https://pubmed.ncbi.nlm.nih.gov/?term=Kim+J&cauthor_id=31812946) | HIIT VS SOC | -0.39104861 | -0.9528089 | 0.1707117 | 1 |
| [Jun Kim 2021](https://pubmed.ncbi.nlm.nih.gov/?term=Kim+J&cauthor_id=31812946) | HIIT-AT VS SOC | -0.10073636 | -1.807392 | 1.605919 | 1 |
| [Jun Kim 2021](https://pubmed.ncbi.nlm.nih.gov/?term=Kim+J&cauthor_id=31812946) | HIIT-MICT VS SOC | 0.72530163 | -0.9928099 | 2.443413 | 1 |
| [Jun Kim 2021](https://pubmed.ncbi.nlm.nih.gov/?term=Kim+J&cauthor_id=31812946) | LICT VS SOC | -0.31193759 | -1.067273 | 0.4433978 | 1 |
| [Jun Kim 2021](https://pubmed.ncbi.nlm.nih.gov/?term=Kim+J&cauthor_id=31812946) | LICT-NUT VS SOC | 0.39346231 | -1.016577 | 1.803502 | 1 |
| [Jun Kim 2021](https://pubmed.ncbi.nlm.nih.gov/?term=Kim+J&cauthor_id=31812946) | MICT VS SOC | -2.3371115 | -3.568985 | -1.105238 | 1 |
| [Jun Kim 2021](https://pubmed.ncbi.nlm.nih.gov/?term=Kim+J&cauthor_id=31812946) | MICT-AT VS SOC | -0.88992419 | -2.580762 | 0.8009141 | 1 |
| [Jun Kim 2021](https://pubmed.ncbi.nlm.nih.gov/?term=Kim+J&cauthor_id=31812946) | MICT-NUT VS SOC | -0.29760391 | -1.188834 | 0.5936266 | 1 |
| [Jun Kim 2021](https://pubmed.ncbi.nlm.nih.gov/?term=Kim+J&cauthor_id=31812946) | MIIT VS SOC | -0.52053963 | -1.143038 | 0.101959 | 1 |
| [Jun Kim 2021](https://pubmed.ncbi.nlm.nih.gov/?term=Kim+J&cauthor_id=31812946) | MIIT-AT VS SOC | -0.51105562 | -1.565214 | 0.5431026 | 1 |
| [Jun Kim 2021](https://pubmed.ncbi.nlm.nih.gov/?term=Kim+J&cauthor_id=31812946) | NUT VS SOC | 0.84131331 | -1.230572 | 2.913198 | 1 |
| [Yun Hee Lee 2010](https://pubmed.ncbi.nlm.nih.gov/?term=Lee+YH&cauthor_id=20421958) | HIIT VS SOC | -0.49792321 | -1.209129 | 0.2132824 | 1 |
| [Yun Hee Lee 2010](https://pubmed.ncbi.nlm.nih.gov/?term=Lee+YH&cauthor_id=20421958) | HIIT-AT VS SOC | -0.10073634 | -2.111244 | 1.909771 | 1 |
| [Yun Hee Lee 2010](https://pubmed.ncbi.nlm.nih.gov/?term=Lee+YH&cauthor_id=20421958) | HIIT-MICT VS SOC | 0.72530166 | -1.29494 | 2.745543 | 1 |
| [Yun Hee Lee 2010](https://pubmed.ncbi.nlm.nih.gov/?term=Lee+YH&cauthor_id=20421958) | LICT VS SOC | -0.36699187 | -1.274055 | 0.540071 | 1 |
| [Yun Hee Lee 2010](https://pubmed.ncbi.nlm.nih.gov/?term=Lee+YH&cauthor_id=20421958) | LICT-NUT VS SOC | 0.39091474 | -1.320696 | 2.102526 | 1 |
| [Yun Hee Lee 2010](https://pubmed.ncbi.nlm.nih.gov/?term=Lee+YH&cauthor_id=20421958) | MICT VS SOC | -2.4769304 | -3.890063 | -1.063798 | 1 |
| [Yun Hee Lee 2010](https://pubmed.ncbi.nlm.nih.gov/?term=Lee+YH&cauthor_id=20421958) | MICT-AT VS SOC | -0.88992419 | -2.887022 | 1.107174 | 1 |
| [Yun Hee Lee 2010](https://pubmed.ncbi.nlm.nih.gov/?term=Lee+YH&cauthor_id=20421958) | MICT-NUT VS SOC | -0.3030829 | -1.385372 | 0.7792058 | 1 |
| [Yun Hee Lee 2010](https://pubmed.ncbi.nlm.nih.gov/?term=Lee+YH&cauthor_id=20421958) | MIIT VS SOC | -0.77331917 | -1.476079 | -0.0705594 | 1 |
| [Yun Hee Lee 2010](https://pubmed.ncbi.nlm.nih.gov/?term=Lee+YH&cauthor_id=20421958) | MIIT-AT VS SOC | -0.58260311 | -2.490083 | 1.324877 | 1 |
| [Yun Hee Lee 2010](https://pubmed.ncbi.nlm.nih.gov/?term=Lee+YH&cauthor_id=20421958) | NUT VS SOC | 0.83876486 | -1.683845 | 3.361374 | 1 |
| [Cao Meng 2022](https://pubmed.ncbi.nlm.nih.gov/?term=Meng+C&cauthor_id=35232402) | HIIT VS SOC | -0.27188917 | -0.7315614 | 0.187783 | 1 |
| [Cao Meng 2022](https://pubmed.ncbi.nlm.nih.gov/?term=Meng+C&cauthor_id=35232402) | HIIT-AT VS SOC | -0.10073634 | -1.449852 | 1.24838 | 1 |
| [Cao Meng 2022](https://pubmed.ncbi.nlm.nih.gov/?term=Meng+C&cauthor_id=35232402) | HIIT-MICT VS SOC | 0.72530165 | -0.6382776 | 2.088881 | 1 |
| [Cao Meng 2022](https://pubmed.ncbi.nlm.nih.gov/?term=Meng+C&cauthor_id=35232402) | LICT VS SOC | -0.31007271 | -0.8777279 | 0.2575825 | 1 |
| [Cao Meng 2022](https://pubmed.ncbi.nlm.nih.gov/?term=Meng+C&cauthor_id=35232402) | LICT-NUT VS SOC | 0.40024362 | -0.6376672 | 1.438154 | 1 |
| [Cao Meng 2022](https://pubmed.ncbi.nlm.nih.gov/?term=Meng+C&cauthor_id=35232402) | MICT VS SOC | -5.5434993 | -7.293721 | -3.793277 | 1 |
| [Cao Meng 2022](https://pubmed.ncbi.nlm.nih.gov/?term=Meng+C&cauthor_id=35232402) | MICT-AT VS SOC | -0.88992419 | -2.218975 | 0.4391263 | 1 |
| [Cao Meng 2022](https://pubmed.ncbi.nlm.nih.gov/?term=Meng+C&cauthor_id=35232402) | MICT-NUT VS SOC | -0.28331616 | -0.9380636 | 0.3714313 | 1 |
| [Cao Meng 2022](https://pubmed.ncbi.nlm.nih.gov/?term=Meng+C&cauthor_id=35232402) | MIIT VS SOC | -0.63314764 | -1.079945 | -0.1863505 | 1 |
| [Cao Meng 2022](https://pubmed.ncbi.nlm.nih.gov/?term=Meng+C&cauthor_id=35232402) | MIIT-AT VS SOC | -0.47810321 | -1.274536 | 0.3183301 | 1 |
| [Cao Meng 2022](https://pubmed.ncbi.nlm.nih.gov/?term=Meng+C&cauthor_id=35232402) | NUT VS SOC | 0.8480955 | -0.6649173 | 2.361108 | 1 |
| [Won-Mok Son 2017](https://pubmed.ncbi.nlm.nih.gov/?term=Son+WM&cauthor_id=28590143) | HIIT VS SOC | -0.35218237 | -0.7859948 | 0.0816301 | 1 |
| [Won-Mok Son 2017](https://pubmed.ncbi.nlm.nih.gov/?term=Son+WM&cauthor_id=28590143) | HIIT-AT VS SOC | -0.10073634 | -1.449874 | 1.248401 | 1 |
| [Won-Mok Son 2017](https://pubmed.ncbi.nlm.nih.gov/?term=Son+WM&cauthor_id=28590143) | HIIT-MICT VS SOC | 0.72530165 | -0.638299 | 2.088902 | 1 |
| [Won-Mok Son 2017](https://pubmed.ncbi.nlm.nih.gov/?term=Son+WM&cauthor_id=28590143) | LICT VS SOC | -0.32370314 | -0.8908544 | 0.2434481 | 1 |
| [Won-Mok Son 2017](https://pubmed.ncbi.nlm.nih.gov/?term=Son+WM&cauthor_id=28590143) | LICT-NUT VS SOC | 0.40024295 | -0.6376873 | 1.438173 | 1 |
| [Won-Mok Son 2017](https://pubmed.ncbi.nlm.nih.gov/?term=Son+WM&cauthor_id=28590143) | MICT VS SOC | -0.67402057 | -1.838511 | 0.4904701 | 1 |
| [Won-Mok Son 2017](https://pubmed.ncbi.nlm.nih.gov/?term=Son+WM&cauthor_id=28590143) | MICT-AT VS SOC | -0.88992419 | -2.218997 | 0.4391483 | 1 |
| [Won-Mok Son 2017](https://pubmed.ncbi.nlm.nih.gov/?term=Son+WM&cauthor_id=28590143) | MICT-NUT VS SOC | -0.28331757 | -0.9380521 | 0.3714169 | 1 |
| [Won-Mok Son 2017](https://pubmed.ncbi.nlm.nih.gov/?term=Son+WM&cauthor_id=28590143) | MIIT VS SOC | -0.66134338 | -1.105265 | -0.217422 | 1 |
| [Won-Mok Son 2017](https://pubmed.ncbi.nlm.nih.gov/?term=Son+WM&cauthor_id=28590143) | MIIT-AT VS SOC | -0.50040377 | -1.295729 | 0.294921 | 1 |
| [Won-Mok Son 2017](https://pubmed.ncbi.nlm.nih.gov/?term=Son+WM&cauthor_id=28590143) | NUT VS SOC | 0.84809482 | -0.6649507 | 2.36114 | 1 |
| [Young-Gyun Seo 2019](https://pubmed.ncbi.nlm.nih.gov/?term=Seo+YG&cauthor_id=30634657) | HIIT VS SOC | -0.49595257 | -1.135133 | 0.1432282 | 1 |
| [Young-Gyun Seo 2019](https://pubmed.ncbi.nlm.nih.gov/?term=Seo+YG&cauthor_id=30634657) | HIIT-AT VS SOC | -0.10073634 | -2.02772 | 1.826248 | 1 |
| [Young-Gyun Seo 2019](https://pubmed.ncbi.nlm.nih.gov/?term=Seo+YG&cauthor_id=30634657) | HIIT-MICT VS SOC | 0.72530165 | -1.211836 | 2.662439 | 1 |
| [Young-Gyun Seo 2019](https://pubmed.ncbi.nlm.nih.gov/?term=Seo+YG&cauthor_id=30634657) | LICT VS SOC | -0.37653034 | -1.242495 | 0.4894348 | 1 |
| [Young-Gyun Seo 2019](https://pubmed.ncbi.nlm.nih.gov/?term=Seo+YG&cauthor_id=30634657) | LICT-NUT VS SOC | 0.39146895 | -1.238105 | 2.021043 | 1 |
| [Young-Gyun Seo 2019](https://pubmed.ncbi.nlm.nih.gov/?term=Seo+YG&cauthor_id=30634657) | MICT VS SOC | -2.4515425 | -3.810609 | -1.092476 | 1 |
| [Young-Gyun Seo 2019](https://pubmed.ncbi.nlm.nih.gov/?term=Seo+YG&cauthor_id=30634657) | MICT-AT VS SOC | -0.88992419 | -2.802914 | 1.023066 | 1 |
| [Young-Gyun Seo 2019](https://pubmed.ncbi.nlm.nih.gov/?term=Seo+YG&cauthor_id=30634657) | MICT-NUT VS SOC | -0.30188585 | -1.332218 | 0.7284459 | 1 |
| [Young-Gyun Seo 2019](https://pubmed.ncbi.nlm.nih.gov/?term=Seo+YG&cauthor_id=30634657) | MIIT VS SOC | -0.84076809 | -1.556233 | -0.125303 | 1 |
| [Young-Gyun Seo 2019](https://pubmed.ncbi.nlm.nih.gov/?term=Seo+YG&cauthor_id=30634657) | MIIT-AT VS SOC | -0.54061831 | -1.749745 | 0.6685079 | 1 |
| [Young-Gyun Seo 2019](https://pubmed.ncbi.nlm.nih.gov/?term=Seo+YG&cauthor_id=30634657) | NUT VS SOC | 0.83931933 | -1.560804 | 3.239443 | 1 |
| [Fabrício Vasconcellos 2016](https://pubmed.ncbi.nlm.nih.gov/?term=Vasconcellos+F&cauthor_id=26208409) | HIIT VS SOC | -0.46298147 | -1.100963 | 0.1750002 | 1 |
| [Fabrício Vasconcellos 2016](https://pubmed.ncbi.nlm.nih.gov/?term=Vasconcellos+F&cauthor_id=26208409) | HIIT-AT VS SOC | -0.10073634 | -2.037919 | 1.836446 | 1 |
| [Fabrício Vasconcellos 2016](https://pubmed.ncbi.nlm.nih.gov/?term=Vasconcellos+F&cauthor_id=26208409) | HIIT-MICT VS SOC | 0.72530165 | -1.221982 | 2.672585 | 1 |
| [Fabrício Vasconcellos 2016](https://pubmed.ncbi.nlm.nih.gov/?term=Vasconcellos+F&cauthor_id=26208409) | LICT VS SOC | -0.30779312 | -1.279495 | 0.6639087 | 1 |
| [Fabrício Vasconcellos 2016](https://pubmed.ncbi.nlm.nih.gov/?term=Vasconcellos+F&cauthor_id=26208409) | LICT-NUT VS SOC | 0.39139655 | -1.248225 | 2.031018 | 1 |
| [Fabrício Vasconcellos 2016](https://pubmed.ncbi.nlm.nih.gov/?term=Vasconcellos+F&cauthor_id=26208409) | MICT VS SOC | -2.443968 | -3.809128 | -1.078808 | 1 |
| [Fabrício Vasconcellos 2016](https://pubmed.ncbi.nlm.nih.gov/?term=Vasconcellos+F&cauthor_id=26208409) | MICT-AT VS SOC | -0.88992419 | -2.813187 | 1.033338 | 1 |
| [Fabrício Vasconcellos 2016](https://pubmed.ncbi.nlm.nih.gov/?term=Vasconcellos+F&cauthor_id=26208409) | MICT-NUT VS SOC | -0.30204206 | -1.338738 | 0.7346536 | 1 |
| [Fabrício Vasconcellos 2016](https://pubmed.ncbi.nlm.nih.gov/?term=Vasconcellos+F&cauthor_id=26208409) | MIIT VS SOC | -0.75230425 | -1.422873 | -0.081736 | 1 |
| [Fabrício Vasconcellos 2016](https://pubmed.ncbi.nlm.nih.gov/?term=Vasconcellos+F&cauthor_id=26208409) | MIIT-AT VS SOC | -0.53131493 | -1.747359 | 0.6847292 | 1 |
| [Fabrício Vasconcellos 2016](https://pubmed.ncbi.nlm.nih.gov/?term=Vasconcellos+F&cauthor_id=26208409) | NUT VS SOC | 0.8392469 | -1.575882 | 3.254375 | 1 |
| [Patricia C H Wong 2008](https://pubmed.ncbi.nlm.nih.gov/?term=Wong+PC&cauthor_id=18461212) | HIIT VS SOC | -0.46759038 | -1.084875 | 0.1496938 | 1 |
| [Patricia C H Wong 2008](https://pubmed.ncbi.nlm.nih.gov/?term=Wong+PC&cauthor_id=18461212) | HIIT-AT VS SOC | -0.10073634 | -1.982047 | 1.780574 | 1 |
| [Patricia C H Wong 2008](https://pubmed.ncbi.nlm.nih.gov/?term=Wong+PC&cauthor_id=18461212) | HIIT-MICT VS SOC | 0.72530165 | -1.166407 | 2.617011 | 1 |
| [Patricia C H Wong 2008](https://pubmed.ncbi.nlm.nih.gov/?term=Wong+PC&cauthor_id=18461212) | LICT VS SOC | -0.35943781 | -1.201143 | 0.4822669 | 1 |
| [Patricia C H Wong 2008](https://pubmed.ncbi.nlm.nih.gov/?term=Wong+PC&cauthor_id=18461212) | LICT-NUT VS SOC | 0.39181132 | -1.192656 | 1.976278 | 1 |
| [Patricia C H Wong 2008](https://pubmed.ncbi.nlm.nih.gov/?term=Wong+PC&cauthor_id=18461212) | MICT VS SOC | -2.427594 | -3.757808 | -1.09738 | 1 |
| [Patricia C H Wong 2008](https://pubmed.ncbi.nlm.nih.gov/?term=Wong+PC&cauthor_id=18461212) | MICT-AT VS SOC | -0.30114778 | -1.302904 | 0.7006083 | 1 |
| [Patricia C H Wong 2008](https://pubmed.ncbi.nlm.nih.gov/?term=Wong+PC&cauthor_id=18461212) | MICT-NUT VS SOC | -0.75485725 | -1.402608 | -0.1071069 | 1 |
| [Patricia C H Wong 2008](https://pubmed.ncbi.nlm.nih.gov/?term=Wong+PC&cauthor_id=18461212) | MIIT VS SOC | -0.53260691 | -1.709554 | 0.6443399 | 1 |
| [Patricia C H Wong 2008](https://pubmed.ncbi.nlm.nih.gov/?term=Wong+PC&cauthor_id=18461212) | MIIT-AT VS SOC | 0.83966183 | -1.493079 | 3.172403 | 1 |
| [Hong-Jie Yu 2020](https://pubmed.ncbi.nlm.nih.gov/?term=Yu+HJ&cauthor_id=31936767) | HIIT VS SOC | -0.46820952 | -1.104486 | 0.1680669 | 1 |
| [Hong-Jie Yu 2020](https://pubmed.ncbi.nlm.nih.gov/?term=Yu+HJ&cauthor_id=31936767) | HIIT-AT VS SOC | -0.10073634 | -2.037202 | 1.835729 | 1 |
| [Hong-Jie Yu 2020](https://pubmed.ncbi.nlm.nih.gov/?term=Yu+HJ&cauthor_id=31936767) | HIIT-MICT VS SOC | 0.72530165 | -1.221268 | 2.671871 | 1 |
| [Hong-Jie Yu 2020](https://pubmed.ncbi.nlm.nih.gov/?term=Yu+HJ&cauthor_id=31936767) | LICT VS SOC | -0.36041338 | -1.229533 | 0.5087066 | 1 |
| [Hong-Jie Yu 2020](https://pubmed.ncbi.nlm.nih.gov/?term=Yu+HJ&cauthor_id=31936767) | LICT-NUT VS SOC | 0.30085648 | -1.38257 | 1.984283 | 1 |
| [Hong-Jie Yu 2020](https://pubmed.ncbi.nlm.nih.gov/?term=Yu+HJ&cauthor_id=31936767) | MICT VS SOC | -2.4454484 | -3.810129 | -1.080768 | 1 |
| [Hong-Jie Yu 2020](https://pubmed.ncbi.nlm.nih.gov/?term=Yu+HJ&cauthor_id=31936767) | MICT-AT VS SOC | -0.88992419 | -2.812464 | 1.032616 | 1 |
| [Hong-Jie Yu 2020](https://pubmed.ncbi.nlm.nih.gov/?term=Yu+HJ&cauthor_id=31936767) | MICT-NUT VS SOC | -0.48081378 | -1.76548 | 0.8038523 | 1 |
| [Hong-Jie Yu 2020](https://pubmed.ncbi.nlm.nih.gov/?term=Yu+HJ&cauthor_id=31936767) | MIIT VS SOC | -0.75834255 | -1.426776 | -0.0899095 | 1 |
| [Hong-Jie Yu 2020](https://pubmed.ncbi.nlm.nih.gov/?term=Yu+HJ&cauthor_id=31936767) | MIIT-AT VS SOC | -0.53279043 | -1.748273 | 0.6826924 | 1 |
| [Hong-Jie Yu 2020](https://pubmed.ncbi.nlm.nih.gov/?term=Yu+HJ&cauthor_id=31936767) | NUT VS SOC | 0.7487072 | -1.695804 | 3.193218 | 1 |
| [Chongwen Zuo 2023](https://pubmed.ncbi.nlm.nih.gov/?term=Zuo+C&cauthor_id=37946224) | HIIT VS SOC | -0.40545457 | -1.049381 | 0.2384719 | 1 |
| [Chongwen Zuo 2023](https://pubmed.ncbi.nlm.nih.gov/?term=Zuo+C&cauthor_id=37946224) | HIIT-AT VS SOC | -0.10073634 | -2.007944 | 1.806471 | 1 |
| [Chongwen Zuo 2023](https://pubmed.ncbi.nlm.nih.gov/?term=Zuo+C&cauthor_id=37946224) | HIIT-MICT VS SOC | 0.72530165 | -1.192164 | 2.642768 | 1 |
| [Chongwen Zuo 2023](https://pubmed.ncbi.nlm.nih.gov/?term=Zuo+C&cauthor_id=37946224) | LICT VS SOC | -0.36290168 | -1.217517 | 0.4917132 | 1 |
| [Chongwen Zuo 2023](https://pubmed.ncbi.nlm.nih.gov/?term=Zuo+C&cauthor_id=37946224) | LICT-NUT VS SOC | 0.39161347 | -1.218453 | 2.001679 | 1 |
| [Chongwen Zuo 2023](https://pubmed.ncbi.nlm.nih.gov/?term=Zuo+C&cauthor_id=37946224) | MICT VS SOC | -2.4157364 | -3.763834 | -1.067639 | 1 |
| [Chongwen Zuo 2023](https://pubmed.ncbi.nlm.nih.gov/?term=Zuo+C&cauthor_id=37946224) | MICT-AT VS SOC | -0.88992419 | -2.782991 | 1.003143 | 1 |
| [Chongwen Zuo 2023](https://pubmed.ncbi.nlm.nih.gov/?term=Zuo+C&cauthor_id=37946224) | MICT-NUT VS SOC | -0.30157417 | -1.319549 | 0.7164011 | 1 |
| [Chongwen Zuo 2023](https://pubmed.ncbi.nlm.nih.gov/?term=Zuo+C&cauthor_id=37946224) | MIIT VS SOC | -0.82865995 | -1.508746 | -0.148574 | 1 |
| [Chongwen Zuo 2023](https://pubmed.ncbi.nlm.nih.gov/?term=Zuo+C&cauthor_id=37946224) | MIIT-AT VS SOC | -0.51508081 | -1.710887 | 0.6807253 | 1 |
| [Chongwen Zuo 2023](https://pubmed.ncbi.nlm.nih.gov/?term=Zuo+C&cauthor_id=37946224) | NUT VS SOC | 0.8394639 | -1.531521 | 3.210448 | 1 |
| [Yuhang Gao 2025](https://pubmed.ncbi.nlm.nih.gov/?term=Gao+Y&cauthor_id=41006526) | HIIT VS SOC | -0.46759038 | -1.084875 | 0.1496938 | 1 |
| [Yuhang Gao 2025](https://pubmed.ncbi.nlm.nih.gov/?term=Gao+Y&cauthor_id=41006526) | HIIT-AT VS SOC | -0.35943781 | -1.201143 | 0.4822669 | 1 |
| [Yuhang Gao 2025](https://pubmed.ncbi.nlm.nih.gov/?term=Gao+Y&cauthor_id=41006526) | HIIT-MICT VS SOC | 0.39181132 | -1.192656 | 1.976278 | 1 |
| [Yuhang Gao 2025](https://pubmed.ncbi.nlm.nih.gov/?term=Gao+Y&cauthor_id=41006526) | LICT VS SOC | -2.427594 | -3.757807 | -1.097381 | 1 |
| [Yuhang Gao 2025](https://pubmed.ncbi.nlm.nih.gov/?term=Gao+Y&cauthor_id=41006526) | LICT-NUT VS SOC | -0.88992419 | -2.756898 | 0.9770494 | 1 |
| [Yuhang Gao 2025](https://pubmed.ncbi.nlm.nih.gov/?term=Gao+Y&cauthor_id=41006526) | MICT VS SOC | -0.30114778 | -1.302904 | 0.7006083 | 1 |
| [Yuhang Gao 2025](https://pubmed.ncbi.nlm.nih.gov/?term=Gao+Y&cauthor_id=41006526) | MICT-AT VS SOC | -0.75485725 | -1.402608 | -0.107107 | 1 |
| [Yuhang Gao 2025](https://pubmed.ncbi.nlm.nih.gov/?term=Gao+Y&cauthor_id=41006526) | MICT-NUT VS SOC | -0.53260691 | -1.709554 | 0.64434 | 1 |
| [Yuhang Gao 2025](https://pubmed.ncbi.nlm.nih.gov/?term=Gao+Y&cauthor_id=41006526) | MIIT VS SOC | 0.83966183 | -1.493079 | 3.172403 | 1 |
| Shitong Shao 2025 | HIIT VS SOC | -0.49603878 | -1.159606 | 0.1675282 | 1 |
| Shitong Shao 2025 | HIIT-AT VS SOC | -0.10073635 | -2.097631 | 1.896158 | 1 |
| Shitong Shao 2025 | HIIT-MICT VS SOC | 0.72530165 | -1.281393 | 2.731996 | 1 |
| Shitong Shao 2025 | LICT VS SOC | -0.2989794 | -1.366602 | 0.7686436 | 1 |
| Shitong Shao 2025 | LICT-NUT VS SOC | 0.39099934 | -1.307278 | 2.089277 | 1 |
| Shitong Shao 2025 | MICT VS SOC | -2.4724579 | -3.875053 | -1.069863 | 1 |
| Shitong Shao 2025 | MICT-AT VS SOC | -0.88992419 | -2.873318 | 1.093469 | 1 |
| Shitong Shao 2025 | MICT-NUT VS SOC | -0.30289998 | -1.376745 | 0.7709454 | 1 |
| Shitong Shao 2025 | MIIT VS SOC | -0.85802482 | -1.613118 | -0.1029313 | 1 |
| Shitong Shao 2025 | MIIT-AT VS SOC | -0.54066035 | -1.798497 | 0.7171764 | 1 |
| Shitong Shao 2025 | NUT VS SOC | 0.83884949 | -1.663858 | 3.341557 | 1 |
| Ting Liao 2024 | HIIT VS SOC | -0.45409046 | -1.110556 | 0.2023748 | 1 |
| Ting Liao 2024 | HIIT-AT VS SOC | -0.10073634 | -2.044768 | 1.843295 | 1 |
| Ting Liao 2024 | HIIT-MICT VS SOC | 0.72530166 | -1.228795 | 2.679398 | 1 |
| Ting Liao 2024 | LICT VS SOC | -0.36121396 | -1.234114 | 0.511686 | 1 |
| Ting Liao 2024 | LICT-NUT VS SOC | 0.39134872 | -1.255014 | 2.037712 | 1 |
| Ting Liao 2024 | MICT VS SOC | -2.4431835 | -3.8132 | -1.073167 | 1 |
| Ting Liao 2024 | MICT-AT VS SOC | -0.88992419 | -2.820085 | 1.040236 | 1 |
| Ting Liao 2024 | MICT-NUT VS SOC | -0.30214532 | -1.343111 | 0.7388202 | 1 |
| Ting Liao 2024 | MIIT VS SOC | -0.77525227 | -1.469133 | -0.0813716 | 1 |
| Ting Liao 2024 | MIIT-AT VS SOC | -0.52880611 | -1.750308 | 0.6926962 | 1 |
| Ting Liao 2024 | NUT VS SOC | 0.83919904 | -1.585998 | 3.264396 | 1 |
| [Neiva Leite 2024](https://link.springer.com/article/10.1007/s00431-024-05734-w#auth-Neiva-Leite-Aff1-Aff2) | HIIT VS SOC | -0.54182971 | -1.212999 | 0.1293391 | 1 |
| [Neiva Leite 2024](https://link.springer.com/article/10.1007/s00431-024-05734-w#auth-Neiva-Leite-Aff1-Aff2) | HIIT-AT VS SOC | -0.10073634 | -2.020542 | 1.819069 | 1 |
| [Neiva Leite 2024](https://link.springer.com/article/10.1007/s00431-024-05734-w#auth-Neiva-Leite-Aff1-Aff2) | HIIT-MICT VS SOC | 0.72530165 | -1.204695 | 2.655299 | 1 |
| [Neiva Leite 2024](https://link.springer.com/article/10.1007/s00431-024-05734-w#auth-Neiva-Leite-Aff1-Aff2) | LICT VS SOC | -0.37380868 | -1.235722 | 0.4881051 | 1 |
| [Neiva Leite 2024](https://link.springer.com/article/10.1007/s00431-024-05734-w#auth-Neiva-Leite-Aff1-Aff2) | LICT-NUT VS SOC | 0.39152078 | -1.230976 | 2.014018 | 1 |
| [Neiva Leite 2024](https://link.springer.com/article/10.1007/s00431-024-05734-w#auth-Neiva-Leite-Aff1-Aff2) | MICT VS SOC | -2.464259 | -3.820567 | -1.107951 | 1 |
| [Neiva Leite 2024](https://link.springer.com/article/10.1007/s00431-024-05734-w#auth-Neiva-Leite-Aff1-Aff2) | MICT-AT VS SOC | -0.88992419 | -2.795683 | 1.015834 | 1 |
| [Neiva Leite 2024](https://link.springer.com/article/10.1007/s00431-024-05734-w#auth-Neiva-Leite-Aff1-Aff2) | MICT-NUT VS SOC | -0.30177405 | -1.327623 | 0.7240754 | 1 |
| [Neiva Leite 2024](https://link.springer.com/article/10.1007/s00431-024-05734-w#auth-Neiva-Leite-Aff1-Aff2) | MIIT VS SOC | -0.78452462 | -1.452168 | -0.1168815 | 1 |
| [Neiva Leite 2024](https://link.springer.com/article/10.1007/s00431-024-05734-w#auth-Neiva-Leite-Aff1-Aff2) | MIIT-AT VS SOC | -0.55356202 | -1.759166 | 0.6520424 | 1 |
| [Neiva Leite 2024](https://link.springer.com/article/10.1007/s00431-024-05734-w#auth-Neiva-Leite-Aff1-Aff2) | NUT VS SOC | 0.83937117 | -1.550182 | 3.228924 | 1 |
| Noelia González-Gálvez 2024 | HIIT VS SOC | -0.63593359 | -1.2135 | -0.0583674 | 1 |
| Noelia González-Gálvez 2024 | HIIT-AT VS SOC | -0.10073634 | -1.774426 | 1.572954 | 1 |
| Noelia González-Gálvez 2024 | HIIT-MICT VS SOC | 0.72530165 | -0.9600686 | 2.410672 | 1 |
| Noelia González-Gálvez 2024 | LICT VS SOC | -0.35289737 | -1.091545 | 0.3857498 | 1 |
| Noelia González-Gálvez 2024 | LICT-NUT VS SOC | 0.39385212 | -0.9828526 | 1.770557 | 1 |
| Noelia González-Gálvez 2024 | MICT VS SOC | -2.4055016 | -3.616008 | -1.194995 | 1 |
| Noelia González-Gálvez 2024 | MICT-AT VS SOC | -0.88992419 | -2.547483 | 0.7676343 | 1 |
| Noelia González-Gálvez 2024 | MICT-NUT VS SOC | -0.29677108 | -1.166849 | 0.5733069 | 1 |
| Noelia González-Gálvez 2024 | MIIT VS SOC | -0.59073468 | -1.19322 | 0.0117512 | 1 |
| Noelia González-Gálvez 2024 | MIIT-AT VS SOC | -0.57985531 | -1.611789 | 0.4520781 | 1 |
| Noelia González-Gálvez 2024 | NUT VS SOC | 0.84170322 | -1.180271 | 2.863678 | 1 |
| [Emir Tas 2023](https://onlinelibrary.wiley.com/authored-by/Tas/Emir) | HIIT VS SOC | -0.52628075 | -1.195803 | 0.1432419 | 1 |
| [Emir Tas 2023](https://onlinelibrary.wiley.com/authored-by/Tas/Emir) | HIIT-AT VS SOC | -0.10073634 | -2.025623 | 1.82415 | 1 |
| [Emir Tas 2023](https://onlinelibrary.wiley.com/authored-by/Tas/Emir) | HIIT-MICT VS SOC | 0.72530166 | -1.20975 | 2.660353 | 1 |
| [Emir Tas 2023](https://onlinelibrary.wiley.com/authored-by/Tas/Emir) | LICT VS SOC | -0.37100805 | -1.235362 | 0.4933456 | 1 |
| [Emir Tas 2023](https://onlinelibrary.wiley.com/authored-by/Tas/Emir) | LICT-NUT VS SOC | 0.39148403 | -1.236023 | 2.018991 | 1 |
| [Emir Tas 2023](https://onlinelibrary.wiley.com/authored-by/Tas/Emir) | MICT VS SOC | -2.4607749 | -3.820191 | -1.101359 | 1 |
| [Emir Tas 2023](https://onlinelibrary.wiley.com/authored-by/Tas/Emir) | MICT-AT VS SOC | -0.88992419 | -2.800801 | 1.020953 | 1 |
| [Emir Tas 2023](https://onlinelibrary.wiley.com/authored-by/Tas/Emir) | MICT-NUT VS SOC | -0.30185333 | -1.330876 | 0.727169 | 1 |
| [Emir Tas 2023](https://onlinelibrary.wiley.com/authored-by/Tas/Emir) | MIIT VS SOC | -0.77909203 | -1.448248 | -0.1099362 | 1 |
| [Emir Tas 2023](https://onlinelibrary.wiley.com/authored-by/Tas/Emir) | MIIT-AT VS SOC | -0.54917639 | -1.758178 | 0.659825 | 1 |
| [Emir Tas 2023](https://onlinelibrary.wiley.com/authored-by/Tas/Emir) | NUT VS SOC | 0.83933441 | -1.557701 | 3.23637 | 1 |
| [Ghazi Racil 2024](https://www.tandfonline.com/author/Racil,+Ghazi) | HIIT VS SOC | -0.43722218 | -1.122508 | 0.2480634 | 1 |
| [Ghazi Racil 2024](https://www.tandfonline.com/author/Racil,+Ghazi) | HIIT-AT VS SOC | -0.10073631 | -2.0633 | 1.861827 | 1 |
| [Ghazi Racil 2024](https://www.tandfonline.com/author/Racil,+Ghazi) | HIIT-MICT VS SOC | 0.72530169 | -1.247232 | 2.697835 | 1 |
| [Ghazi Racil 2024](https://www.tandfonline.com/author/Racil,+Ghazi) | LICT VS SOC | -0.37282792 | -1.256489 | 0.5108327 | 1 |
| [Ghazi Racil 2024](https://www.tandfonline.com/author/Racil,+Ghazi) | LICT-NUT VS SOC | 0.39122235 | -1.273366 | 2.05581 | 1 |
| [Ghazi Racil 2024](https://www.tandfonline.com/author/Racil,+Ghazi) | MICT VS SOC | -2.4433284 | -3.826859 | -1.059798 | 1 |
| [Ghazi Racil 2024](https://www.tandfonline.com/author/Racil,+Ghazi) | MICT-AT VS SOC | -0.88992418 | -2.838749 | 1.0589 | 1 |
| [Ghazi Racil 2024](https://www.tandfonline.com/author/Racil,+Ghazi) | MICT-NUT VS SOC | -0.30241822 | -1.354928 | 0.7500921 | 1 |
| [Ghazi Racil 2024](https://www.tandfonline.com/author/Racil,+Ghazi) | MIIT VS SOC | -0.8651109 | -1.591749 | -0.1384725 | 1 |
| [Ghazi Racil 2024](https://www.tandfonline.com/author/Racil,+Ghazi) | MIIT-AT VS SOC | -0.52404516 | -1.759439 | 0.7113483 | 1 |
| [Ghazi Racil 2024](https://www.tandfonline.com/author/Racil,+Ghazi) | NUT VS SOC | 0.83907263 | -1.613338 | 3.291483 | 1 |

**Table S22** Sensitivity Analysis Using DBP as the Outcome Measure.

| **dropped_id** | **comparison** | **eff** | **lci** | **uci** | **connected** |
| --- | --- | --- | --- | --- | --- |
| [María José Aguilar-Cordero 2020](https://pubmed.ncbi.nlm.nih.gov/?term=Aguilar-Cordero+MJ&cauthor_id=31678988) | HIIT VS SOC | -0.30895605 | -0.7690948 | 0.1511827 | 1 |
| [María José Aguilar-Cordero 2020](https://pubmed.ncbi.nlm.nih.gov/?term=Aguilar-Cordero+MJ&cauthor_id=31678988) | HIIT-AT VS SOC | -0.22361457 | -1.561668 | 1.114439 | 1 |
| [María José Aguilar-Cordero 2020](https://pubmed.ncbi.nlm.nih.gov/?term=Aguilar-Cordero+MJ&cauthor_id=31678988) | HIIT-MICT VS SOC | -0.33542185 | -1.67525 | 1.004406 | 1 |
| [María José Aguilar-Cordero 2020](https://pubmed.ncbi.nlm.nih.gov/?term=Aguilar-Cordero+MJ&cauthor_id=31678988) | LICT VS SOC | -0.05635994 | -0.6244104 | 0.5116905 | 1 |
| [María José Aguilar-Cordero 2020](https://pubmed.ncbi.nlm.nih.gov/?term=Aguilar-Cordero+MJ&cauthor_id=31678988) | LICT-NUT VS SOC | -0.28887092 | -1.310965 | 0.7332227 | 1 |
| [María José Aguilar-Cordero 2020](https://pubmed.ncbi.nlm.nih.gov/?term=Aguilar-Cordero+MJ&cauthor_id=31678988) | MICT VS SOC | 1.1064256 | -0.1048881 | 2.317739 | 1 |
| [María José Aguilar-Cordero 2020](https://pubmed.ncbi.nlm.nih.gov/?term=Aguilar-Cordero+MJ&cauthor_id=31678988) | MICT-AT VS SOC | -0.20527062 | -1.49466 | 1.084119 | 1 |
| [María José Aguilar-Cordero 2020](https://pubmed.ncbi.nlm.nih.gov/?term=Aguilar-Cordero+MJ&cauthor_id=31678988) | MICT-NUT VS SOC | -0.57431486 | -1.220135 | 0.0715056 | 1 |
| [María José Aguilar-Cordero 2020](https://pubmed.ncbi.nlm.nih.gov/?term=Aguilar-Cordero+MJ&cauthor_id=31678988) | MIIT VS SOC | -0.66586023 | -1.146527 | -0.1851936 | 1 |
| [María José Aguilar-Cordero 2020](https://pubmed.ncbi.nlm.nih.gov/?term=Aguilar-Cordero+MJ&cauthor_id=31678988) | MIIT-AT VS SOC | -0.47796656 | -1.26643 | 0.3104971 | 1 |
| [Thaynã Alves Bezerra 2022](https://pubmed.ncbi.nlm.nih.gov/?term=Bezerra+TA&cauthor_id=35699999) | HIIT VS SOC | -0.30794572 | -0.7858531 | 0.1699616 | 1 |
| [Thaynã Alves Bezerra 2022](https://pubmed.ncbi.nlm.nih.gov/?term=Bezerra+TA&cauthor_id=35699999) | HIIT-AT VS SOC | -0.22361457 | -1.60741 | 1.160181 | 1 |
| [Thaynã Alves Bezerra 2022](https://pubmed.ncbi.nlm.nih.gov/?term=Bezerra+TA&cauthor_id=35699999) | HIIT-MICT VS SOC | -0.33542185 | -1.720934 | 1.05009 | 1 |
| [Thaynã Alves Bezerra 2022](https://pubmed.ncbi.nlm.nih.gov/?term=Bezerra+TA&cauthor_id=35699999) | LICT VS SOC | -0.06447071 | -0.6566835 | 0.5277421 | 1 |
| [Thaynã Alves Bezerra 2022](https://pubmed.ncbi.nlm.nih.gov/?term=Bezerra+TA&cauthor_id=35699999) | LICT-NUT VS SOC | -0.27889671 | -1.374656 | 0.8168623 | 1 |
| [Thaynã Alves Bezerra 2022](https://pubmed.ncbi.nlm.nih.gov/?term=Bezerra+TA&cauthor_id=35699999) | MICT VS SOC | 1.1064256 | -0.1552335 | 2.368085 | 1 |
| [Thaynã Alves Bezerra 2022](https://pubmed.ncbi.nlm.nih.gov/?term=Bezerra+TA&cauthor_id=35699999) | MICT-AT VS SOC | -0.20527062 | -1.542068 | 1.131527 | 1 |
| [Thaynã Alves Bezerra 2022](https://pubmed.ncbi.nlm.nih.gov/?term=Bezerra+TA&cauthor_id=35699999) | MICT-NUT VS SOC | -0.55530818 | -1.364154 | 0.2535374 | 1 |
| [Thaynã Alves Bezerra 2022](https://pubmed.ncbi.nlm.nih.gov/?term=Bezerra+TA&cauthor_id=35699999) | MIIT VS SOC | -0.6745409 | -1.174853 | -0.1742292 | 1 |
| [Thaynã Alves Bezerra 2022](https://pubmed.ncbi.nlm.nih.gov/?term=Bezerra+TA&cauthor_id=35699999) | MIIT-AT VS SOC | -0.47735139 | -1.299791 | 0.3450879 | 1 |
| [Thaynã Alves Bezerra 2022](https://pubmed.ncbi.nlm.nih.gov/?term=Bezerra+TA&cauthor_id=35699999) | NUT VS SOC | 0.44716302 | -1.137485 | 2.031811 | 1 |
| [N Cvetković 2018](https://pubmed.ncbi.nlm.nih.gov/?term=Cvetkovi%C4%87+N&cauthor_id=29979479) | HIIT VS SOC | -0.23345316 | -0.7164278 | 0.2495215 | 1 |
| [N Cvetković 2018](https://pubmed.ncbi.nlm.nih.gov/?term=Cvetkovi%C4%87+N&cauthor_id=29979479) | HIIT-AT VS SOC | -0.22361457 | -1.527152 | 1.079923 | 1 |
| [N Cvetković 2018](https://pubmed.ncbi.nlm.nih.gov/?term=Cvetkovi%C4%87+N&cauthor_id=29979479) | HIIT-MICT VS SOC | -0.33542185 | -1.640782 | 0.969938 | 1 |
| [N Cvetković 2018](https://pubmed.ncbi.nlm.nih.gov/?term=Cvetkovi%C4%87+N&cauthor_id=29979479) | LICT VS SOC | 0.11596264 | -0.5036566 | 0.7355819 | 1 |
| [N Cvetković 2018](https://pubmed.ncbi.nlm.nih.gov/?term=Cvetkovi%C4%87+N&cauthor_id=29979479) | LICT-NUT VS SOC | -0.28904361 | -1.273211 | 0.6951234 | 1 |
| [N Cvetković 2018](https://pubmed.ncbi.nlm.nih.gov/?term=Cvetkovi%C4%87+N&cauthor_id=29979479) | MICT VS SOC | 1.1064256 | -0.0666495 | 2.279501 | 1 |
| [N Cvetković 2018](https://pubmed.ncbi.nlm.nih.gov/?term=Cvetkovi%C4%87+N&cauthor_id=29979479) | MICT-AT VS SOC | -0.20527062 | -1.458806 | 1.048264 | 1 |
| [N Cvetković 2018](https://pubmed.ncbi.nlm.nih.gov/?term=Cvetkovi%C4%87+N&cauthor_id=29979479) | MICT-NUT VS SOC | -0.57438988 | -1.195989 | 0.0472088 | 1 |
| [N Cvetković 2018](https://pubmed.ncbi.nlm.nih.gov/?term=Cvetkovi%C4%87+N&cauthor_id=29979479) | MIIT VS SOC | -0.61288106 | -1.083752 | -0.1420101 | 1 |
| [N Cvetković 2018](https://pubmed.ncbi.nlm.nih.gov/?term=Cvetkovi%C4%87+N&cauthor_id=29979479) | MIIT-AT VS SOC | -0.45720915 | -1.221575 | 0.3071569 | 1 |
| [N Cvetković 2018](https://pubmed.ncbi.nlm.nih.gov/?term=Cvetkovi%C4%87+N&cauthor_id=29979479) | NUT VS SOC | 0.43701616 | -0.9994127 | 1.873445 | 1 |
| [Catherine L Davis 2020](https://pubmed.ncbi.nlm.nih.gov/?term=Davis+CL&cauthor_id=31754238) | HIIT VS SOC | -0.38310238 | -0.6539444 | -0.1122604 | 1 |
| [Catherine L Davis 2020](https://pubmed.ncbi.nlm.nih.gov/?term=Davis+CL&cauthor_id=31754238) | HIIT-AT VS SOC | -0.22361457 | -1.10232 | 0.6550906 | 1 |
| [Catherine L Davis 2020](https://pubmed.ncbi.nlm.nih.gov/?term=Davis+CL&cauthor_id=31754238) | HIIT-MICT VS SOC | -0.33542185 | -1.216828 | 0.5459839 | 1 |
| [Catherine L Davis 2020](https://pubmed.ncbi.nlm.nih.gov/?term=Davis+CL&cauthor_id=31754238) | LICT VS SOC | -0.46419424 | -0.8123195 | -0.1160691 | 1 |
| [Catherine L Davis 2020](https://pubmed.ncbi.nlm.nih.gov/?term=Davis+CL&cauthor_id=31754238) | LICT-NUT VS SOC | -0.30376722 | -0.7373282 | 0.1297937 | 1 |
| [Catherine L Davis 2020](https://pubmed.ncbi.nlm.nih.gov/?term=Davis+CL&cauthor_id=31754238) | MICT VS SOC | 1.1064256 | 0.4363384 | 1.776513 | 1 |
| [Catherine L Davis 2020](https://pubmed.ncbi.nlm.nih.gov/?term=Davis+CL&cauthor_id=31754238) | MICT-AT VS SOC | -0.20527062 | -1.00793 | 0.5973884 | 1 |
| [Catherine L Davis 2020](https://pubmed.ncbi.nlm.nih.gov/?term=Davis+CL&cauthor_id=31754238) | MICT-NUT VS SOC | -0.58727336 | -0.8297934 | -0.3447533 | 1 |
| [Catherine L Davis 2020](https://pubmed.ncbi.nlm.nih.gov/?term=Davis+CL&cauthor_id=31754238) | MIIT VS SOC | -0.59871089 | -0.8450183 | -0.3524035 | 1 |
| [Catherine L Davis 2020](https://pubmed.ncbi.nlm.nih.gov/?term=Davis+CL&cauthor_id=31754238) | MIIT-AT VS SOC | -0.50760163 | -0.9242743 | -0.0909289 | 1 |
| [Catherine L Davis 2020](https://pubmed.ncbi.nlm.nih.gov/?term=Davis+CL&cauthor_id=31754238) | NUT VS SOC | 0.42229264 | -0.1740575 | 1.018643 | 1 |
| [Nathalie J Farpour-Lambert 2009](https://pubmed.ncbi.nlm.nih.gov/?term=Farpour-Lambert+NJ&cauthor_id=20082930) | HIIT VS SOC | -0.28841818 | -0.7649912 | 0.1881548 | 1 |
| [Nathalie J Farpour-Lambert 2009](https://pubmed.ncbi.nlm.nih.gov/?term=Farpour-Lambert+NJ&cauthor_id=20082930) | HIIT-AT VS SOC | -0.22361457 | -1.591165 | 1.143936 | 1 |
| [Nathalie J Farpour-Lambert 2009](https://pubmed.ncbi.nlm.nih.gov/?term=Farpour-Lambert+NJ&cauthor_id=20082930) | HIIT-MICT VS SOC | -0.33542185 | -1.704709 | 1.033865 | 1 |
| [Nathalie J Farpour-Lambert 2009](https://pubmed.ncbi.nlm.nih.gov/?term=Farpour-Lambert+NJ&cauthor_id=20082930) | LICT VS SOC | -0.05811029 | -0.6420211 | 0.5258005 | 1 |
| [Nathalie J Farpour-Lambert 2009](https://pubmed.ncbi.nlm.nih.gov/?term=Farpour-Lambert+NJ&cauthor_id=20082930) | LICT-NUT VS SOC | -0.28874141 | -1.342905 | 0.7654225 | 1 |
| [Nathalie J Farpour-Lambert 2009](https://pubmed.ncbi.nlm.nih.gov/?term=Farpour-Lambert+NJ&cauthor_id=20082930) | MICT VS SOC | 1.1064256 | -0.1373945 | 2.350246 | 1 |
| [Nathalie J Farpour-Lambert 2009](https://pubmed.ncbi.nlm.nih.gov/?term=Farpour-Lambert+NJ&cauthor_id=20082930) | MICT-AT VS SOC | -0.20527062 | -1.525245 | 1.114704 | 1 |
| [Nathalie J Farpour-Lambert 2009](https://pubmed.ncbi.nlm.nih.gov/?term=Farpour-Lambert+NJ&cauthor_id=20082930) | MICT-NUT VS SOC | -0.57426127 | -1.240535 | 0.0920125 | 1 |
| [Nathalie J Farpour-Lambert 2009](https://pubmed.ncbi.nlm.nih.gov/?term=Farpour-Lambert+NJ&cauthor_id=20082930) | MIIT VS SOC | -0.66357808 | -1.157907 | -0.1692493 | 1 |
| [Nathalie J Farpour-Lambert 2009](https://pubmed.ncbi.nlm.nih.gov/?term=Farpour-Lambert+NJ&cauthor_id=20082930) | MIIT-AT VS SOC | -0.26732556 | -1.355852 | 0.8212011 | 1 |
| [Nathalie J Farpour-Lambert 2009](https://pubmed.ncbi.nlm.nih.gov/?term=Farpour-Lambert+NJ&cauthor_id=20082930) | NUT VS SOC | 0.43731834 | -1.10443 | 1.979066 | 1 |
| [Vandana Jain 2022](https://pubmed.ncbi.nlm.nih.gov/?term=Jain+V&cauthor_id=36124513) | disconnected |  |  |  | 0 |
| [Jun Kim 2021](https://pubmed.ncbi.nlm.nih.gov/?term=Kim+J&cauthor_id=31812946) | HIIT VS SOC | -0.30327153 | -0.7867174 | 0.1801743 | 1 |
| [Jun Kim 2021](https://pubmed.ncbi.nlm.nih.gov/?term=Kim+J&cauthor_id=31812946) | HIIT-AT VS SOC | -0.22361457 | -1.603833 | 1.156604 | 1 |
| [Jun Kim 2021](https://pubmed.ncbi.nlm.nih.gov/?term=Kim+J&cauthor_id=31812946) | HIIT-MICT VS SOC | -0.33542185 | -1.717361 | 1.046517 | 1 |
| [Jun Kim 2021](https://pubmed.ncbi.nlm.nih.gov/?term=Kim+J&cauthor_id=31812946) | LICT VS SOC | -0.06126124 | -0.65385 | 0.5313275 | 1 |
| [Jun Kim 2021](https://pubmed.ncbi.nlm.nih.gov/?term=Kim+J&cauthor_id=31812946) | LICT-NUT VS SOC | -0.28869004 | -1.356539 | 0.7791589 | 1 |
| [Jun Kim 2021](https://pubmed.ncbi.nlm.nih.gov/?term=Kim+J&cauthor_id=31812946) | MICT VS SOC | 1.1064256 | -0.1513093 | 2.364161 | 1 |
| [Jun Kim 2021](https://pubmed.ncbi.nlm.nih.gov/?term=Kim+J&cauthor_id=31812946) | MICT-AT VS SOC | -0.20527062 | -1.538365 | 1.127824 | 1 |
| [Jun Kim 2021](https://pubmed.ncbi.nlm.nih.gov/?term=Kim+J&cauthor_id=31812946) | MICT-NUT VS SOC | -0.57424069 | -1.249235 | 0.1007536 | 1 |
| [Jun Kim 2021](https://pubmed.ncbi.nlm.nih.gov/?term=Kim+J&cauthor_id=31812946) | MIIT VS SOC | -0.66125435 | -1.208461 | -0.1140482 | 1 |
| [Jun Kim 2021](https://pubmed.ncbi.nlm.nih.gov/?term=Kim+J&cauthor_id=31812946) | MIIT-AT VS SOC | -0.47606909 | -1.296172 | 0.3440343 | 1 |
| [Jun Kim 2021](https://pubmed.ncbi.nlm.nih.gov/?term=Kim+J&cauthor_id=31812946) | NUT VS SOC | 0.43736971 | -1.124948 | 1.999687 | 1 |
| [Yun Hee Lee 2010](https://pubmed.ncbi.nlm.nih.gov/?term=Lee+YH&cauthor_id=20421958) | HIIT VS SOC | -0.2148567 | -0.7393327 | 0.3096193 | 1 |
| [Yun Hee Lee 2010](https://pubmed.ncbi.nlm.nih.gov/?term=Lee+YH&cauthor_id=20421958) | HIIT-AT VS SOC | -0.22361457 | -1.613361 | 1.166132 | 1 |
| [Yun Hee Lee 2010](https://pubmed.ncbi.nlm.nih.gov/?term=Lee+YH&cauthor_id=20421958) | HIIT-MICT VS SOC | -0.33542185 | -1.726878 | 1.056034 | 1 |
| [Yun Hee Lee 2010](https://pubmed.ncbi.nlm.nih.gov/?term=Lee+YH&cauthor_id=20421958) | LICT VS SOC | -0.04845257 | -0.645237 | 0.5483319 | 1 |
| [Yun Hee Lee 2010](https://pubmed.ncbi.nlm.nih.gov/?term=Lee+YH&cauthor_id=20421958) | LICT-NUT VS SOC | -0.28865295 | -1.366762 | 0.7894565 | 1 |
| [Yun Hee Lee 2010](https://pubmed.ncbi.nlm.nih.gov/?term=Lee+YH&cauthor_id=20421958) | MICT VS SOC | 1.1064256 | -0.1617584 | 2.37461 | 1 |
| [Yun Hee Lee 2010](https://pubmed.ncbi.nlm.nih.gov/?term=Lee+YH&cauthor_id=20421958) | MICT-AT VS SOC | -0.20527062 | -1.548228 | 1.137687 | 1 |
| [Yun Hee Lee 2010](https://pubmed.ncbi.nlm.nih.gov/?term=Lee+YH&cauthor_id=20421958) | MICT-NUT VS SOC | -0.57422609 | -1.255756 | 0.1073039 | 1 |
| [Yun Hee Lee 2010](https://pubmed.ncbi.nlm.nih.gov/?term=Lee+YH&cauthor_id=20421958) | MIIT VS SOC | -0.6382382 | -1.148557 | -0.1279191 | 1 |
| [Yun Hee Lee 2010](https://pubmed.ncbi.nlm.nih.gov/?term=Lee+YH&cauthor_id=20421958) | MIIT-AT VS SOC | -0.7390002 | -1.977777 | 0.499777 | 1 |
| [Yun Hee Lee 2010](https://pubmed.ncbi.nlm.nih.gov/?term=Lee+YH&cauthor_id=20421958) | NUT VS SOC | 0.43740679 | -1.140328 | 2.015141 | 1 |
| [Won-Mok Son 2017](https://pubmed.ncbi.nlm.nih.gov/?term=Son+WM&cauthor_id=28590143) | HIIT VS SOC | -0.30895605 | -0.7690947 | 0.1511826 | 1 |
| [Won-Mok Son 2017](https://pubmed.ncbi.nlm.nih.gov/?term=Son+WM&cauthor_id=28590143) | HIIT-AT VS SOC | -0.22361457 | -1.561668 | 1.114439 | 1 |
| [Won-Mok Son 2017](https://pubmed.ncbi.nlm.nih.gov/?term=Son+WM&cauthor_id=28590143) | HIIT-MICT VS SOC | -0.33542185 | -1.67525 | 1.004406 | 1 |
| [Won-Mok Son 2017](https://pubmed.ncbi.nlm.nih.gov/?term=Son+WM&cauthor_id=28590143) | LICT VS SOC | -0.0563599 | -0.6244102 | 0.5116903 | 1 |
| [Won-Mok Son 2017](https://pubmed.ncbi.nlm.nih.gov/?term=Son+WM&cauthor_id=28590143) | LICT-NUT VS SOC | -0.28887112 | -1.310963 | 0.7332209 | 1 |
| [Won-Mok Son 2017](https://pubmed.ncbi.nlm.nih.gov/?term=Son+WM&cauthor_id=28590143) | MICT VS SOC | -0.20527062 | -1.49466 | 1.084119 | 1 |
| [Won-Mok Son 2017](https://pubmed.ncbi.nlm.nih.gov/?term=Son+WM&cauthor_id=28590143) | MICT-AT VS SOC | -0.57431491 | -1.220135 | 0.0715053 | 1 |
| [Won-Mok Son 2017](https://pubmed.ncbi.nlm.nih.gov/?term=Son+WM&cauthor_id=28590143) | MICT-NUT VS SOC | -0.66586018 | -1.146527 | -0.1851937 | 1 |
| [Won-Mok Son 2017](https://pubmed.ncbi.nlm.nih.gov/?term=Son+WM&cauthor_id=28590143) | MIIT VS SOC | -0.47796657 | -1.26643 | 0.3104969 | 1 |
| [Won-Mok Son 2017](https://pubmed.ncbi.nlm.nih.gov/?term=Son+WM&cauthor_id=28590143) | MIIT-AT VS SOC | 0.43718863 | -1.056327 | 1.930704 | 1 |
| [Young-Gyun Seo 2019](https://pubmed.ncbi.nlm.nih.gov/?term=Seo+YG&cauthor_id=30634657) | HIIT VS SOC | -0.32939299 | -0.8131169 | 0.1543309 | 1 |
| [Young-Gyun Seo 2019](https://pubmed.ncbi.nlm.nih.gov/?term=Seo+YG&cauthor_id=30634657) | HIIT-AT VS SOC | -0.22361457 | -1.603633 | 1.156404 | 1 |
| [Young-Gyun Seo 2019](https://pubmed.ncbi.nlm.nih.gov/?term=Seo+YG&cauthor_id=30634657) | HIIT-MICT VS SOC | -0.33542185 | -1.717161 | 1.046317 | 1 |
| [Young-Gyun Seo 2019](https://pubmed.ncbi.nlm.nih.gov/?term=Seo+YG&cauthor_id=30634657) | LICT VS SOC | -0.07560799 | -0.6678416 | 0.5166256 | 1 |
| [Young-Gyun Seo 2019](https://pubmed.ncbi.nlm.nih.gov/?term=Seo+YG&cauthor_id=30634657) | LICT-NUT VS SOC | -0.28869083 | -1.356324 | 0.7789422 | 1 |
| [Young-Gyun Seo 2019](https://pubmed.ncbi.nlm.nih.gov/?term=Seo+YG&cauthor_id=30634657) | MICT VS SOC | 1.1064256 | -0.1510895 | 2.363941 | 1 |
| [Young-Gyun Seo 2019](https://pubmed.ncbi.nlm.nih.gov/?term=Seo+YG&cauthor_id=30634657) | MICT-AT VS SOC | -0.20527062 | -1.538158 | 1.127617 | 1 |
| [Young-Gyun Seo 2019](https://pubmed.ncbi.nlm.nih.gov/?term=Seo+YG&cauthor_id=30634657) | MICT-NUT VS SOC | -0.57424101 | -1.249098 | 0.1006157 | 1 |
| [Young-Gyun Seo 2019](https://pubmed.ncbi.nlm.nih.gov/?term=Seo+YG&cauthor_id=30634657) | MIIT VS SOC | -0.73064276 | -1.278736 | -0.1825498 | 1 |
| [Young-Gyun Seo 2019](https://pubmed.ncbi.nlm.nih.gov/?term=Seo+YG&cauthor_id=30634657) | MIIT-AT VS SOC | -0.4833735 | -1.303346 | 0.3365994 | 1 |
| [Young-Gyun Seo 2019](https://pubmed.ncbi.nlm.nih.gov/?term=Seo+YG&cauthor_id=30634657) | NUT VS SOC | 0.43736892 | -1.124624 | 1.999362 | 1 |
| [Fabrício Vasconcellos 2016](https://pubmed.ncbi.nlm.nih.gov/?term=Vasconcellos+F&cauthor_id=26208409) | HIIT VS SOC | -0.30445202 | -0.7780588 | 0.1691548 | 1 |
| [Fabrício Vasconcellos 2016](https://pubmed.ncbi.nlm.nih.gov/?term=Vasconcellos+F&cauthor_id=26208409) | HIIT-AT VS SOC | -0.22361457 | -1.593448 | 1.146219 | 1 |
| [Fabrício Vasconcellos 2016](https://pubmed.ncbi.nlm.nih.gov/?term=Vasconcellos+F&cauthor_id=26208409) | HIIT-MICT VS SOC | -0.33542186 | -1.70699 | 1.036146 | 1 |
| [Fabrício Vasconcellos 2016](https://pubmed.ncbi.nlm.nih.gov/?term=Vasconcellos+F&cauthor_id=26208409) | LICT VS SOC | -0.03059085 | -0.6765426 | 0.6153609 | 1 |
| [Fabrício Vasconcellos 2016](https://pubmed.ncbi.nlm.nih.gov/?term=Vasconcellos+F&cauthor_id=26208409) | LICT-NUT VS SOC | -0.28873198 | -1.345366 | 0.7679024 | 1 |
| [Fabrício Vasconcellos 2016](https://pubmed.ncbi.nlm.nih.gov/?term=Vasconcellos+F&cauthor_id=26208409) | MICT VS SOC | 1.1064256 | -0.1399045 | 2.352756 | 1 |
| [Fabrício Vasconcellos 2016](https://pubmed.ncbi.nlm.nih.gov/?term=Vasconcellos+F&cauthor_id=26208409) | MICT-AT VS SOC | -0.20527062 | -1.527611 | 1.11707 | 1 |
| [Fabrício Vasconcellos 2016](https://pubmed.ncbi.nlm.nih.gov/?term=Vasconcellos+F&cauthor_id=26208409) | MICT-NUT VS SOC | -0.57425746 | -1.242106 | 0.0935909 | 1 |
| [Fabrício Vasconcellos 2016](https://pubmed.ncbi.nlm.nih.gov/?term=Vasconcellos+F&cauthor_id=26208409) | MIIT VS SOC | -0.66735843 | -1.163801 | -0.1709156 | 1 |
| [Fabrício Vasconcellos 2016](https://pubmed.ncbi.nlm.nih.gov/?term=Vasconcellos+F&cauthor_id=26208409) | MIIT-AT VS SOC | -0.47647212 | -1.288622 | 0.3356779 | 1 |
| [Fabrício Vasconcellos 2016](https://pubmed.ncbi.nlm.nih.gov/?term=Vasconcellos+F&cauthor_id=26208409) | NUT VS SOC | 0.43732777 | -1.108134 | 1.98279 | 1 |
| [Patricia C H Wong 2008](https://pubmed.ncbi.nlm.nih.gov/?term=Wong+PC&cauthor_id=18461212) | HIIT VS SOC | -0.30895605 | -0.7690946 | 0.1511825 | 1 |
| [Patricia C H Wong 2008](https://pubmed.ncbi.nlm.nih.gov/?term=Wong+PC&cauthor_id=18461212) | HIIT-AT VS SOC | -0.22361457 | -1.561668 | 1.114438 | 1 |
| [Patricia C H Wong 2008](https://pubmed.ncbi.nlm.nih.gov/?term=Wong+PC&cauthor_id=18461212) | HIIT-MICT VS SOC | -0.33542185 | -1.67525 | 1.004406 | 1 |
| [Patricia C H Wong 2008](https://pubmed.ncbi.nlm.nih.gov/?term=Wong+PC&cauthor_id=18461212) | LICT VS SOC | -0.05635989 | -0.6244103 | 0.5116905 | 1 |
| [Patricia C H Wong 2008](https://pubmed.ncbi.nlm.nih.gov/?term=Wong+PC&cauthor_id=18461212) | LICT-NUT VS SOC | -0.28887113 | -1.310963 | 0.7332208 | 1 |
| [Patricia C H Wong 2008](https://pubmed.ncbi.nlm.nih.gov/?term=Wong+PC&cauthor_id=18461212) | MICT VS SOC | 1.1064256 | -0.1048877 | 2.317739 | 1 |
| [Patricia C H Wong 2008](https://pubmed.ncbi.nlm.nih.gov/?term=Wong+PC&cauthor_id=18461212) | MICT-AT VS SOC | -0.57431491 | -1.220135 | 0.0715052 | 1 |
| [Patricia C H Wong 2008](https://pubmed.ncbi.nlm.nih.gov/?term=Wong+PC&cauthor_id=18461212) | MICT-NUT VS SOC | -0.66586019 | -1.146527 | -0.1851935 | 1 |
| [Patricia C H Wong 2008](https://pubmed.ncbi.nlm.nih.gov/?term=Wong+PC&cauthor_id=18461212) | MIIT VS SOC | -0.47796657 | -1.26643 | 0.3104969 | 1 |
| [Patricia C H Wong 2008](https://pubmed.ncbi.nlm.nih.gov/?term=Wong+PC&cauthor_id=18461212) | MIIT-AT VS SOC | 0.43718863 | -1.056327 | 1.930704 | 1 |
| [Hong-Jie Yu 2020](https://pubmed.ncbi.nlm.nih.gov/?term=Yu+HJ&cauthor_id=31936767) | HIIT VS SOC | -0.30783064 | -0.787734 | 0.1720727 | 1 |
| [Hong-Jie Yu 2020](https://pubmed.ncbi.nlm.nih.gov/?term=Yu+HJ&cauthor_id=31936767) | HIIT-AT VS SOC | -0.22361457 | -1.612568 | 1.165339 | 1 |
| [Hong-Jie Yu 2020](https://pubmed.ncbi.nlm.nih.gov/?term=Yu+HJ&cauthor_id=31936767) | HIIT-MICT VS SOC | -0.33542185 | -1.726085 | 1.055242 | 1 |
| [Hong-Jie Yu 2020](https://pubmed.ncbi.nlm.nih.gov/?term=Yu+HJ&cauthor_id=31936767) | LICT VS SOC | -0.06531282 | -0.6601909 | 0.5295653 | 1 |
| [Hong-Jie Yu 2020](https://pubmed.ncbi.nlm.nih.gov/?term=Yu+HJ&cauthor_id=31936767) | LICT-NUT VS SOC | -0.27419862 | -1.384653 | 0.8362563 | 1 |
| [Hong-Jie Yu 2020](https://pubmed.ncbi.nlm.nih.gov/?term=Yu+HJ&cauthor_id=31936767) | MICT VS SOC | 1.1064256 | -0.1608887 | 2.37374 | 1 |
| [Hong-Jie Yu 2020](https://pubmed.ncbi.nlm.nih.gov/?term=Yu+HJ&cauthor_id=31936767) | MICT-AT VS SOC | -0.20527062 | -1.547407 | 1.136866 | 1 |
| [Hong-Jie Yu 2020](https://pubmed.ncbi.nlm.nih.gov/?term=Yu+HJ&cauthor_id=31936767) | MICT-NUT VS SOC | -0.54623699 | -1.404141 | 0.311667 | 1 |
| [Hong-Jie Yu 2020](https://pubmed.ncbi.nlm.nih.gov/?term=Yu+HJ&cauthor_id=31936767) | MIIT VS SOC | -0.67546922 | -1.177917 | -0.1730219 | 1 |
| [Hong-Jie Yu 2020](https://pubmed.ncbi.nlm.nih.gov/?term=Yu+HJ&cauthor_id=31936767) | MIIT-AT VS SOC | -0.47728429 | -1.303537 | 0.348968 | 1 |
| [Hong-Jie Yu 2020](https://pubmed.ncbi.nlm.nih.gov/?term=Yu+HJ&cauthor_id=31936767) | NUT VS SOC | 0.4518611 | -1.147461 | 2.051183 | 1 |
| [Chongwen Zuo 2023](https://pubmed.ncbi.nlm.nih.gov/?term=Zuo+C&cauthor_id=37946224) | HIIT VS SOC | -0.28588282 | -0.7786006 | 0.206835 | 1 |
| [Chongwen Zuo 2023](https://pubmed.ncbi.nlm.nih.gov/?term=Zuo+C&cauthor_id=37946224) | HIIT-AT VS SOC | -0.22361457 | -1.605211 | 1.157982 | 1 |
| [Chongwen Zuo 2023](https://pubmed.ncbi.nlm.nih.gov/?term=Zuo+C&cauthor_id=37946224) | HIIT-MICT VS SOC | -0.33542185 | -1.718737 | 1.047893 | 1 |
| [Chongwen Zuo 2023](https://pubmed.ncbi.nlm.nih.gov/?term=Zuo+C&cauthor_id=37946224) | LICT VS SOC | -0.06553783 | -0.6566307 | 0.5255551 | 1 |
| [Chongwen Zuo 2023](https://pubmed.ncbi.nlm.nih.gov/?term=Zuo+C&cauthor_id=37946224) | LICT-NUT VS SOC | -0.28868459 | -1.358019 | 0.7806497 | 1 |
| [Chongwen Zuo 2023](https://pubmed.ncbi.nlm.nih.gov/?term=Zuo+C&cauthor_id=37946224) | MICT VS SOC | 1.1064256 | -0.152821 | 2.365672 | 1 |
| [Chongwen Zuo 2023](https://pubmed.ncbi.nlm.nih.gov/?term=Zuo+C&cauthor_id=37946224) | MICT-AT VS SOC | -0.20527062 | -1.539792 | 1.129251 | 1 |
| [Chongwen Zuo 2023](https://pubmed.ncbi.nlm.nih.gov/?term=Zuo+C&cauthor_id=37946224) | MICT-NUT VS SOC | -0.57423854 | -1.250179 | 0.101702 | 1 |
| [Chongwen Zuo 2023](https://pubmed.ncbi.nlm.nih.gov/?term=Zuo+C&cauthor_id=37946224) | MIIT VS SOC | -0.69875675 | -1.218071 | -0.179442 | 1 |
| [Chongwen Zuo 2023](https://pubmed.ncbi.nlm.nih.gov/?term=Zuo+C&cauthor_id=37946224) | MIIT-AT VS SOC | -0.47119773 | -1.292741 | 0.3503456 | 1 |
| [Chongwen Zuo 2023](https://pubmed.ncbi.nlm.nih.gov/?term=Zuo+C&cauthor_id=37946224) | NUT VS SOC | 0.43737515 | -1.127174 | 2.001925 | 1 |
| [Yuhang Gao 2025](https://pubmed.ncbi.nlm.nih.gov/?term=Gao+Y&cauthor_id=41006526) | HIIT VS SOC | -0.30895605 | -0.7690946 | 0.1511825 | 1 |
| [Yuhang Gao 2025](https://pubmed.ncbi.nlm.nih.gov/?term=Gao+Y&cauthor_id=41006526) | HIIT-AT VS SOC | -0.05635989 | -0.62441 | 0.5116902 | 1 |
| [Yuhang Gao 2025](https://pubmed.ncbi.nlm.nih.gov/?term=Gao+Y&cauthor_id=41006526) | HIIT-MICT VS SOC | -0.28887113 | -1.310963 | 0.7332209 | 1 |
| [Yuhang Gao 2025](https://pubmed.ncbi.nlm.nih.gov/?term=Gao+Y&cauthor_id=41006526) | LICT VS SOC | 1.1064256 | -0.1048878 | 2.317739 | 1 |
| [Yuhang Gao 2025](https://pubmed.ncbi.nlm.nih.gov/?term=Gao+Y&cauthor_id=41006526) | LICT-NUT VS SOC | -0.20527062 | -1.49466 | 1.084119 | 1 |
| [Yuhang Gao 2025](https://pubmed.ncbi.nlm.nih.gov/?term=Gao+Y&cauthor_id=41006526) | MICT VS SOC | -0.57431491 | -1.220135 | 0.0715052 | 1 |
| [Yuhang Gao 2025](https://pubmed.ncbi.nlm.nih.gov/?term=Gao+Y&cauthor_id=41006526) | MICT-AT VS SOC | -0.66586018 | -1.146526 | -0.1851939 | 1 |
| [Yuhang Gao 2025](https://pubmed.ncbi.nlm.nih.gov/?term=Gao+Y&cauthor_id=41006526) | MICT-NUT VS SOC | -0.47796657 | -1.26643 | 0.3104969 | 1 |
| [Yuhang Gao 2025](https://pubmed.ncbi.nlm.nih.gov/?term=Gao+Y&cauthor_id=41006526) | MIIT VS SOC | 0.43718863 | -1.056327 | 1.930704 | 1 |
| Shitong Shao 2025 | HIIT VS SOC | -0.34392002 | -0.8124631 | 0.1246231 | 1 |
| Shitong Shao 2025 | HIIT-AT VS SOC | -0.22361457 | -1.563315 | 1.116086 | 1 |
| Shitong Shao 2025 | HIIT-MICT VS SOC | -0.33542185 | -1.676895 | 1.006052 | 1 |
| Shitong Shao 2025 | LICT VS SOC | 0.15255779 | -0.5395138 | 0.8446294 | 1 |
| Shitong Shao 2025 | LICT-NUT VS SOC | -0.28886348 | -1.312755 | 0.7350285 | 1 |
| Shitong Shao 2025 | MICT VS SOC | 1.1064256 | -0.1067076 | 2.319559 | 1 |
| Shitong Shao 2025 | MICT-AT VS SOC | -0.20527062 | -1.49637 | 1.085828 | 1 |
| Shitong Shao 2025 | MICT-NUT VS SOC | -0.57431168 | -1.22128 | 0.0726569 | 1 |
| Shitong Shao 2025 | MIIT VS SOC | -0.80154335 | -1.342252 | -0.2608343 | 1 |
| Shitong Shao 2025 | MIIT-AT VS SOC | -0.487713 | -1.277767 | 0.3023413 | 1 |
| Shitong Shao 2025 | NUT VS SOC | 0.43719628 | -1.059027 | 1.93342 | 1 |
| Ting Liao 2024 | HIIT VS SOC | -0.2889347 | -0.7805255 | 0.2026561 | 1 |
| Ting Liao 2024 | HIIT-AT VS SOC | -0.22361457 | -1.604321 | 1.157091 | 1 |
| Ting Liao 2024 | HIIT-MICT VS SOC | -0.33542185 | -1.717848 | 1.047004 | 1 |
| Ting Liao 2024 | LICT VS SOC | -0.06519459 | -0.6558442 | 0.525455 | 1 |
| Ting Liao 2024 | LICT-NUT VS SOC | -0.28868811 | -1.357063 | 0.7796866 | 1 |
| Ting Liao 2024 | MICT VS SOC | 1.1064256 | -0.1518443 | 2.364696 | 1 |
| Ting Liao 2024 | MICT-AT VS SOC | -0.20527062 | -1.53887 | 1.128329 | 1 |
| Ting Liao 2024 | MICT-NUT VS SOC | -0.57423993 | -1.249569 | 0.1010893 | 1 |
| Ting Liao 2024 | MIIT VS SOC | -0.69521513 | -1.213299 | -0.1771315 | 1 |
| Ting Liao 2024 | MIIT-AT VS SOC | -0.47205732 | -1.292906 | 0.3487914 | 1 |
| Ting Liao 2024 | NUT VS SOC | 0.43737164 | -1.125736 | 2.000479 | 1 |
| [Neiva Leite 2024](https://link.springer.com/article/10.1007/s00431-024-05734-w#auth-Neiva-Leite-Aff1-Aff2) | HIIT VS SOC | -0.31958228 | -0.8330698 | 0.1939052 | 1 |
| [Neiva Leite 2024](https://link.springer.com/article/10.1007/s00431-024-05734-w#auth-Neiva-Leite-Aff1-Aff2) | HIIT-AT VS SOC | -0.22361457 | -1.601797 | 1.154568 | 1 |
| [Neiva Leite 2024](https://link.springer.com/article/10.1007/s00431-024-05734-w#auth-Neiva-Leite-Aff1-Aff2) | HIIT-MICT VS SOC | -0.33542185 | -1.715328 | 1.044484 | 1 |
| [Neiva Leite 2024](https://link.springer.com/article/10.1007/s00431-024-05734-w#auth-Neiva-Leite-Aff1-Aff2) | LICT VS SOC | -0.06563713 | -0.6559699 | 0.5246957 | 1 |
| [Neiva Leite 2024](https://link.springer.com/article/10.1007/s00431-024-05734-w#auth-Neiva-Leite-Aff1-Aff2) | LICT-NUT VS SOC | -0.28869813 | -1.354351 | 0.7769549 | 1 |
| [Neiva Leite 2024](https://link.springer.com/article/10.1007/s00431-024-05734-w#auth-Neiva-Leite-Aff1-Aff2) | MICT VS SOC | 1.1064256 | -0.1490747 | 2.361926 | 1 |
| [Neiva Leite 2024](https://link.springer.com/article/10.1007/s00431-024-05734-w#auth-Neiva-Leite-Aff1-Aff2) | MICT-AT VS SOC | -0.20527062 | -1.536257 | 1.125716 | 1 |
| [Neiva Leite 2024](https://link.springer.com/article/10.1007/s00431-024-05734-w#auth-Neiva-Leite-Aff1-Aff2) | MICT-NUT VS SOC | -0.57424391 | -1.247839 | 0.0993513 | 1 |
| [Neiva Leite 2024](https://link.springer.com/article/10.1007/s00431-024-05734-w#auth-Neiva-Leite-Aff1-Aff2) | MIIT VS SOC | -0.6781412 | -1.182019 | -0.174263 | 1 |
| [Neiva Leite 2024](https://link.springer.com/article/10.1007/s00431-024-05734-w#auth-Neiva-Leite-Aff1-Aff2) | MIIT-AT VS SOC | -0.48064308 | -1.300712 | 0.3394263 | 1 |
| [Neiva Leite 2024](https://link.springer.com/article/10.1007/s00431-024-05734-w#auth-Neiva-Leite-Aff1-Aff2) | NUT VS SOC | 0.43736162 | -1.121655 | 1.996379 | 1 |
| Noelia González-Gálvez 2024 | HIIT VS SOC | -0.4299142 | -0.83707 | -0.0227584 | 1 |
| Noelia González-Gálvez 2024 | HIIT-AT VS SOC | -0.22361457 | -1.367876 | 0.920647 | 1 |
| Noelia González-Gálvez 2024 | HIIT-MICT VS SOC | -0.33542185 | -1.481759 | 0.8109149 | 1 |
| Noelia González-Gálvez 2024 | LICT VS SOC | 0.01330526 | -0.4496096 | 0.4762202 | 1 |
| Noelia González-Gálvez 2024 | LICT-NUT VS SOC | -0.29032066 | -1.092132 | 0.511491 | 1 |
| Noelia González-Gálvez 2024 | MICT VS SOC | 1.1064256 | 0.1133351 | 2.099516 | 1 |
| Noelia González-Gálvez 2024 | MICT-AT VS SOC | -0.20527062 | -1.292227 | 0.8816854 | 1 |
| Noelia González-Gálvez 2024 | MICT-NUT VS SOC | -0.57505688 | -1.079324 | -0.0707902 | 1 |
| Noelia González-Gálvez 2024 | MIIT VS SOC | -0.46165222 | -0.8681929 | -0.0551116 | 1 |
| Noelia González-Gálvez 2024 | MIIT-AT VS SOC | -0.51342859 | -1.154938 | 0.1280807 | 1 |
| Noelia González-Gálvez 2024 | NUT VS SOC | 0.43573914 | -0.7251962 | 1.596674 | 1 |
| [Emir Tas 2023](https://onlinelibrary.wiley.com/authored-by/Tas/Emir) | HIIT VS SOC | -0.35015625 | -0.8513561 | 0.1510436 | 1 |
| [Emir Tas 2023](https://onlinelibrary.wiley.com/authored-by/Tas/Emir) | HIIT-AT VS SOC | -0.22361457 | -1.588039 | 1.14081 | 1 |
| [Emir Tas 2023](https://onlinelibrary.wiley.com/authored-by/Tas/Emir) | HIIT-MICT VS SOC | -0.33542185 | -1.701587 | 1.030744 | 1 |
| [Emir Tas 2023](https://onlinelibrary.wiley.com/authored-by/Tas/Emir) | LICT VS SOC | -0.06877433 | -0.6519113 | 0.5143626 | 1 |
| [Emir Tas 2023](https://onlinelibrary.wiley.com/authored-by/Tas/Emir) | LICT-NUT VS SOC | -0.28875447 | -1.339534 | 0.7620249 | 1 |
| [Emir Tas 2023](https://onlinelibrary.wiley.com/authored-by/Tas/Emir) | MICT VS SOC | 1.1064256 | -0.1339571 | 2.346808 | 1 |
| [Emir Tas 2023](https://onlinelibrary.wiley.com/authored-by/Tas/Emir) | MICT-AT VS SOC | -0.20527062 | -1.522007 | 1.111465 | 1 |
| [Emir Tas 2023](https://onlinelibrary.wiley.com/authored-by/Tas/Emir) | MICT-NUT VS SOC | -0.57426656 | -1.238383 | 0.0898499 | 1 |
| [Emir Tas 2023](https://onlinelibrary.wiley.com/authored-by/Tas/Emir) | MIIT VS SOC | -0.68771033 | -1.185075 | -0.1903459 | 1 |
| [Emir Tas 2023](https://onlinelibrary.wiley.com/authored-by/Tas/Emir) | MIIT-AT VS SOC | -0.48928114 | -1.298809 | 0.3202472 | 1 |
| [Emir Tas 2023](https://onlinelibrary.wiley.com/authored-by/Tas/Emir) | NUT VS SOC | 0.43730528 | -1.099355 | 1.973965 | 1 |
| [Ghazi Racil 2024](https://www.tandfonline.com/author/Racil,+Ghazi) | HIIT VS SOC | -0.25127362 | -0.7714269 | 0.2688797 | 1 |
| [Ghazi Racil 2024](https://www.tandfonline.com/author/Racil,+Ghazi) | HIIT-AT VS SOC | -0.22361458 | -1.628183 | 1.180954 | 1 |
| [Ghazi Racil 2024](https://www.tandfonline.com/author/Racil,+Ghazi) | HIIT-MICT VS SOC | -0.33542187 | -1.741681 | 1.070837 | 1 |
| [Ghazi Racil 2024](https://www.tandfonline.com/author/Racil,+Ghazi) | LICT VS SOC | -0.06599682 | -0.6708291 | 0.5388355 | 1 |
| [Ghazi Racil 2024](https://www.tandfonline.com/author/Racil,+Ghazi) | LICT-NUT VS SOC | -0.28859779 | -1.382612 | 0.8054168 | 1 |
| [Ghazi Racil 2024](https://www.tandfonline.com/author/Racil,+Ghazi) | MICT VS SOC | 1.1064256 | -0.1779831 | 2.390834 | 1 |
| [Ghazi Racil 2024](https://www.tandfonline.com/author/Racil,+Ghazi) | MICT-AT VS SOC | -0.20527062 | -1.56356 | 1.153019 | 1 |
| [Ghazi Racil 2024](https://www.tandfonline.com/author/Racil,+Ghazi) | MICT-NUT VS SOC | -0.57420474 | -1.265862 | 0.1174523 | 1 |
| [Ghazi Racil 2024](https://www.tandfonline.com/author/Racil,+Ghazi) | MIIT VS SOC | -0.7080389 | -1.258392 | -0.1576855 | 1 |
| [Ghazi Racil 2024](https://www.tandfonline.com/author/Racil,+Ghazi) | MIIT-AT VS SOC | -0.46135497 | -1.300745 | 0.3780347 | 1 |
| [Ghazi Racil 2024](https://www.tandfonline.com/author/Racil,+Ghazi) | NUT VS SOC | 0.43746193 | -1.164165 | 2.039089 | 1 |

**Table S23** Sensitivity Analysis Using MAS as the Outcome Measure.

| **dropped_id** | **comparison** | **eff** | **lci** | **uci** | **connected** |
| --- | --- | --- | --- | --- | --- |
| [Wissal Abassi 2020](https://pubmed.ncbi.nlm.nih.gov/?term=Abassi+W&cauthor_id=33581014) | HIIT VS SOC | 1.2626625 | 0.8739721 | 1.651353 | 1 |
| [Wissal Abassi 2020](https://pubmed.ncbi.nlm.nih.gov/?term=Abassi+W&cauthor_id=33581014) | HIIT-AT VS SOC | 1.242539 | 0.6937947 | 1.791283 | 1 |
| [Wissal Abassi 2020](https://pubmed.ncbi.nlm.nih.gov/?term=Abassi+W&cauthor_id=33581014) | MICT VS SOC | 0.79332508 | 0.2618567 | 1.324793 | 1 |
| [Wissal Abassi 2020](https://pubmed.ncbi.nlm.nih.gov/?term=Abassi+W&cauthor_id=33581014) | MIIT VS SOC | 0.62943444 | 0.0726317 | 1.186237 | 1 |
| [Wissal Abassi 2022](https://pubmed.ncbi.nlm.nih.gov/?term=Abassi+W&cauthor_id=34749418) | HIIT VS SOC | 1.3078389 | 0.9011702 | 1.714508 | 1 |
| [Wissal Abassi 2022](https://pubmed.ncbi.nlm.nih.gov/?term=Abassi+W&cauthor_id=34749418) | HIIT-AT VS SOC | 1.269484 | 0.7161242 | 1.822844 | 1 |
| [Wissal Abassi 2022](https://pubmed.ncbi.nlm.nih.gov/?term=Abassi+W&cauthor_id=34749418) | MICT VS SOC | 1.0619522 | 0.3060997 | 1.817805 | 1 |
| [Wissal Abassi 2022](https://pubmed.ncbi.nlm.nih.gov/?term=Abassi+W&cauthor_id=34749418) | MIIT VS SOC | 0.67062878 | 0.1685577 | 1.1727 | 1 |
| [G Racil 2013](https://pubmed.ncbi.nlm.nih.gov/?term=Racil+G&cauthor_id=23824463) | HIIT VS SOC | 1.4405824 | 1.032085 | 1.84908 | 1 |
| [G Racil 2013](https://pubmed.ncbi.nlm.nih.gov/?term=Racil+G&cauthor_id=23824463) | HIIT-AT VS SOC | 1.3486576 | 0.7948188 | 1.902496 | 1 |
| [G Racil 2013](https://pubmed.ncbi.nlm.nih.gov/?term=Racil+G&cauthor_id=23824463) | MICT VS SOC | 0.67504236 | -0.0270657 | 1.37715 | 1 |
| [G Racil 2013](https://pubmed.ncbi.nlm.nih.gov/?term=Racil+G&cauthor_id=23824463) | MIIT VS SOC | 0.76523162 | 0.2624071 | 1.268056 | 1 |
| [Ghazi Racil 2015](https://pubmed.ncbi.nlm.nih.gov/?term=Racil+G&cauthor_id=26701117) | HIIT VS SOC | 1.4409722 | 0.9905018 | 1.891443 | 1 |
| [Ghazi Racil 2015](https://pubmed.ncbi.nlm.nih.gov/?term=Racil+G&cauthor_id=26701117) | HIIT-AT VS SOC | 0.88493922 | 0.3407478 | 1.429131 | 1 |
| [Ghazi Racil 2015](https://pubmed.ncbi.nlm.nih.gov/?term=Racil+G&cauthor_id=26701117) | MICT VS SOC | 0.76550931 | 0.2447943 | 1.286224 | 1 |
| [Chongwen Zuo 2023](https://pubmed.ncbi.nlm.nih.gov/?term=Zuo+C&cauthor_id=37946224) | MIIT VS SOC | 1.3964238 | 1.01801 | 1.774838 | 1 |
| [Chongwen Zuo 2023](https://pubmed.ncbi.nlm.nih.gov/?term=Zuo+C&cauthor_id=37946224) | HIIT VS SOC | 1.3223196 | 0.7761365 | 1.868503 | 1 |
| [Chongwen Zuo 2023](https://pubmed.ncbi.nlm.nih.gov/?term=Zuo+C&cauthor_id=37946224) | HIIT-AT VS SOC | 0.86205062 | 0.3325436 | 1.391558 | 1 |
| [Chongwen Zuo 2023](https://pubmed.ncbi.nlm.nih.gov/?term=Zuo+C&cauthor_id=37946224) | MICT VS SOC | 0.60264843 | 0.0303212 | 1.174976 | 1 |
| [Ghazi Racil 2024](https://www.tandfonline.com/author/Racil,+Ghazi) | HIIT VS SOC | 1.3904942 | 0.9798781 | 1.80111 | 1 |
| [Ghazi Racil 2024](https://www.tandfonline.com/author/Racil,+Ghazi) | HIIT-AT VS SOC | 1.3187829 | 0.7643871 | 1.873179 | 1 |
| [Ghazi Racil 2024](https://www.tandfonline.com/author/Racil,+Ghazi) | MICT VS SOC | 0.85900404 | 0.3232009 | 1.394807 | 1 |
| [Ghazi Racil 2024](https://www.tandfonline.com/author/Racil,+Ghazi) | MIIT VS SOC | 0.84470268 | 0.2358984 | 1.453507 | 1 |

**Table S24** Sensitivity Analysis Using RHR as the Outcome Measure.

| **dropped_id** | **comparison** | **eff** | **lci** | **uci** | **connected** |
| --- | --- | --- | --- | --- | --- |
| [Napasakorn Chuensiri 2018](https://pubmed.ncbi.nlm.nih.gov/?term=Chuensiri+N&cauthor_id=29099231) | HIIT VS SOC | -0.86622128 | -1.544252 | -0.1881908 | 1 |
| [Napasakorn Chuensiri 2018](https://pubmed.ncbi.nlm.nih.gov/?term=Chuensiri+N&cauthor_id=29099231) | HIIT-NUT VS SOC | -1.7473817 | -2.878197 | -0.6165665 | 1 |
| [Napasakorn Chuensiri 2018](https://pubmed.ncbi.nlm.nih.gov/?term=Chuensiri+N&cauthor_id=29099231) | LICT VS SOC | -0.69297266 | -1.408247 | 0.0223016 | 1 |
| [Napasakorn Chuensiri 2018](https://pubmed.ncbi.nlm.nih.gov/?term=Chuensiri+N&cauthor_id=29099231) | LICT-NUT VS SOC | 0.10621183 | -0.8487934 | 1.061217 | 1 |
| [Napasakorn Chuensiri 2018](https://pubmed.ncbi.nlm.nih.gov/?term=Chuensiri+N&cauthor_id=29099231) | MICT VS SOC | -0.50931592 | -1.468259 | 0.4496267 | 1 |
| [Napasakorn Chuensiri 2018](https://pubmed.ncbi.nlm.nih.gov/?term=Chuensiri+N&cauthor_id=29099231) | MICT-AT VS SOC | -2.1632326 | -3.483729 | -0.8427362 | 1 |
| [Napasakorn Chuensiri 2018](https://pubmed.ncbi.nlm.nih.gov/?term=Chuensiri+N&cauthor_id=29099231) | MICT-NUT VS SOC | 0.42484733 | -0.524709 | 1.374404 | 1 |
| [Napasakorn Chuensiri 2018](https://pubmed.ncbi.nlm.nih.gov/?term=Chuensiri+N&cauthor_id=29099231) | MIIT VS SOC | -0.43580288 | -1.19069 | 0.3190844 | 1 |
| [N Cvetković 2018](https://pubmed.ncbi.nlm.nih.gov/?term=Cvetkovi%C4%87+N&cauthor_id=29979479) | HIIT VS SOC | -0.60351916 | -1.231288 | 0.0242499 | 1 |
| [N Cvetković 2018](https://pubmed.ncbi.nlm.nih.gov/?term=Cvetkovi%C4%87+N&cauthor_id=29979479) | HIIT-NUT VS SOC | -1.483042 | -2.527972 | -0.4381115 | 1 |
| [N Cvetković 2018](https://pubmed.ncbi.nlm.nih.gov/?term=Cvetkovi%C4%87+N&cauthor_id=29979479) | LICT VS SOC | -0.55541042 | -1.369799 | 0.2589785 | 1 |
| [N Cvetković 2018](https://pubmed.ncbi.nlm.nih.gov/?term=Cvetkovi%C4%87+N&cauthor_id=29979479) | LICT-NUT VS SOC | 0.10621183 | -0.774076 | 0.9864997 | 1 |
| [N Cvetković 2018](https://pubmed.ncbi.nlm.nih.gov/?term=Cvetkovi%C4%87+N&cauthor_id=29979479) | MICT VS SOC | -0.24789305 | -1.134684 | 0.6388977 | 1 |
| [N Cvetković 2018](https://pubmed.ncbi.nlm.nih.gov/?term=Cvetkovi%C4%87+N&cauthor_id=29979479) | MICT-AT VS SOC | -2.1632326 | -3.430743 | -0.8957224 | 1 |
| [N Cvetković 2018](https://pubmed.ncbi.nlm.nih.gov/?term=Cvetkovi%C4%87+N&cauthor_id=29979479) | MICT-NUT VS SOC | 0.42484733 | -0.449526 | 1.299221 | 1 |
| [N Cvetković 2018](https://pubmed.ncbi.nlm.nih.gov/?term=Cvetkovi%C4%87+N&cauthor_id=29979479) | MIIT VS SOC | -0.41984983 | -1.035443 | 0.1957432 | 1 |
| [Meng Cao 2022](https://pubmed.ncbi.nlm.nih.gov/?term=Cao+M&cauthor_id=36143083) | HIIT VS SOC | -0.8749594 | -1.55803 | -0.1918886 | 1 |
| [Meng Cao 2022](https://pubmed.ncbi.nlm.nih.gov/?term=Cao+M&cauthor_id=36143083) | HIIT-NUT VS SOC | -1.7553417 | -2.861487 | -0.6491967 | 1 |
| [Meng Cao 2022](https://pubmed.ncbi.nlm.nih.gov/?term=Cao+M&cauthor_id=36143083) | LICT VS SOC | -0.73651805 | -1.421973 | -0.0510631 | 1 |
| [Meng Cao 2022](https://pubmed.ncbi.nlm.nih.gov/?term=Cao+M&cauthor_id=36143083) | LICT-NUT VS SOC | 0.10621183 | -0.8110533 | 1.023477 | 1 |
| [Meng Cao 2022](https://pubmed.ncbi.nlm.nih.gov/?term=Cao+M&cauthor_id=36143083) | MICT VS SOC | -0.51866163 | -1.462723 | 0.4253994 | 1 |
| [Meng Cao 2022](https://pubmed.ncbi.nlm.nih.gov/?term=Cao+M&cauthor_id=36143083) | MICT-AT VS SOC | -2.1632326 | -3.456697 | -0.869768 | 1 |
| [Meng Cao 2022](https://pubmed.ncbi.nlm.nih.gov/?term=Cao+M&cauthor_id=36143083) | MICT-NUT VS SOC | 0.42484733 | -0.4867433 | 1.336438 | 1 |
| [Meng Cao 2022](https://pubmed.ncbi.nlm.nih.gov/?term=Cao+M&cauthor_id=36143083) | MIIT VS SOC | -0.57648633 | -1.221214 | 0.068241 | 1 |
| [Vandana Jain 2022](https://pubmed.ncbi.nlm.nih.gov/?term=Jain+V&cauthor_id=36124513) | HIIT VS SOC | -0.77477142 | -1.306159 | -0.2433836 | 1 |
| [Vandana Jain 2022](https://pubmed.ncbi.nlm.nih.gov/?term=Jain+V&cauthor_id=36124513) | HIIT-NUT VS SOC | -1.6534373 | -2.618618 | -0.688257 | 1 |
| [Vandana Jain 2022](https://pubmed.ncbi.nlm.nih.gov/?term=Jain+V&cauthor_id=36124513) | LICT VS SOC | -0.69662393 | -1.324822 | -0.0684261 | 1 |
| [Vandana Jain 2022](https://pubmed.ncbi.nlm.nih.gov/?term=Jain+V&cauthor_id=36124513) | LICT-NUT VS SOC | -0.41981421 | -1.223253 | 0.3836251 | 1 |
| [Vandana Jain 2022](https://pubmed.ncbi.nlm.nih.gov/?term=Jain+V&cauthor_id=36124513) | MICT VS SOC | -2.1632326 | -3.40819 | -0.9182751 | 1 |
| [Vandana Jain 2022](https://pubmed.ncbi.nlm.nih.gov/?term=Jain+V&cauthor_id=36124513) | MICT-AT VS SOC | -0.52228129 | -1.097642 | 0.0530792 | 1 |
| [Patricia C H Wong 2008](https://pubmed.ncbi.nlm.nih.gov/?term=Wong+PC&cauthor_id=18461212) | HIIT VS SOC | -0.7747714 | -1.306159 | -0.2433837 | 1 |
| [Patricia C H Wong 2008](https://pubmed.ncbi.nlm.nih.gov/?term=Wong+PC&cauthor_id=18461212) | HIIT-NUT VS SOC | -1.6534373 | -2.618617 | -0.6882572 | 1 |
| [Patricia C H Wong 2008](https://pubmed.ncbi.nlm.nih.gov/?term=Wong+PC&cauthor_id=18461212) | LICT VS SOC | -0.69662391 | -1.324822 | -0.0684262 | 1 |
| [Patricia C H Wong 2008](https://pubmed.ncbi.nlm.nih.gov/?term=Wong+PC&cauthor_id=18461212) | LICT-NUT VS SOC | 0.10621183 | -0.7412809 | 0.9537045 | 1 |
| [Patricia C H Wong 2008](https://pubmed.ncbi.nlm.nih.gov/?term=Wong+PC&cauthor_id=18461212) | MICT VS SOC | -0.4198142 | -1.223253 | 0.3836251 | 1 |
| [Patricia C H Wong 2008](https://pubmed.ncbi.nlm.nih.gov/?term=Wong+PC&cauthor_id=18461212) | MICT-AT VS SOC | 0.42484733 | -0.4165004 | 1.266195 | 1 |
| [Patricia C H Wong 2008](https://pubmed.ncbi.nlm.nih.gov/?term=Wong+PC&cauthor_id=18461212) | MICT-NUT VS SOC | -0.52228127 | -1.097641 | 0.053079 | 1 |
| [C Y Rodriguez-Triviño 2025](https://pubmed.ncbi.nlm.nih.gov/?term=Rodriguez-Trivi%C3%B1o+CY&cauthor_id=40531756) | HIIT VS SOC | -0.73321397 | -1.108903 | -0.3575252 | 1 |
| [C Y Rodriguez-Triviño 2025](https://pubmed.ncbi.nlm.nih.gov/?term=Rodriguez-Trivi%C3%B1o+CY&cauthor_id=40531756) | HIIT-NUT VS SOC | -1.3525924 | -1.98977 | -0.7154153 | 1 |
| [C Y Rodriguez-Triviño 2025](https://pubmed.ncbi.nlm.nih.gov/?term=Rodriguez-Trivi%C3%B1o+CY&cauthor_id=40531756) | LICT VS SOC | -0.64001333 | -1.042661 | -0.2373657 | 1 |
| [C Y Rodriguez-Triviño 2025](https://pubmed.ncbi.nlm.nih.gov/?term=Rodriguez-Trivi%C3%B1o+CY&cauthor_id=40531756) | LICT-NUT VS SOC | 0.10621183 | -0.3960474 | 0.608471 | 1 |
| [C Y Rodriguez-Triviño 2025](https://pubmed.ncbi.nlm.nih.gov/?term=Rodriguez-Trivi%C3%B1o+CY&cauthor_id=40531756) | MICT VS SOC | 0.17333136 | -0.4716374 | 0.8183001 | 1 |
| [C Y Rodriguez-Triviño 2025](https://pubmed.ncbi.nlm.nih.gov/?term=Rodriguez-Trivi%C3%B1o+CY&cauthor_id=40531756) | MICT-AT VS SOC | -2.1632326 | -3.204357 | -1.122108 | 1 |
| [C Y Rodriguez-Triviño 2025](https://pubmed.ncbi.nlm.nih.gov/?term=Rodriguez-Trivi%C3%B1o+CY&cauthor_id=40531756) | MICT-NUT VS SOC | 0.42484733 | -0.0669721 | 0.9166668 | 1 |
| [C Y Rodriguez-Triviño 2025](https://pubmed.ncbi.nlm.nih.gov/?term=Rodriguez-Trivi%C3%B1o+CY&cauthor_id=40531756) | MIIT VS SOC | -0.46547518 | -0.8371261 | -0.0938242 | 1 |
| [Xinghao Wang 2025](https://pubmed.ncbi.nlm.nih.gov/?term=Wang+X&cauthor_id=41323591) | HIIT VS SOC | -0.73321391 | -1.108903 | -0.3575251 | 1 |
| [Xinghao Wang 2025](https://pubmed.ncbi.nlm.nih.gov/?term=Wang+X&cauthor_id=41323591) | HIIT-NUT VS SOC | -0.64001331 | -1.042661 | -0.2373657 | 1 |
| [Xinghao Wang 2025](https://pubmed.ncbi.nlm.nih.gov/?term=Wang+X&cauthor_id=41323591) | LICT VS SOC | 0.10621183 | -0.3960474 | 0.608471 | 1 |
| [Xinghao Wang 2025](https://pubmed.ncbi.nlm.nih.gov/?term=Wang+X&cauthor_id=41323591) | LICT-NUT VS SOC | -0.91130798 | -1.536678 | -0.2859384 | 1 |
| [Xinghao Wang 2025](https://pubmed.ncbi.nlm.nih.gov/?term=Wang+X&cauthor_id=41323591) | MICT VS SOC | -2.1632326 | -3.204357 | -1.122108 | 1 |
| [Xinghao Wang 2025](https://pubmed.ncbi.nlm.nih.gov/?term=Wang+X&cauthor_id=41323591) | MICT-AT VS SOC | 0.42484733 | -0.0669721 | 0.9166668 | 1 |
| [Xinghao Wang 2025](https://pubmed.ncbi.nlm.nih.gov/?term=Wang+X&cauthor_id=41323591) | MICT-NUT VS SOC | -0.46547515 | -0.8371261 | -0.0938242 | 1 |
| Shitong Shao 2025 | HIIT VS SOC | -0.85535077 | -1.514545 | -0.1961563 | 1 |
| Shitong Shao 2025 | HIIT-NUT VS SOC | -1.7375639 | -2.90157 | -0.5735581 | 1 |
| Shitong Shao 2025 | LICT VS SOC | -0.89113138 | -2.007293 | 0.2250301 | 1 |
| Shitong Shao 2025 | LICT-NUT VS SOC | 0.10621192 | -0.9080372 | 1.120461 | 1 |
| Shitong Shao 2025 | MICT VS SOC | -0.49762316 | -1.47397 | 0.4787235 | 1 |
| Shitong Shao 2025 | MICT-AT VS SOC | -2.1632326 | -3.527189 | -0.7992763 | 1 |
| Shitong Shao 2025 | MICT-NUT VS SOC | 0.42484742 | -0.5842727 | 1.433968 | 1 |
| Shitong Shao 2025 | MIIT VS SOC | -0.66227269 | -1.573594 | 0.2490491 | 1 |
| Ting Liao 2024 | HIIT VS SOC | -0.73906754 | -1.334943 | -0.1431918 | 1 |
| Ting Liao 2024 | HIIT-NUT VS SOC | -1.6194431 | -2.673701 | -0.5651853 | 1 |
| Ting Liao 2024 | LICT VS SOC | -0.71318831 | -1.385954 | -0.0404226 | 1 |
| Ting Liao 2024 | LICT-NUT VS SOC | 0.10621183 | -0.8107387 | 1.023162 | 1 |
| Ting Liao 2024 | MICT VS SOC | -0.3827753 | -1.265672 | 0.5001213 | 1 |
| Ting Liao 2024 | MICT-AT VS SOC | -2.1632326 | -3.456474 | -0.8699911 | 1 |
| Ting Liao 2024 | MICT-NUT VS SOC | 0.42484733 | -0.4864267 | 1.336121 | 1 |
| Ting Liao 2024 | MIIT VS SOC | -0.58524027 | -1.254705 | 0.0842249 | 1 |

**Meta-regression**

**Countries Conducting Research**

**Table S25** Meta-Regression Analysis of VO_2_peak Using Countries Conducting Research as a Moderator.

| **intervention measures** | **covariate** | **Coefficient** | **Std. err.** | **z** | **P>z** | **[95% conf.** | **interval]** |
| --- | --- | --- | --- | --- | --- | --- | --- |
| AT VS HIIT | country | -0.2361002 | 28.00959 | -0.01 | 0.993 | -55.13388 | 54.66168 |
| AT VS HIIT | _cons | 4.24985 | 308.0903 | 0.01 | 0.989 | -599.596 | 608.0957 |
| AT VS HIIT-AT | country | 0.6237048 | 28.01452 | 0.02 | 0.982 | -54.28374 | 55.53115 |
| AT VS HIIT-AT | _cons | -2.329257 | 308.1133 | -0.01 | 0.994 | -606.2202 | 601.5617 |
| AT VS LICT | country | 1.237118 | 28.01746 | 0.04 | 0.965 | -53.67609 | 56.15032 |
| AT VS LICT | _cons | -2.889491 | 308.0959 | -0.01 | 0.993 | -606.7463 | 600.9674 |
| AT VS MICT | country | -0.1642252 | 28.01027 | -0.01 | 0.995 | -55.06335 | 54.7349 |
| AT VS MICT | _cons | 2.02133 | 308.092 | 0.01 | 0.995 | -601.8278 | 605.8705 |
| AT VS MIIT | country | -0.0491471 | 28.0093 | 0 | 0.999 | -54.94637 | 54.84808 |
| AT VS MIIT | _cons | 2.037383 | 308.0909 | 0.01 | 0.995 | -601.8098 | 605.8845 |
| AT VS MIIT-AT | _cons | 0.699996 | 1.735207 | 0.4 | 0.687 | -2.700948 | 4.10094 |
| AT VS NUT | country | -1.994586 | 28.03888 | -0.07 | 0.943 | -56.94979 | 52.96061 |
| AT VS NUT | _cons | 9.355472 | 308.1959 | 0.03 | 0.976 | -594.6973 | 613.4083 |
| AT VS SOC | country | 0.1571202 | 28.00901 | 0.01 | 0.996 | -54.73954 | 55.05378 |
| AT VS SOC | _cons | -2.129503 | 308.0887 | -0.01 | 0.994 | -605.9722 | 601.7132 |

**Table S26** Meta-Regression Analysis of VO_2_max Using Countries Conducting Research as a Moderator.

| **intervention measures** | **covariate** | **Coefficient** | **Std. err.** | **z** | **P>z** | **[95% conf.** | **interval]** |
| --- | --- | --- | --- | --- | --- | --- | --- |
| HIIT VS HICT-AT | country | -0.4611429 | 22.93466 | -0.02 | 0.984 | -45.41226 | 44.48997 |
| HIIT VS HICT-AT | _cons | 4.438892 | 389.858 | 0.01 | 0.991 | -759.6688 | 768.5466 |
| HIIT-AT VS HICT-AT | _cons | 0.8666119 | 366.9235 | 0 | 0.998 | -718.2903 | 720.0235 |
| HIIT-MICT VS HICT-AT | _cons | 3.866612 | 366.9242 | 0.01 | 0.992 | -715.2915 | 723.0248 |
| LICT VS HICT-AT | country | -0.1628871 | 22.93566 | -0.01 | 0.994 | -45.11595 | 44.79018 |
| LICT VS HICT-AT | _cons | 0.2929725 | 389.857 | 0 | 0.999 | -763.8128 | 764.3987 |
| MICT VS HICT-AT | _cons | -1.724384 | 91.80261 | -0.02 | 0.985 | -181.6542 | 178.2054 |
| MICT-AT VS HICT-AT | country | -2.009148 | 23.04964 | -0.09 | 0.931 | -47.18562 | 43.16732 |
| MICT-AT VS HICT-AT | _cons | 30.14574 | 391.5698 | 0.08 | 0.939 | -737.317 | 797.6085 |
| MIIT VS HICT-AT | country | -0.1715012 | 22.93527 | -0.01 | 0.994 | -45.12381 | 44.78081 |
| MIIT VS HICT-AT | _cons | 0.8601637 | 389.8587 | 0 | 0.998 | -763.2488 | 764.9691 |
| MIIT-AT VS HICT-AT | _cons | -4.997664 | 4.08238 | -1.22 | 0.221 | -12.99898 | 3.003653 |
| SOC VS HICT-AT | country | -0.15415 | 22.93373 | -0.01 | 0.995 | -45.10344 | 44.79514 |
| SOC VS HICT-AT | _cons | -1.179224 | 389.852 | 0 | 0.998 | -765.275 | 762.9166 |

**Table S27** Meta-Regression Analysis of HR_max_ Using Countries Conducting Research as a Moderator.

| **intervention measures** | **covariate** | **Coefficient** | **Std. err.** | **z** | **P>z** | **[95% conf.** | **interval]** |
| --- | --- | --- | --- | --- | --- | --- | --- |
| LICT VS HIIT | country | -0.3672485 | 0.7190062 | -0.51 | 0.61 | -1.776475 | 1.041978 |
| LICT VS HIIT | _cons | -1.105213 | 5.947003 | -0.19 | 0.853 | -12.76113 | 10.5507 |
| MICT VS HIIT | country | 0.6224833 | 3.754729 | 0.17 | 0.868 | -6.73665 | 7.981616 |
| MICT VS HIIT | _cons | -4.524833 | 33.05712 | -0.14 | 0.891 | -69.3156 | 60.26594 |
| MIIT VS HIIT | country | 0.0533245 | 0.7432318 | 0.07 | 0.943 | -1.403383 | 1.510032 |
| MIIT VS HIIT | _cons | 0.5212365 | 4.656273 | 0.11 | 0.911 | -8.604891 | 9.647364 |
| SOC VS HIIT | country | 0.3537101 | 0.5553058 | 0.64 | 0.524 | -0.7346694 | 1.44209 |
| SOC VS HIIT | _cons | -1.9698 | 3.955875 | -0.5 | 0.619 | -9.723174 | 5.783573 |

**Table S28** Meta-Regression Analysis of SBP Using Countries Conducting Research as a Moderator.

| **intervention measures** | **covariate** | **Coefficient** | **Std. err.** | **z** | **P>z** | **[95% conf.** | **interval]** |
| --- | --- | --- | --- | --- | --- | --- | --- |
| HIIT-AT VS HIIT | _cons | 2.312366 | 5.984908 | 0.39 | 0.699 | -9.417838 | 14.04257 |
| HIIT-MICT VS HIIT | _cons | 10.51237 | 5.515806 | 1.91 | 0.057 | -0.2984162 | 21.32315 |
| LICT VS HIIT | country | -0.4044217 | 0.5313196 | -0.76 | 0.447 | -1.445789 | 0.6369455 |
| LICT VS HIIT | _cons | 2.480961 | 3.222232 | 0.77 | 0.441 | -3.834498 | 8.796419 |
| LICT-NUT VS HIIT | country | 2.281322 | 3298.563 | 0 | 0.999 | -6462.782 | 6467.345 |
| LICT-NUT VS HIIT | _cons | -34.37874 | 52776.99 | 0 | 0.999 | -103475.4 | 103406.6 |
| MICT VS HIIT | country | -0.6593193 | 0.3537901 | -1.86 | 0.062 | -1.352735 | 0.0340966 |
| MICT VS HIIT | _cons | -0.8551715 | 3.790089 | -0.23 | 0.821 | -8.283609 | 6.573266 |
| MICT-AT VS HIIT | _cons | -9.455928 | 5.521248 | -1.71 | 0.087 | -20.27738 | 1.365519 |
| MICT-NUT VS HIIT | country | -0.4244257 | 0.4159224 | -1.02 | 0.308 | -1.239619 | 0.3907671 |
| MICT-NUT VS HIIT | _cons | 2.179623 | 4.00977 | 0.54 | 0.587 | -5.679382 | 10.03863 |
| MIIT VS HIIT | country | -0.7024152 | 0.267638 | -2.62 | 0.009 | -1.226976 | -0.1778543 |
| MIIT VS HIIT | _cons | 2.46153 | 2.306246 | 1.07 | 0.286 | -2.058629 | 6.981689 |
| MIIT-AT VS HIIT | country | 1.433603 | 3.34184 | 0.43 | 0.668 | -5.116282 | 7.983489 |
| MIIT-AT VS HIIT | _cons | -28.58322 | 53.50888 | -0.53 | 0.593 | -133.4587 | 76.29225 |
| NUT VS HIIT | _cons | 2.309762 | 6597.109 | 0 | 1 | -12927.79 | 12932.41 |
| SOC VS HIIT | country | -0.2922536 | 0.261793 | -1.12 | 0.264 | -0.8053585 | 0.2208513 |
| SOC VS HIIT | _cons | 3.60463 | 2.35365 | 1.53 | 0.126 | -1.008439 | 8.217698 |

**Table S29** Meta-Regression Analysis of DBP Using Countries Conducting Research as a Moderator.

| **intervention measures** | **covariate** | **Coefficient** | **Std. err.** | **z** | **P>z** | **[95% conf.** | **interval]** |
| --- | --- | --- | --- | --- | --- | --- | --- |
| HIIT-AT VS HIIT | _cons | 1.198736 | 4.804643 | 0.25 | 0.803 | -8.218191 | 10.61566 |
| HIIT-MICT VS HIIT | _cons | 0.5987356 | 4.677883 | 0.13 | 0.898 | -8.569747 | 9.767219 |
| LICT VS HIIT | country | -0.326574 | 0.5036932 | -0.65 | 0.517 | -1.313795 | 0.6606466 |
| LICT VS HIIT | _cons | 1.967438 | 3.54111 | 0.56 | 0.578 | -4.97301 | 8.907886 |
| LICT-NUT VS HIIT | country | -2.773827 | 590.6038 | 0 | 0.996 | -1160.336 | 1154.788 |
| LICT-NUT VS HIIT | _cons | 42.03569 | 9449.656 | 0 | 0.996 | -18478.95 | 18563.02 |
| MICT VS HIIT | _cons | 2.702509 | 4.969179 | 0.54 | 0.587 | -7.036904 | 12.44192 |
| MICT-AT VS HIIT | _cons | -2.172244 | 5.942334 | -0.37 | 0.715 | -13.819 | 9.474516 |
| MICT-NUT VS HIIT | country | -0.1451685 | 0.4723069 | -0.31 | 0.759 | -1.070873 | 0.7805361 |
| MICT-NUT VS HIIT | _cons | -2.135613 | 4.382615 | -0.49 | 0.626 | -10.72538 | 6.454155 |
| MIIT VS HIIT | country | -0.3505166 | 0.2994054 | -1.17 | 0.242 | -0.9373404 | 0.2363072 |
| MIIT VS HIIT | _cons | 0.1565621 | 2.564158 | 0.06 | 0.951 | -4.869095 | 5.182219 |
| MIIT-AT VS HIIT | country | -0.481678 | 0.4523409 | -1.06 | 0.287 | -1.36825 | 0.4048939 |
| MIIT-AT VS HIIT | _cons | 1.177241 | 3.724771 | 0.32 | 0.752 | -6.123176 | 8.477659 |
| NUT VS HIIT | _cons | 9.102152 | 1181.218 | 0.01 | 0.994 | -2306.042 | 2324.246 |
| SOC VS HIIT | country | -0.1747524 | 0.3000109 | -0.58 | 0.56 | -0.7627629 | 0.4132581 |
| SOC VS HIIT | _cons | 2.573668 | 2.555494 | 1.01 | 0.314 | -2.435008 | 7.582345 |

**Table S30** Meta-Regression Analysis of MAS Using Countries Conducting Research as a Moderator.

| **intervention measures** | **covariate** | **Coefficient** | **Std. err.** | **z** | **P>z** | **[95% conf.** | **interval]** |
| --- | --- | --- | --- | --- | --- | --- | --- |
| HIIT VS HIIT-AT | _cons | -0.2102627 | 0.3914043 | -0.54 | 0.591 | -0.977401 | 0.5568757 |
| HIIT VS MICT | _cons | -0.4615526 | 0.3700899 | -1.25 | 0.212 | -1.186915 | 0.2638103 |
| HIIT VS MIIT | country | -0.037027 | 0.0832848 | -0.44 | 0.657 | -0.2002622 | 0.1262083 |
| HIIT VS MIIT | _cons | -0.362973 | 0.519159 | -0.7 | 0.484 | -1.380506 | 0.6545599 |
| HIIT VS SOC | _cons | -1.295018 | 0.2608376 | -4.96 | 0 | -1.80625 | -0.7837858 |

**Table S31** Meta-Regression Analysis of RHR Using Countries Conducting Research as a Moderator.

| **intervention measures** | **covariate** | **Coefficient** | **Std. err.** | **z** | **P>z** | **[95% conf.** | **interval]** |
| --- | --- | --- | --- | --- | --- | --- | --- |
| HIIT VS HIIT-NUT | _cons | -1.1 | 0.4626518 | -2.38 | 0.017 | -2.006781 | -0.1932192 |
| HIIT VS LICT | country | 0.2937627 | 0.515947 | 0.57 | 0.569 | -0.7174747 | 1.305 |
| HIIT VS LICT | _cons | -0.6520472 | 1.829877 | -0.36 | 0.722 | -4.238541 | 2.934447 |
| HIIT VS LICT-NUT | _cons | 22.40883 | 6.758849 | 3.32 | 0.001 | 9.16173 | 35.65593 |
| HIIT VS MICT | country | -3.51 | 2.735379 | -1.28 | 0.199 | -8.871243 | 1.851243 |
| HIIT VS MICT | _cons | 5.12 | 2.837285 | 1.8 | 0.071 | -0.4409762 | 10.68098 |
| HIIT VS MICT-AT | _cons | 16.95149 | 7.206614 | 2.35 | 0.019 | 2.826791 | 31.0762 |
| HIIT VS MICT-NUT | _cons | 25.40883 | 6.722556 | 3.78 | 0 | 12.23286 | 38.5848 |
| HIIT VS MIIT | country | -1.092638 | 1.54881 | -0.71 | 0.481 | -4.12825 | 1.942973 |
| HIIT VS MIIT | _cons | 2.077815 | 2.237271 | 0.93 | 0.353 | -2.307155 | 6.462785 |
| HIIT VS SOC | country | 1.271339 | 0.4265239 | 2.98 | 0.003 | 0.4353671 | 2.10731 |
| HIIT VS SOC | _cons | 1.067425 | 1.110843 | 0.96 | 0.337 | -1.109787 | 3.244637 |

**follow-up time**

**Table S32** Meta-Regression Analysis of VO_2_peak Using follow-up time as a Moderator.

| **intervention measures** | **covariate** | **Coefficient** | **Std. err.** | **z** | **P>z** | **[95% conf.** | **interval]** |
| --- | --- | --- | --- | --- | --- | --- | --- |
| AT VS HIIT | time | 0.1109056 | 14.72145 | 0.01 | 0.994 | -28.74261 | 28.96442 |
| AT VS HIIT | _cons | 1.163101 | 353.1888 | 0 | 0.997 | -691.0741 | 693.4003 |
| AT VS HIIT-AT | time | 0.756772 | 14.73573 | 0.05 | 0.959 | -28.12473 | 29.63827 |
| AT VS HIIT-AT | _cons | -6.704013 | 353.2611 | -0.02 | 0.985 | -699.083 | 685.675 |
| AT VS LICT | time | -0.2018791 | 14.71653 | -0.01 | 0.989 | -29.04574 | 28.64199 |
| AT VS LICT | _cons | 7.645194 | 353.1987 | 0.02 | 0.983 | -684.6115 | 699.9019 |
| AT VS MICT | time | -0.0699377 | 14.72507 | 0 | 0.996 | -28.93054 | 28.79066 |
| AT VS MICT | _cons | 1.331284 | 353.2077 | 0 | 0.997 | -690.9432 | 693.6057 |
| AT VS MIIT | time | 0.070966 | 14.71566 | 0 | 0.996 | -28.77119 | 28.91312 |
| AT VS MIIT | _cons | 0.1573843 | 353.1616 | 0 | 1 | -692.0266 | 692.3414 |
| AT VS MIIT-AT | _cons | 0.7000025 | 2.010509 | 0.35 | 0.728 | -3.240522 | 4.640527 |
| AT VS NUT | time | -0.9342518 | 14.73471 | -0.06 | 0.949 | -29.81375 | 27.94525 |
| AT VS NUT | _cons | 9.9409 | 353.3264 | 0.03 | 0.978 | -682.5662 | 702.448 |
| AT VS SOC | time | 0.0681211 | 14.71538 | 0 | 0.996 | -28.7735 | 28.90974 |
| AT VS SOC | _cons | -2.394806 | 353.158 | -0.01 | 0.995 | -694.5717 | 689.7821 |

**Table S33** Meta-Regression Analysis of VO_2_max Using follow-up time as a Moderator.

| **intervention measures** | **covariate** | **Coefficient** | **Std. err.** | **z** | **P>z** | **[95% conf.** | **interval]** |
| --- | --- | --- | --- | --- | --- | --- | --- |
| HIIT VS HICT-AT | time | -0.7625921 | 47.67265 | -0.02 | 0.987 | -94.19926 | 92.67408 |
| HIIT VS HICT-AT | _cons | 6.404063 | 572.0124 | 0.01 | 0.991 | -1114.72 | 1127.528 |
| HIIT-AT VS HICT-AT | _cons | -0.7027037 | 190.6807 | 0 | 0.997 | -374.4299 | 373.0245 |
| HIIT-MICT VS HICT-AT | _cons | 2.297296 | 190.6819 | 0.01 | 0.99 | -371.4323 | 376.0269 |
| LICT VS HICT-AT | time | -0.1821861 | 47.66517 | 0 | 0.997 | -93.6042 | 93.23983 |
| LICT VS HICT-AT | _cons | -0.3301964 | 571.9687 | 0 | 1 | -1121.368 | 1120.708 |
| MICT VS HICT-AT | _cons | -1.981203 | 95.4133 | -0.02 | 0.983 | -188.9878 | 185.0254 |
| MICT-AT VS HICT-AT | time | -1.151729 | 47.68215 | -0.02 | 0.981 | -94.60702 | 92.30356 |
| MICT-AT VS HICT-AT | _cons | 13.52112 | 572.2348 | 0.02 | 0.981 | -1108.038 | 1135.081 |
| MIIT VS HICT-AT | time | -0.1346723 | 47.67213 | 0 | 0.998 | -93.57033 | 93.30099 |
| MIIT VS HICT-AT | _cons | 0.2582771 | 572.0083 | 0 | 1 | -1120.857 | 1121.374 |
| MIIT-AT VS HICT-AT | _cons | -3.691637 | 95.40582 | -0.04 | 0.969 | -190.6836 | 183.3003 |
| SOC VS HICT-AT | time | -0.2242311 | 47.66498 | 0 | 0.996 | -93.64588 | 93.19742 |
| SOC VS HICT-AT | _cons | -1.108863 | 571.9587 | 0 | 0.998 | -1122.127 | 1119.91 |

**Table S34** Meta-Regression Analysis of HR_max_ Using follow-up time as a Moderator.

| **intervention measures** | **covariate** | **Coefficient** | **Std. err.** | **z** | **P>z** | **[95% conf.** | **interval]** |
| --- | --- | --- | --- | --- | --- | --- | --- |
| LICT VS HIIT | _cons | -3.779305 | 3.543223 | -1.07 | 0.286 | -10.72389 | 3.165285 |
| MICT VS HIIT | time | -0.209859 | 0.8013741 | -0.26 | 0.793 | -1.780523 | 1.360805 |
| MICT VS HIIT | _cons | 2.329577 | 7.759844 | 0.3 | 0.764 | -12.87944 | 17.53859 |
| MIIT VS HIIT | time | -0.7566377 | 0.9890617 | -0.77 | 0.444 | -2.695163 | 1.181888 |
| MIIT VS HIIT | _cons | 8.431108 | 10.30601 | 0.82 | 0.413 | -11.7683 | 28.63052 |
| SOC VS HIIT | time | -0.1048056 | 0.913957 | -0.11 | 0.909 | -1.896128 | 1.686517 |
| SOC VS HIIT | _cons | 0.8014265 | 9.83083 | 0.08 | 0.935 | -18.46665 | 20.0695 |

**Table S35** Meta-Regression Analysis of SBP Using follow-up time as a Moderator.

| **intervention measures** | **covariate** | **Coefficient** | **Std. err.** | **z** | **P>z** | **[95% conf.** | **interval]** |
| --- | --- | --- | --- | --- | --- | --- | --- |
| HIIT-AT VS HIIT | _cons | 0.912895 | 6.47342 | 0.14 | 0.888 | -11.77477 | 13.60056 |
| HIIT-MICT VS HIIT | _cons | 9.112895 | 6.042364 | 1.51 | 0.132 | -2.72992 | 20.95571 |
| LICT VS HIIT | time | 0.7787584 | 0.6586592 | 1.18 | 0.237 | -0.5121898 | 2.069707 |
| LICT VS HIIT | _cons | -8.860629 | 7.690869 | -1.15 | 0.249 | -23.93446 | 6.213198 |
| LICT-NUT VS HIIT | time | -0.7415877 | 177.4799 | 0 | 0.997 | -348.5957 | 347.1125 |
| LICT-NUT VS HIIT | _cons | 24.31869 | 3194.66 | 0.01 | 0.994 | -6237.099 | 6285.737 |
| MICT VS HIIT | _cons | -3.739617 | 3.444292 | -1.09 | 0.278 | -10.49031 | 3.011072 |
| MICT-AT VS HIIT | _cons | -3.495924 | 5.78442 | -0.6 | 0.546 | -14.83318 | 7.841331 |
| MICT-NUT VS HIIT | time | 0.9302961 | 0.6720653 | 1.38 | 0.166 | -0.3869277 | 2.24752 |
| MICT-NUT VS HIIT | _cons | -12.64168 | 8.830067 | -1.43 | 0.152 | -29.94829 | 4.664936 |
| MIIT VS HIIT | time | 0.3659513 | 0.6093035 | 0.6 | 0.548 | -0.8282616 | 1.560164 |
| MIIT VS HIIT | _cons | -4.974306 | 5.489437 | -0.91 | 0.365 | -15.7334 | 5.784793 |
| MIIT-AT VS HIIT | time | 0.1781466 | 3.83061 | 0.05 | 0.963 | -7.329711 | 7.686004 |
| MIIT-AT VS HIIT | _cons | -4.133684 | 42.25702 | -0.1 | 0.922 | -86.95592 | 78.68855 |
| NUT VS HIIT | _cons | 2.37153 | 3194.631 | 0 | 0.999 | -6258.99 | 6263.734 |
| SOC VS HIIT | time | 0.5977931 | 0.6077145 | 0.98 | 0.325 | -0.5933054 | 1.788892 |

**Table S36** Meta-Regression Analysis of DBP Using follow-up time as a Moderator.

| **intervention measures** | **covariate** | **Coefficient** | **Std. err.** | **z** | **P>z** | **[95% conf.** | **interval]** |
| --- | --- | --- | --- | --- | --- | --- | --- |
| HIIT-AT VS HIIT | _cons | 0.6297508 | 4.451458 | 0.14 | 0.887 | -8.094947 | 9.354449 |
| HIIT-MICT VS HIIT | _cons | 0.0297508 | 4.314334 | 0.01 | 0.994 | -8.426189 | 8.485691 |
| LICT VS HIIT | time | 0.6343124 | 0.561551 | 1.13 | 0.259 | -0.4663074 | 1.734932 |
| LICT VS HIIT | _cons | -7.197256 | 6.769836 | -1.06 | 0.288 | -20.46589 | 6.071378 |
| LICT-NUT VS HIIT | time | -0.3744568 | 140.1087 | 0 | 0.998 | -274.9824 | 274.2335 |
| LICT-NUT VS HIIT | _cons | 10.51153 | 2521.977 | 0 | 0.997 | -4932.472 | 4953.495 |
| MICT VS HIIT | _cons | 6.624977 | 4.01964 | 1.65 | 0.099 | -1.253373 | 14.50333 |
| MICT-AT VS HIIT | _cons | 1.925013 | 4.984157 | 0.39 | 0.699 | -7.843756 | 11.69378 |
| MICT-NUT VS HIIT | time | 0.4472403 | 0.5662823 | 0.79 | 0.43 | -0.6626527 | 1.557133 |
| MICT-NUT VS HIIT | _cons | -6.54964 | 7.591784 | -0.86 | 0.388 | -21.42926 | 8.329983 |
| MIIT VS HIIT | time | 0.585936 | 0.5327569 | 1.1 | 0.271 | -0.4582483 | 1.63012 |
| MIIT VS HIIT | _cons | -7.128248 | 4.81146 | -1.48 | 0.138 | -16.55854 | 2.302041 |
| MIIT-AT VS HIIT | _cons | -0.1041436 | 2.864847 | -0.04 | 0.971 | -5.719141 | 5.510854 |
| NUT VS HIIT | _cons | 2.931089 | 2521.949 | 0 | 0.999 | -4939.999 | 4945.861 |
| SOC VS HIIT | time | 0.4238162 | 0.5118955 | 0.83 | 0.408 | -0.5794805 | 1.427113 |
| SOC VS HIIT | _cons | -1.560777 | 5.07244 | -0.31 | 0.758 | -11.50258 | 8.381023 |

**Table S37** Meta-Regression Analysis of MAS Using follow-up time as a Moderator.

| **intervention measures** | **covariate** | **Coefficient** | **Std. err.** | **z** | **P>z** | **[95% conf.** | **interval]** |
| --- | --- | --- | --- | --- | --- | --- | --- |
| HIIT VS HIIT-AT | _cons | -0.1948639 | 0.4713964 | -0.41 | 0.679 | -1.118784 | 0.729056 |
| HIIT VS MICT | _cons | -0.4542103 | 0.4235329 | -1.07 | 0.284 | -1.28432 | 0.375899 |
| HIIT VS MIIT | time | 0.0719697 | 0.3503311 | 0.21 | 0.837 | -0.6146666 | 0.7586059 |
| HIIT VS MIIT | _cons | -1.204681 | 3.002641 | -0.4 | 0.688 | -7.089749 | 4.680387 |
| HIIT VS SOC | time | 0.0227549 | 0.1510809 | 0.15 | 0.88 | -0.2733582 | 0.318868 |
| HIIT VS SOC | _cons | -1.545519 | 1.647885 | -0.94 | 0.348 | -4.775314 | 1.684277 |
| YG VS CE | constant term | 0.0901942 | 0.3950688 | 0.23 | 0.819 | -0.6841265 | 0.8645149 |

**Table S38** Meta-Regression Analysis of RHR Using follow-up time as a Moderator.

| **intervention measures** | **covariate** | **Coefficient** | **Std. err.** | **z** | **P>z** | **[95% conf.** | **interval]** |
| --- | --- | --- | --- | --- | --- | --- | --- |
| HIIT VS HIIT-NUT | _cons | -1.1 | 0.4626518 | -2.38 | 0.017 | -2.006781 | -0.1932192 |
| HIIT VS LICT | _cons | -1.331516 | 1.633645 | -0.82 | 0.415 | -4.533401 | 1.87037 |
| HIIT VS LICT-NUT | _cons | -0.9117644 | 2941.569 | 0 | 1 | -5766.281 | 5764.458 |
| HIIT VS MICT | time | -0.5014286 | 0.3907684 | -1.28 | 0.199 | -1.26732 | 0.2644633 |
| HIIT VS MICT | _cons | 6.122857 | 3.611752 | 1.7 | 0.09 | -0.9560466 | 13.20176 |
| HIIT VS MICT-AT | _cons | -4.468425 | 1.60174 | -2.79 | 0.005 | -7.607779 | -1.329072 |
| HIIT VS MICT-NUT | _cons | 2.088236 | 2941.569 | 0 | 0.999 | -5763.281 | 5767.457 |
| HIIT VS MIIT | time | -0.3334922 | 0.3197973 | -1.04 | 0.297 | -0.9602835 | 0.293299 |
| HIIT VS MIIT | _cons | 3.943969 | 3.164882 | 1.25 | 0.213 | -2.259086 | 10.14702 |
| HIIT VS SOC | time | -0.7405563 | 490.2616 | 0 | 0.999 | -961.6356 | 960.1545 |
| HIIT VS SOC | _cons | 11.41825 | 5883.139 | 0 | 0.998 | -11519.32 | 11542.16 |

**Mean age**

**Table S39** Meta-Regression Analysis of VO_2_peak Using Mean age as a Moderator.

| **intervention measures** | **covariate** | **Coefficient** | **Std. err.** | **z** | **P>z** | **[95% conf.** | **interval]** |
| --- | --- | --- | --- | --- | --- | --- | --- |
| AT VS HIIT | age | -0.1629362 | 36.56415 | 0 | 0.996 | -71.82736 | 71.50149 |
| AT VS HIIT | _cons | 5.595404 | 570.387 | 0.01 | 0.992 | -1112.343 | 1123.533 |
| AT VS HIIT-AT | age | 0.7430577 | 36.56935 | 0.02 | 0.984 | -70.93154 | 72.41766 |
| AT VS HIIT-AT | _cons | -8.943162 | 570.4696 | -0.02 | 0.987 | -1127.043 | 1109.157 |
| AT VS LICT | age | 1.280284 | 36.5698 | 0.04 | 0.972 | -70.39521 | 72.95577 |
| AT VS LICT | _cons | -12.75015 | 570.428 | -0.02 | 0.982 | -1130.768 | 1105.268 |
| AT VS MICT | age | 0.0949384 | 36.56614 | 0 | 0.998 | -71.57339 | 71.76326 |
| AT VS MICT | _cons | 0.3336926 | 570.4116 | 0 | 1 | -1117.652 | 1118.32 |
| AT VS MIIT | age | -0.2312484 | 36.56379 | -0.01 | 0.995 | -71.89495 | 71.43246 |
| AT VS MIIT | _cons | 4.889208 | 570.3831 | 0.01 | 0.993 | -1113.041 | 1122.82 |
| AT VS MIIT-AT | _cons | 0.6999596 | 1.777357 | 0.39 | 0.694 | -2.783597 | 4.183516 |
| AT VS NUT | age | 1.759845 | 36.58655 | 0.05 | 0.962 | -69.94847 | 73.46816 |
| AT VS NUT | _cons | -21.32172 | 570.5477 | -0.04 | 0.97 | -1139.575 | 1096.931 |
| AT VS SOC | age | 0.261418 | 36.56397 | 0.01 | 0.994 | -71.40265 | 71.92548 |
| AT VS SOC | _cons | -4.267159 | 570.3858 | -0.01 | 0.994 | -1122.203 | 1113.668 |

**Table S40** Meta-Regression Analysis of VO_2_max Using Mean age as a Moderator.

| **intervention measures** | **covariate** | **Coefficient** | **Std. err.** | **z** | **P>z** | **[95% conf.** | **interval]** |
| --- | --- | --- | --- | --- | --- | --- | --- |
| HIIT VS HICT-AT | age | 1.56729 | 146.6899 | 0.01 | 0.991 | -285.9396 | 289.0742 |
| HIIT VS HICT-AT | _cons | -21.07779 | 1760.297 | -0.01 | 0.99 | -3471.197 | 3429.042 |
| HIIT-AT VS HICT-AT | _cons | -2.363647 | 278.7405 | -0.01 | 0.993 | -548.685 | 543.9577 |
| HIIT-MICT VS HICT-AT | _cons | 0.6363532 | 278.7413 | 0 | 0.998 | -545.6866 | 546.9593 |
| LICT VS HICT-AT | age | 0.1878278 | 146.6932 | 0 | 0.999 | -287.3256 | 287.7013 |
| LICT VS HICT-AT | _cons | -4.251855 | 1760.344 | 0 | 0.998 | -3454.463 | 3445.96 |
| MICT VS HICT-AT | _cons | -1.311789 | 406.3369 | 0 | 0.997 | -797.7176 | 795.094 |
| MICT-AT VS HICT-AT | age | -0.413237 | 146.6903 | 0 | 0.998 | -287.9209 | 287.0945 |
| MICT-AT VS HICT-AT | _cons | 1.397719 | 1760.283 | 0 | 0.999 | -3448.693 | 3451.489 |
| MIIT VS HICT-AT | age | -0.2791059 | 146.6933 | 0 | 0.998 | -287.7927 | 287.2344 |
| MIIT VS HICT-AT | _cons | 2.568546 | 1760.349 | 0 | 0.999 | -3447.653 | 3452.79 |
| MIIT-AT VS HICT-AT | _cons | -3.466044 | 146.7344 | -0.02 | 0.981 | -291.0602 | 284.1281 |
| SOC VS HICT-AT | age | 0.4021473 | 146.687 | 0 | 0.998 | -287.0991 | 287.9034 |
| SOC VS HICT-AT | _cons | -8.625362 | 1760.255 | 0 | 0.996 | -3458.662 | 3441.412 |

**Table S41** Meta-Regression Analysis of HR_max_ Using Mean age as a Moderator.

| **intervention measures** | **covariate** | **Coefficient** | **Std. err.** | **z** | **P>z** | **[95% conf.** | **interval]** |
| --- | --- | --- | --- | --- | --- | --- | --- |
| LICT VS HIIT | age | -5.272013 | 3.897056 | -1.35 | 0.176 | -12.9101 | 2.366077 |
| LICT VS HIIT | _cons | 62.67516 | 48.90854 | 1.28 | 0.2 | -33.18381 | 158.5341 |
| MICT VS HIIT | age | -6.153256 | 25.14271 | -0.24 | 0.807 | -55.43207 | 43.12556 |
| MICT VS HIIT | _cons | 100.7674 | 409.4371 | 0.25 | 0.806 | -701.7146 | 903.2494 |
| MIIT VS HIIT | age | 0.0063795 | 0.7596334 | 0.01 | 0.993 | -1.482474 | 1.495234 |
| MIIT VS HIIT | _cons | 0.4225239 | 10.01279 | 0.04 | 0.966 | -19.20219 | 20.04724 |
| SOC VS HIIT | age | -0.049686 | 0.7635379 | -0.07 | 0.948 | -1.546193 | 1.446821 |
| SOC VS HIIT | _cons | 0.452224 | 10.31957 | 0.04 | 0.965 | -19.77376 | 20.67821 |

**Table S42** Meta-Regression Analysis of SBP Using Mean age as a Moderator.

| **intervention measures** | **covariate** | **Coefficient** | **Std. err.** | **z** | **P>z** | **[95% conf.** | **interval]** |
| --- | --- | --- | --- | --- | --- | --- | --- |
| HIIT-AT VS HIIT | _cons | 3.390569 | 7.470512 | 0.45 | 0.65 | -11.25137 | 18.0325 |
| HIIT-MICT VS HIIT | _cons | 11.59057 | 7.10025 | 1.63 | 0.103 | -2.325665 | 25.5068 |
| LICT VS HIIT | age | -2.120134 | 2.104737 | -1.01 | 0.314 | -6.245343 | 2.005074 |
| LICT VS HIIT | _cons | 25.28369 | 25.59283 | 0.99 | 0.323 | -24.87733 | 75.44472 |
| LICT-NUT VS HIIT | age | 9.054851 | 3185.134 | 0 | 0.998 | -6233.694 | 6251.803 |
| LICT-NUT VS HIIT | _cons | -96.81825 | 36947.55 | 0 | 0.998 | -72512.69 | 72319.05 |
| MICT VS HIIT | age | -2.277299 | 1.801818 | -1.26 | 0.206 | -5.808797 | 1.254199 |
| MICT VS HIIT | _cons | 24.19706 | 23.44488 | 1.03 | 0.302 | -21.75407 | 70.14819 |
| MICT-AT VS HIIT | _cons | -5.944228 | 5.634422 | -1.05 | 0.291 | -16.98749 | 5.099036 |
| MICT-NUT VS HIIT | age | 0.7507933 | 2.218044 | 0.34 | 0.735 | -3.596492 | 5.098079 |
| MICT-NUT VS HIIT | _cons | -5.598104 | 24.47694 | -0.23 | 0.819 | -53.57203 | 42.37582 |
| MIIT VS HIIT | age | -0.7883488 | 0.8588244 | -0.92 | 0.359 | -2.471614 | 0.894916 |
| MIIT VS HIIT | _cons | 7.215375 | 10.87289 | 0.66 | 0.507 | -14.0951 | 28.52585 |
| MIIT-AT VS HIIT | age | -0.4197769 | 2.226886 | -0.19 | 0.85 | -4.784394 | 3.94484 |
| MIIT-AT VS HIIT | _cons | 2.721237 | 26.0953 | 0.1 | 0.917 | -48.42461 | 53.86708 |
| NUT VS HIIT | _cons | 2.373847 | 3726.602 | 0 | 0.999 | -7301.633 | 7306.38 |
| SOC VS HIIT | age | -0.7455341 | 1.19894 | -0.62 | 0.534 | -3.095414 | 1.604346 |
| SOC VS HIIT | _cons | 11.92048 | 15.82705 | 0.75 | 0.451 | -19.09997 | 42.94093 |

**Table S43** Meta-Regression Analysis of DBP Using Mean age as a Moderator.

| **intervention measures** | **covariate** | **Coefficient** | **Std. err.** | **z** | **P>z** | **[95% conf.** | **interval]** |
| --- | --- | --- | --- | --- | --- | --- | --- |
| HIIT-AT VS HIIT | _cons | 3.28174 | 5.294522 | 0.62 | 0.535 | -7.095332 | 13.65881 |
| HIIT-MICT VS HIIT | _cons | 2.68174 | 5.179764 | 0.52 | 0.605 | -7.470412 | 12.83389 |
| LICT VS HIIT | age | -2.199549 | 1.779118 | -1.24 | 0.216 | -5.686557 | 1.287459 |
| LICT VS HIIT | _cons | 26.733 | 21.53896 | 1.24 | 0.215 | -15.48258 | 68.94857 |
| LICT-NUT VS HIIT | age | 3.470287 | 1460.037 | 0 | 0.998 | -2858.15 | 2865.09 |
| LICT-NUT VS HIIT | _cons | -39.07435 | 16936.42 | 0 | 0.998 | -33233.86 | 33155.71 |
| MICT VS HIIT | _cons | 3.222995 | 4.317234 | 0.75 | 0.455 | -5.238627 | 11.68462 |
| MICT-AT VS HIIT | _cons | -0.1426839 | 4.860767 | -0.03 | 0.977 | -9.669612 | 9.384244 |
| MICT-NUT VS HIIT | age | -0.5770802 | 1.878333 | -0.31 | 0.759 | -4.258546 | 3.104385 |
| MICT-NUT VS HIIT | _cons | 5.905798 | 20.5187 | 0.29 | 0.773 | -34.31011 | 46.1217 |
| MIIT VS HIIT | age | 0.1390787 | 0.7888488 | 0.18 | 0.86 | -1.407036 | 1.685194 |
| MIIT VS HIIT | _cons | -4.295173 | 10.12192 | -0.42 | 0.671 | -24.13377 | 15.54343 |
| MIIT-AT VS HIIT | age | 0.7415896 | 2.692559 | 0.28 | 0.783 | -4.535729 | 6.018908 |
| MIIT-AT VS HIIT | _cons | -7.050954 | 29.25926 | -0.24 | 0.81 | -64.39806 | 50.29615 |
| NUT VS HIIT | _cons | 3.020748 | 1708.245 | 0 | 0.999 | -3345.078 | 3351.119 |
| SOC VS HIIT | age | -0.8895389 | 0.9985125 | -0.89 | 0.373 | -2.846588 | 1.06751 |
| SOC VS HIIT | _cons | 13.46609 | 13.08245 | 1.03 | 0.303 | -12.17504 | 39.10722 |

**Table S44** Meta-Regression Analysis of MAS Using Mean age as a Moderator.

| **intervention measures** | **covariate** | **Coefficient** | **Std. err.** | **z** | **P>z** | **[95% conf.** | **interval]** |
| --- | --- | --- | --- | --- | --- | --- | --- |
| HIIT VS HIIT-AT | _cons | -0.3278777 | 0.5517714 | -0.59 | 0.552 | -1.40933 | 0.7535743 |
| HIIT VS MICT | age | -0.8363275 | 1.694187 | -0.49 | 0.622 | -4.156873 | 2.484218 |
| HIIT VS MICT | _cons | 12.9627 | 27.35283 | 0.47 | 0.636 | -40.64787 | 66.57326 |
| HIIT VS MIIT | age | -0.0306456 | 0.1086119 | -0.28 | 0.778 | -0.243521 | 0.1822299 |
| HIIT VS MIIT | _cons | -0.154236 | 1.444965 | -0.11 | 0.915 | -2.986316 | 2.677844 |
| HIIT VS SOC | age | -0.2486256 | 0.3249788 | -0.77 | 0.444 | -0.8855724 | 0.3883211 |
| HIIT VS SOC | _cons | 2.592968 | 5.16103 | 0.5 | 0.615 | -7.522465 | 12.7084 |

**Table S45** Meta-Regression Analysis of RHR Using Mean age as a Moderator.

| **intervention measures** | **covariate** | **Coefficient** | **Std. err.** | **z** | **P>z** | **[95% conf.** | **interval]** |
| --- | --- | --- | --- | --- | --- | --- | --- |
| HIIT VS HIIT-NUT | country | 0.25246 | 2.592925 | 0.1 | 0.922 | -4.829579 | 5.334499 |
| HIIT VS LICT | _cons | -1.1 | 0.4626518 | -2.38 | 0.017 | -2.006781 | -0.1932192 |
| HIIT VS LICT | age | 18.1026 | 11.18969 | 1.62 | 0.106 | -3.828786 | 40.03398 |
| HIIT VS LICT-NUT | _cons | 3.898177 | 2.81368 | 1.39 | 0.166 | -1.616535 | 9.412889 |
| HIIT VS MICT | age | 1.329545 | 1.036128 | 1.28 | 0.199 | -0.7012286 | 3.360319 |
| HIIT VS MICT | _cons | -12.53636 | 10.96362 | -1.14 | 0.253 | -34.02466 | 8.951935 |
| HIIT VS MICT-AT | _cons | -2.948833 | 3.850662 | -0.77 | 0.444 | -10.49599 | 4.598325 |
| HIIT VS MICT-NUT | _cons | 6.898177 | 2.725346 | 2.53 | 0.011 | 1.556597 | 12.23976 |
| HIIT VS MIIT | age | 1.365842 | 1.16583 | 1.17 | 0.241 | -0.9191428 | 3.650826 |
| HIIT VS MIIT | _cons | -15.93514 | 15.31871 | -1.04 | 0.298 | -45.95927 | 14.08899 |
| HIIT VS SOC | age | 0.6068355 | 1.514201 | 0.4 | 0.689 | -2.360944 | 3.574615 |
| HIIT VS SOC | _cons | -4.141112 | 16.95511 | -0.24 | 0.807 | -37.37252 | 29.0903 |

**Network GRADE Classification**

**Table S46** Grade Grading Evaluation Form of VO_2_peak

| **Comparison** | **Number of studies** | **Within-study bias** | **Reporting bias** | **Indirectness** | **Imprecision** | **Heterogeneity** | **Incoherence** | **Confidence rating** | **Reason(s) for downgrading** |
| --- | --- | --- | --- | --- | --- | --- | --- | --- | --- |
| AT:MIIT | 1 | No concerns | Low risk | No concerns | Some concerns | No concerns | No concerns | Moderate | ["Imprecision"] |
| AT:MIIT-AT | 1 | No concerns | Low risk | No concerns | Some concerns | Some concerns | No concerns | Low | ["Imprecision","Heterogeneity"] |
| AT:SOC | 1 | No concerns | Low risk | No concerns | Some concerns | Some concerns | No concerns | Low | ["Imprecision","Heterogeneity"] |
| HIIT:HIIT-AT | 1 | No concerns | Low risk | No concerns | No concerns | Major concerns | No concerns | Low | ["Heterogeneity"] |
| HIIT:MICT | 5 | No concerns | Low risk | No concerns | No concerns | Some concerns | No concerns | Moderate | ["Heterogeneity"] |
| HIIT:MIIT | 3 | No concerns | Low risk | No concerns | No concerns | Some concerns | No concerns | Moderate | ["Heterogeneity"] |
| HIIT:NUT | 1 | No concerns | Low risk | No concerns | No concerns | No concerns | No concerns | High | [] |
| HIIT:SOC | 8 | No concerns | Low risk | No concerns | No concerns | Some concerns | No concerns | Moderate | ["Heterogeneity"] |
| HIIT-AT:SOC | 2 | No concerns | Low risk | No concerns | No concerns | Some concerns | No concerns | Moderate | ["Heterogeneity"] |
| LICT:SOC | 2 | No concerns | Low risk | No concerns | Some concerns | No concerns | No concerns | Moderate | ["Imprecision"] |
| MICT:NUT | 1 | No concerns | Low risk | No concerns | No concerns | Some concerns | Some concerns | Low | ["Heterogeneity","Incoherence"] |
| MICT:SOC | 2 | No concerns | Low risk | No concerns | Some concerns | No concerns | No concerns | Moderate | ["Imprecision"] |
| MIIT:MIIT-AT | 1 | No concerns | Low risk | No concerns | Some concerns | Some concerns | No concerns | Low | ["Imprecision","Heterogeneity"] |
| MIIT:NUT | 1 | No concerns | Low risk | No concerns | No concerns | No concerns | No concerns | High | [] |
| MIIT:SOC | 4 | No concerns | Low risk | No concerns | No concerns | Some concerns | No concerns | Moderate | ["Heterogeneity"] |
| MIIT-AT:SOC | 1 | No concerns | Low risk | No concerns | Some concerns | No concerns | No concerns | Moderate | ["Imprecision"] |
| AT:HIIT | 0 | No concerns | Low risk | No concerns | Some concerns | No concerns | No concerns | Moderate | ["Imprecision"] |
| AT:HIIT-AT | 0 | No concerns | Low risk | No concerns | Some concerns | Some concerns | No concerns | Low | ["Imprecision","Heterogeneity"] |
| AT:LICT | 0 | No concerns | Low risk | No concerns | Some concerns | Some concerns | No concerns | Low | ["Imprecision","Heterogeneity"] |
| AT:MICT | 0 | No concerns | Low risk | No concerns | Some concerns | Some concerns | No concerns | Low | ["Imprecision","Heterogeneity"] |
| AT:NUT | 0 | No concerns | Low risk | No concerns | Some concerns | No concerns | No concerns | Moderate | ["Imprecision"] |
| HIIT:LICT | 0 | No concerns | Low risk | No concerns | Some concerns | Some concerns | No concerns | Low | ["Imprecision","Heterogeneity"] |
| HIIT:MIIT-AT | 0 | No concerns | Low risk | No concerns | Some concerns | No concerns | No concerns | High | ["Imprecision"] |
| HIIT-AT:LICT | 0 | No concerns | Low risk | No concerns | Some concerns | Some concerns | No concerns | Low | ["Imprecision","Heterogeneity"] |
| HIIT-AT:MICT | 0 | No concerns | Low risk | No concerns | Some concerns | Some concerns | No concerns | Low | ["Imprecision","Heterogeneity"] |
| HIIT-AT:MIIT | 0 | No concerns | Low risk | No concerns | No concerns | Major concerns | No concerns | Low | ["Heterogeneity"] |
| HIIT-AT:MIIT-AT | 0 | No concerns | Low risk | No concerns | Some concerns | Some concerns | No concerns | Low | ["Imprecision","Heterogeneity"] |
| HIIT-AT:NUT | 0 | No concerns | Low risk | No concerns | No concerns | Some concerns | No concerns | Moderate | ["Heterogeneity"] |
| LICT:MICT | 0 | No concerns | Low risk | No concerns | Some concerns | Some concerns | No concerns | Low | ["Imprecision","Heterogeneity"] |
| LICT:MIIT | 0 | No concerns | Low risk | No concerns | Some concerns | Some concerns | No concerns | Low | ["Imprecision","Heterogeneity"] |
| LICT:MIIT-AT | 0 | No concerns | Low risk | No concerns | Some concerns | Some concerns | No concerns | Low | ["Imprecision","Heterogeneity"] |
| LICT:NUT | 0 | No concerns | Low risk | No concerns | No concerns | Some concerns | No concerns | Moderate | ["Heterogeneity"] |
| MICT:MIIT | 0 | No concerns | Low risk | No concerns | No concerns | Some concerns | No concerns | Moderate | ["Heterogeneity"] |
| MICT:MIIT-AT | 0 | No concerns | Low risk | No concerns | Some concerns | Some concerns | No concerns | Low | ["Imprecision","Heterogeneity"] |
| MIIT-AT:NUT | 0 | No concerns | Low risk | No concerns | Some concerns | No concerns | No concerns | Moderate | ["Imprecision"] |
| NUT:SOC | 0 | No concerns | Low risk | No concerns | Some concerns | No concerns | No concerns | Moderate | ["Imprecision"] |

**Table S47** Grade Grading Evaluation Form of VO_2_max

| **Comparison** | **Number of studies** | **Within-study bias** | **Reporting bias** | **Indirectness** | **Imprecision** | **Heterogeneity** | **Incoherence** | **Confidence rating** | **Reason(s) for downgrading** |
| --- | --- | --- | --- | --- | --- | --- | --- | --- | --- |
| HICT-AT:SOC | 1 | No concerns | Low risk | No concerns | No concerns | Some concerns | No concerns | Moderate | ["Heterogeneity"] |
| HIIT:MIIT | 2 | Some concerns | Low risk | No concerns | Some concerns | Some concerns | No concerns | Very low | ["Within-study bias","Imprecision","Heterogeneity"] |
| HIIT:MIIT-AT | 1 | Some concerns | Low risk | No concerns | No concerns | Some concerns | No concerns | Low | ["Within-study bias","Heterogeneity"] |
| HIIT:SOC | 4 | No concerns | Low risk | No concerns | Some concerns | Some concerns | No concerns | Low | ["Imprecision","Heterogeneity"] |
| HIIT-AT:HIIT-MICT | 1 | Some concerns | Low risk | No concerns | Some concerns | Some concerns | No concerns | Very low | ["Within-study bias","Imprecision","Heterogeneity"] |
| HIIT-AT:SOC | 1 | No concerns | Low risk | No concerns | Some concerns | Some concerns | No concerns | Low | ["Imprecision","Heterogeneity"] |
| HIIT-MICT:SOC | 1 | No concerns | Low risk | No concerns | No concerns | Some concerns | No concerns | Moderate | ["Heterogeneity"] |
| LICT:MICT | 1 | Some concerns | Low risk | No concerns | Major concerns | No concerns | No concerns | Very low | ["Within-study bias","Imprecision"] |
| LICT:MIIT | 1 | Some concerns | Low risk | No concerns | Some concerns | Some concerns | No concerns | Very low | ["Within-study bias","Imprecision","Heterogeneity"] |
| LICT:SOC | 3 | No concerns | Low risk | No concerns | No concerns | Some concerns | No concerns | Moderate | ["Heterogeneity"] |
| MICT:SOC | 1 | Some concerns | Low risk | No concerns | Major concerns | No concerns | No concerns | Very low | ["Within-study bias","Imprecision"] |
| MICT-AT:SOC | 2 | No concerns | Low risk | No concerns | Some concerns | Some concerns | No concerns | Low | ["Imprecision","Heterogeneity"] |
| MIIT:SOC | 3 | Some concerns | Low risk | No concerns | Some concerns | Some concerns | No concerns | Very low | ["Within-study bias","Imprecision","Heterogeneity"] |
| MIIT-AT:SOC | 1 | No concerns | Low risk | No concerns | No concerns | No concerns | No concerns | High | [] |
| HICT-AT:HIIT | 0 | Some concerns | Low risk | No concerns | No concerns | No concerns | No concerns | Moderate | ["Within-study bias"] |
| HICT-AT:HIIT-AT | 0 | No concerns | Low risk | No concerns | Major concerns | No concerns | No concerns | Low | ["Imprecision"] |
| HICT-AT:HIIT-MICT | 0 | Some concerns | Low risk | No concerns | No concerns | No concerns | No concerns | Moderate | ["Within-study bias"] |
| HICT-AT:LICT | 0 | No concerns | Low risk | No concerns | No concerns | No concerns | No concerns | High | [] |
| HICT-AT:MICT | 0 | Some concerns | Low risk | No concerns | Major concerns | No concerns | No concerns | Very low | ["Within-study bias","Imprecision"] |
| HICT-AT:MICT-AT | 0 | No concerns | Low risk | No concerns | No concerns | No concerns | No concerns | High | [] |
| HICT-AT:MIIT | 0 | No concerns | Low risk | No concerns | Major concerns | No concerns | No concerns | Low | ["Imprecision"] |
| HICT-AT:MIIT-AT | 0 | No concerns | Low risk | No concerns | No concerns | No concerns | No concerns | High | [] |
| HIIT:HIIT-AT | 0 | Some concerns | Low risk | No concerns | No concerns | No concerns | No concerns | Moderate | ["Within-study bias"] |
| HIIT:HIIT-MICT | 0 | No concerns | Low risk | No concerns | Some concerns | Some concerns | No concerns | Low | ["Imprecision","Heterogeneity"] |
| HIIT:LICT | 0 | Some concerns | Low risk | No concerns | Some concerns | Some concerns | No concerns | Very low | ["Within-study bias","Imprecision","Heterogeneity"] |
| HIIT:MICT | 0 | No concerns | Low risk | No concerns | Major concerns | No concerns | No concerns | Low | ["Imprecision"] |
| HIIT:MICT-AT | 0 | No concerns | Low risk | No concerns | No concerns | No concerns | No concerns | High | [] |
| HIIT-AT:LICT | 0 | No concerns | Low risk | No concerns | No concerns | No concerns | No concerns | High | [] |
| HIIT-AT:MICT | 0 | Some concerns | Low risk | No concerns | Major concerns | No concerns | No concerns | Very low | ["Within-study bias","Imprecision"] |
| HIIT-AT:MICT-AT | 0 | No concerns | Low risk | No concerns | No concerns | No concerns | No concerns | High | [] |
| HIIT-AT:MIIT | 0 | Some concerns | Low risk | No concerns | No concerns | No concerns | No concerns | Moderate | ["Within-study bias"] |
| HIIT-AT:MIIT-AT | 0 | No concerns | Low risk | No concerns | No concerns | No concerns | No concerns | High | [] |
| HIIT-MICT:LICT | 0 | No concerns | Low risk | No concerns | Some concerns | Some concerns | No concerns | Low | ["Imprecision","Heterogeneity"] |
| HIIT-MICT:MICT | 0 | No concerns | Low risk | No concerns | No concerns | No concerns | No concerns | High | [] |
| HIIT-MICT:MICT-AT | 0 | Some concerns | Low risk | No concerns | Major concerns | No concerns | No concerns | Very low | ["Within-study bias","Imprecision"] |
| HIIT-MICT:MIIT | 0 | No concerns | Low risk | No concerns | No concerns | Some concerns | No concerns | Moderate | ["Heterogeneity"] |
| HIIT-MICT:MIIT-AT | 0 | Some concerns | Low risk | No concerns | Some concerns | Some concerns | No concerns | Very low | ["Within-study bias","Imprecision","Heterogeneity"] |
| LICT:MICT-AT | 0 | No concerns | Low risk | No concerns | No concerns | No concerns | No concerns | High | [] |
| LICT:MIIT-AT | 0 | Some concerns | Low risk | No concerns | Major concerns | No concerns | No concerns | Very low | ["Within-study bias","Imprecision"] |
| MICT:MICT-AT | 0 | No concerns | Low risk | No concerns | No concerns | No concerns | No concerns | High | [] |
| MICT:MIIT | 0 | No concerns | Low risk | No concerns | Major concerns | No concerns | No concerns | Low | ["Imprecision"] |
| MICT:MIIT-AT | 0 | Some concerns | Low risk | No concerns | No concerns | No concerns | No concerns | Moderate | ["Within-study bias"] |
| MICT-AT:MIIT | 0 | No concerns | Low risk | No concerns | No concerns | No concerns | No concerns | High | [] |
| MICT-AT:MIIT-AT | 0 | No concerns | Low risk | No concerns | No concerns | No concerns | No concerns | High | [] |
| MIIT:MIIT-AT | 0 | No concerns | Low risk | No concerns | Some concerns | Some concerns | No concerns | Low | ["Imprecision","Heterogeneity"] |

**Table S48** Grade Grading Evaluation Form of HR_max_

| **Comparison** | **Number of studies** | **Within-study bias** | **Reporting bias** | **Indirectness** | **Imprecision** | **Heterogeneity** | **Incoherence** | **Confidence rating** | **Reason(s) for downgrading** |
| --- | --- | --- | --- | --- | --- | --- | --- | --- | --- |
| HIIT:LICT | 1 | No concerns | Low risk | No concerns | Some concerns | Some concerns | No concerns | Low | ["Imprecision","Heterogeneity"] |
| HIIT:MICT | 2 | No concerns | Low risk | No concerns | No concerns | Some concerns | No concerns | Moderate | ["Heterogeneity"] |
| HIIT:MIIT | 4 | No concerns | Low risk | No concerns | No concerns | No concerns | No concerns | High | [] |
| HIIT:SOC | 7 | No concerns | Low risk | No concerns | No concerns | No concerns | No concerns | High | [] |
| LICT:MIIT | 1 | Some concerns | Low risk | No concerns | No concerns | Some concerns | No concerns | Low | ["Within-study bias","Heterogeneity"] |
| LICT:SOC | 3 | No concerns | Low risk | No concerns | Some concerns | Some concerns | No concerns | Low | ["Imprecision","Heterogeneity"] |
| MICT:SOC | 1 | No concerns | Low risk | No concerns | Major concerns | No concerns | No concerns | Low | ["Imprecision"] |
| MIIT:SOC | 6 | Some concerns | Low risk | No concerns | No concerns | Major concerns | Major concerns | Very low | ["Within-study bias","Heterogeneity","Incoherence"] |
| LICT:MICT | 0 | No concerns | Low risk | No concerns | Some concerns | Some concerns | No concerns | Low | ["Imprecision","Heterogeneity"] |
| MICT:MIIT | 0 | Some concerns | Low risk | No concerns | Major concerns | No concerns | No concerns | Very low | ["Within-study bias","Imprecision"] |

**Table S49** Grade Grading Evaluation Form of SBP

| **Comparison** | **Number of studies** | **Within-study bias** | **Reporting bias** | **Indirectness** | **Imprecision** | **Heterogeneity** | **Incoherence** | **Confidence rating** | **Reason(s) for downgrading** |
| --- | --- | --- | --- | --- | --- | --- | --- | --- | --- |
| HIIT:LICT | 1 | Some concerns | Low risk | No concerns | Some concerns | Some concerns | No concerns | Very low | ["Within-study bias","Imprecision","Heterogeneity"] |
| HIIT:MICT | 1 | No concerns | Low risk | No concerns | Some concerns | No concerns | No concerns | Moderate | ["Imprecision"] |
| HIIT:MIIT | 4 | Some concerns | Low risk | No concerns | Some concerns | Some concerns | No concerns | Very low | ["Within-study bias","Imprecision","Heterogeneity"] |
| HIIT:MIIT-AT | 1 | Some concerns | Low risk | No concerns | Some concerns | Some concerns | No concerns | Very low | ["Within-study bias","Imprecision","Heterogeneity"] |
| HIIT:SOC | 7 | Some concerns | Low risk | No concerns | Some concerns | Some concerns | No concerns | Very low | ["Within-study bias","Imprecision","Heterogeneity"] |
| HIIT-AT:HIIT-MICT | 1 | No concerns | Low risk | No concerns | Some concerns | Some concerns | No concerns | Low | ["Imprecision","Heterogeneity"] |
| HIIT-AT:SOC | 1 | No concerns | Low risk | No concerns | Major concerns | No concerns | No concerns | Low | ["Imprecision"] |
| HIIT-MICT:SOC | 1 | Some concerns | Low risk | No concerns | Some concerns | Some concerns | No concerns | High | ["Within-study bias","Imprecision","Heterogeneity"] |
| LICT:MIIT | 1 | No concerns | Low risk | No concerns | Some concerns | Some concerns | No concerns | Low | ["Imprecision","Heterogeneity"] |
| LICT:SOC | 4 | Some concerns | Low risk | No concerns | Some concerns | Some concerns | No concerns | Very low | ["Within-study bias","Imprecision","Heterogeneity"] |
| LICT-NUT:MICT-NUT | 1 | No concerns | Low risk | No concerns | Some concerns | No concerns | No concerns | Moderate | ["Imprecision"] |
| LICT-NUT:NUT | 1 | Some concerns | Low risk | No concerns | Some concerns | Some concerns | No concerns | Very low | ["Within-study bias","Imprecision","Heterogeneity"] |
| LICT-NUT:SOC | 1 | Some concerns | Low risk | No concerns | Some concerns | Some concerns | No concerns | Very low | ["Within-study bias","Imprecision","Heterogeneity"] |
| MICT:SOC | 2 | Some concerns | Low risk | No concerns | No concerns | Some concerns | No concerns | Low | ["Within-study bias","Heterogeneity"] |
| MICT-AT:SOC | 1 | No concerns | Low risk | No concerns | Some concerns | Some concerns | No concerns | Low | ["Imprecision","Heterogeneity"] |
| MICT-NUT:SOC | 3 | Some concerns | Low risk | No concerns | Some concerns | Some concerns | No concerns | Very low | ["Within-study bias","Imprecision","Heterogeneity"] |
| MIIT:SOC | 6 | No concerns | Low risk | No concerns | No concerns | Some concerns | No concerns | Moderate | ["Heterogeneity"] |
| MIIT-AT:SOC | 2 | No concerns | Low risk | No concerns | Some concerns | No concerns | No concerns | Moderate | ["Imprecision"] |
| HIIT:HIIT-AT | 0 | No concerns | Low risk | No concerns | No concerns | No concerns | No concerns | High | [] |
| HIIT:HIIT-MICT | 0 | No concerns | Low risk | No concerns | Some concerns | No concerns | No concerns | Moderate | ["Imprecision"] |
| HIIT:LICT-NUT | 0 | Some concerns | Low risk | No concerns | Some concerns | Some concerns | No concerns | Very low | ["Within-study bias","Imprecision","Heterogeneity"] |
| HIIT:MICT-AT | 0 | Some concerns | Low risk | No concerns | Major concerns | No concerns | No concerns | Very low | ["Within-study bias","Imprecision"] |
| HIIT:MICT-NUT | 0 | No concerns | Low risk | No concerns | Major concerns | No concerns | No concerns | Low | ["Imprecision"] |
| HIIT:NUT | 0 | Some concerns | Low risk | No concerns | Some concerns | No concerns | No concerns | Low | ["Within-study bias","Imprecision"] |
| HIIT-AT:LICT | 0 | Some concerns | Low risk | No concerns | Major concerns | No concerns | No concerns | Very low | ["Within-study bias","Imprecision"] |
| HIIT-AT:LICT-NUT | 0 | No concerns | Low risk | No concerns | No concerns | No concerns | No concerns | High | [] |
| HIIT-AT:MICT | 0 | No concerns | Low risk | No concerns | No concerns | No concerns | No concerns | High | [] |
| HIIT-AT:MICT-AT | 0 | Some concerns | Low risk | No concerns | Major concerns | No concerns | No concerns | Very low | ["Within-study bias","Imprecision"] |
| HIIT-AT:MICT-NUT | 0 | Some concerns | Low risk | No concerns | Major concerns | No concerns | No concerns | Very low | ["Within-study bias","Imprecision"] |
| HIIT-AT:MIIT | 0 | Some concerns | Low risk | No concerns | No concerns | No concerns | No concerns | Moderate | ["Within-study bias"] |
| HIIT-AT:MIIT-AT | 0 | No concerns | Low risk | No concerns | No concerns | No concerns | No concerns | High | [] |
| HIIT-AT:NUT | 0 | No concerns | Low risk | No concerns | Major concerns | No concerns | No concerns | Very low | ["Imprecision"] |
| HIIT-MICT:LICT | 0 | No concerns | Low risk | No concerns | Some concerns | No concerns | No concerns | Moderate | ["Imprecision"] |
| HIIT-MICT:LICT-NUT | 0 | Some concerns | Low risk | No concerns | Major concerns | No concerns | No concerns | Very low | ["Within-study bias","Imprecision"] |
| HIIT-MICT:MICT | 0 | No concerns | Low risk | No concerns | No concerns | No concerns | No concerns | High | [] |
| HIIT-MICT:MICT-AT | 0 | Some concerns | Low risk | No concerns | No concerns | Some concerns | No concerns | Low | ["Within-study bias","Heterogeneity"] |
| HIIT-MICT:MICT-NUT | 0 | Some concerns | Low risk | No concerns | Some concerns | No concerns | No concerns | Low | ["Within-study bias","Imprecision"] |
| HIIT-MICT:MIIT | 0 | No concerns | Low risk | No concerns | No concerns | Some concerns | No concerns | Moderate | ["Heterogeneity"] |
| HIIT-MICT:MIIT-AT | 0 | No concerns | Low risk | No concerns | No concerns | Some concerns | No concerns | Moderate | ["Heterogeneity"] |
| HIIT-MICT:NUT | 0 | Some concerns | Low risk | No concerns | Major concerns | No concerns | No concerns | Very low | ["Within-study bias","Imprecision"] |
| LICT:LICT-NUT | 0 | Some concerns | Low risk | No concerns | Some concerns | Some concerns | No concerns | Very low | ["Within-study bias","Imprecision","Heterogeneity"] |
| LICT:MICT | 0 | Some concerns | Low risk | No concerns | Some concerns | Some concerns | No concerns | Very low | ["Within-study bias","Imprecision","Heterogeneity"] |
| LICT:MICT-AT | 0 | No concerns | Low risk | No concerns | No concerns | No concerns | No concerns | High | [] |
| LICT:MICT-NUT | 0 | No concerns | Low risk | No concerns | Major concerns | No concerns | No concerns | Low | ["Imprecision"] |
| LICT:MIIT-AT | 0 | Some concerns | Low risk | No concerns | Some concerns | Some concerns | No concerns | Very low | ["Within-study bias","Imprecision","Heterogeneity"] |
| LICT:NUT | 0 | No concerns | Low risk | No concerns | Some concerns | No concerns | No concerns | Moderate | ["Imprecision"] |
| LICT-NUT:MICT | 0 | Some concerns | Low risk | No concerns | No concerns | Some concerns | No concerns | Low | ["Within-study bias","Heterogeneity"] |
| LICT-NUT:MICT-AT | 0 | No concerns | Low risk | No concerns | Some concerns | No concerns | No concerns | Moderate | ["Imprecision"] |
| LICT-NUT:MIIT | 0 | Some concerns | Low risk | No concerns | No concerns | Some concerns | No concerns | Low | ["Within-study bias","Heterogeneity"] |
| LICT-NUT:MIIT-AT | 0 | No concerns | Low risk | No concerns | Some concerns | No concerns | No concerns | Moderate | ["Imprecision"] |
| MICT:MICT-AT | 0 | Some concerns | Low risk | No concerns | Major concerns | No concerns | No concerns | Very low | ["Within-study bias","Imprecision"] |
| MICT:MICT-NUT | 0 | No concerns | Low risk | No concerns | Some concerns | Some concerns | No concerns | Low | ["Imprecision","Heterogeneity"] |
| MICT:MIIT | 0 | Some concerns | Low risk | No concerns | Some concerns | Some concerns | No concerns | Very low | ["Within-study bias","Imprecision","Heterogeneity"] |
| MICT:MIIT-AT | 0 | No concerns | Low risk | No concerns | No concerns | No concerns | No concerns | High | [] |
| MICT:NUT | 0 | Some concerns | Low risk | No concerns | No concerns | No concerns | No concerns | Moderate | ["Within-study bias"] |
| MICT-AT:MICT-NUT | 0 | No concerns | Low risk | No concerns | Major concerns | No concerns | No concerns | Low | ["Imprecision"] |
| MICT-AT:MIIT | 0 | Some concerns | Low risk | No concerns | Major concerns | No concerns | No concerns | Very low | ["Within-study bias","Imprecision"] |
| MICT-AT:MIIT-AT | 0 | No concerns | Low risk | No concerns | No concerns | No concerns | No concerns | High | [] |
| MICT-AT:NUT | 0 | Some concerns | Low risk | No concerns | No concerns | Some concerns | No concerns | Low | ["Within-study bias","Heterogeneity"] |
| MICT-NUT:MIIT | 0 | No concerns | Low risk | No concerns | Some concerns | Some concerns | No concerns | Low | ["Imprecision","Heterogeneity"] |
| MICT-NUT:MIIT-AT | 0 | Some concerns | Low risk | No concerns | Major concerns | No concerns | No concerns | Very low | ["Within-study bias","Imprecision"] |
| MICT-NUT:NUT | 0 | Some concerns | Low risk | No concerns | Some concerns | No concerns | No concerns | Low | ["Within-study bias","Imprecision"] |
| MIIT:MIIT-AT | 0 | No concerns | Low risk | No concerns | No concerns | No concerns | No concerns | High | [] |
| MIIT:NUT | 0 | No concerns | Low risk | No concerns | No concerns | Some concerns | No concerns | Moderate | ["Heterogeneity"] |
| MIIT-AT:NUT | 0 | Some concerns | Low risk | No concerns | No concerns | Some concerns | No concerns | Low | ["Within-study bias","Heterogeneity"] |
| NUT:SOC | 0 | No concerns | Low risk | No concerns | Some concerns | Some concerns | No concerns | Low | ["Imprecision","Heterogeneity"] |

**Table S50** Grade Grading Evaluation Form of DBP

| **Comparison** | **Number of studies** | **Within-study bias** | **Reporting bias** | **Indirectness** | **Imprecision** | **Heterogeneity** | **Incoherence** | **Confidence rating** | **Reason(s) for downgrading** |
| --- | --- | --- | --- | --- | --- | --- | --- | --- | --- |
| HIIT:LICT | 1 | No concerns | Low risk | No concerns | No concerns | No concerns | No concerns | High | [] |
| HIIT:MIIT | 4 | Some concerns | Low risk | No concerns | Some concerns | Some concerns | Some concerns | Very low | ["Within-study bias","Imprecision","Heterogeneity","Incoherence"] |
| HIIT:MIIT-AT | 1 | Some concerns | Low risk | No concerns | Major concerns | No concerns | No concerns | Very low | ["Imprecision"] |
| HIIT:SOC | 6 | No concerns | Low risk | No concerns | Some concerns | Some concerns | No concerns | Low | ["Imprecision","Heterogeneity"] |
| HIIT-AT:HIIT-MICT | 1 | Some concerns | Low risk | No concerns | Major concerns | No concerns | No concerns | Very low | ["Within-study bias","Imprecision"] |
| HIIT-AT:SOC | 1 | No concerns | Low risk | No concerns | No concerns | No concerns | No concerns | High | [] |
| HIIT-MICT:SOC | 1 | Some concerns | Low risk | No concerns | Major concerns | No concerns | No concerns | Very low | ["Within-study bias","Imprecision"] |
| LICT:MIIT | 1 | Some concerns | Low risk | No concerns | Some concerns | Some concerns | No concerns | Very low | ["Within-study bias","Imprecision","Heterogeneity"] |
| LICT:SOC | 4 | Some concerns | Low risk | No concerns | Some concerns | Some concerns | No concerns | Very low | ["Within-study bias","Imprecision","Heterogeneity"] |
| LICT-NUT:MICT-NUT | 1 | No concerns | Low risk | No concerns | Major concerns | No concerns | No concerns | Low | ["Imprecision"] |
| LICT-NUT:NUT | 1 | No concerns | Low risk | No concerns | Some concerns | Some concerns | No concerns | Low | ["Imprecision","Heterogeneity"] |
| LICT-NUT:SOC | 1 | Some concerns | Low risk | No concerns | No concerns | No concerns | No concerns | Moderate | ["Within-study bias"] |
| MICT:SOC | 1 | Some concerns | Low risk | No concerns | Some concerns | Some concerns | No concerns | Very low | ["Within-study bias","Imprecision","Heterogeneity"] |
| MICT-AT:SOC | 1 | Some concerns | Low risk | No concerns | No concerns | No concerns | No concerns | Moderate | ["Within-study bias"] |
| MICT-NUT:SOC | 3 | Some concerns | Low risk | No concerns | No concerns | Some concerns | No concerns | Low | ["Within-study bias","Heterogeneity"] |
| MIIT:SOC | 5 | No concerns | Low risk | No concerns | No concerns | Some concerns | No concerns | Moderate | ["Heterogeneity"] |
| MIIT-AT:SOC | 2 | Some concerns | Low risk | No concerns | Some concerns | Some concerns | No concerns | Very low | ["Within-study bias","Imprecision","Heterogeneity"] |
| HIIT:HIIT-AT | 0 | Some concerns | Low risk | No concerns | Major concerns | No concerns | No concerns | Very low | ["Within-study bias","Imprecision"] |
| HIIT:HIIT-MICT | 0 | No concerns | Low risk | No concerns | No concerns | No concerns | No concerns | High | [] |
| HIIT:LICT-NUT | 0 | Some concerns | Low risk | No concerns | Major concerns | No concerns | No concerns | Very low | ["Within-study bias","Imprecision"] |
| HIIT:MICT | 0 | Some concerns | Low risk | No concerns | Some concerns | Some concerns | No concerns | Very low | ["Within-study bias","Imprecision","Heterogeneity"] |
| HIIT:MICT-AT | 0 | No concerns | Low risk | No concerns | No concerns | No concerns | No concerns | High | [] |
| HIIT:MICT-NUT | 0 | Some concerns | Low risk | No concerns | Some concerns | Some concerns | No concerns | Very low | ["Within-study bias","Imprecision","Heterogeneity"] |
| HIIT:NUT | 0 | No concerns | Low risk | No concerns | Major concerns | No concerns | No concerns | Low | ["Imprecision"] |
| HIIT-AT:LICT | 0 | No concerns | Low risk | No concerns | Major concerns | No concerns | No concerns | Low | ["Imprecision"] |
| HIIT-AT:LICT-NUT | 0 | Some concerns | Low risk | No concerns | No concerns | No concerns | No concerns | Moderate | ["Within-study bias"] |
| HIIT-AT:MICT | 0 | No concerns | Low risk | No concerns | Major concerns | No concerns | No concerns | Low | ["Imprecision"] |
| HIIT-AT:MICT-AT | 0 | Some concerns | Low risk | No concerns | No concerns | No concerns | No concerns | Moderate | ["Within-study bias"] |
| HIIT-AT:MICT-NUT | 0 | Some concerns | Low risk | No concerns | Major concerns | No concerns | No concerns | Very low | ["Within-study bias","Imprecision"] |
| HIIT-AT:MIIT | 0 | No concerns | Low risk | No concerns | No concerns | No concerns | No concerns | High | [] |
| HIIT-AT:MIIT-AT | 0 | Some concerns | Low risk | No concerns | No concerns | No concerns | No concerns | Moderate | ["Within-study bias"] |
| HIIT-AT:NUT | 0 | Some concerns | Low risk | No concerns | Major concerns | No concerns | No concerns | Very low | ["Within-study bias","Imprecision"] |
| HIIT-MICT:LICT | 0 | Some concerns | Low risk | No concerns | Major concerns | No concerns | No concerns | Very low | ["Within-study bias","Imprecision"] |
| HIIT-MICT:LICT-NUT | 0 | No concerns | Low risk | No concerns | No concerns | No concerns | No concerns | High | [] |
| HIIT-MICT:MICT | 0 | No concerns | Low risk | No concerns | Major concerns | No concerns | No concerns | Low | ["Imprecision"] |
| HIIT-MICT:MICT-AT | 0 | Some concerns | Low risk | No concerns | No concerns | No concerns | No concerns | Moderate | ["Within-study bias"] |
| HIIT-MICT:MICT-NUT | 0 | Some concerns | Low risk | No concerns | No concerns | No concerns | No concerns | Moderate | ["Within-study bias"] |
| HIIT-MICT:MIIT | 0 | Some concerns | Low risk | No concerns | No concerns | No concerns | No concerns | Moderate | ["Within-study bias"] |
| HIIT-MICT:MIIT-AT | 0 | Some concerns | Low risk | No concerns | No concerns | No concerns | No concerns | Moderate | ["Within-study bias"] |
| HIIT-MICT:NUT | 0 | No concerns | Low risk | No concerns | Major concerns | No concerns | No concerns | Low | ["Imprecision"] |
| LICT:LICT-NUT | 0 | Some concerns | Low risk | No concerns | Major concerns | No concerns | No concerns | Very low | ["Within-study bias","Imprecision"] |
| LICT:MICT | 0 | Some concerns | Low risk | No concerns | Some concerns | Some concerns | No concerns | Very low | ["Within-study bias","Imprecision","Heterogeneity"] |
| LICT:MICT-AT | 0 | No concerns | Low risk | No concerns | No concerns | No concerns | No concerns | High | [] |
| LICT:MICT-NUT | 0 | Some concerns | Low risk | No concerns | Some concerns | Some concerns | No concerns | Very low | ["Within-study bias","Imprecision","Heterogeneity"] |
| LICT:MIIT-AT | 0 | Some concerns | Low risk | No concerns | Major concerns | No concerns | No concerns | Very low | ["Within-study bias","Imprecision"] |
| LICT:NUT | 0 | No concerns | Low risk | No concerns | No concerns | No concerns | No concerns | High | [] |
| LICT-NUT:MICT | 0 | No concerns | Low risk | No concerns | No concerns | No concerns | No concerns | High | [] |
| LICT-NUT:MICT-AT | 0 | Some concerns | Low risk | No concerns | Major concerns | No concerns | No concerns | Very low | ["Within-study bias","Imprecision"] |
| LICT-NUT:MIIT | 0 | No concerns | Low risk | No concerns | Major concerns | No concerns | No concerns | Low | ["Imprecision"] |
| LICT-NUT:MIIT-AT | 0 | Some concerns | Low risk | No concerns | No concerns | No concerns | No concerns | Moderate | ["Within-study bias"] |
| MICT:MICT-AT | 0 | No concerns | Low risk | No concerns | No concerns | No concerns | No concerns | High | [] |
| MICT:MICT-NUT | 0 | Some concerns | Low risk | No concerns | No concerns | Some concerns | No concerns | Low | ["Within-study bias","Heterogeneity"] |
| MICT:MIIT | 0 | No concerns | Low risk | No concerns | No concerns | Some concerns | No concerns | Moderate | ["Heterogeneity"] |
| MICT:MIIT-AT | 0 | No concerns | Low risk | No concerns | Some concerns | Some concerns | No concerns | Low | ["Imprecision","Heterogeneity"] |
| MICT:NUT | 0 | Some concerns | Low risk | No concerns | Major concerns | No concerns | No concerns | Very low | ["Within-study bias","Imprecision"] |
| MICT-AT:MICT-NUT | 0 | No concerns | Low risk | No concerns | No concerns | No concerns | No concerns | High | [] |
| MICT-AT:MIIT | 0 | No concerns | Low risk | No concerns | Major concerns | No concerns | No concerns | Low | ["Imprecision"] |
| MICT-AT:MIIT-AT | 0 | Some concerns | Low risk | No concerns | No concerns | No concerns | No concerns | Moderate | ["Within-study bias"] |
| MICT-AT:NUT | 0 | No concerns | Low risk | No concerns | Major concerns | No concerns | No concerns | Low | ["Imprecision"] |
| MICT-NUT:MIIT | 0 | Some concerns | Low risk | No concerns | No concerns | No concerns | No concerns | Moderate | ["Within-study bias"] |
| MICT-NUT:MIIT-AT | 0 | Some concerns | Low risk | No concerns | No concerns | No concerns | No concerns | Moderate | ["Within-study bias"] |
| MICT-NUT:NUT | 0 | No concerns | Low risk | No concerns | Some concerns | Some concerns | No concerns | Low | ["Imprecision","Heterogeneity"] |
| MIIT:MIIT-AT | 0 | No concerns | Low risk | No concerns | No concerns | No concerns | No concerns | High | [] |
| MIIT:NUT | 0 | No concerns | Low risk | No concerns | Some concerns | Some concerns | No concerns | Low | ["Imprecision","Heterogeneity"] |
| MIIT-AT:NUT | 0 | No concerns | Low risk | No concerns | Major concerns | No concerns | No concerns | Low | ["Imprecision"] |

**Table S51** Grade Grading Evaluation Form of MAS

| **Comparison** | **Number of studies** | **Within-study bias** | **Reporting bias** | **Indirectness** | **Imprecision** | **Heterogeneity** | **Incoherence** | **Confidence rating** | **Reason(s) for downgrading** |
| --- | --- | --- | --- | --- | --- | --- | --- | --- | --- |
| HIIT:HIIT-AT | 1 | No concerns | Low risk | No concerns | Some concerns | Some concerns | No concerns | Low | ["Imprecision","Heterogeneity"] |
| HIIT:MICT | 2 | Some concerns | Low risk | No concerns | Some concerns | Some concerns | No concerns | Very low | ["Within-study bias","Imprecision","Heterogeneity"] |
| HIIT:MIIT | 3 | Some concerns | Low risk | No concerns | No concerns | Some concerns | No concerns | Low | ["Within-study bias","Heterogeneity"] |
| HIIT:SOC | 5 | No concerns | Low risk | No concerns | No concerns | No concerns | No concerns | High | [] |
| HIIT-AT:SOC | 1 | Some concerns | Low risk | No concerns | No concerns | Some concerns | No concerns | Low | ["Within-study bias","Heterogeneity"] |
| MICT:SOC | 2 | No concerns | Low risk | No concerns | No concerns | Some concerns | No concerns | Moderate | ["Heterogeneity"] |
| MIIT:SOC | 2 | Some concerns | Low risk | No concerns | No concerns | Some concerns | No concerns | Low | ["Within-study bias","Heterogeneity"] |
| HIIT-AT:MICT | 0 | No concerns | Low risk | No concerns | Major concerns | No concerns | No concerns | Low | ["Imprecision"] |
| HIIT-AT:MIIT | 0 | No concerns | Low risk | No concerns | Some concerns | Some concerns | No concerns | Low | ["Imprecision","Heterogeneity"] |
| MICT:MIIT | 0 | No concerns | Low risk | No concerns | Major concern | No concerns | No concerns | Low | ["Imprecision"] |

**Table S52** Grade Grading Evaluation Form of RHR

| **Comparison** | **Number of studies** | **Within-study bias** | **Reporting bias** | **Indirectness** | **Imprecision** | **Heterogeneity** | **Incoherence** | **Confidence rating** | **Reason(s) for downgrading** |
| --- | --- | --- | --- | --- | --- | --- | --- | --- | --- |
| HIIT:HIIT-NUT | 1 | Some concerns | Low risk | No concerns | Some concerns | Some concerns | No concerns | Very low | ["Within-study bias","Imprecision","Heterogeneity"] |
| HIIT:LICT | 1 | No concerns | Low risk | No concerns | Major concerns | No concerns | No concerns | Low | ["Imprecision"] |
| HIIT:MICT | 2 | Some concerns | Low risk | No concerns | No concerns | Some concerns | No concerns | Low | ["Within-study bias","Heterogeneity"] |
| HIIT:MIIT | 2 | Some concerns | Low risk | No concerns | Some concerns | No concerns | No concerns | Low | ["Within-study bias","Imprecision"] |
| HIIT:SOC | 3 | Some concerns | Low risk | No concerns | No concerns | Some concerns | No concerns | Low | ["Within-study bias","Heterogeneity"] |
| HIIT-NUT:MICT | 1 | No concerns | Low risk | No concerns | Some concerns | No concerns | No concerns | Moderate | ["Imprecision"] |
| LICT:MIIT | 1 | No concerns | Low risk | No concerns | Some concerns | No concerns | No concerns | Moderate | ["Imprecision"] |
| LICT:SOC | 2 | Some concerns | Low risk | No concerns | No concerns | Major concerns | No concerns | Very low | ["Within-study bias","Heterogeneity"] |
| LICT-NUT:MICT-NUT | 1 | Some concerns | Low risk | No concerns | Some concerns | Some concerns | No concerns | Very low | ["Within-study bias","Imprecision","Heterogeneity"] |
| LICT-NUT:SOC | 1 | No concerns | Low risk | No concerns | No concerns | No concerns | No concerns | High | [] |
| MICT-AT:SOC | 1 | Some concerns | Low risk | No concerns | No concerns | Some concerns | No concerns | Low | ["Within-study bias","Heterogeneity"] |
| MICT-NUT:SOC | 1 | Some concerns | Low risk | No concerns | Some concerns | No concerns | No concerns | Low | ["Within-study bias","Imprecision"] |
| MIIT:SOC | 2 | No concerns | Low risk | No concerns | Some concerns | Some concerns | No concerns | Low | ["Imprecision","Heterogeneity"] |
| HIIT:LICT-NUT | 0 | No concerns | Low risk | No concerns | Some concerns | No concerns | No concerns | Moderate | ["Imprecision"] |
| HIIT:MICT-AT | 0 | No concerns | Low risk | No concerns | No concerns | No concerns | No concerns | High | [] |
| HIIT:MICT-NUT | 0 | No concerns | Low risk | No concerns | No concerns | Some concerns | Some concerns | Low | ["Heterogeneity","Incoherence"] |
| HIIT-NUT:LICT | 0 | Some concerns | Low risk | No concerns | No concerns | No concerns | No concerns | Moderate | ["Within-study bias"] |
| HIIT-NUT:LICT-NUT | 0 | No concerns | Low risk | No concerns | Some concerns | Some concerns | No concerns | Low | ["Imprecision","Heterogeneity"] |
| HIIT-NUT:MICT-AT | 0 | Some concerns | Low risk | No concerns | No concerns | No concerns | No concerns | Moderate | ["Within-study bias"] |
| HIIT-NUT:MICT-NUT | 0 | Some concerns | Low risk | No concerns | No concerns | No concerns | No concerns | Moderate | ["Within-study bias"] |
| HIIT-NUT:MIIT | 0 | Some concerns | Low risk | No concerns | Some concerns | Some concerns | No concerns | Very low | ["Within-study bias","Imprecision","Heterogeneity"] |
| HIIT-NUT:SOC | 0 | No concerns | Low risk | No concerns | No concerns | No concerns | No concerns | Low | [] |
| LICT:LICT-NUT | 0 | Some concerns | Low risk | No concerns | Some concerns | No concerns | No concerns | Low | ["Within-study bias","Imprecision"] |
| LICT:MICT | 0 | Some concerns | Low risk | No concerns | No concerns | No concerns | No concerns | Moderate | ["Within-study bias"] |
| LICT:MICT-AT | 0 | Some concerns | Low risk | No concerns | Major concerns | No concerns | Some concerns | Very low | ["Within-study bias","Imprecision","Incoherence"] |
| LICT:MICT-NUT | 0 | Some concerns | Low risk | No concerns | No concerns | Major concerns | No concerns | Very low | ["Within-study bias","Heterogeneity"] |
| LICT-NUT:MICT | 0 | No concerns | Low risk | No concerns | Some concerns | No concerns | No concerns | Moderate | ["Imprecision"] |
| LICT-NUT:MICT-AT | 0 | Some concerns | Low risk | No concerns | Some concerns | No concerns | Some concerns | Very low | ["Within-study bias","Imprecision","Incoherence"] |
| LICT-NUT:MIIT | 0 | No concerns | Low risk | No concerns | Some concerns | Some concerns | No concerns | Low | ["Imprecision","Heterogeneity"] |
| MICT:MICT-AT | 0 | Some concerns | Low risk | No concerns | No concerns | No concerns | No concerns | Moderate | ["Within-study bias"] |
| MICT:MICT-NUT | 0 | Some concerns | Low risk | No concerns | Some concerns | No concerns | No concerns | Low | ["Within-study bias","Imprecision"] |
| MICT:MIIT | 0 | No concerns | Low risk | No concerns | No concerns | No concerns | No concerns | High | [] |
| MICT:SOC | 0 | Some concerns | Low risk | No concerns | Some concerns | Some concerns | No concerns | Very low | ["Within-study bias","Imprecision","Heterogeneity"] |
| MICT-AT:MICT-NUT | 0 | Some concerns | Low risk | No concerns | No concerns | No concerns | No concerns | Moderate | ["Within-study bias"] |
| MICT-AT:MIIT | 0 | No concerns | Low risk | No concerns | Some concerns | No concerns | No concerns | Moderate | ["Imprecision"] |
| MICT-NUT:MIIT | 0 | Some concerns | Low risk | No concerns | Some concerns | No concerns | No concerns | Low | ["Within-study bias","Imprecision"] |

**Figure 1** The risk of bias summery graph according to Cochrane risk of bias tool for randomized trials (RoB2).

**
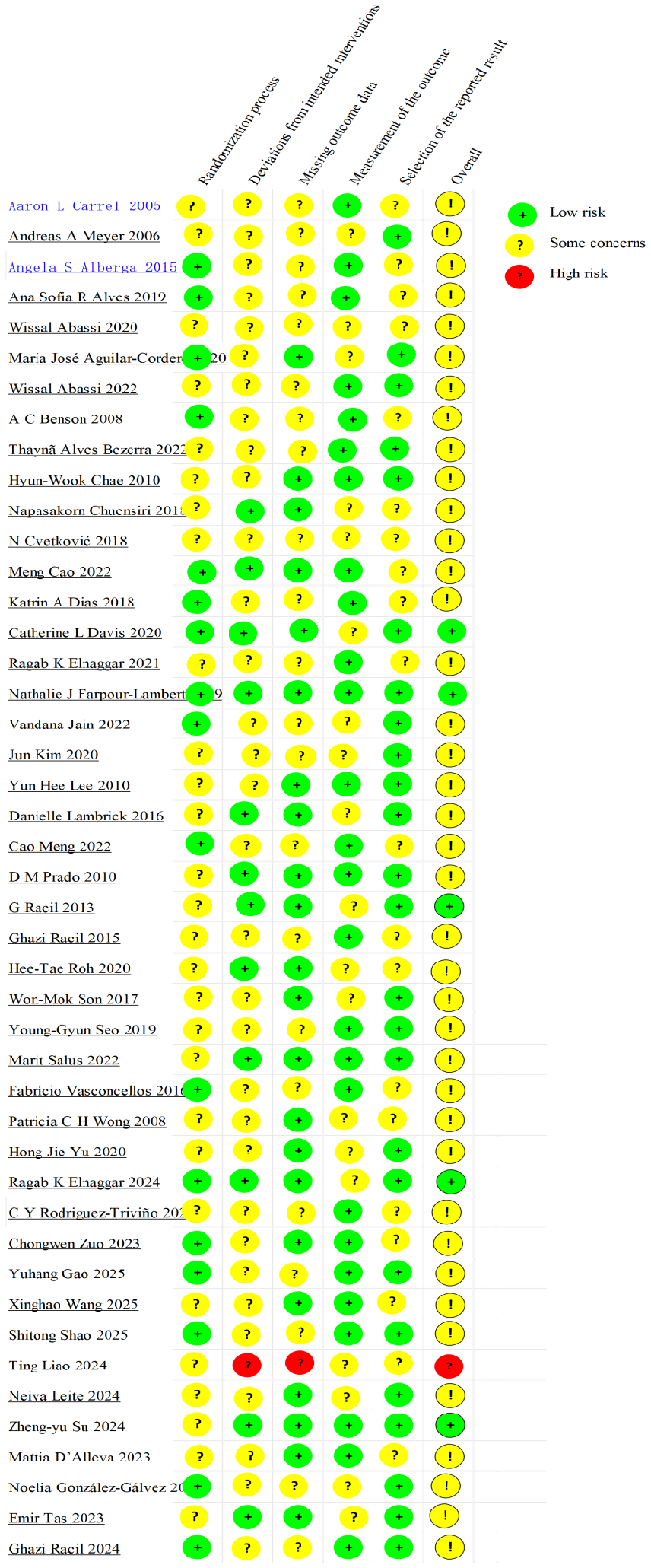
**

**Figure 2** Loop Inconsistency Diagram of the Outcome Measure VO_2_peak

**
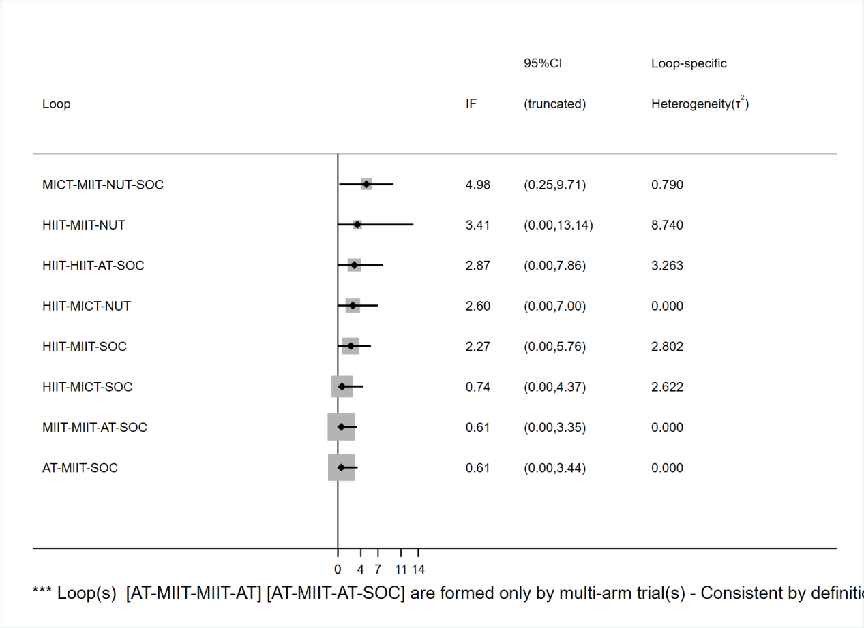
**

**Figure 3** Loop Inconsistency Diagram of the Outcome Measure VO_2_max

**
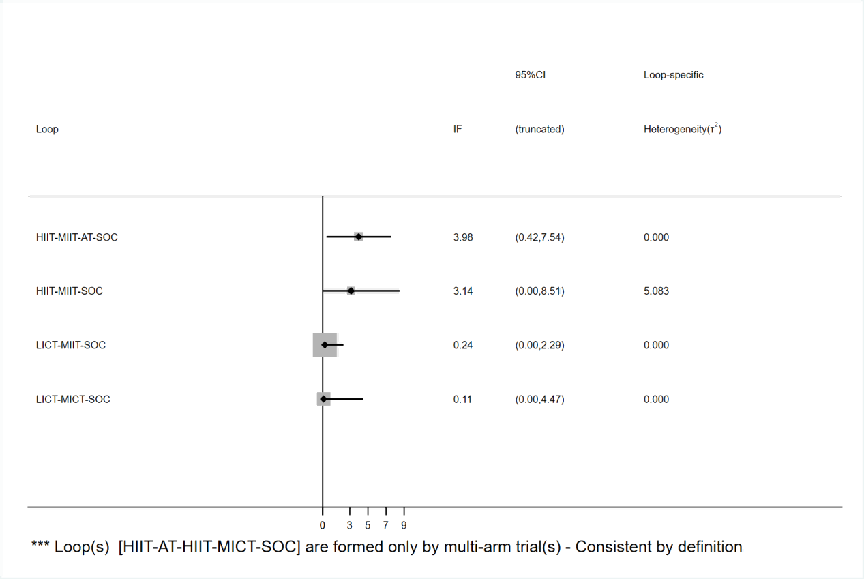
**

**Figure 4** Loop Inconsistency Diagram of the Outcome Measure HR_max_

**
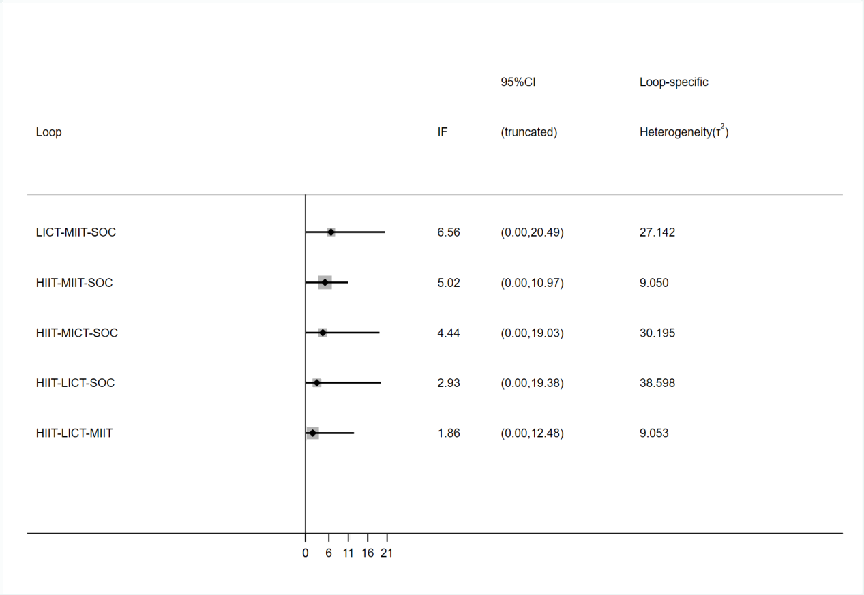
**

**Figure 5** Loop Inconsistency Diagram of the Outcome Measure SBP

**
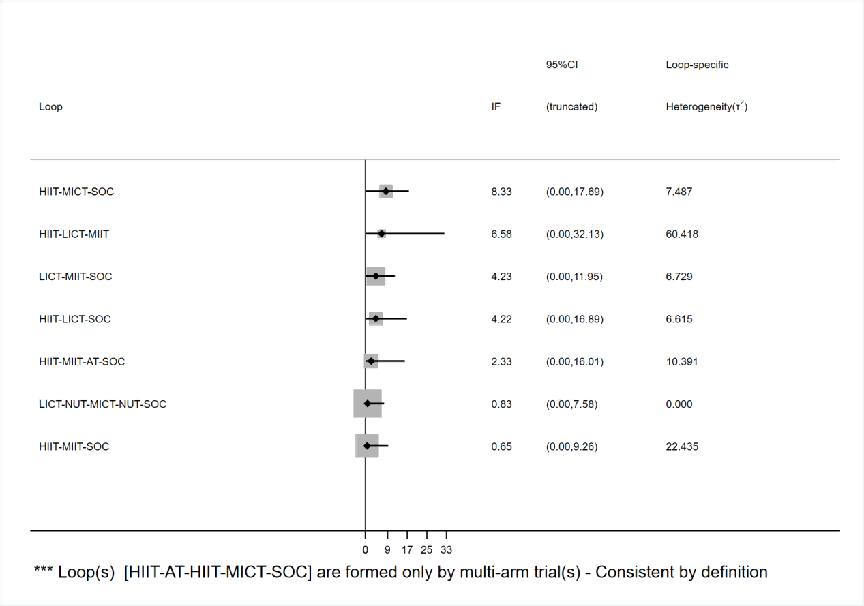
**

**Figure 6** Loop Inconsistency Diagram of the Outcome Measure DBP

**
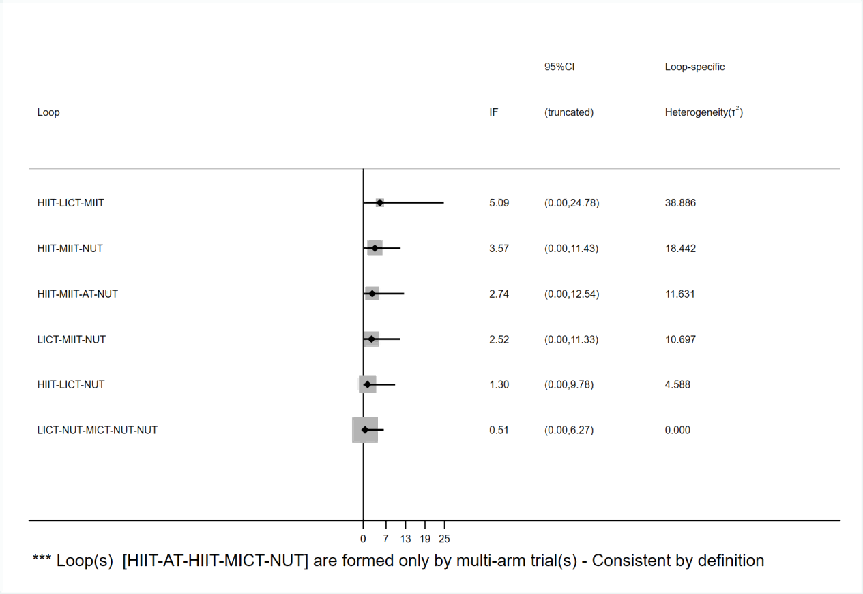
**

**Figure 7** Loop Inconsistency Diagram of the Outcome Measure MAS


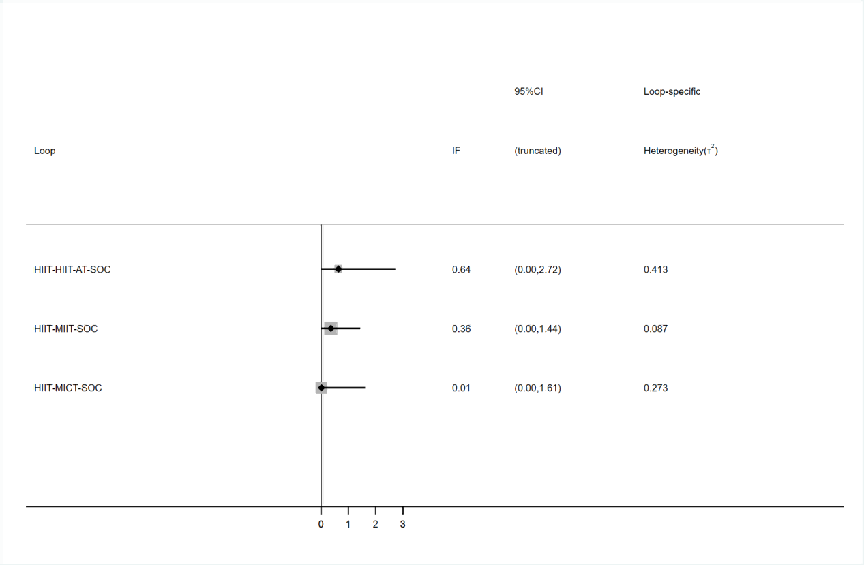


**Figure 8** Loop Inconsistency Diagram of the Outcome Measure RHR

**
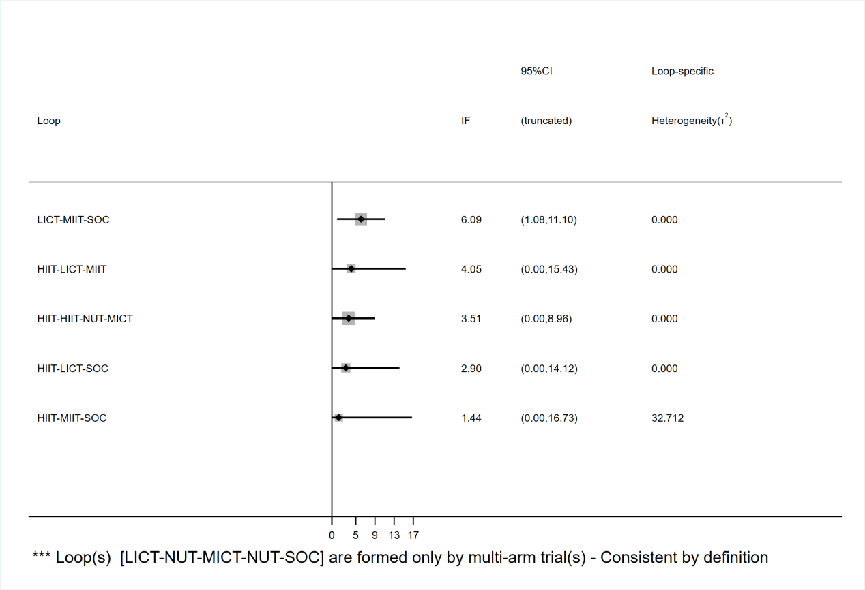
**

**Figure 9** Funnel Plot of the Outcome Measure VO_2_peak

**
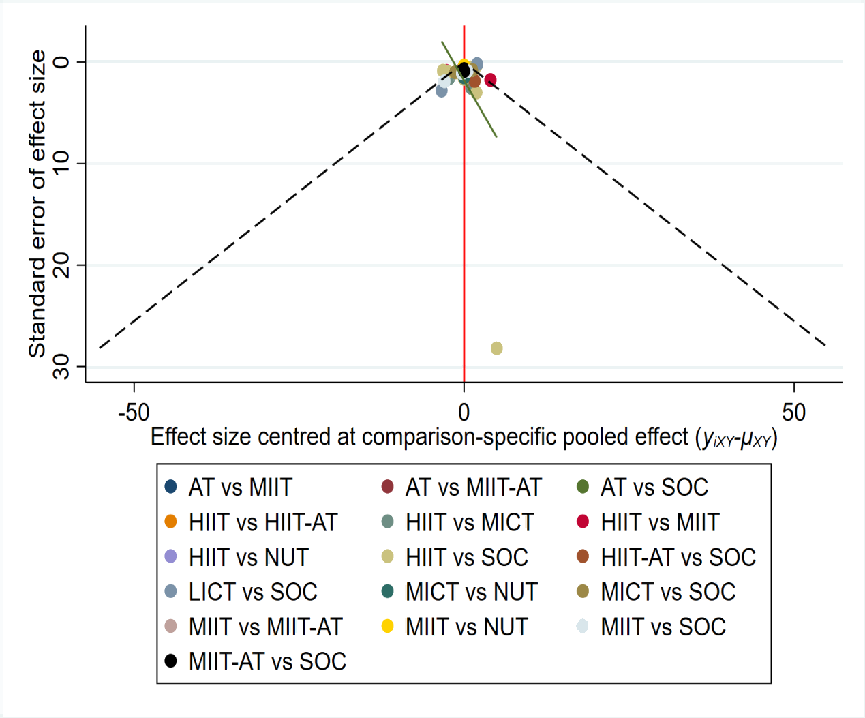
**

**Figure 10** Funnel Plot for the Outcome Measure VO_2_max

**
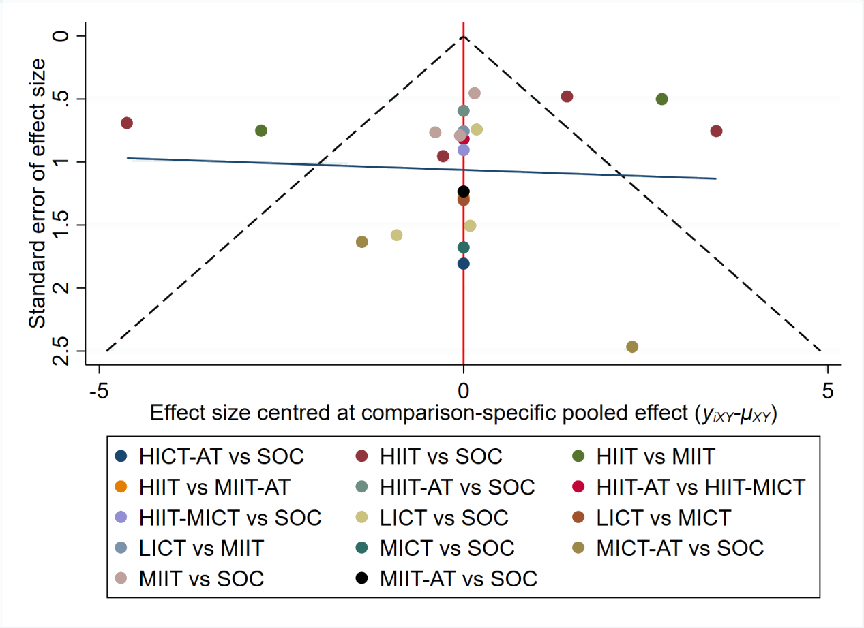
**

**Figure 11** Funnel Plot of the Outcome Measure HR_max_

**
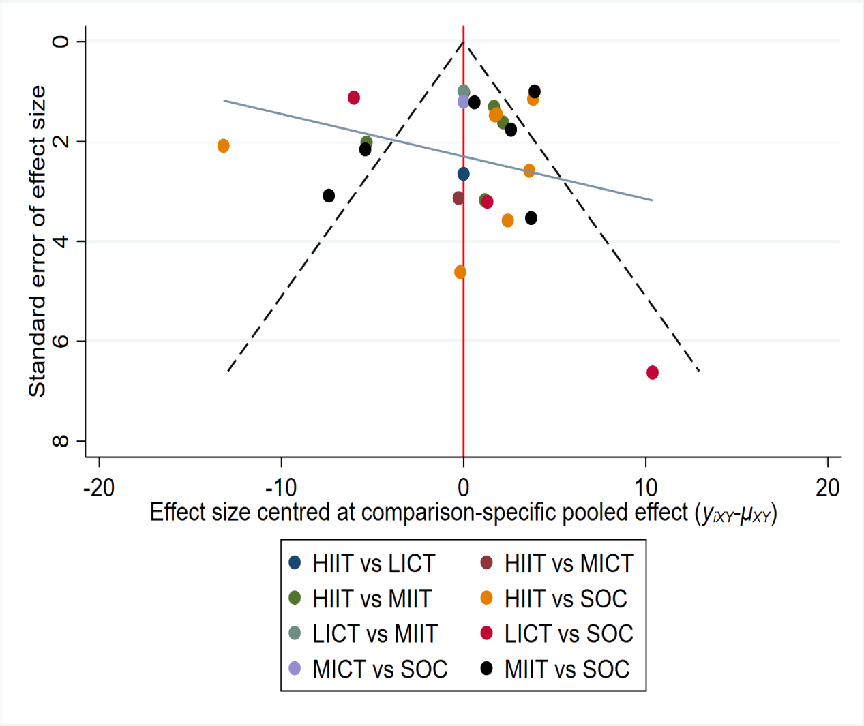
**

**Figure 12** Funnel Plot of the Outcome Measure SBP

**
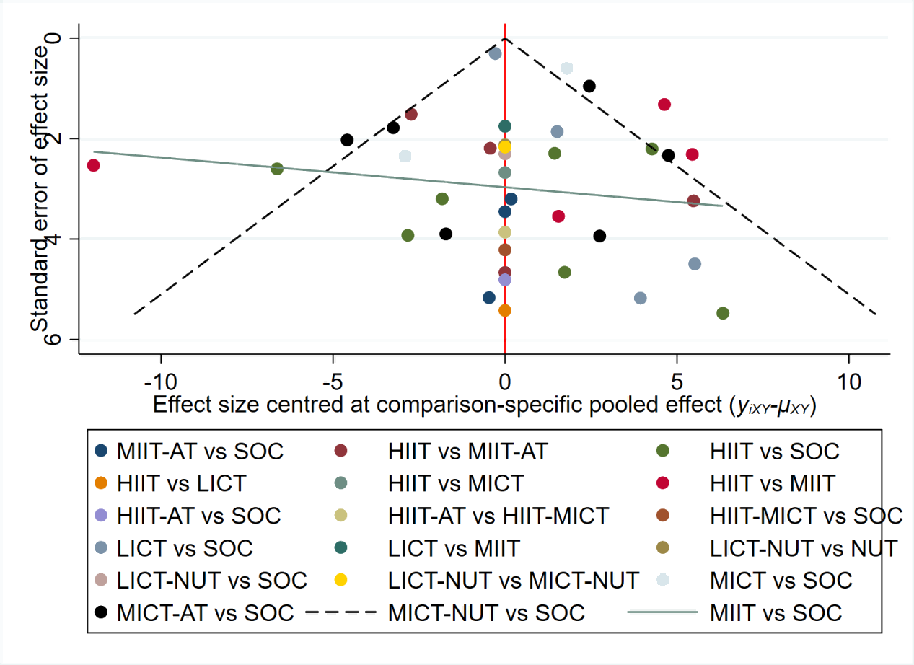
**

**Figure 13** Funnel Plot of the Outcome Measure DBP

**
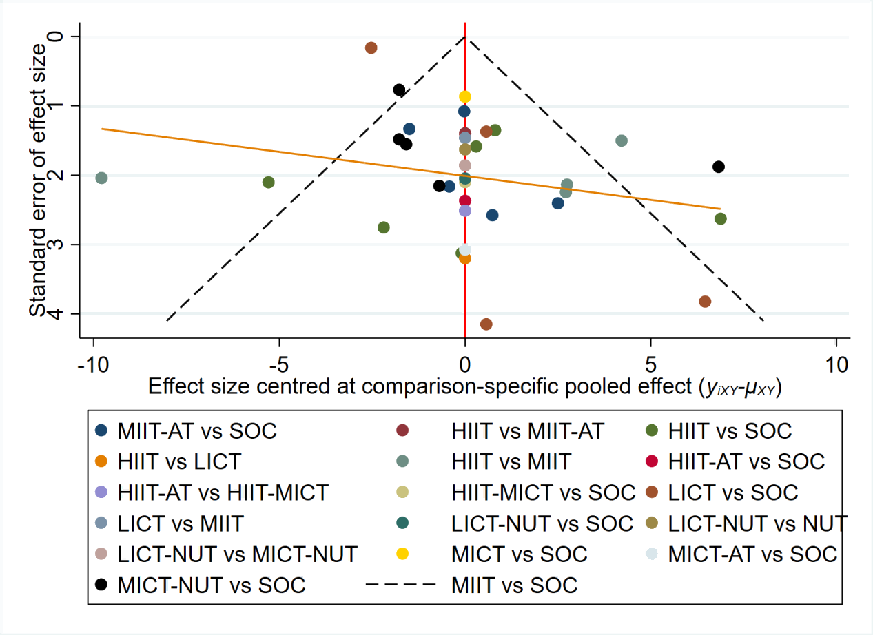
**

**Figure 14** Funnel Plot of the Outcome Measure MAS

**
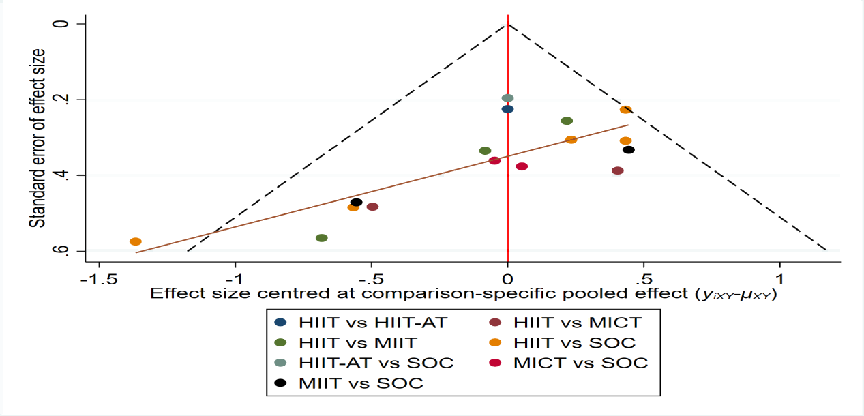
**

**Figure 15** Funnel Plot of the Outcome Measure RHR


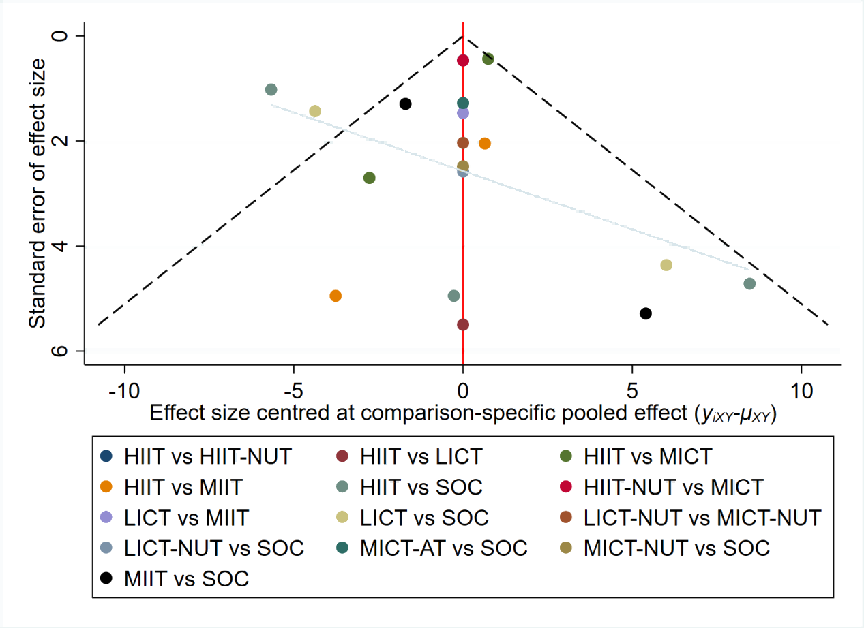

Supplement: Supplementary file 1 [file Table_1.docx]
